# Photoactivatable Electrophilic Glycosylselenosulfonates for Ultrafast Modification of Alkynes and Thiols

**DOI:** 10.1021/acscentsci.5c00650

**Published:** 2025-07-07

**Authors:** Weitao Yan, Wenchao Liu, Qinshuo Zhang, Wentao Lin, Yujie Liao, Yiqun Geng, Ruo Wang, Chunfa Xu

**Affiliations:** ⊥ Key Laboratory of Molecular Synthesis and Functionalization Discovery, College of Chemistry, 12423 Fuzhou University, Fuzhou, 350108, China; ‡ Key Laboratory of Organofluorine Chemistry, Shanghai Institute of Organic Chemistry, Chinese Academy of Sciences, Shanghai 200032, China; § State Key of Laboratory of Bioactive Substance and Function of Natural Medicine, Institute of Materia Media, Chinese Academy of Medical Sciences and Peking Union Medical College, Beijing 100050, China; ∥ Shengli Clinical Medical College of Fujian Medical University, Department of Breast Surgery, Fujian Provincial Hospital, Fuzhou University Affiliated Provincial Hospital, Fuzhou 350001, China

## Abstract

Glycosylseleno scaffolds exhibit wide-ranging applications in multidisciplinary fields, particularly in drug discovery and biophysical chemistry, where they serve as valuable tools for biomolecular structural analysis. However, efficient methods toward glycosylseleno scaffolds remain underexplored. Herein, we present the design of a novel class of bench-stable reagents, glycosylseleno­sulfonates, which uniquely integrate radical reactivity with electrophilic properties, thereby facilitating the straightforward incorporation of glycosylseleno moieties under mild reaction conditions. Upon photoirradiation, the radical addition of alkynes with glycosylseleno­sulfonates proceeds at an exceptionally fast rate, achieving completion in less than 1 min. Likewise, the functionalization of cysteine-containing molecules is achieved in a comparably short time frame, typically within 1 min in most instances. Additive experiments involving various amino acids confirm the robustness of these transformations, demonstrating consistently high efficiency across diverse reaction environments with negligible interference. Importantly, successful peptide and protein labeling in aqueous conditions highlights the method’s potential for bioorthogonal applications. These findings collectively underscore the broad applicability and operational simplicity of glycosylseleno­sulfonates in developing rapid and efficient labeling techniques for biological and chemical research. This work not only advances synthetic methodologies for glycosylseleno scaffolds but also opens new avenues for functional studies of complex biological systems.

## Graphical abstract

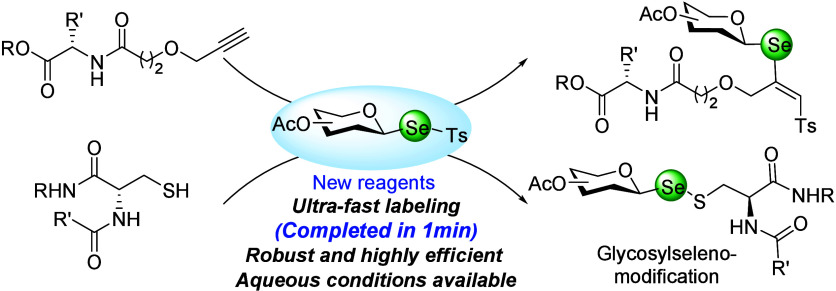

## Introduction

Glycosylseleno-based conjugates represent a leading class of glycomimetics, in which the oxygen atom linked to the anomeric carbon is replaced with a selenium atom.^1^ In comparison to normal *O*-congeners, glycosylseleno conjugates offer several advantages. First, selenium’s natural isotopic distribution includes ^77^Se, which, with an abundance of 7.6%, serves as an excellent NMR-active nucleus (Figure 1A).^2,3^ Thus, glycosylseleno compounds can be used as powerful probes to investigate the molecular structure–function relationship of carbohydrates in essential life processes through ^77^Se NMR spectroscopy. Second, selenium’s pronounced heavy-atom effects and anomalous diffraction signals^4,5^ greatly facilitate the structural determination of carbohydrate–protein complexes with improved resolution (Figure 1B). Third, glycosylseleno conjugates are inert to glycosidases in vivo, rendering them highly metabolically stable as potential drug candidates, thus promising elevated bioavailability (Figure 1C).^6–9^ Furthermore, the larger atomic radius of selenium induces the distinct bond lengths and angles in selenoglycosides compared to *O*-glycosides, resulting in unique spatial structures that enable specific binding (Figure 1D).^10^ Collectively, these unique properties confer distinctive advantages upon glycosylseleno conjugates in biophysical detection techniques such as anomalous diffraction and nuclear magnetic resonance. Not only do they serve as valuable tools for deciphering sugar complex structures, but they also hold significant promise as candidate molecules in drug development endeavors. Nevertheless, despite these prominent advantages, the widespread utilization of glycosylseleno conjugates has been hampered by synthetic challenges.^7–9,11–16^ To overcome this limitation, the straightforward synthesis of glycosylseleno conjugates with precise stereocontrol and structural diversity is imperative.

**Figure 1:**
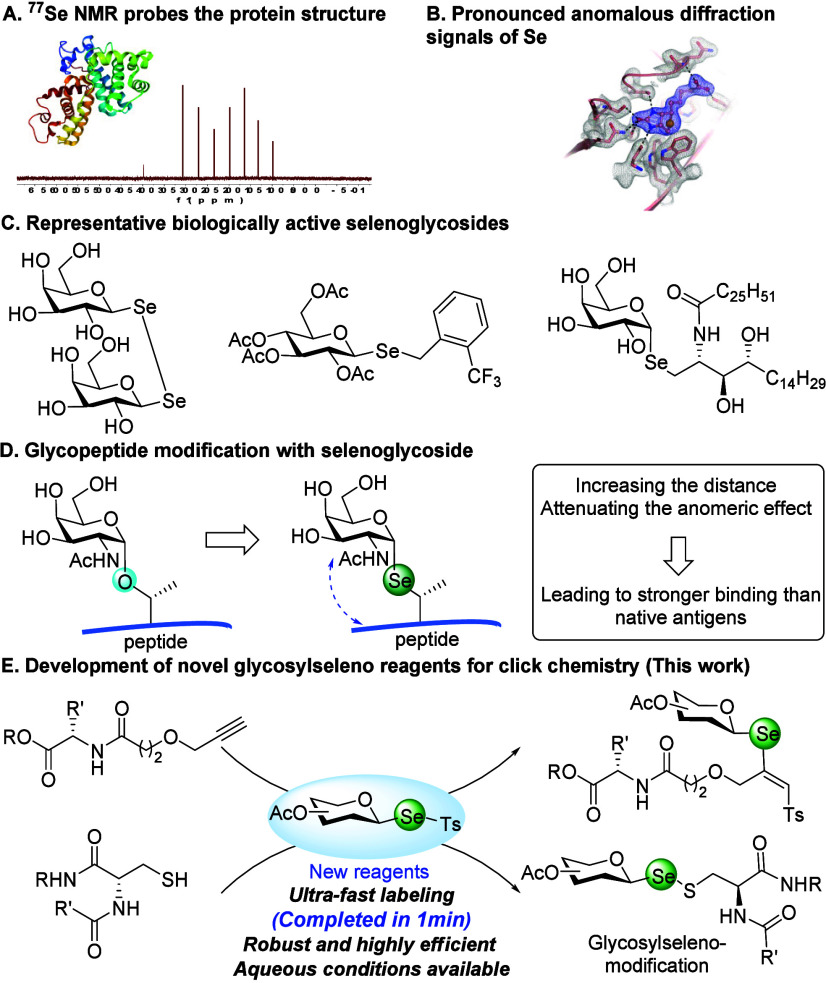
Applications of glycosylseleno conjugates.

Click chemistry has emerged as a powerful and versatile tool in modern organic synthesis, characterized by its modularity, unparalleled regioselectivity, excellent yields, and remarkable substrate tolerance as well as rapid reaction rates.^17–19^ This powerful synthetic paradigm enables the efficient assembly of complex molecules, making it invaluable across diverse fields such as drug discovery, bioconjugation, and material science.^20–27^ With the increasing demand for sophisticated bioconjugates and labeling strategies, particularly in biological applications, the development of new reagents and methodologies for bioorthogonal click chemistry has become increasingly imperative.^28–35^ These reactions must be biocompatible and able to be conducted in approximately physiological environments. Moreover, reactions with ultrarapid kinetics are preferred, as they enable real-time applications in biological contexts, facilitating dynamic studies of biomolecular interactions and functions. Given the unique advantages of glycosylseleno conjugates in structural biology and biophysical analysis, we envisioned that a rapid glycosylseleno labeling method would serve as a potential tool for addressing intricate biological questions, ultimately enhancing our understanding of complex biological processes (Figure 1E).^36^

To date, the predominant reagents for glycosylseleno-unit incorporation have included glycosyl isoselenuronium salt **1**, selenocyanate **2**, and selenobenzoate **3**, which were pioneered by Szilágyi,^37^ Misra,^38^ and Ishihara,^39^ respectively. These reagents had been demonstrated, in a limited way, to functionalize substrates such as primary alkyl halides, anhydrides, and activated aromatic fluorides via nucleophilic reactions, as well as aromatic iodides through transition-metal-catalyzed cross-coupling reactions.^40,41^ However, existing methods fall short of the criteria required for bioorthogonal chemistry. Notably, Davis et al. introduced a method for the site-specific incorporation of glycosylseleno moieties at cysteine residues using diselenide **4** (Figure 2A).^42,43^ While this method represents a significant advancement in the field, its practical utility is limited by relatively long reaction times, rendering it less suitable for real-time detection. As a result, there is a demand for the development of novel reagents capable of rapidly transferring glycosylseleno units to a broad spectrum of structurally diverse substrates, meeting the stringent requirements of bioorthogonal chemistry.

**Figure 2:**
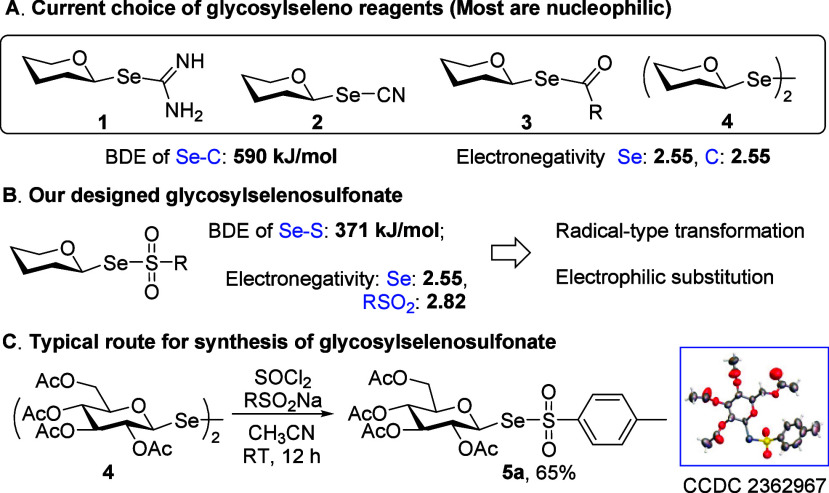
Glycosylseleno reagents and our design and synthesis.

Current glycosylseleno reagents predominantly (**1**, **2**, and **3**) rely on a carbon-centered leaving group attached to selenium. However, this structural motif is associated with a relatively high bond dissociation energy (BDE) and limited bond polarization,^44–47^ which significantly diminish reactivity. Moreover, the diselenide **4**, featuring a nonpolar covalent Se–Se bond, has shown limited reactivity in practical applications thus far.^42^ Recently, we demonstrated that glycosylthiosulfonate readily couples with boronic acids to give aryl thioglycosides,^48^ highlighting the superiority of the sulfonyl group-based reagents.^49–52^ Inspired by this finding and motivated by ongoing interests in carbohydrate chemistry^53–57^ and the development of innovative reagents for rapid incorporation of glycosylseleno moieties, we propose substituting the carbon-centered leaving group with a sulfonyl group. The sulfonyl group offers higher electronegativity^44^ and a lower Se–S BDE (Figure 2B),^45^ enabling versatile transformations. Its polarized Se–SO_2_ bond facilitates electrophilic substitution, while the low BDE supports radical functionalization.^58–60^ This modification holds promise not only for expanding the structural diversity of selenoglycosides but also for rapidly labeling biomolecules.

## Results and Discussion

### Synthesis of Glycosylselenosulfonate

Our investigation began with the synthesis of glycosylselenosulfonate **5**. Initially, we treated the nucleophilic-type reagent **3** with tosyl chloride (TsCl) in the presence of bases such as Cs_2_CO_3_ and piperidine but failed to obtain the desired product. We then investigated the reaction between the sulfinate and glycosyl diselenide **4**, which is proposed to in situ generate a glycosylselenium cation via cleavage of the Se–Se bond upon activation with SOCl_2_.^61,62^ Pleasingly, this approach successfully produced a series of aryl sulfinate-derived glycosylseleno­sulfonates **5**. Other activators, including *N*-bromosuccinimide (NBS) and iodine, were also tested but resulted in diminished yields due to significant selenium precipitation (see Table S2). Specifically, the 2,3,4,6-tetra-acetylglucosyl diselenide **4a** was converted to glycosylseleno­sulfonate **5a** ^63^ with a 65% isolated yield using SOCl_2_ in CH_3_CN at room temperature over 12 h (Figure 2C). The structure of **5a** was unambiguously determined by X-ray diffraction (CCDC: 2362967). Under identical conditions, glycosylseleno­sulfonates derived from galactose, arabinose, xylose, and maltose were successfully synthesized, highlighting the broad applicability of this methodology.

### Photocatalytic Radical Addition of Alkynes with Glycosylseleno­sulfonate

With these newly developed reagents in hand, we performed a variety of reactions to demonstrate their excellent reactivity. Vinyl sulfones are widely prevalent in pharmaceuticals and bioactive molecules, serving as key organic building blocks.^64,65^ The radical addition of alkynes offers a straightforward and atom-economical methodology for constructing diverse functionalized vinyl sulfones, while enabling the synthesis of selenoglycosides simultaneously.^66^ Therefore, we initiated our investigation into the reaction between **5a** and alkyne **6a**, as shown in Table 1.

**Table 1:**
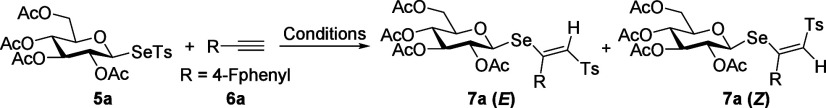

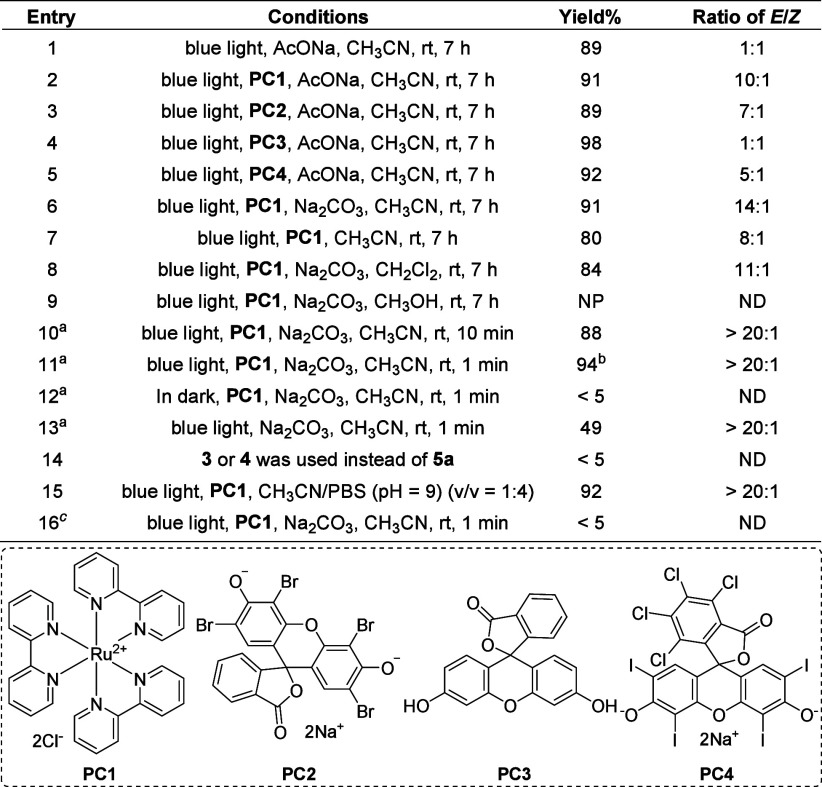
Optimization for Photocatalytic Addition with Alkynes

Reaction conditions: **6a** (0.20 mmol, 2.0 equiv), **5a** (0.10 mmol, 1.0 equiv), solvent (1 mL), catalyst (2 mol%), base (0.1 mmol, 1.0 equiv), blue LEDs. Yields and *E*/*Z* ratios were determined by ^1^H NMR using 1,3,5-trimethoxybenzene as an internal standard.

a **6a** (0.12 mmol), **5a** (0.10 mmol).

b Isolated yield.

c Reaction was performed in air. ND = not determined.

Upon irradiation with blue light, the glycosylseleno and tosyl groups, two fragments of **5a**, were readily installed into the triple bond, yielding the potentially bioactive glycosylseleno-based vinyl sulfone with a good yield but low stereoselectivity (Table 1, entry 1). This result demonstrated the reagent’s radical nature, distinguishing it from the commonly used nucleophilic reagents.^37–41^ Given the established role of photocatalysts in enhancing both efficiency and selectivity in radical functionalization,^67–69^ we evaluated several photocatalysts. To note, the addition of tris­(2,2′-bipyridyl)­ruthenium­(II) chloride **PC1** significantly improved the *E*/*Z* ratio (Table 1, entry 2). While other photocatalysts including Eosin Y (**PC2**), Fluorescein (**PC3**), and Rose Bengal (**PC4**) also influenced the selectivity, none outperformed **PC1** (Table 1, entries 3–5). Using Na_2_CO_3_ as a base instead of AcONa slightly improved the outcome (Table 1, entry 6), whereas the absence of a base led to diminished yield and selectivity (Table 1, entry 7). Employing dichloromethane as the solvent led to a lower yield (Table 1, entry 8), and using MeOH caused complete decomposition of **5a** without product formation (Table 1, entry 9). Further examination of the reaction kinetics revealed that the transformation reached completion within 10 min, providing **7a** in good yield and excellent *E*-selectivity (Table 1, entry 10). Even at 1 min, the product was obtained in good quantity (Table 1, entry 11). A control reaction conducted in the dark completely halted product formation, underscoring the necessity of light activation (Table 1, entry 12). Additionally, performing the reaction without a photocatalyst resulted in a moderate yield, emphasizing **PC1**’s critical role in enhancing the reaction efficiency (Table 1, entry 13). Finally, replacing **5a** with established glycosylseleno reagents such as **3** or **4** failed to produce any product, further demonstrating the unique reactivity of the glycosylseleno­sulfonate reagent (Table 1, entry 14). Pleasingly, the reaction carried out in CH_3_CN/PBS exhibited remarkable performance, delivering both excellent yield and high selectivity (Table 1, entry 15), making this approach a highly promising strategy for further exploration in biochemical applications. Notably, this photocatalyzed transformation was exceptionally sensitive to oxygen. Under aerobic conditions, the reaction failed to proceed, with the starting materials remaining largely unchanged (Table 1, entry 16).

Armed with the optimal conditions, our focus shifted toward exploring the generality of this method. As shown in Scheme 1, this radical approach demonstrated remarkable insensitivity to electronic property and steric hindrance, exhibiting broad compatibility for both terminal and internal alkynes. Arylalkynes featuring a variety of functional groups, involving halogens, cyano, aldehyde, and methoxy groups, participated efficiently in this protocol, delivering products with excellent stereoselectivity (**7a**–**f**). The reason for the relatively lower yield of the cyano-containing product **7c** may be that the radical can add to the carbon–nitrogen triple bond of the aryl nitrile,^70,71^ thus competing with its addition to the carbon–carbon triple bond. Heteroaryl alkynes containing thiophene and pyridine units were also well-tolerated, yielding the desired products (**7g**–**h**). Encouraged by these results, we evaluated the reactivity of glycosylseleno donors derived from different sugars. Donors based on galactose, arabinose, xylose, and maltose effectively transferred the glycosylseleno unit to alkynes with high selectivity (**7i**–**l**). Internal alkynes exhibited excellent regioselectivity and stereoselectivity under the reaction conditions (**7m**–**n**). Reaction with conjugated 1,3-enyne selectively functionalized the triple bond, leading to the production of a thermally stable 1,3-diene product (**7o**). Additionally, alkyl-substituted terminal alkynes proved effective in this protocol (**7p**–**s**), with no significantly adverse impact from the presence of a chloro group (**7q**). Electronically biased ethyl propiolate also reacted smoothly, delivering the product in a regioselective and stereocontrolled manner (**7r**). Notably, the reaction with a cyclopropane-substituted alkyne, which is typically prone to produce an allene product,^72^ exclusively furnished the addition product (**7s**). This observation suggests that termination of the active vinyl intermediate outpaces cyclopropane ring-opening. To further demonstrate the potential of this protocol for biomolecule labeling, we synthesized an amino acid-based alkyne via a condensation reaction with propargyl-NHS.^73^ The incorporation of the glycosylseleno unit under standard conditions proceeded smoothly, yielding glycosylseleno-labeled products (**7t**–**z**), thereby underscoring the versatility of our method for biological applications. Importantly, this two-step strategy was successfully applied to the labeling of insulin, resulting in the detection of the glycosylseleno-labeled product. This outcome underscores the potential of the glycosylselenosulfonate reagents for application in biomacromolecule labeling (Figure S15).

**Scheme 1:**
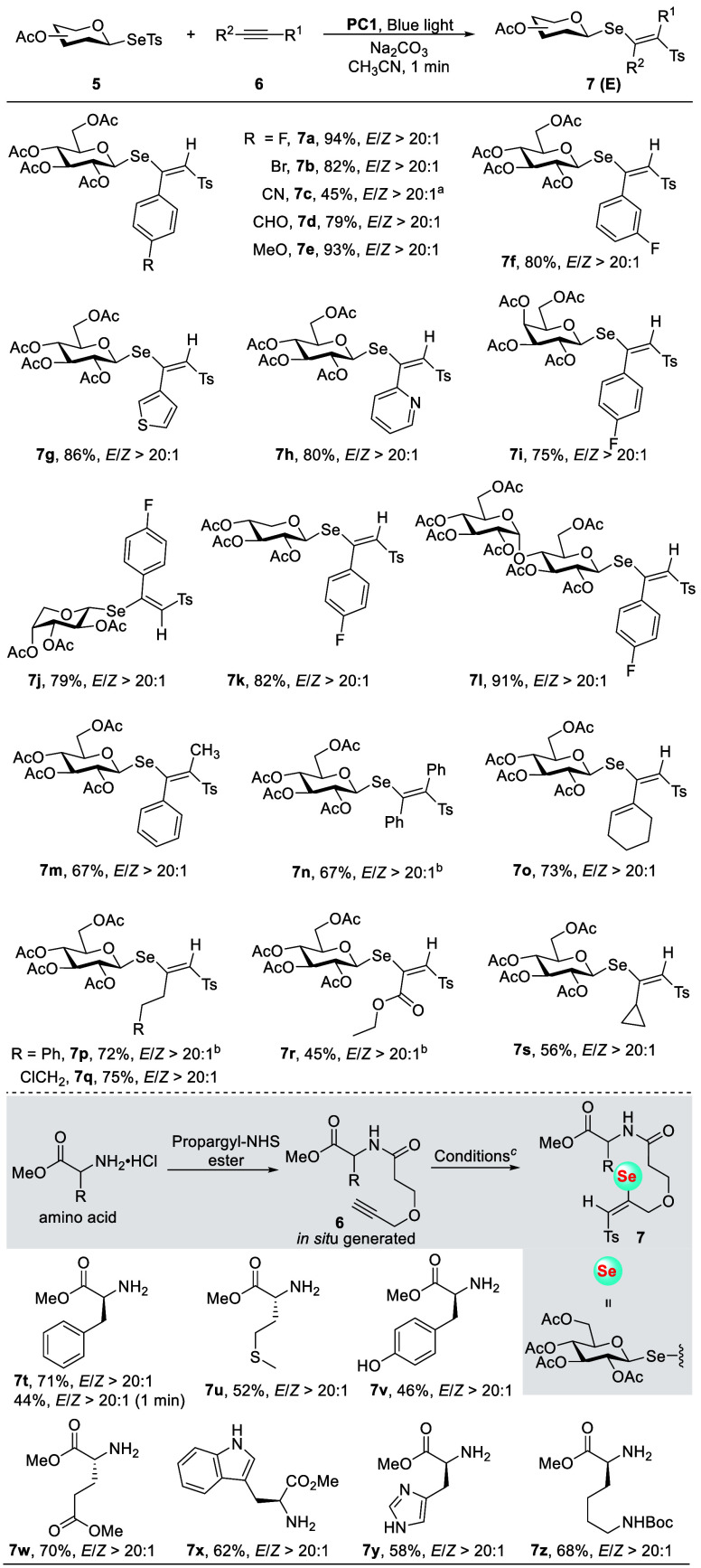
Reaction Scope for Photocatalytic Addition

Reaction conditions: **5** (0.10 mmol, 1.0 equiv), **6** (0.12 mmol, 1.2 equiv), CH_3_CN (1 mL), Ru­(bpy)_3_Cl_2_·6H_2_O (**PC1**) (2 mol%), Na_2_CO_3_ (0.10 mmol, 1.0 equiv), 450–465 nm LED, 1 min, N_2_. Isolated yields. ^*a*^1 h. ^*b*^10 min. ^*c*^PBS (pH = 9):CH_3_CN (4:1) as solvent, **5a** (2.0 equiv), 30 min.

To further probe the tolerance of this radical addition reaction, we conducted a series of experiments using additives bearing diverse functional groups, including phenols, alcohols, carboxylic acids, amines, amides, amino acids, alkenes, and thiols (Scheme S1). Remarkably, the presence of hydroxyl, amino, carboxyl, and amide groups exerted a negligible influence on the reaction outcome. This robustness strongly supports the method’s potential for rapid glycosylselenylation of biomolecules in complex environments. Interestingly, no product formation was observed when introducing styrene, suggesting that alkenes exhibit significantly higher reactivity than alkynes under these conditions, consistent with the established trends in addition reactions involving unsaturated bonds. Given the potential formation of multiple isomers with alkene substrates, we defer further investigation of these reactions. In contrast, the introduction of thiols had a pronounced effect on the reaction. Specifically, using a sterically hindered thiol, such as *tert*-butyl thiol, resulted in the formation of the desired product with a yield of 27%. However, when thiophenol was employed as an additive, the addition reaction was completely suppressed.

### Glycosylselenylation of Cysteine-Containing Molecules

Building on the seminal results of radical addition reactions with thiol additives (Scheme S1), we hypothesized that our newly developed glycosylseleno­sulfonate reagents, characterized by their high electrophilicity, would rapidly and efficiently modify thiol groups. To test this hypothesis, we investigated the reactions with a range of cysteine-based peptides under mild conditions (Scheme 2 and Table S10). The reaction proceeded efficiently with DMSO identified as the optimal solvent. Furthermore, when the reaction was conducted under dark conditions, a nearly quantitative yield was also obtained. This result suggests that the reaction process with the thiol group differs from the addition reaction and is more likely to proceed via a nucleophilic substitution process. Using a PBS buffer mixture yielded comparable outcomes, indicating the method’s robustness. Substrate scope studies revealed that all tested glycosylseleno­sulfonates efficiently labeled cysteine substrates (**9a**–**e**). Notably, a variety of cysteine-containing peptides could also be labeled successfully (**9f**–**i**). To further demonstrate the robustness of this protocol, we examined the modification of oligopeptides in a DMSO–PBS mixture. Remarkably, the reaction for synthesis of peptide modification (**9j**) was completed within just 5 min using 4 equiv of glycosylseleno­sulfonate **5a**. Furthermore, oxytocin, following TCEP reduction of its disulfide bond to completely expose the thiol groups, reacted with reagent **5a** to afford diglycosylseleno-labeled product **9k**. These results collectively highlight the method’s potential for diverse applications in biomolecule labeling and beyond.

**Scheme 2:**
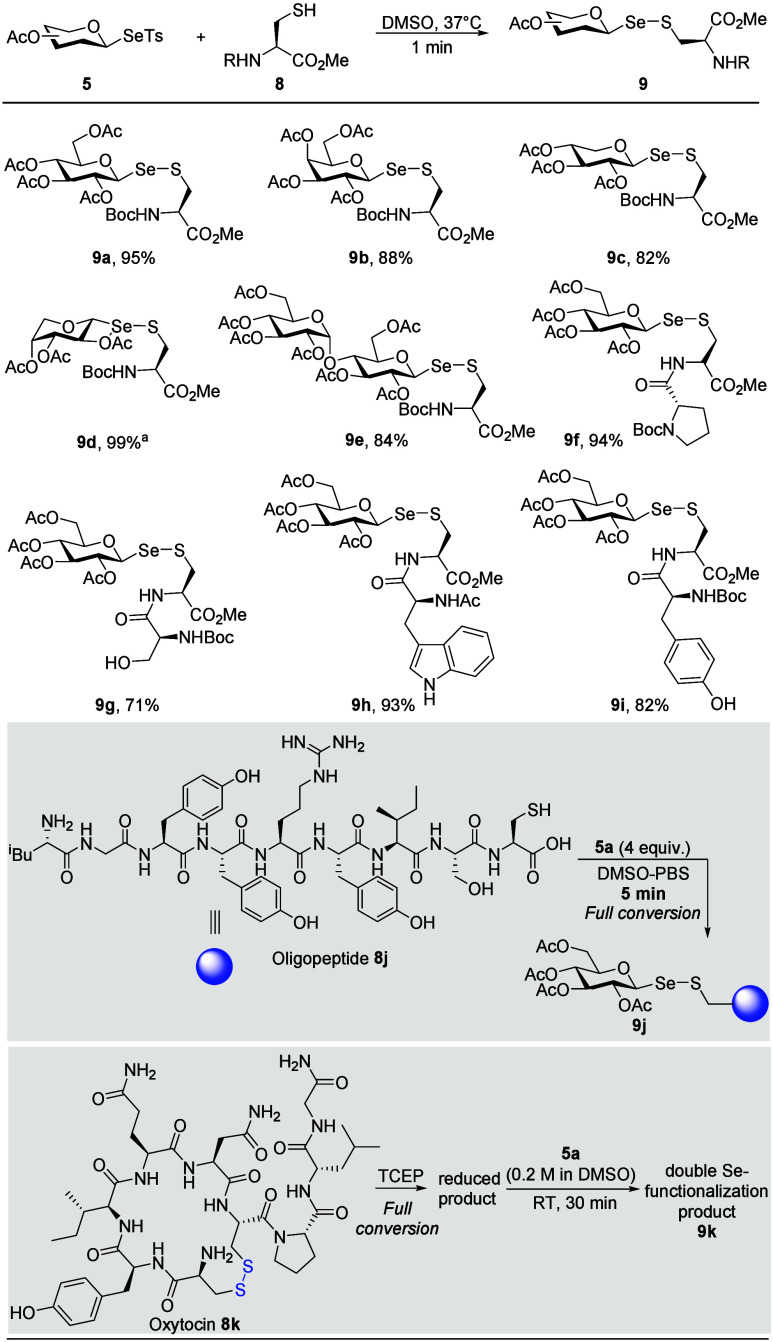
Reaction Scope for Cysteine-Containing Molecules

Reaction conditions: **5** (0.1 mmol), **8** (0.1 mmol), DMSO (2 mL), air, 1 min. Isolated yields. ^*a*^ **5** (1.5 equiv). ^*b*^Reaction condition: CH_3_CN (2 mL).

To further assess the tolerance of the cysteine-based glycosylselenylation method, we conducted additive experiments, focusing on the influence of amino acids (Scheme S2). Acidic and neutral amino acids had little to no effect on the reaction efficiency, while the presence of tyrosine caused a slight decrease in the yield. Basic amino acids such as ornithine and arginine moderately reduced the reaction efficiency under the standard conditions. Encouragingly, adjusting the solution pH using a DMSO–PBS buffer system significantly improved the modification efficiency, demonstrating the method’s adaptability. These findings highlight the potential for fine-tuning the reaction conditions to accommodate challenging substrates. Interestingly, reagent **5a** did not react with the exposed ε-NH_2_ of lysine under the same conditions, further highlighting the site-selectivity for the thiol group.

The radical addition reaction could proceed through either a bimolecular homolytic radical functionalization pathway (S_H_2) or a single electron transfer (SET) pathway (Figure S6).^74–76^ To elucidate the mechanism, several experiments were conducted (Figure 3). First, a radical trapping experiment using stoichiometric TMS_3_SiH^77^ under standard conditions yielded the protonated byproduct **10** in 30% yield, alongside the formation of the selenoglycoside **7a** in 54% yield (Figure 3A). This suggests that the terminal formation of the C–Se bond likely occurs via a radical process. Additionally, the reaction was completely inhibited by the addition of TEMPO, further supporting its radical nature (Figure 3A). Next, the addition of exogenous nucleophiles, such as sodium methoxide or potassium selenocyanate, did not yield the corresponding products **11** (Figure 3B), ruling out vinyl cation intermediates and excluding the SET pathway (Figure S6). Crossover experiments with equimolar amounts of **5b** and **5e** (Figure 3C) revealed HRMS signals for all four addition products, reinforcing the radical character of the reaction (Figure S7). Furthermore, the reaction of **5a** with butylated hydroxytoluene **12** (BHT) in the absence of alkyne was performed. Significant amounts of product **13** resulting from the combination of Ts radical with BHT, along with a large quantity of diselenide **4a**, were detected (Figure 3D). This experimental result is consistent with the former observations. Given the well-established role of photoinduced energy transfer in alkene isomerization,^78–81^ a photoisomerization study was also performed (Figure 3E). Prolonged blue-light irradiation (7 h) converted the *E* isomer into its *Z* congener both with and without a photocatalyst, whereas 1 min irradiation caused no isomerization. Based on these findings, a plausible mechanism via the S_H_2 pathway is proposed (Figure 3F). Upon photoirradiation, the photolabile Se–S bond of **5** undergoes homolytic cleavage, generating a seleno-centered radical **14** and a sulfonyl radical **15**. These radicals react with alkynes to form C–Se and C–S bonds in a regio- and stereocontrolled fashion, yielding the *E*-configured products **7**.^82–84^ Prolonged irradiation leads to *E*- to *Z*- isomerization.

**Figure 3:**
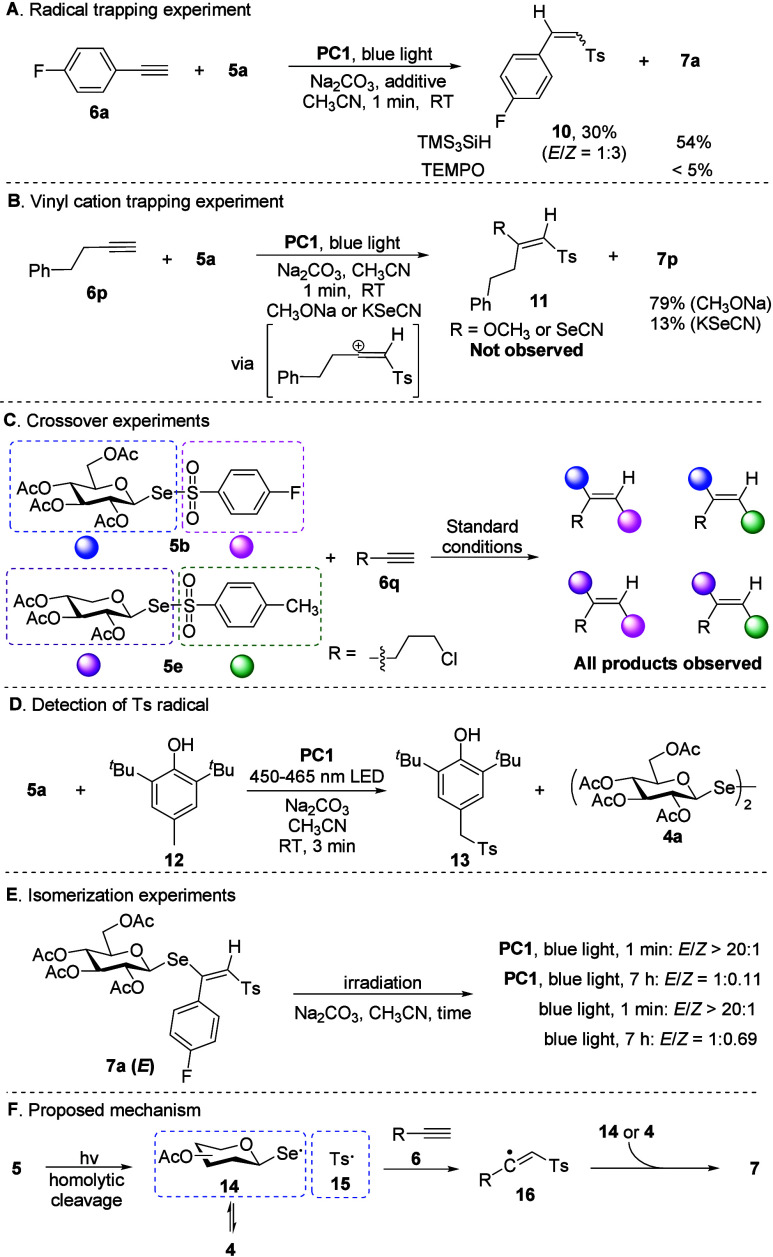
Mechanistic studies.

In conclusion, we have developed a novel class of glycosylseleno­sulfonates as highly versatile reagents for efficient glycosylselenylation. These innovative reagents uniquely integrate radical and electrophilic properties, enabling rapid transformations under mild conditions. Of special note, the glycosylseleno­sulfonates facilitate an ultrafast radical addition to alkynes, yielding structurally diverse glycosylseleno-based vinyl sulfones with excellent regio- and stereocontrol. Furthermore, these reagents demonstrate exceptional efficiency in the selective labeling of thiols under mild conditions, underscoring their pronounced electrophilicity. Additive experiments confirmed the robustness and broad functional group tolerance of these reactions, including compatibility with various biomolecular substrates, such as peptides. Compared with existing methods, this approach provides distinct advantages in kinetics, reagent efficiency, and substrate scope, showcasing its potential for bioorthogonal applications and the functionalization of complex biomolecules. This work not only expands the toolbox for glycosylselenylation but also opens new avenues for the development of selenium-based reagents in chemical biology.

## Supplementary Material





## Notes

Experimental procedures, characterization of new compounds, X-ray data for **5a**, and spectroscopic data (PDF)
CIF file for compound (CCDC 2362967) (ZIP)
