# Supplementary material for: Photoactivatable Electrophilic Glycosylselenosulfonates for Ultrafast Modification of Alkynes and Thiols
Source: ACS Cent Sci. 2025 Jul 7;11(8):1400–7. doi: 10.1021/acscentsci.5c00650 (PMC12395299; doi:10.1021/acscentsci.5c00650)
Supplement: Supplementary file 1 [file oc5c00650_si_001.pdf]

Supporting Information for Publication

Photoactivatable Electrophilic Glycosylselenosulfonates for UltraFast Modification of Alkynes and Thiols

Weitao Yan,[a,†] Wenchao Liu,[a,†] Qinshuo Zhang,[a] Wentao Lin,[a] Yujie Liao,[a] Yiqun Geng,[c] Ruo Wang,[d,]* Chunfa Xu[a,b]*

[a]Key Laboratory of Molecular Synthesis and Functionalization Discovery, College of Chemistry, Fuzhou University, Fuzhou, 350108, China. E-mail: xucf@fzu.edu.cn.

[b]Key Laboratory of Organofluorine Chemistry, Shanghai Institute of Organic Chemistry, Chinese Academy of Sciences, Shanghai 200032, China.

[c]State Key of Laboratory of Bioactive Substance and Function of Natural Medicine, Institute of Materia Media, Chinese Academy of Medical Sciences and Peking Union Medical College, Beijing China.

[d]Shengli Clinical Medical College of Fujian Medical University, Department of Breast Surgery, Fujian Provincial Hospital, Fuzhou University Affiliated Provincial Hospital, Fuzhou 350001, China.

Table of content

1. General information......3
2. Synthesis of glycosylselenosulfonate **5**......4
    2.1 General procedure for optimization of glycosylselenosulfonate synthesis......4
    2.2 General procedure for synthesis of glycosylselenosulfonate **5** (Procedure A)......5
    2.3 Synthesis of glycosylselenosulfonate in the presence of TEMPO......5
    2.4 ^{77}Se NMR and XRD analysis for glycosylselenosulfonate synthesis......5
3. Synthesis of glycosylseleno-based vinyl sulfone **7**......7
    3.1 General procedure for optimization......7
    3.2 General procedure for synthesis of product **7** (Procedure B)......12
    3.3 General procedure for additive experiment......12
4. Synthesis of product **9**......12
    4.1 General procedure for optimization......12
    4.2 General procedure for synthesis of product **9** (Procedure C)......13
    4.3 General procedure for additive experiments......13
5. Mechanistic studies for photocatalytic radical addition reaction......14
    5.1 Radical trapping experiments......14
    5.2 **4a** as starting material for addition reaction......16
    5.3 Proposed mechanism via SET process......17
    5.4 Attempt to trap vinyl cation......17
    5.5 Procedure for isomerization experiments......18
    5.6 Crossover reaction......19
    5.7 Regioselectivity discussion......21
    5.8 Reaction kinetics......21
    5.9 Reaction with lysine bearing an exposed ε-NH_2 group......22
6. Reactivity comparison experiments......23
    6.1 Photocatalytic radical addition reaction......23
    6.2 Glycosylselenylation of cysteine-containing molecules......23
7. Oligopeptide modification......24
8. The cytotoxicity of the substrates to 293T cells......25
9. Experimental characterization data for substrates and products......26
10. X-Ray for compound **5a**......50
11. References......52
12. NMR Spectra of compounds......53

1. General information

Unless otherwise stated, all reactions were set up under inert atmosphere (N_2) utilizing glassware that were oven dried and cooled under nitrogen purging. Silica Gel Flash Column Chromatography was performed on deactivated silica gel (particle size 300-400 mesh). Starting materials were purchased directly from commercial suppliers (Sigma Aldrich, Energy Chemical, Bidepharm, Tansoole) and used without further purifications unless otherwise stated. All solvents were dried according to standard procedures or brought from commercial suppliers. Reactions were monitored using thin-layer chromatography (TLC) with F254 indicator. Visualization of the developed plates was performed under UV light (254 nm) or H_2SO_4-EtOH (10% H_2SO_4 v/v). PBS (Na_2HPO_4-KH_2PO_4) pH value: 7.2-7.4.

^{1}H NMR, ^{19}F NMR, ^{13}C NMR and 2D-NMR spectra were recorded using Bruker AVIII 400 and JEOL JNM-ECA600 spectrometer. ^{1}H NMR and ^{13}C NMR chemical shifts were reported in parts per million (ppm) downfield from tetramethylsilane and ^{19}F NMR chemical shifts were determined relative to $CFCl_3$ as the external standard and low field is positive. Because of the complicated structure of sugar, the ^{13}C NMR chemical shifts retain two decimal places. Coupling constants (*J*) are reported in Hertz (Hz). The residual solvent peak was used as an internal reference: ^{1}H NMR ($CDCl_3$ δ 7.26 ppm), ^{13}C NMR ($CDCl_3$ δ 77.16 ppm), ^{1}H NMR (DMSO-d_6 δ 2.50 ppm), ^{13}C NMR (DMSO-d_6 δ 39.50 ppm), ^{1}H NMR (CD_3OD δ 4.87 ppm), ^{13}C NMR (CD_3OD δ 49.00 ppm). The following abbreviations were used to explain the multiplicities: s = singlet, d = doublet, t = triplet, q = quartet, m = multiplet, br = broad. IR spectra were recorded using Nicolet iS50 spectrometer. HRMS data was recorded using HRMR Exactive Plus instrument. *In situ* IR was recorded using ReactIR 15. The wavelength range of blue light is 450-465 nm.

Handling precautions: Standard laboratory safety protocols were strictly adhered to when handling these selenium reagents. This includes the use of personal protective equipment and conducting manipulations within a certified chemical fume hood to minimize exposure. To mitigate potential photochemical exposure risks, all reaction setups were shielded with stainless steel enclosures throughout the experimental procedures.

Waste disposal: Following the reactions, purification of the desired compounds was performed *via* silica gel column chromatography with solid loading of the crude material. This procedure effectively adsorbs residual selenium species and photocatalyst onto the silica gel matrix. The resulting selenium- and photocatalyst-contaminated silica gel was subsequently collected and disposed of as hazardous chemical solid waste, in accordance with institutional and local environmental safety regulations.

2. Synthesis of glycosylselenosulfonate 5

2.1 General procedure for optimization of glycosylselenosulfonate synthesis

To an oven-dried 5 mL tube equipped with a stirring bar were added **4a**[1], **S1b**, promoter and solvent (1 mL). The tube was sealed with a Teflon screw cap and the mixture was stirred at room temperature. Upon completion, the yield was determined by ^{19}F NMR using trifluoromethoxybenzene as an internal standard.

Table S1. Effect of solvent

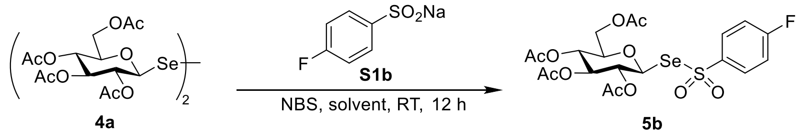

Entry	Solvent	Yield (%)[a]
1	CH_3CN	25
2	CH_2Cl_2	5
3	THF	25
4	DMF	NP
5	toluene	NP
6	EtOH	NP

[a]Reaction condition: **4a** (0.05 mmol, 1.0 equiv.), **S1b** (0.25 mmol, 5.0 equiv.), NBS (0.10 mmol, 2.0 equiv.), solvent (1 mL) at rt for 12 h, yields were determined by ^{19}F NMR using trifluoromethoxybenzene as an internal standard. NP = no product. NBS = *N*-Bromosuccinimide.

Table S2. Effect of oxidant

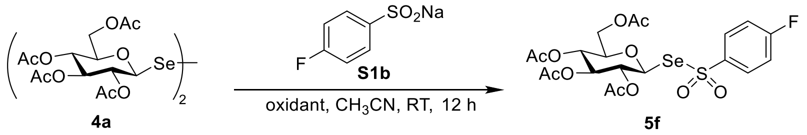

Entry	Oxidant	Yield (%)[a]
1	NBS	25
2	$SOCl_2$	59
3	I_2	10
4	NCS	34
5	NIS	45
6	TMSCl	trace

[a]Reaction condition: **4a** (0.05 mmol, 1.0 equiv.), **S1b** (0.25 mmol, 5.0 equiv.), oxidant (0.10 mmol, 2.0 equiv.), CH_3CN (1 mL) at rt for 12 h, yields were determined by ^{19}F NMR using trifluoromethoxybenzene as an internal standard. NBS = *N*-Bromosuccinimide. NCS = *N*-Chlorosuccinimide. NIS = *N*-Iodosuccinimide.

2.2 General procedure for synthesis of glycosylselenosulfonate 5 (Procedure A)

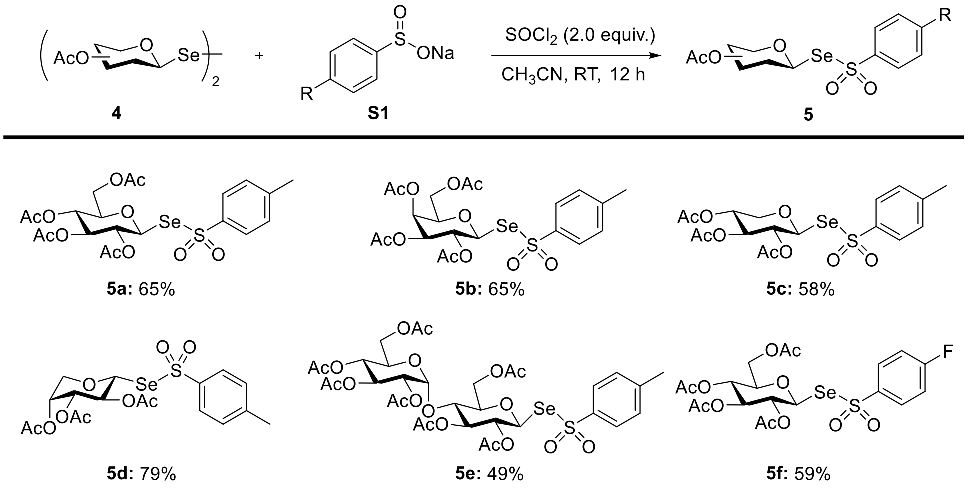

A flask was charged with **4** (0.32 mmol, 1.0 equiv.), **S1** (1.6 mmol, 5.0 equiv.), $SOCl_2$ (0.64 mmol, 2.0 equiv.) and CH_3CN (6.0 mL). The reaction mixture was stirred at room temperature in dark for 12 h. Upon completion, the reaction mixture was filtered with $NaHCO_3$ and then concentration. The resulting residue was purified by column chromatography on silica gel with dichloromethane as eluent to give the targeted product.

2.3 Synthesis of glycosylselenosulfonate in the presence of TEMPO

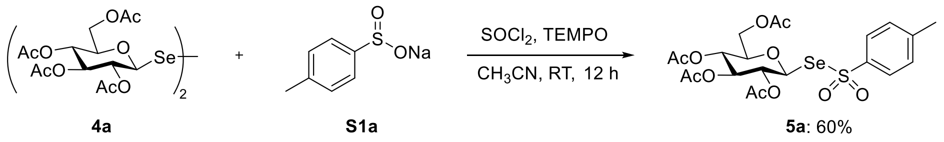

A flask was charged with **4a** (0.05 mmol, 1.0 equiv.), **S1a** (0.25 mmol, 5.0 equiv.), TEMPO (0.05 mmol, 1.0 equiv.) $SOCl_2$ (0.10 mmol, 2.0 equiv.) and CH_3CN (1.0 mL). The reaction mixture was stirred at room temperature in dark for 12 h. Upon completion, the reaction mixture was filtered with $NaHCO_3$ and then concentration. Yield was determined by ^{1}H NMR using 1,3,5-trimethoxybenzene as an internal standard.

The yield in this condition with the addition of TEMPO is comparable to the standard conditions, implying that the radical species is possibly not involved in the transformation.

2.4 ^{77}Se NMR and XRD analysis for glycosylselenosulfonate synthesis

A flask was charged with **4a** (0.05 mmol, 1.0 equiv.), **S1a** (0.25 mmol, 5.0 equiv.), $SOCl_2$ (0.1 mmol, 2.0 equiv.) and CH_3CN (1.0 mL). The reaction mixture was stirred at room temperature in dark for 4 h, and then directly submitted for ^{77}Se NMR detection. The crude ^{77}Se NMR spectrum was listed below (bottom). Only the signals for starting material diselenide **4a** and the product **5a** can be observed.

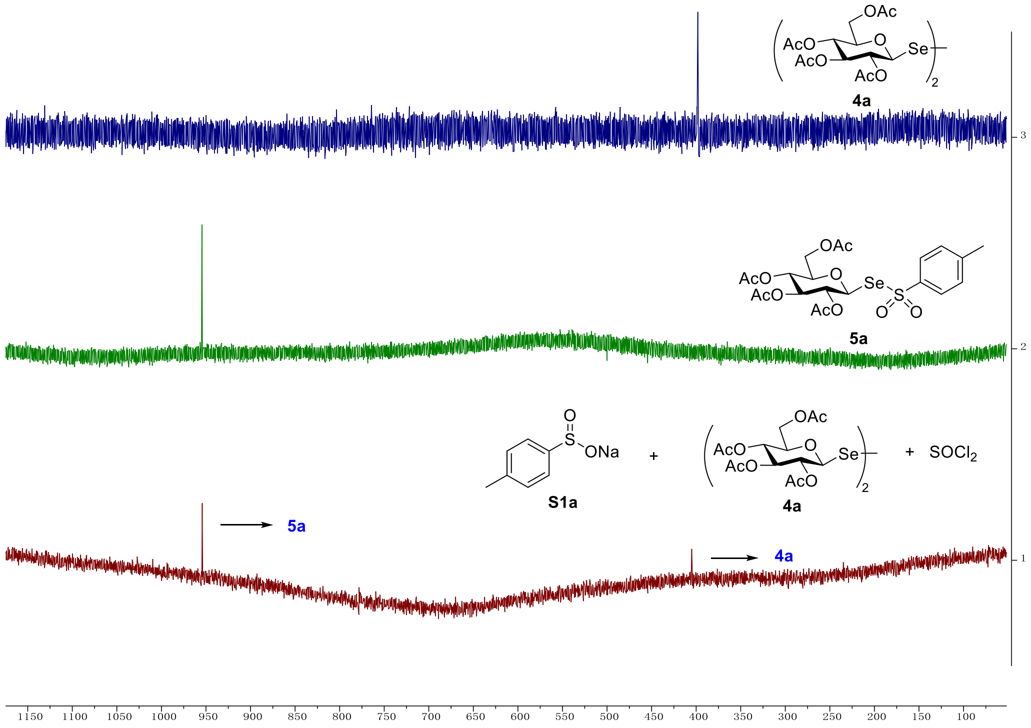

Figure S1. ^{77}Se NMR spectra for compounds

Then, in an effort to detect the possible intermediate for this transformation, a reaction without addition of **S1a** was conducted. However, no new signal was detected except for the starting material **4a**. Additionally, some reddish-brown precipitation was formed. These results suggested the intermediate is easy to decompose in the absence of the **S1a**. The reddish-brown precipitation was further analyzed by XRD. The signal was consistent with the Se_4S_4 (Figure S2). Additional elemental analysis also demonstrated the existence of Se and S (Figure S3-4).

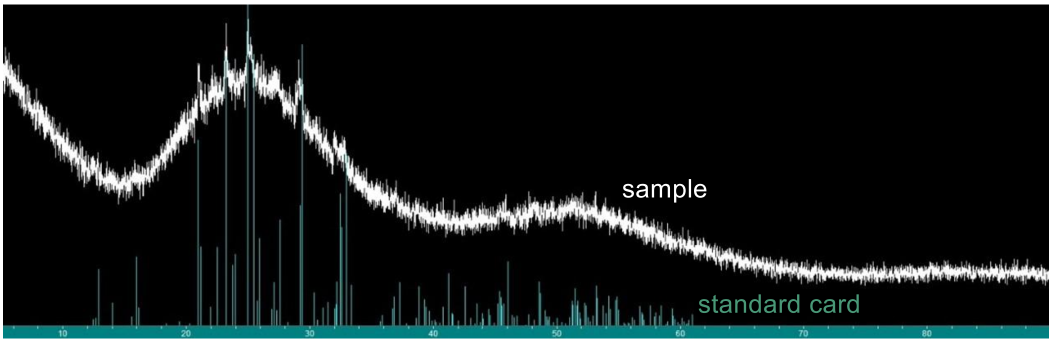

Figure S2. X-ray powder diffraction

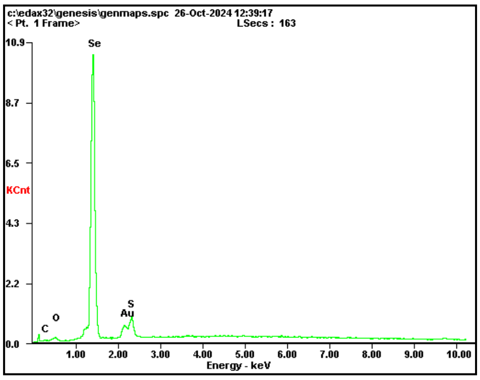

Figure S3. Elemental analysis

Element	Wt%	At%
CK	02.04	11.15
OK	01.86	07.64
SeL	78.59	65.30
AuM	11.63	03.87
SK	05.88	12.03
Matrix	Correction	ZAF

Figure S4. Elemental content

3. Synthesis of glycosylseleno-based vinyl sulfone 7

3.1 General procedure for optimization

In a glove box filled with nitrogen, to an oven-dried 5 mL tube equipped with a stirring bar were added **6a** (0.2 mmol, 2.0 equiv.), **5a** (0.1 mmol, 1.0 equiv.), photocatalyst (0.002 mmol, 2 mol%), base (0.1 mmol, 1.0 equiv.), solvent (1 mL). The tube was sealed with a Teflon screw cap and the mixture was stirred at room temperature. Upon completion, the yield was determined by ^{1}H NMR using 1,3,5-trimethoxybenzene as an internal standard.

Table S3. Effect of photocatalyst

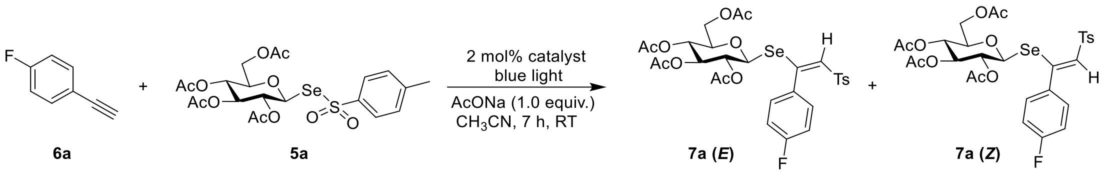

Entry	Catalyst	Yield (%)[a]	***E*:*Z***
1	**PC1**	91	10:1
2	**PC2**	89	7:1
3	**PC3**	98	1:1
4	**PC4**	92	5:1
5	**PC5**	88	2:1
6	**PC6**	85	3:1
7	no catalyst	89	1:1

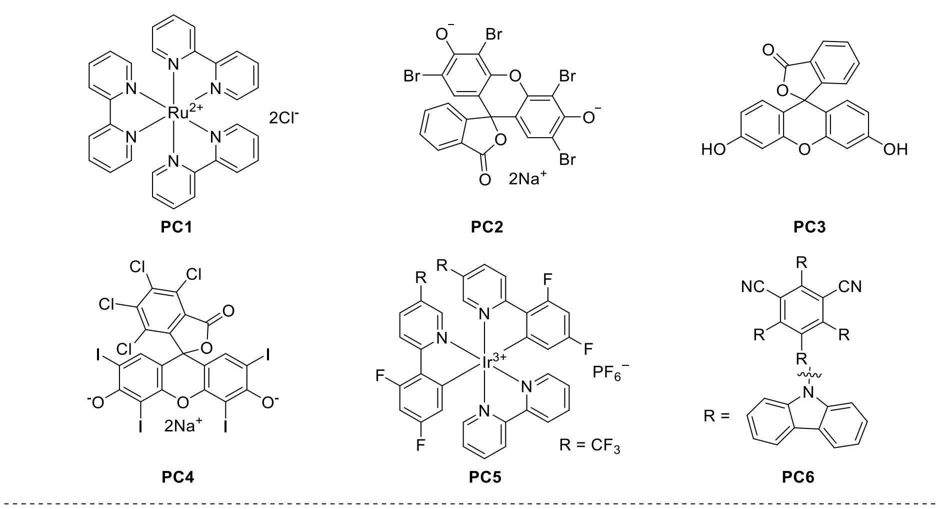

[a]Reaction conditions: **6a** (0.2 mmol, 2.0 equiv.), **5a** (0.1 mmol, 1.0 equiv.), catalyst (0.002 mmol, 2 mol%), Na_2CO_3 (0.1 mmol, 1.0 equiv.), CH_3CN (1 mL), blue light (450-465 nm), 7 h, yields were determined by ^{1}H NMR using 1,3,5-trimethoxybenzene as an internal standard.

Table S4. Effect of base

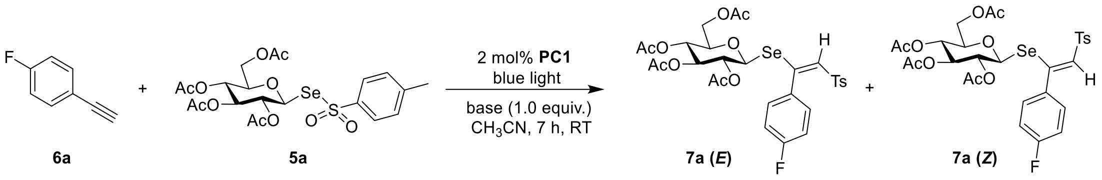

Entry	Base	Yield (%)[a]	*E*:*Z*
1	none	80	8:1
2	CH_3CO_2Na	91	10:1
3	Na_2CO_3	91	14:1
4	Cs_2CO_3	70	1:1
5	KOH	86	8:1

[a]Reaction conditions: **6a** (0.2 mmol, 2.0 equiv.), **5a** (0.1 mmol, 1.0 equiv.), **PC1** (0.002 mmol, 2 mol%), base (0.1 mmol, 1.0 equiv.), CH_3CN (1 mL), blue light (450-465 nm), 7 h, yields were determined by ^1H NMR using 1,3,5-trimethoxybenzene as an internal standard.

Table S5. Effect of solvent

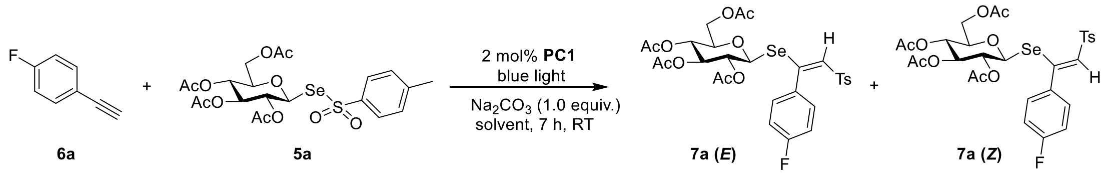

Entry	Solvent	Yield (%)[a]	*E*:*Z*
1	CH_3CN	91	14:1
2	DMSO	77	13:1
3	DMF	62	>20:1
4	CH_3OH	NP	ND
5	CH_2Cl_2	84	11:1
6	toluene	91	5:1

[a]Reaction conditions: **6a** (0.2 mmol, 2.0 equiv.), **5a** (0.1 mmol, 1.0 equiv.), **PC1** (0.002 mmol, 2 mol%), Na_2CO_3 (0.1 mmol, 1.0 equiv.), solvent (1 mL), blue light (450-465 nm), 7 h, yields were determined by ^1H NMR using 1,3,5-trimethoxybenzene as an internal standard.

Table S6. Effect of equivalent

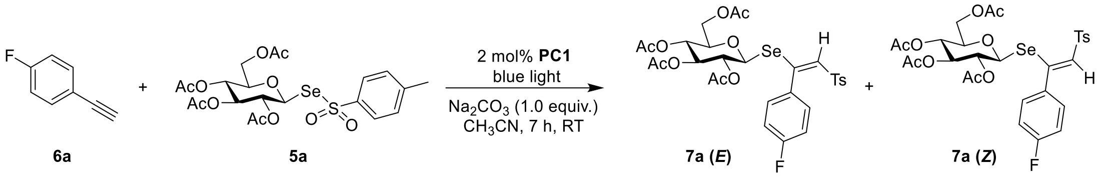

Entry	6a:5a	Yield (%)[a]	*E*:*Z*
1	2:1	91	14:1
2	1:1	88	13:1
3	1.2:1	96	10:1
4	1:1.2	89	13:1

[a]Reaction conditions: **6a** (0.2 mmol, 2.0 equiv.), **5a** (0.1 mmol, 1.0 equiv.), **PC1** (0.002 mmol, 2 mol%), Na_2CO_3 (0.1 mmol, 1.0 equiv.), CH_3CN (1 mL), blue light (450-465 nm), 7 h, yields were determined by ^1H NMR using 1,3,5-trimethoxybenzene as an internal standard.

Table S7. Effect of time

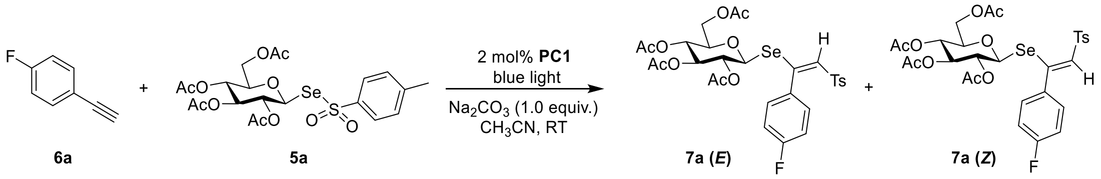

Entry	Time	Yield (%)[a]	*E*:*Z*
1	1 min	88 (94)[b]	>20:1
2	10 min	88	>20:1
3	7 h	96	10:1

[a]Reaction conditions: **6a** (0.12 mmol, 1.2 equiv.), **5a** (0.1 mmol, 1.0 equiv.), **PC1** (0.002 mmol, 2 mol%), Na_2CO_3 (0.1 mmol, 1.0 equiv.), CH_3CN (1 mL), blue light (450-465 nm), yields were determined by ^1H NMR using 1,3,5-trimethoxybenzene as an internal standard; [b]Isolated yield.

Table S8. Reaction investigation with various photocatalyst in 1 min

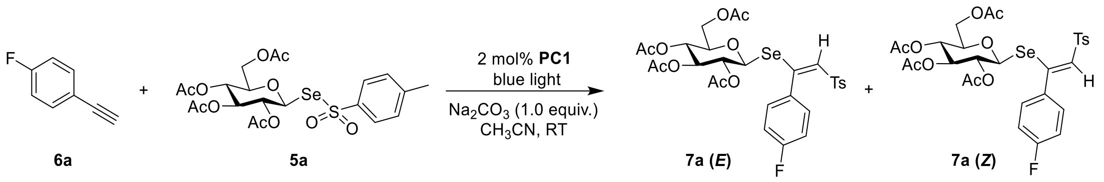

Entry	Catalyst	Yield (%)[a]	*E*:*Z*
1	**PC1**[b]	92	>20:1
2	**PC2**	70	>20:1
3	**PC3**	41	>20:1
4	**PC4**	61	>20:1

[a]Reaction condition: **6a** (0.12 mmol, 1.2 equiv.), **5a** (0.1 mmol, 1.0 equiv.), **PC1** (0.002 mmol, 2 mol%), Na_2CO_3 (0.1 mmol, 1.0 equiv.), CH_3CN (1 mL), blue light (450-465 nm), yields were determined by ^1H NMR using 1,3,5-trimethoxybenzene as an internal standard; [b]White light.

Table S9. Control experiments

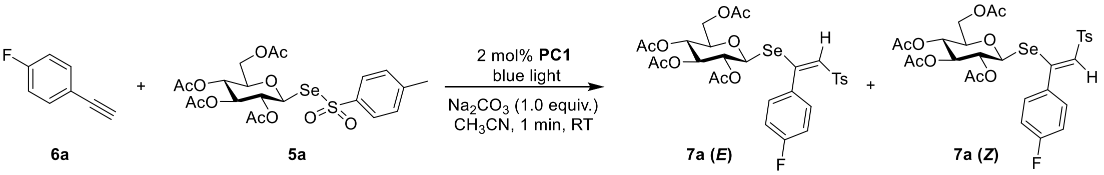

Entry	Deviation from standard condition	Yield (%)[a]	*E*:*Z*
1	none	88	＞20:1
2	no catalyst	49	＞20:1
3	no base	13	＞20:1
4	no light	N.P.	N.D.
5	in air	N.P.	N.D.

[a]Reaction conditions: **6a** (0.12 mmol, 1.2 equiv.), **5a** (0.1 mmol, 1.0 equiv.), **PC1** (0.002 mmol, 2 mol%), Na_2CO_3 (0.1 mmol, 1.0 equiv.), CH_3CN (1 mL), blue light (450-465 nm), 1 min, yields were determined by ^1H NMR using 1,3,5-trimethoxybenzene as an internal standard.

Table S10. Reaction performed in CH_3CN/PBS

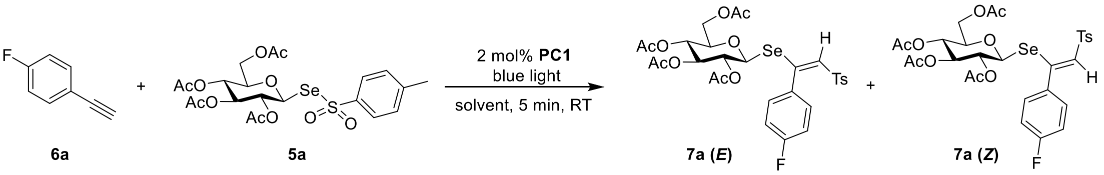

Entry	solvent	Yield (%)[a]	*E*:*Z*
1	CH_3CN/PBS (pH 9.0) = 1 : 4	92	＞20:1

aReaction conditions: **6a** (0.12 mmol, 1.2 equiv.), **5a** (0.1 mmol, 1.0 equiv.), **PC1** (0.002 mmol, 2 mol%), solvent (1 mL), blue light (450-465 nm), 5 min, yields were determined by ^{1}H NMR using 1,3,5-trimethoxybenzene as an internal standard.

3.2 General procedure for synthesis of product 7 (Procedure B)

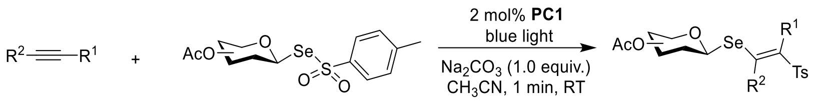

6 **5** **7**

In a glove box filled with nitrogen, to an oven-dried 10 mL tube equipped with a stirring bar were added **6** (0.12 mmol, 1.2 equiv.), **5** (0.1 mmol, 1.0 equiv.), **PC1** (0.002 mmol, 2 mol%), Na_2CO_3 (0.1 mmol, 1.0 equiv.), and CH_3CN (1 mL). The reaction mixture was stirred under irradiation with a blue LED (450-465 nm) at room temperature for 1 min. Upon completion, the resulting residue was purified by column chromatography on silica gel with petroleum ether/ethyl acetate as eluent to give the targeted product.

3.3 General procedure for additive experiment

In a glove box filled with nitrogen, to an oven-dried 5 mL tube equipped with a stirring bar were added **6a** (0.06 mmol, 1.2 equiv.), **5a** (0.05 mmol, 1.0 equiv.), photocatalyst (0.001 mmol, 2 mol%), Na_2CO_3 (0.05 mmol, 1.0 equiv.), additive (0.05 mmol, 1.0 equiv.), CH_3CN (0.5 mL). The tube was sealed with a Teflon screw cap and the mixture was stirred at room temperature for 1 min under blue LED (450-465 nm) irradiation. Upon completion, the yield was determined by ^{1}H NMR using 1,3,5-trimethoxybenzene as an internal standard.

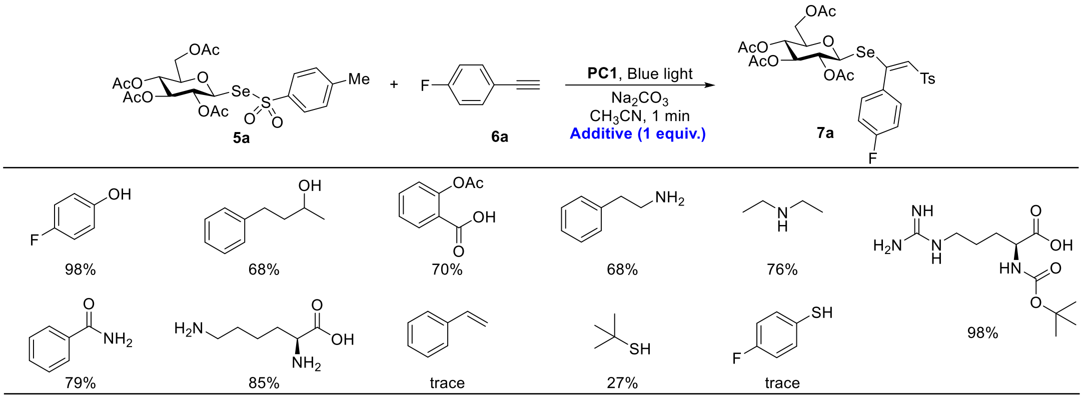

aReaction conditions: **6a** (0.06 mmol, 1.2 equiv.), **5a** (0.05 mmol, 1.0 equiv.), **PC1** (0.001 mmol, 2 mol%), Na_2CO_3 (0.05 mmol, 1.0 equiv.), additive (0.05 mmol, 1.0 equiv.), CH_3CN (0.5 mL), blue light (450-465 nm), 1 min, yields were determined by ^{1}H NMR using 1,3,5-trimethoxybenzene as an internal standard.

Scheme S1. Additive investigation for photocatalytic addition reactions

4. Synthesis of product 9

4.1 General procedure for optimization

To a 10 mL tube equipped with a stirring bar were added **5a** (0.05 mmol, 1.0 equiv.), **8a** (0.05 mmol, 1.0 equiv.) and solvent (1 mL). The tube was sealed with a Teflon screw cap and the mixture was stirred

at room temperature. Upon completion, the yield was determined by ^{1}H NMR using 1,3,5-trimethoxybenzene as an internal standard.

Table S11. Reaction Optimization

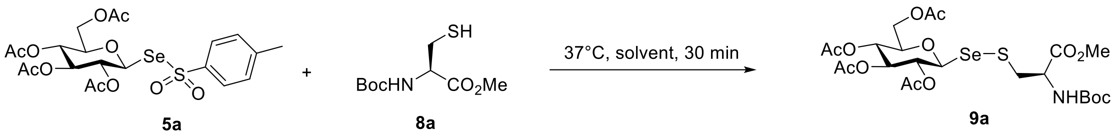

Entry	Solvent	Yield (%)[a]
1	DMSO	99 (95)[b]
2	DMF	79 (75)[b]
3	DMSO : PBS (pH 9.0) = 1 : 1	83
4	DMSO : PBS (pH 7.2) = 1 : 1	95
5	DMSO, 1 min	99
6	DMSO, 1 min, in dark	99

[a]Reaction condition: **5a** (0.05 mmol), **8a** (0.05 mmol), solvent (1 mL), 30 min, yields were determined by ^{1}H NMR using 1,3,5-trimethoxybenzene as an internal standard; [b]Isolated yield.

4.2 General procedure for synthesis of product 9 (Procedure C)

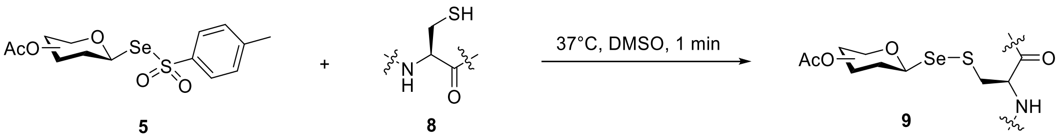

To a 10 mL tube equipped with a stirring bar were added **5** (0.10 mmol, 1.0 equiv.), **8** (0.10 mmol, 1.0 equiv.) and DMSO (2 mL). The tube was sealed with a Teflon screw cap and the solution was stirred at room temperature for 1 min. Upon completion, the crude mixture was extracted with ethyl acetate/H_2O. The organic phase was separated and dried over $MgSO_4$. After filtration, the solvent was removed under reduced pressure using a rotary evaporator. The resulting residue was purified by column chromatography on silica gel with petroleum ether/ethyl acetate as eluent to give the targeted product.

4.3 General procedure for additive experiments

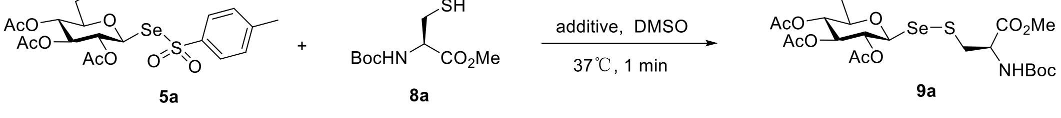

To a 10 mL tube equipped with a stirring bar were added **5a** (0.05 mmol, 1.0 equiv.), **8a** (0.05 mmol, 1.0 equiv.), additive (0.05 mmol, 1.0 equiv.) and DMSO (1 mL). The tube was sealed with a Teflon screw cap and the solution was stirred at room temperature for 1 min. Upon completion, the yield was determined by ^{1}H NMR using 1,3,5-trimethoxybenzene as an internal standard.

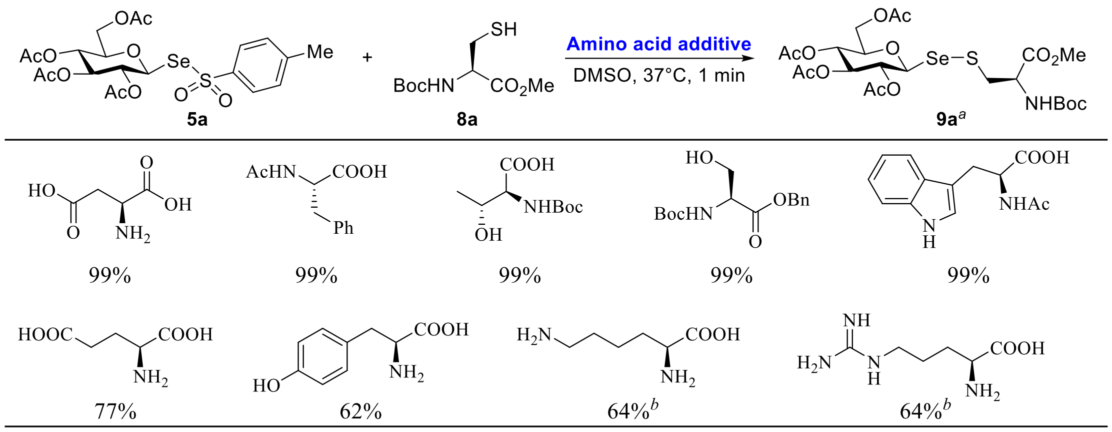

[a]Reaction condition: **5a** (0.05 mmol, 1.0 equiv.), **8a** (0.05 mmol, 1.0 equiv.), additive (0.05 mmol, 1.0 equiv.), solvent (1 mL), 1 min, yields were determined by ^{1}H NMR using 1,3,5-trimethoxybenzene as an internal standard; [b]DMSO/PBS (pH 7.2) = 1:1.

Scheme S2. Additive investigation for thiol functionalization

5. Mechanistic studies for photocatalytic radical addition reaction

5.1 Radical trapping experiments

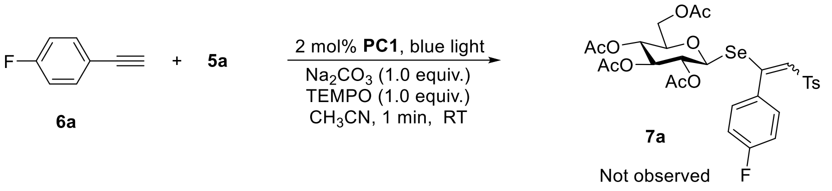

In a glove box filled with nitrogen, to an oven-dried 10 mL tube equipped with a stirring bar were added **6a** (0.12 mmol, 1.2 equiv.), **5a** (0.10 mmol, 1.0 equiv.), **PC1** (0.002 mmol, 2 mol%), Na_2CO_3 (0.10 mmol, 1.0 equiv.), TEMPO (0.10 mmol, 1.0 equiv.), CH_3CN (1 mL). The reaction mixture was stirred under irradiation of a commercially available bule LED (450-465 nm) at room temperature for 1 min. Upon completion, the crude mixture was concentrated and the mixture was determined by HRMS and ^{1}H NMR analysis.

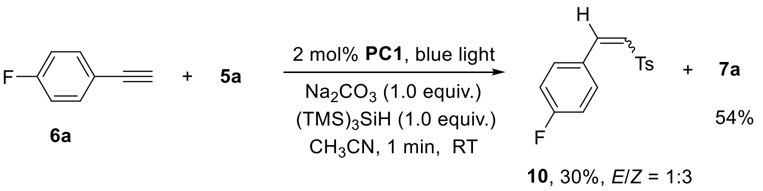

In a glove box filled with nitrogen, to an oven-dried 10 mL tube equipped with a stirring bar were added **6a** (0.12 mmol, 1.2 equiv.), **5a** (0.10 mmol, 1.0 equiv.), **PC1** (0.002 mmol, 2 mol%), Na_2CO_3 (0.10 mmol, 1.0 equiv.), $(TMS)_3SiH$ (0.10 mmol, 1.0 equiv.), CH_3CN (1 mL). The reaction mixture was stirred under irradiation of a commercially available bule LED (450-465 nm) at room temperature for 1 min. Upon completion, the crude mixture was concentrated and the yield was determined by ^{1}H NMR using

1,3,5-trimethoxybenzene as an internal standard. The isomer ratio of **10** was determined according to *J* value of the peaks in ^{1}H NMR spectra and the literature[2,3]

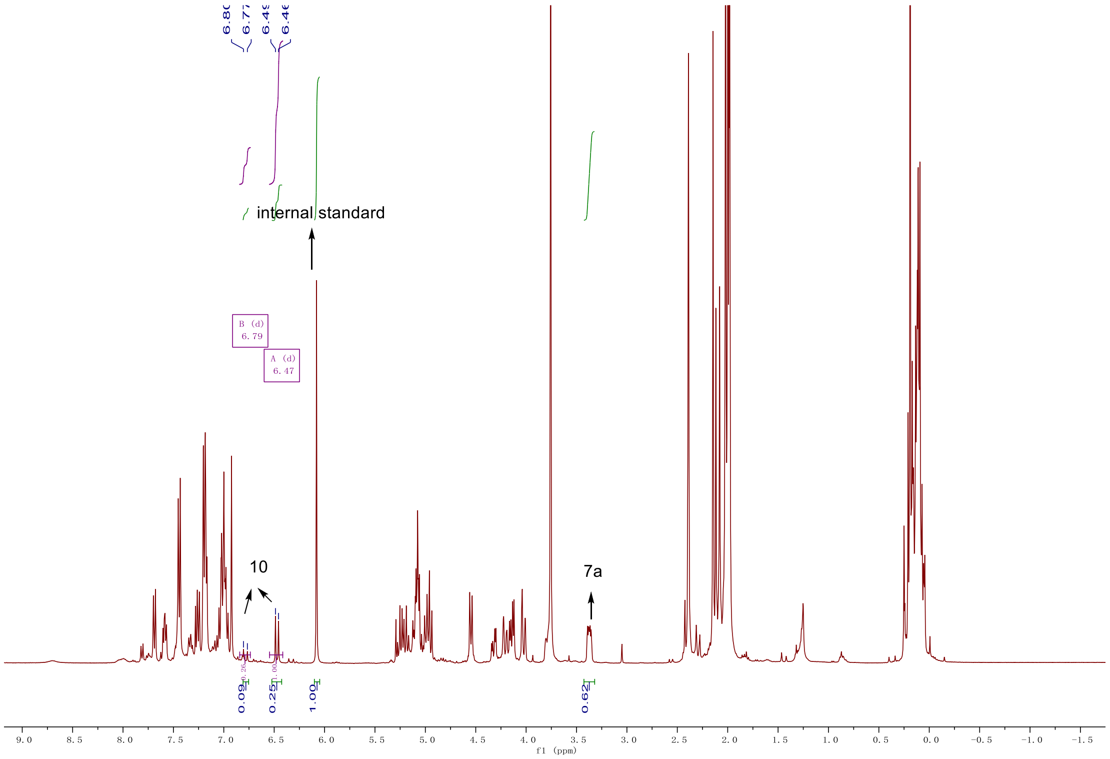

Figure S5. Crude ^{1}H NMR spectrum for radical trapping experiment with TMS_3SiH

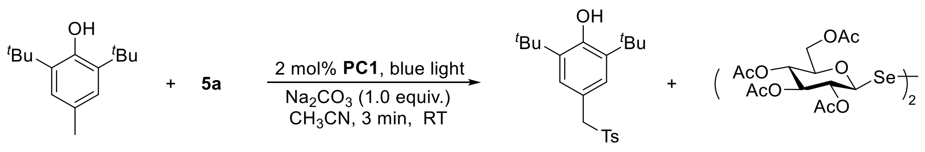

12 **13** **4a**

In a glove box filled with nitrogen, to an oven-dried 10 mL tube equipped with a stirring bar were added **5a** (0.10 mmol, 1.0 equiv.), **PC1** (0.002 mmol, 2 mol%), Na_2CO_3 (0.10 mmol, 1.0 equiv.), butylated hydroxytoluene **12** (BHT) (0.10 mmol, 1.0 equiv.), CH_3CN (1 mL). The reaction mixture was stirred under irradiation of a commercially available blue LED (450-465 nm) at room temperature for 3 min. Upon completion, the crude mixture was concentrated and the mixture was determined by HRMS analysis.

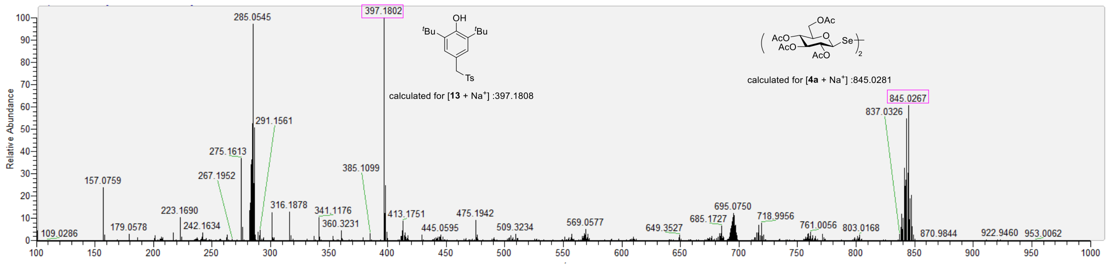

Figure S6. HRMS analysis for radical trapping experiment with BHT

5.2 4a as starting material for addition reaction

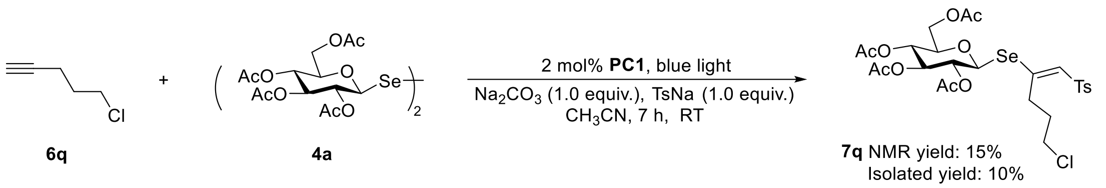

In a glove box filled with nitrogen, to an oven-dried 10 mL tube equipped with a stirring bar were added **5a** (0.10 mmol, 1.0 equiv.), **PC1** (0.002 mmol, 2 mol%), Na_2CO_3 (0.10 mmol, 1.0 equiv.), BHT (0.10 mmol, 1.0 equiv.), CH_3CN (1 mL). The reaction mixture was stirred under irradiation of a commercially available bule LED (450-465 nm) at room temperature for 7 h. Upon completion, the crude mixture was concentrated and the mixture was determined by ^1H NMR using 1,3,5-trimethoxybenzene as an internal standard and was isolated.

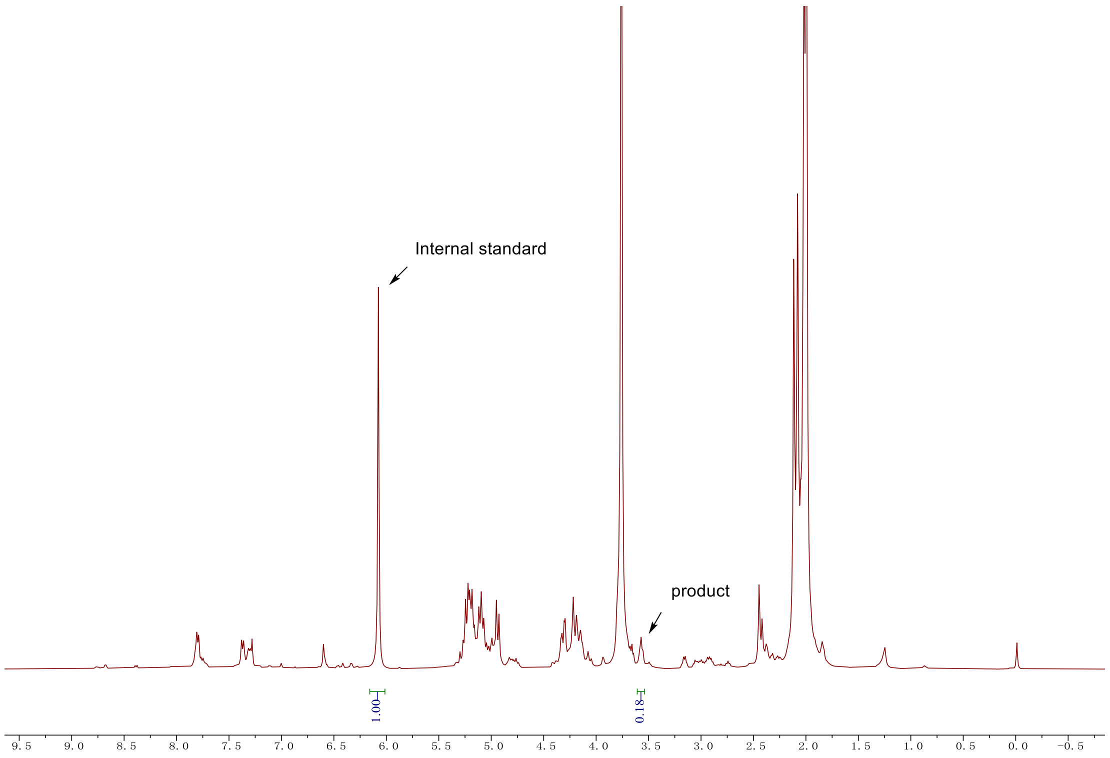

Figure S7. Crude ^1H NMR spectrum

5.3 Proposed mechanism via SET process

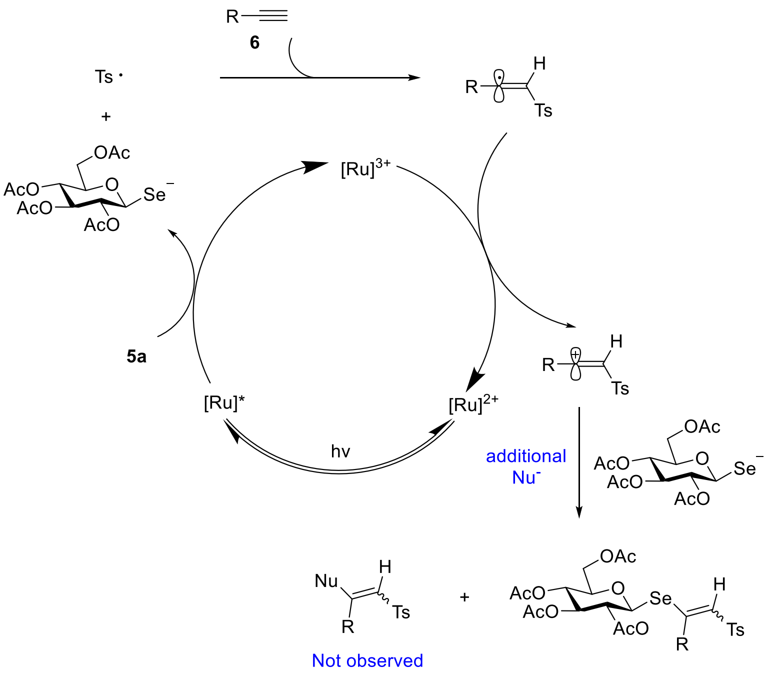

Figure S8. Single electron transfer process

5.4 Attempt to trap vinyl cation

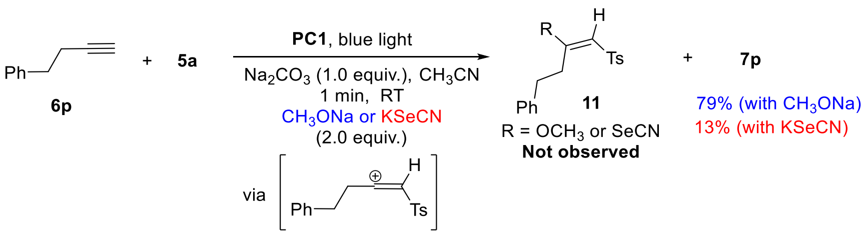

In a glove box filled with nitrogen, to an oven-dried 5 mL tube equipped with a stirring bar were added **6p** (0.06 mmol, 1.2 equiv.), **5a** (0.05 mmol, 1.0 equiv.), **PC1** (0.001 mmol, 2 mol%), Na_2CO_3 (0.05 mmol, 1.0 equiv.), CH_3ONa or KSeCN (0.10 mmol, 2.0 equiv.), CH_3CN (0.5 mL). The reaction mixture was stirred under irradiation of a commercially available bule LED at room temperature for 1 min. Upon completion, the crude mixture was concentrated and the residue was determined by HRMS and crude ^1H NMR spectra analysis.

As the potential product **11** via vinyl cation intermediate was not formed, therefore, the single electron transfer mechanism was possibly not involved.

5.5 Procedure for isomerization experiments

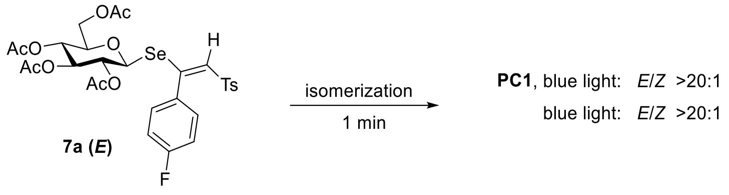

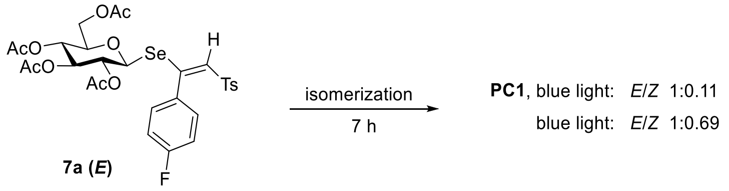

In a glove box filled with nitrogen, to an oven-dried 5 mL tube equipped with a stirring bar were added **7a (*E*)** (0.01 mmol, 1.0 equiv.), **PC1** (0.0002 mmol, 2 mol%), Na_2CO_3 (0.01 mmol, 1.0 equiv.), and CH_3CN (0.1 mL). The reaction mixture was stirred under irradiation with a blue LED at room temperature. Upon completion, the crude mixture was concentrated and the mixture was determined by ^{1}H NMR spectra analysis using 1,3,5-trimethoxybenzene as an internal standard.

Discussion on the isomerization: At the early stage (1 min), the photoisomerization process does occur but remains below the detection limit of NMR spectroscopy, resulting in no observable *Z*-isomer signal. Over extended reaction time (7 h), the extent of isomerization progressively increases, leading to sufficient accumulation of the *Z*-isomer for clear detection. This observation aligns with the gradual nature of the photocatalytic isomerization process, where prolonged irradiation enhances the conversion efficiency.

5.6 Crossover reaction

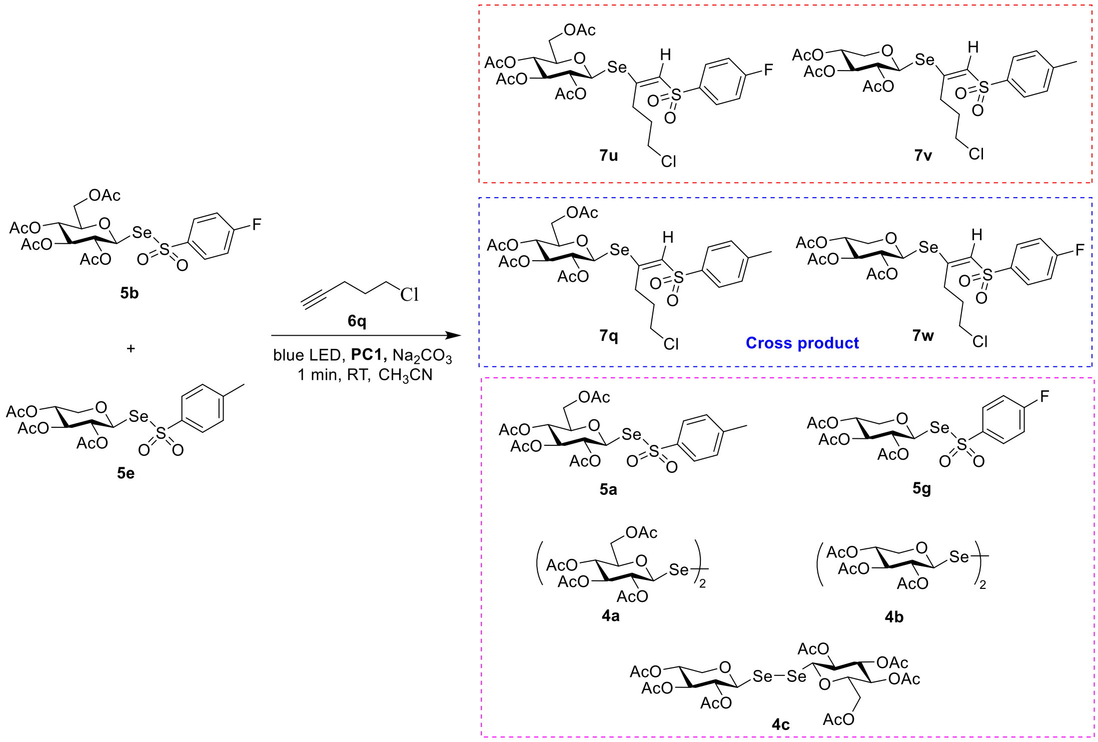

In a glove box filled with nitrogen, to an oven-dried 10 mL tube equipped with a stirring bar were added **6q** (0.12 mmol, 1.2 equiv.), **5b** (0.10 mmol, 1.0 equiv.), **5e** (0.10 mmol, 1.0 equiv.), **PC1** (0.002 mmol, 2 mol%), Na_2CO_3 (0.10 mmol, 1.0 equiv.) and CH_3CN (1 mL). The reaction mixture was stirred under irradiation with a blue LED at room temperature for 1 min. Upon completion, the crude mixture was concentrated and the residue was determined by HRMS analysis.

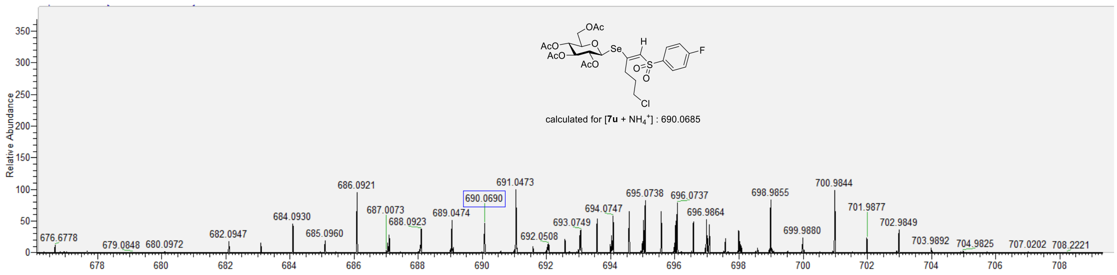

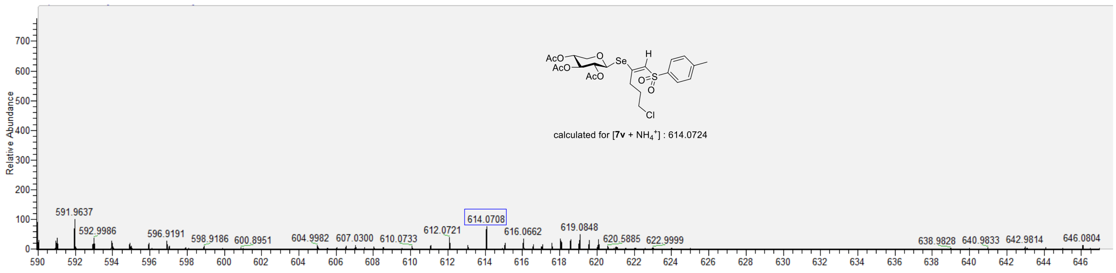

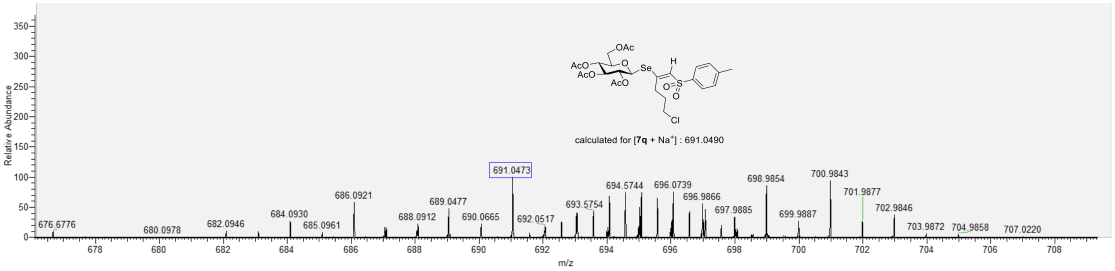
Relative Abundance
m/z
calculated for [7q + Na+] : 691.0490
691.0473

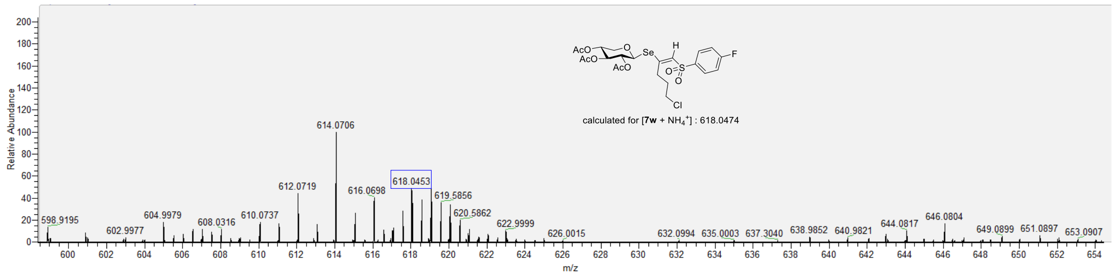
Relative Abundance
m/z
calculated for [7w + NH4+] : 618.0474
618.0453

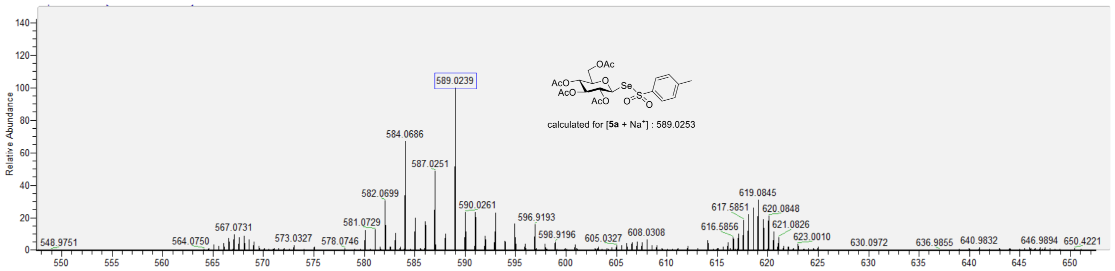
Relative Abundance
m/z
calculated for [5a + Na+] : 589.0253
589.0239

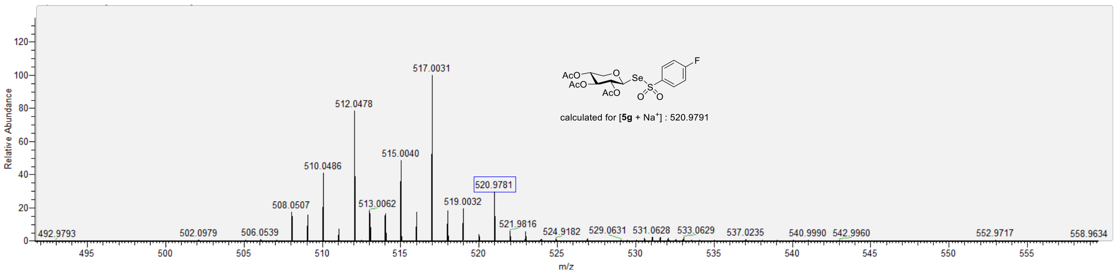
Relative Abundance
m/z
calculated for [5g + Na+] : 520.9791
520.9781

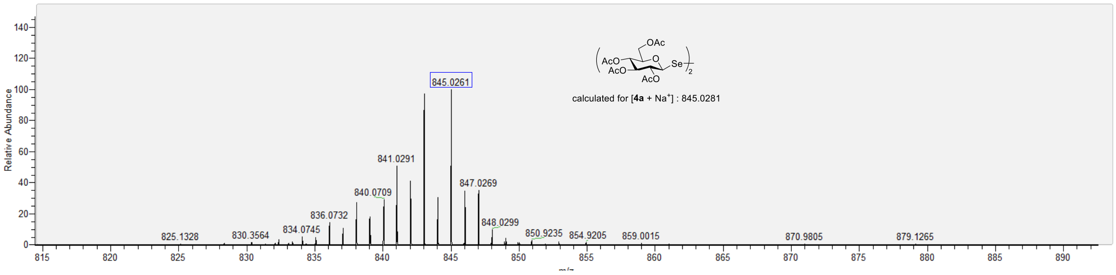
Relative Abundance
calculated for [4a + Na+] : 845.0281
845.0261

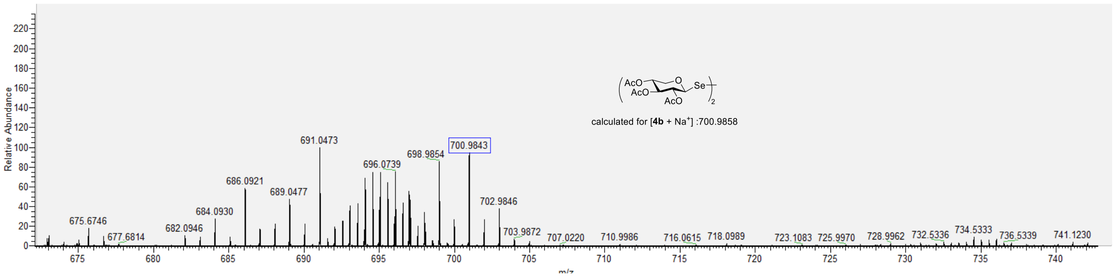
Relative Abundance
calculated for [4b + Na+] :700.9858
700.9843

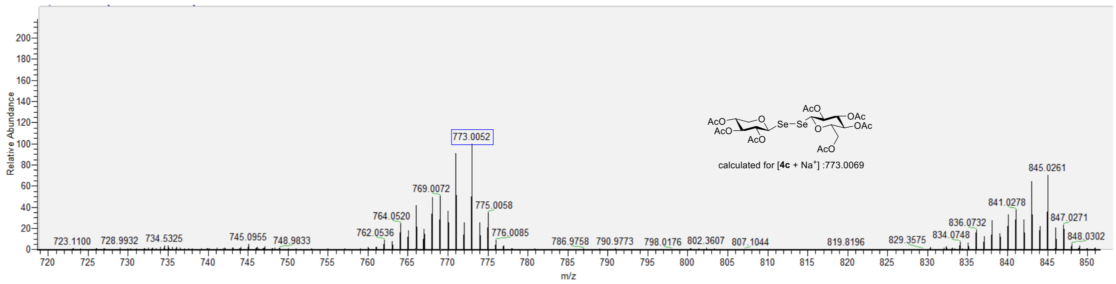

Figure S9. HRMS analysis for crossover reaction

5.7 Regioselectivity discussion

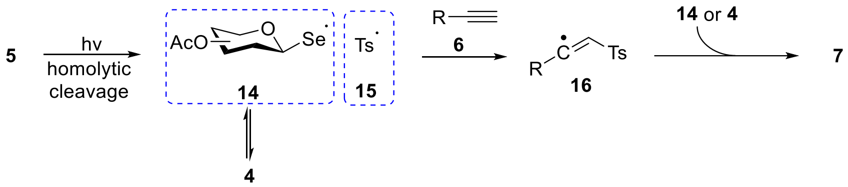

Figure S10. Proposed mechanism

Through high-resolution mass spectrometry (HRMS) analysis, we deduced that Ts radicals and glycosylselenyl radicals are readily generated in the reaction system. Due to the extremely fast self-coupling (dimerization) rate of the glycosylselenyl radical, it predominantly exists in a dormant diselenide form after its initial generation. The Ts radical, on the other hand, reacts with the alkyne present in the system to form a vinyl radical intermediate. The regioselectivity of this addition step is primarily governed by the relative stability of the possible vinyl radicals formed. Specifically, a vinyl radical with the radical center at the carbon adjacent to the aryl group (an α-aryl vinyl radical) is more stable than one where the radical center is at the terminal carbon (a β-aryl vinyl radical). Consequently, the Ts radical preferentially adds to the acetylenic carbon atom that is further away from the aryl substituent, leading to the formation of the more stabilized α-aryl vinyl radical.

5.8 Reaction kinetics

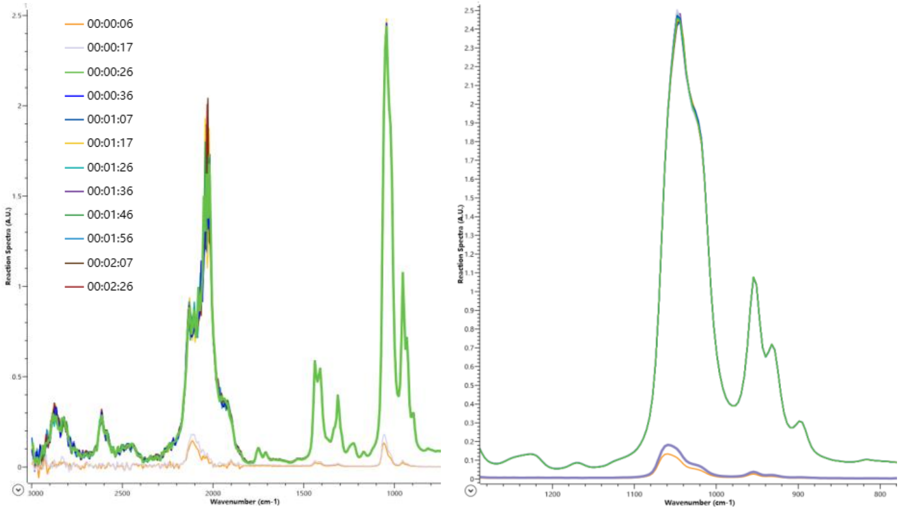

Figure S11. In situ IR for cysteine coupling reaction

A 10 mL reaction tube equipped with a stirring bar was charged with compound **5a** (0.05 mmol, 1.0 equiv.) and **8a** (0.05 mmol, 1.0 equiv.). The *in situ* IR probe was positioned in the reaction vessel prior to initiation. After establishing the instrument detection parameters, DMSO (1 mL) was rapidly introduced, and the reaction mixture was stirred at room temperature.

The reaction was monitored at 10-second intervals. Notably, the conversion was relatively low at the first and second monitoring points, yet reached near-quantitative conversion by the third time point. Subsequent measurements showed minimal further changes, allowing us to conclude that the reaction proceeded to completion within 30 seconds.

5.9 Reaction with lysine bearing an exposed ε-NH_2 group

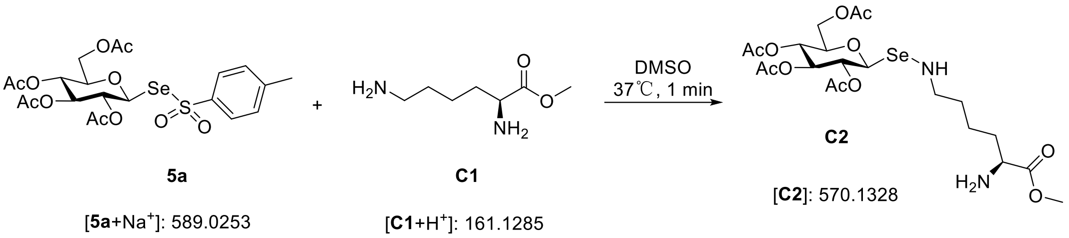

A flask was charged with methyl *L*-lysinate dihydrochloride (0.05 mmol, 1.0 equiv.), Et_3N (0.10 mmol, 2.0 equiv.) and DMSO-d_6 (1.0 mL). The reaction mixture was stirred at room temperature for 10 h. After that, **5a** (0.05 mmol, 1.0 equiv.) was added to the solution. The tube was sealed with a Teflon screw cap and the solution was stirred at 37°C for 1 min. The mixture was analyzed by HRMS analysis.

HRMS result: only signals for **C1** and **5a** were detected. The expected product **C2** was not observed.

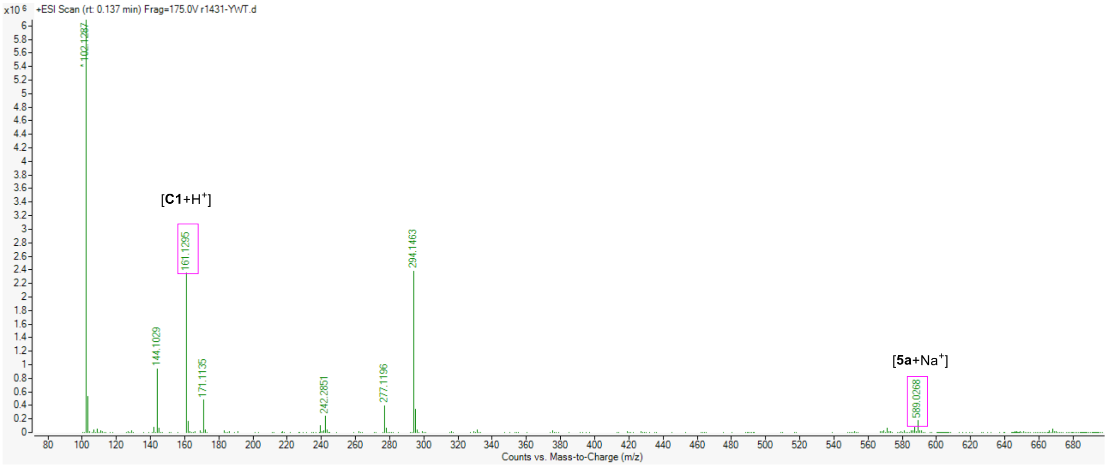

Figure S12. HRMS analysis for reaction with lysine

6. Reactivity comparison experiments

6.1 Photocatalytic radical addition reaction

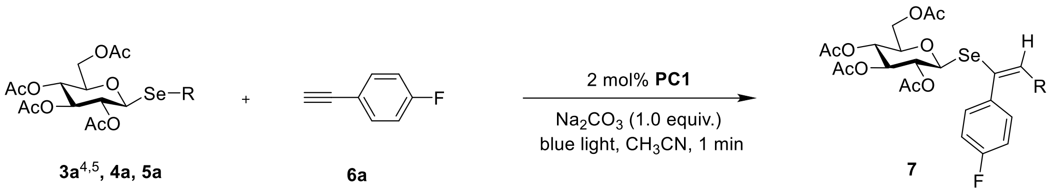

3a[4,5], 4a, 5a   6a   7

Entry	Substrate	Yield (%)[a]
1	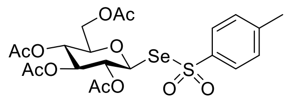 5a	91
2	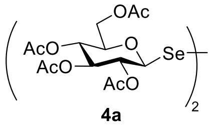 4a	NP
3	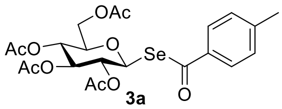 3a	NP

[a]Reaction condition: **6a** (0.06 mmol, 1.2 equiv.), Na_2CO_3 (0.05 mmol, 1.0 equiv.), **PC1** (2 mol%), CH_3CN (1 mL) at rt for 1 min under N_2 atmosphere, yields were determined by ^1H NMR using 1,3,5-trimethoxybenzene as an internal standard. NP = no product.

Scheme S3. Reactivity comparison in the photocatalytic radical addition reactions

6.2 Glycosylselenylation of cysteine-containing molecules

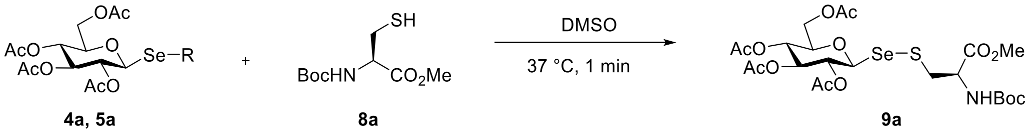

4a, 5a   8a   9a

Entry	Substrate	Yield (%)[a]
1	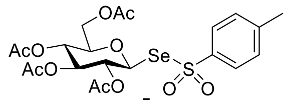 5a	99
2	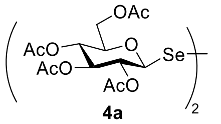 4a	NP

[a]Reaction condition: **8a** (0.05 mmol, 1.0 equiv.), DMSO (1 mL) at rt for 1 min, yields were determined by ^1H NMR using 1,3,5-trimethoxybenzene as an internal standard. NP = no product.

Scheme S4. Reactivity comparison in glycosylselenylation of cysteine-containing molecules

7. Oligopeptide modification

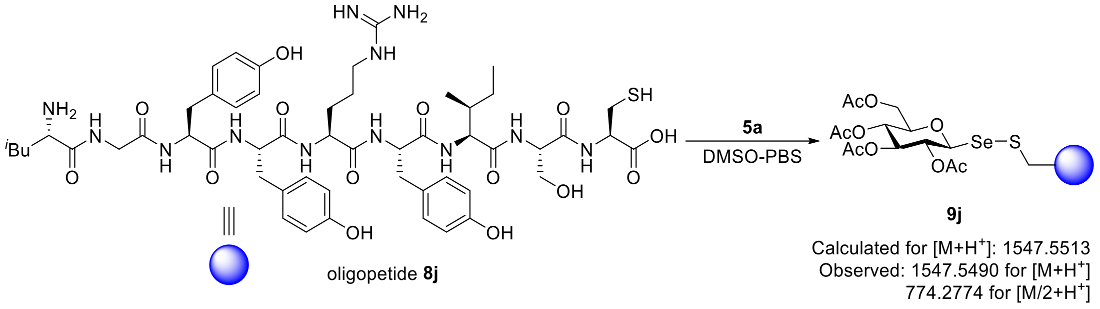

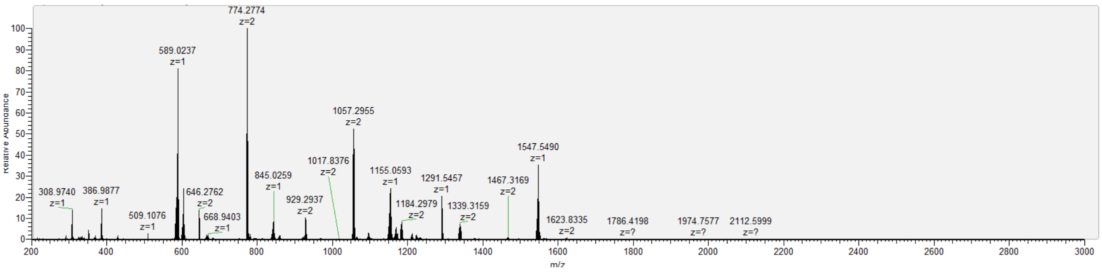

Figure S13. HRMS analysis for **9j**

9j was synthesized according to general procedure C with a modification of solvent using DMSO/PBS (pH 7.2) (v/v 1:4) for 5 min. The conversion of oligopeptide **8j** and the formation of product **9j** were determined by HRMS analysis. According to the result, the oligopeptide reactant was found to be completely consumed.

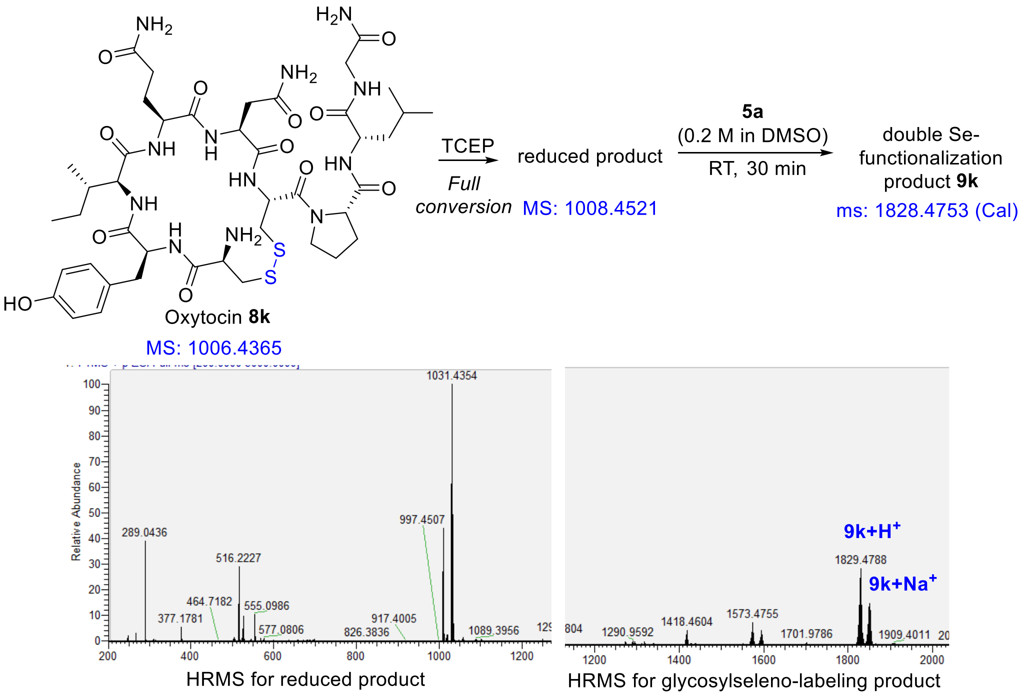

Figure S14. HRMS analysis for **9k**

Oligopeptide **8k** (10.9 mg, 0.01 mmol, 1.0 equiv.) TCEP solution (0.5 M, 50 μL) and H_2O (2 mL) was added to a 10 mL round flask. Then the mixture was stirred at room temperature for 12 h. After that, a DMSO solution of **5a** (0.2 M, 0.5 mL, 10.0 equiv.) was added to the flask. The mixture was stirred at room temperature for another 0.5 h. The formation of product **9k** were detected by HRMS analysis.

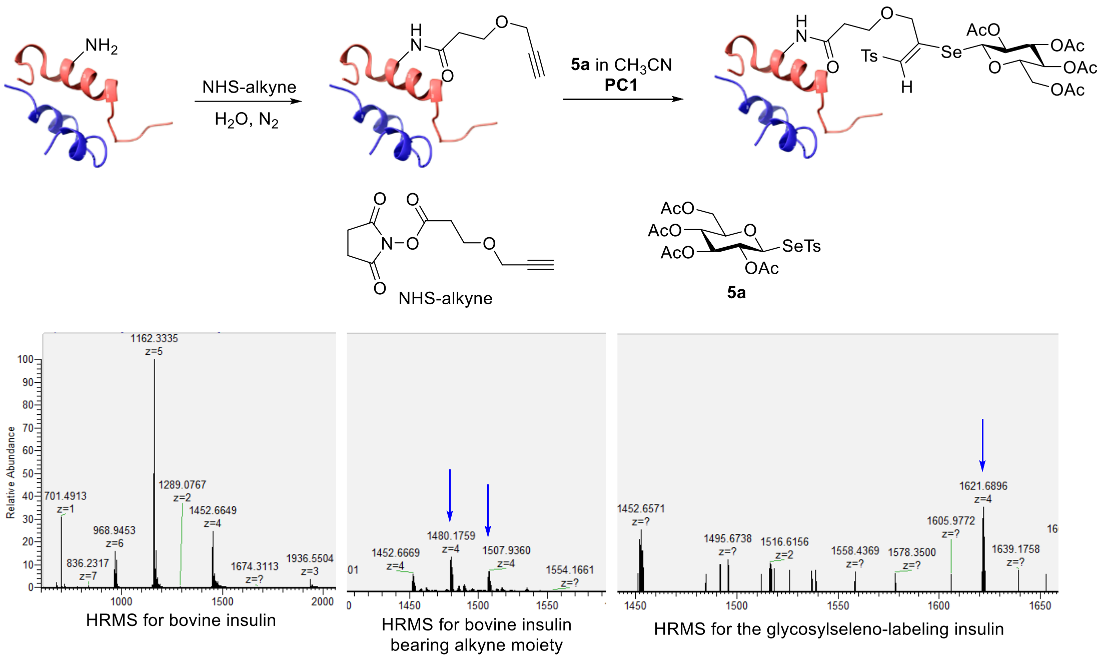

Figure S15. HRMS analysis for bovine insulin modification

A flask was charged with bovine insulin (5.0 mg, 0.9 μmol, 1.0 equiv.), propargyl-NHS ester (1.1 mg, 5 μmol, 5.6 equiv.), and H_2O (3.0 mL, bubbled with N_2 for 0.5 h). The reaction mixture was stirred at room temperature for 10 h under N_2. Then, the reaction mixture was added **PC1** (0.64 mg, 1.0 μmol, 1.1 equiv.) and a solution of **5a** (0.1 M, 0.5 mL, 50.0 equiv.), and stirred for another 30 min at room temperature under irradiation with a blue LED (450-465 nm). The product was determined by HRMS analysis.

8. The cytotoxicity of the substrates to 293T cells

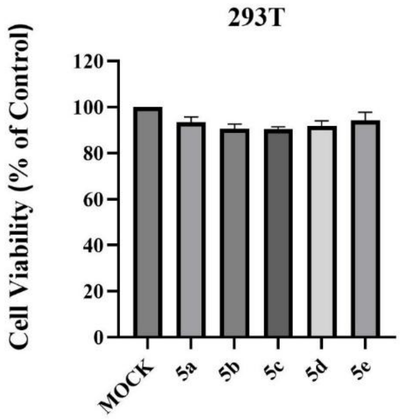

Figure S16. cytotoxicity of the substrates to 293T cells

Cytotoxicity was evaluated using the Cell Counting Kit-8 (CCK-8) assay. Briefly, 293T cells (human embryonic kidney cell line) were seeded into 96-well plates at a density of 3000 cells/well and incubated for 24 h. Subsequently, the cells were treated with various concentrations of the test compounds (prepared by dilution) and incubated for an additional 24 h. Following treatment, CCK-8 solution (MCE, Catalog

No. HY-K0301) was added to each well, and the plates were incubated for 1 h. The absorbance at 450 nm was then measured using a microplate reader. Cell viability was expressed as a percentage relative to untreated control cells. Data, such as the cell viability (%) at a 5 μM compound concentration, were plotted using GraphPad Prism 10 software. Results are presented as the mean ± standard deviation (SD) from three independent experiments. The results indicate that, at concentrations up to 5 μM, the substrates exhibited minimal impact on cell viability.

9. Experimental characterization data for substrates and products

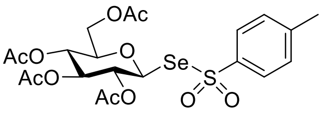

5a

(2*R*,3*R*,4*S*,5*R*,6*S*)-2-(acetoxymethyl)-6-(tosylselanyl)tetrahydro-2*H*-pyran-3,4,5-triyl triacetate

5a was synthesized according to general procedure A and isolated by column chromatography on silica gel using dichloromethane as the eluent, giving the title product as a white solid (235.2 mg, 65% yield). **^{1}H NMR (400 MHz, CDCl$_3$)** δ 7.82 – 7.75 (m, 2H), 7.32 (d, *J* = 8.1 Hz, 2H), 5.55 – 5.52 (m, 1H), 5.33 – 5.24 (m, 1H), 5.13 – 5.03 (m, 2H), 4.19 – 4.15 (m, 1H), 4.04 – 4.00 (m, 1H), 3.81 – 3.74 (m, 1H), 2.44 (s, 3H), 2.06 (s, 3H), 2.03 (s, 3H), 2.00 (s, 6H, *2CH$_3$*) ppm. **^{13}C NMR (101 MHz, CDCl$_3$)** δ 170.63, 170.04, 169.92, 169.52, 145.30, 129.88, 126.79, 86.41, 77.67, 73.58, 69.78, 68.03, 61.83, 21.80, 20.86, 20.68 ppm. **IR (thin film, cm^{-1})**: 2954, 2922, 2851, 2322, 2285, 1748, 1593, 1374, 1221, 1056, 810, 701, 646, 575. **$[\alpha]_D^{25}$** = +69.2 (c = 0.20, CHCl$_3$). **HRMS** (ESI-TOF): calculated for $C_{21}H_{26}O_{11}SSeNa^+$ [M+Na$^+$]: 589.0253, found 589.0252.

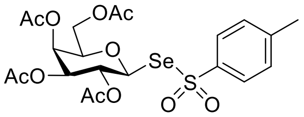

5b

(2*R*,3*S*,4*S*,5*R*,6*S*)-2-(acetoxymethyl)-6-(tosylselanyl)tetrahydro-2*H*-pyran-3,4,5-triyl triacetate

5b was synthesized according to general procedure A and isolated by column chromatography on silica gel using dichloromethane as the eluent, giving the title product as a white solid (235.2 mg, 65% yield). **^{1}H NMR (400 MHz, CDCl$_3$)** δ 7.83 – 7.76 (m, 2H), 7.33 (d, *J* = 8.1 Hz, 2H), 5.56 (d, *J* = 10.4 Hz, 1H), 5.46 (d, *J* = 3.3 Hz, 1H), 5.27 (t, *J* = 10.2 Hz, 1H), 5.14 – 5.11 (m, 1H), 4.09 – 3.91 (m, 3H), 2.45 (s, 3H), 2.11 (s, 3H), 2.06 (s, 3H), 2.02 (s, 3H), 1.98 (s, 3H) ppm. **^{13}C NMR (101 MHz, CDCl$_3$)** δ 170.43, 170.22, 170.16, 169.91, 145.38, 145.26, 129.87, 126.79, 87.12, 76.35, 71.54, 67.11, 66.94, 61.06, 21.80, 20.83, 20.80, 20.73, 20.66 ppm. **IR (thin film, cm^{-1})**: 2955, 2923, 2852, 2286, 1746, 1457, 1369, 1217, 1072,

811, 646, 599, 575, 483. **[α]D[25]** = +78.7 (c = 0.46, $CHCl_3$). **HRMS** (ESI-TOF): calculated for $C_{21}H_{26}O_{11}SSeNa^+$ [M+Na$^+$]: 589.0253, found 589.0250.

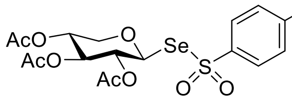

5c

(2*S*,3*R*,4*S*,5*R*)-2-(tosylselanyl)tetrahydro-2*H*-pyran-3,4,5-triyl triacetate

5c was synthesized according to general procedure A and isolated by column chromatography on silica gel using dichloromethane as the eluent, giving the title product as a white solid (183.2 mg, 58% yield). **^{1}H NMR (400 MHz, $CDCl_3$)** δ 7.80 – 7.73 (m, 2H), 7.30 (d, *J* = 8.1 Hz, 2H), 6.21 (d, *J* = 3.3 Hz, 1H), 5.05 (d, *J* = 4.5 Hz, 1H), 5.00 (t, *J* = 3.9 Hz, 1H), 4.69 (d, *J* = 3.7 Hz, 1H), 4.05 – 4.01 (m, 1H), 3.64 – 3.60 (m, 1H), 2.43 (s, 3H), 2.14 – 2.03 (m, 9H) ppm. **^{13}C NMR (101 MHz, $CDCl_3$)** δ 169.78, 169.32, 168.32, 145.37, 144.97, 129.81, 126.75, 87.12, 68.99, 66.40, 66.32, 62.81, 21.81, 20.96, 20.84, 20.78 ppm. **IR (thin film, cm^{-1})**: 2955, 2922, 2852, 2323, 2285, 2078, 1746, 1593, 1371, 1318, 1241, 1213, 1064, 905, 645, 574. **[α]D[25]** = -61.1 (c = 0.36, $CHCl_3$). **HRMS** (ESI-TOF): calculated for $C_{18}H_{22}O_9SSeNa^+$ [M+Na$^+$]: 517.0042, found 517.0043.

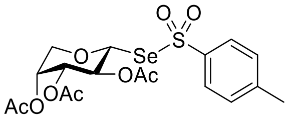

5d

(2*R*,3*S*,4*R*,5*R*)-2-(tosylselanyl)tetrahydro-2*H*-pyran-3,4,5-triyl triacetate

5d was synthesized according to general procedure A and isolated by column chromatography on silica gel using dichloromethane as the eluent, giving the title product as a white solid (249.5 mg, 79% yield). **^{1}H NMR (400 MHz, $CDCl_3$)** δ 7.76 (d, *J* = 8.3 Hz, 2H), 7.31 (d, *J* = 8.1 Hz, 2H), 6.16 (d, *J* = 2.9 Hz, 1H), 5.32 – 5.13 (m, 3H), 3.87 – 3.82 (m, 1H), 3.69 – 3.65 (m, 1H), 2.44 (s, 3H), 2.14-2.09 (m, 6H), 2.00 (s, 3H) ppm. **^{13}C NMR (101 MHz, $CDCl_3$)** δ 169.75, 169.25, 168.75, 145.36, 144.90, 129.84, 126.68, 86.98, 70.72, 66.77, 65.16, 61.26, 21.80, 20.89, 20.79 ppm. **IR (thin film, cm^{-1})**: 2955, 2922, 2321, 2049, 1749, 1459, 1371, 1214, 1061, 913, 807, 722, 646, 574, 511. **[α]D[25]** = +73.8 (c = 0.26, $CHCl_3$). **HRMS** (ESI-TOF): calculated for $C_{18}H_{22}O_9SSeNa^+$ [M+Na$^+$]: 517.0042, found 517.0042.

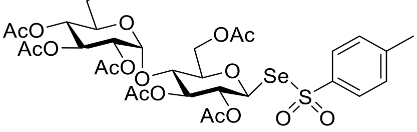

5e

(2*R*,3*R*,4*S*,5*R*,6*R*)-2-(acetoxymethyl)-6-(((2*R*,3*R*,4*S*,5*R*,6*S*)-4,5-diacetoxy-2-(acetoxymethyl)-6-

(tosylselanyl)tetrahydro-2*H*-pyran-3-yl)oxy)tetrahydro-2*H*-pyran-3,4,5-triyl triacetate

5e was synthesized according to general procedure A and isolated by column chromatography on silica gel using dichloromethane as the eluent, giving the title product as a white solid (267.7 mg, 49% yield). **^{1}H NMR (400 MHz, CDCl$_3$)** δ 7.74 (dd, *J* = 8.4, 2.1 Hz, 2H), 7.29 (d, *J* = 8.2 Hz, 2H), 5.53 – 5.46 (m, 1H), 5.33 – 5.24 (m, 3H), 5.02 – 4.97 (m, 1H), 4.92 – 4.87 (m, 1H), 4.81 – 4.77 (m, 1H), 4.26 – 4.13 (m, 2H), 4.10 – 4.03 (m, 1H), 4.00 – 3.85 (m, 3H), 3.72 – 3.65 (m, 1H), 2.40 (s, 3H), 2.06 (s, 3H), 2.03 (s, 3H), 2.00 (s, 3H), 1.99 – 1.90 (m, 12H) ppm. **^{13}C NMR (101 MHz, CDCl$_3$)** δ 170.54, 170.45, 170.19, 169.86, 169.84, 169.78, 169.37, 145.19, 145.03, 129.79, 126.66, 95.64, 85.86, 77.54, 75.59, 72.45, 70.30, 70.01, 69.19, 68.52, 68.00, 62.58, 61.48, 21.64, 20.81, 20.76, 20.65, 20.57, 20.55, 20.49 ppm. **IR (thin film, cm^{-1})**: 2955, 2924, 2166, 2049, 1981, 1747, 1457, 1370, 1322, 1226, 1135, 1040, 898, 812, 758, 647, 603, 576, 512, 476. **$[\alpha]_D^{25}$** = +63.7 (c = 0.46, CHCl$_3$). **HRMS** (ESI-TOF): calculated for $C_{33}H_{42}O_{19}SSeNa^+$ [M+Na$^+$]: 877.1098, found 877.1080.

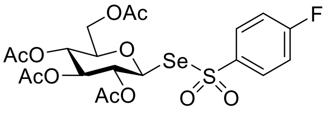

5f

(2*R*,3*R*,4*S*,5*R*,6*S*)-2-(acetoxymethyl)-6-(((4-fluorophenyl)sulfonyl)selanyl)tetrahydro-2*H*-pyran-3,4,5-triyl triacetate

5f was synthesized according to general procedure A and isolated by column chromatography on silica gel using dichloromethane as the eluent, giving the title product as a white solid (215.0 mg, 59% yield). **^{1}H NMR (400 MHz, CDCl$_3$)** δ 7.98 – 7.91 (m, 2H), 7.21 (t, *J* = 8.0 Hz, 2H), 5.58 (d, *J* = 10.5 Hz, 1H), 5.30 (t, *J* = 9.3 Hz, 1H), 5.11 – 5.05 (m, 2H), 4.19 – 4.14 (m, 1H), 4.10 (d, *J* = 12.7 Hz, 1H), 3.82 – 3.78 (m, 1H), 2.07 (s, 3H), 2.04 (s, 3H), 2.02 (s, 3H), 2.01 (s, 3H). **^{13}C NMR (101 MHz, CDCl$_3$)** δ 170.55, 170.01, 169.98, 169.54, 165.72 (d, *J* = 257.4 Hz), 144.01 (d, *J* = 3.3 Hz), 129.69 (d, *J* = 9.7 Hz), 116.57 (d, *J* = 22.9 Hz), 86.51, 77.78, 73.41, 69.67, 67.98, 61.77, 20.86, 20.71, 20.69, 20.69. **IR (thin film, cm^{-1}):** 2957, 2926, 2229, 2185, 2170, 2049, 2034, 2005, 1748, 1597, 1500, 1457, 1377, 1223, 1137, 1083, 1047, 900, 800, 762, 720. **$[\alpha]_D^{25}$** = -3.6 (c = 0.10, CHCl$_3$). **HRMS** (ESI-TOF): calculated for $C_{20}H_{23}FO_{11}SSeNa^+$ [M+Na$^+$]:593.0003, found 593.0006.

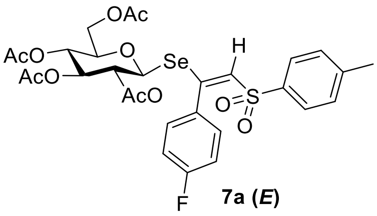

7a (*E*)

(2*R*,3*R*,4*S*,5*R*,6*S*)-2-(acetoxymethyl)-6-(((*E*)-1-(4-fluorophenyl)-2-tosylvinyl)selanyl)tetrahydro-2*H*-pyran-3,4,5-triyl triacetate

7a (*E*) was synthesized according to general procedure B and isolated by column chromatography on silica gel using petroleum ether/ethyl acetate (3:1 v/v) as the eluent, giving the title product as a white solid (64.6mg, 94% yield).

^{1}H NMR (400 MHz, CDCl$_3$) δ 7.44 (d, *J* = 8.4 Hz, 2H), 7.22 – 7.15 (m, 4H), 7.00 (t, *J* = 8.6 Hz, 2H), 6.91 (s, 1H), 5.11 – 5.02 (m, 2H), 5.02 – 4.95 (m, 1H), 4.56 – 4.47 (m, 1H), 4.16 – 4.12 (m, 1H), 4.04 – 4.04 – 4.00 (m, 1H), 3.37 – 3.32 (m, 1H), 2.39 (s, 3H), 2.15 (s, 3H), 2.03 – 1.96 (m, 9H) ppm. **^{13}C NMR (101 MHz, CDCl$_3$)** δ 170.78, 170.12, 169.52, 169.37, 163.49 (d, *J* = 251.2 Hz), 150.10, 144.43, 138.34, 131.14 (d, *J* = 8.5 Hz), 130.21, 130.19 (d, *J* = 1.7 Hz), 129.72, 127.74, 115.29 (d, *J* = 21.8 Hz), 80.68, 77.38, 73.48, 69.82, 67.92, 62.04, 21.73, 20.87, 20.74, 20.66, 20.64 ppm. **IR (thin film, cm^{-1}):** 2954, 2922, 2853, 2285, 2164, 2051, 1981, 1751, 1460, 1376, 1259, 1225, 1085, 1018, 801, 556. **[α]$_D$^{25}** = -3.6 (c = 0.11, CHCl$_3$). **HRMS** (ESI-TOF): calculated for $C_{29}H_{32}FO_{11}SSe^+$ [M+H$^+$]: 687.0809, found 687.0802.

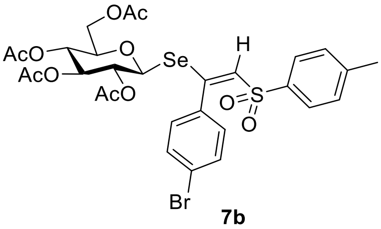

7b

(2*R*,3*R*,4*S*,5*R*,6*S*)-2-(acetoxymethyl)-6-(((*E*)-1-(4-bromophenyl)-2-tosylvinyl)selanyl)tetrahydro-2*H*-pyran-3,4,5-triyl triacetate

7b was synthesized according to general procedure B and isolated by column chromatography on silica gel using petroleum ether/ethyl acetate (3:1 v/v) as the eluent, giving the title product as a white solid (61.5 mg, 82% yield).

^{1}H NMR (400 MHz, CDCl$_3$) δ 7.45 – 7.42 (m, 4H), 7.20 (d, *J* = 8.1 Hz, 2H), 7.05 (d, *J* = 8.4 Hz, 2H), 6.91 (s, 1H), 5.11 – 5.02 (m, 2H), 5.03 – 4.93 (m, 1H), 4.56 – 4.49 (m, 1H), 4.18 – 4.08 (m, 1H), 4.03 – 3.99 (m, 1H), 3.37 – 3.33 (m, 1H), 2.40 (s, 3H), 2.14 (s, 3H), 2.01 (s, 3H), 2.00 (s, 3 H), 1.98 (s, 3H) ppm. **^{13}C NMR (101 MHz, CDCl$_3$)** δ 170.76, 170.10, 169.51, 169.36, 149.50, 144.53, 138.17, 133.19, 131.31, 130.50, 130.38, 129.75, 127.78, 124.31, 80.59, 77.36, 73.43, 69.81, 67.89, 62.02, 21.73, 20.86,

20.71, 20.63, 20.63 ppm. **IR (thin film, cm^{-1}):** 2955, 2922, 2852, 2643, 2284, 2192, 2165, 2100, 2049, 2014, 1981, 1751, 1460, 1376, 1222, 1085, 1058, 802, 555, 462. **$[\alpha]_D^{25}$** = -8.3 (c = 0.09, $CHCl_3$). **HRMS** (ESI-TOF): $C_{29}H_{32}BrO_{11}SSe^+$ calculated for $[M+H^+]$: 747.0008, found 746.9999.

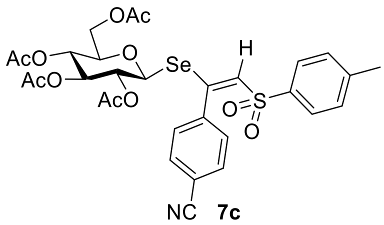

7c

(2*R*,3*R*,4*S*,5*R*,6*S*)-2-(acetoxymethyl)-6-(((*E*)-1-(4-cyanophenyl)-2-tosylvinyl)selanyl)tetrahydro-2*H*-pyran-3,4,5-triyl triacetate

7c was synthesized according to general procedure B (with reaction time 1 h) and isolated by column chromatography on silica gel using petroleum ether/ethyl acetate (3:1 v/v) as the eluent, giving the title product as a white solid (31.2 mg, 45% yield).

^{1}H NMR (400 MHz, $CDCl_3$) δ 7.61 (d, *J* = 7.9 Hz, 2H), 7.46 (d, *J* = 8.0 Hz, 2H), 7.31 (d, *J* = 8.0 Hz, 2H), 7.23 (d, *J* = 9.2 Hz, 2H), 6.92 (s, 1H), 5.09 – 5.01 (m, 2H), 4.96 (t, *J* = 9.5 Hz, 1H), 4.56 (d, *J* = 9.6 Hz, 1H), 4.15 – 4.11 (m, 1H), 4.03 – 4.00 (m, 1H), 3.43 – 3.35 (m, 1H), 2.40 (s, 3H), 2.14 (s, 3H), 2.00 – 1.97 (m, 9H) ppm. **^{13}C NMR (101 MHz, $CDCl_3$)** δ 170.69, 170.04, 169.52, 169.34, 147.92, 144.91, 139.32, 138.06, 131.79, 130.92, 129.99, 129.64, 127.74, 118.19, 113.53, 80.63, 77.36, 73.36, 69.81, 67.89, 62.05, 21.78, 20.86, 20.71, 20.63 ppm. **IR (thin film, cm^{-1}):** 2955, 2922, 2852, 2322, 2229, 2165, 2050, 1981, 1751, 1595, 1494, 1459, 1375, 1222, 1148, 1084, 1043, 907, 841, 802, 760, 659, 600, 559, 527. **$[\alpha]_D^{25}$** = -1.4 (c = 0.11, $CHCl_3$). **HRMS** (ESI-TOF): calculated for $C_{30}H_{32}NO_{11}SSe^+$ $[M+H^+]$: 694.0856, found 694.0850.

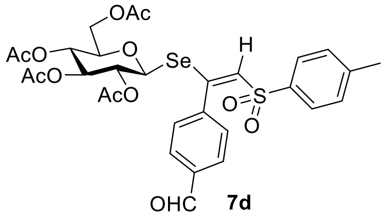

7d

(2*R*,3*R*,4*S*,5*R*,6*S*)-2-(acetoxymethyl)-6-(((*E*)-1-(4-formylphenyl)-2-tosylvinyl)selanyl)tetrahydro-2H-pyran-3,4,5-triyl triacetate

7d was synthesized according to general procedure B and isolated by column chromatography on silica gel using petroleum ether/ethyl acetate (3:1 v/v) as the eluent, giving the title product as a white solid (54.6 mg, 79% yield).

^{1}H NMR (400 MHz, $CDCl_3$) δ 10.06 (s, 1H), 7.85 (d, *J* = 7.9 Hz, 2H), 7.47 (d, *J* = 8.0 Hz, 2H), 7.38 (d,

J = 7.9 Hz, 2H), 7.21 (d, *J* = 8.0 Hz, 2H), 6.93 (s, 1H), 5.10 – 5.03 (m, 2H), 5.00 – 4.94 (m, 1H), 4.56 – 4.47 (m, 1H), 4.15 – 4.11 (m, 1H), 4.03 – 3.99 (m, 1H), 3.35 – 3.30 (m, 1H), 2.40 (s, 3H), 2.16 (s, 3H), 2.03 – 1.97 (m, 9H) ppm. **^{13}C NMR (101 MHz, CDCl$_3$)** δ 191.48, 170.72, 170.08, 169.54, 169.35, 148.90, 144.76, 140.48, 138.13, 136.83, 130.53, 129.91, 129.55, 129.22, 127.77, 80.59, 77.40, 73.37, 69.80, 67.89, 62.06, 21.76, 20.87, 20.72, 20.63 ppm. **IR (thin film, cm^{-1}):** 2955, 2923, 2852, 2647, 2285, 2165, 2076, 2049, 2012, 1981, 1752, 1702, 1577, 1459, 1376, 1222, 1148, 1085, 1056, 907, 811, 762, 651, 600, 555, 442. **[α]$_D^{25}$** = +1.8 (c = 0.11, CHCl$_3$). **HRMS** (ESI-TOF): calculated for $C_{30}H_{33}O_{12}SSe^+$ [M+H$^+$]: 697.0852, found 697.0851.

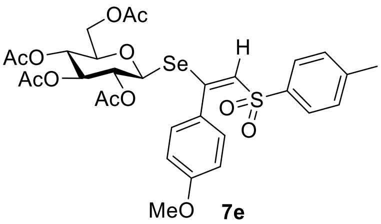

7e

(2*R*,3*R*,4*S*,5*R*,6*S*)-2-(acetoxymethyl)-6-(((*E*)-1-(4-methoxyphenyl)-2-tosylvinyl)selanyl)tetrahydro-2*H*-pyran-3,4,5-triyl triacetate

7e was synthesized according to general procedure B and isolated by column chromatography on silica gel using petroleum ether/ethyl acetate (3:1 v/v) as the eluent, giving the title product as a white solid (65.0 mg, 93% yield).

^{1}H NMR (500 MHz, CDCl$_3$) δ 7.44 (dd, *J* = 8.3, 1.5 Hz, 2H), 7.20 – 7.11 (m, 4H), 6.87 (d, *J* = 1.5 Hz, 1H), 6.82 (dd, *J* = 8.7, 1.5 Hz, 2H), 5.09 – 5.02 (m, 2H), 5.01 – 4.94 (m, 1H), 4.49 – 4.43 (m, 1H), 4.13 – 4.11 (m, 1H), 4.02 - 3.99 (m, 1H), 3.85 (s, 3H), 3.32 – 3.27 (m, 1H), 2.38 (s, 3H), 2.14 (s, 3H), 2.03 (s, 3H), 1.99 – 1.97 (m, 6H) ppm. **^{13}C NMR (101 MHz, CDCl$_3$)** δ 170.82, 170.18, 169.51, 169.36, 160.95, 151.57, 144.12, 138.56, 130.81, 129.58, 129.25, 127.75, 126.22, 113.48, 80.84, 77.26, 73.57, 69.87, 67.97, 62.02, 55.49, 21.70, 20.87, 20.76, 20.66, 20.63 ppm. **IR (thin film, cm^{-1}):** 3726, 2954, 2922, 2852, 2647, 2323, 2285, 2186, 2165, 2111, 2079, 2050, 1981, 1752, 1604, 1503, 1459, 1375, 1291, 1223, 1138, 1084, 1035, 897, 802, 556, 487, 463, 449, 430. **[α]$_D^{25}$** = +2.2 (c = 0.09, CHCl$_3$). **HRMS** (ESI-TOF): calculated for $C_{30}H_{35}O_{12}SSe^+$ [M+H$^+$]: 699.1009, found 699.1004.

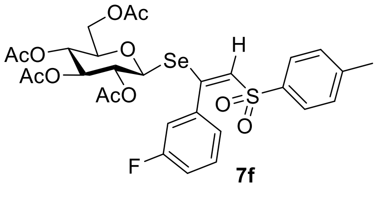

7f

(2*R*,3*R*,4*S*,5*R*,6*S*)-2-(acetoxymethyl)-6-(((*E*)-1-(3-fluorophenyl)-2-tosylvinyl)selanyl)tetrahydro-2*H*-pyran-3,4,5-triyl triacetate

7f was synthesized according to general procedure B and isolated by column chromatography on silica gel using petroleum ether/ethyl acetate (3:1 v/v) as the eluent, giving the title product as a white solid (54.8 mg, 80% yield).

^{1}H NMR (400 MHz, CDCl$_3$) δ 7.45 (d, *J* = 8.3 Hz, 2H), 7.32 – 7.28 (m, 1H), 7.20 (d, *J* = 7.9 Hz, 2H), 7.08 – 7.07 (m, 1H), 7.01 (dt, *J* = 7.7, 1.3 Hz, 1H), 6.92 (s, 1H), 6.82 – 6.79 (m, 1H), 5.11 – 4.99 (m, 2H), 5.02 – 4.93 (m, 1H), 4.53 – 4.43 (m, 1H), 4.15 – 4.09 (m, 1H), 4.05 – 4.01 (m, 1H), 3.35 – 3.32 (m, 1H), 2.40 (s, 3H), 2.15 (s, 3H), 2.04 – 1.97 (m, 9H) ppm. **^{13}C NMR (101 MHz, CDCl$_3$)** δ 170.82, 170.11, 169.55, 169.36, 162.07 (d, *J* = 248.0 Hz), 149.04 (d, *J* = 2.3 Hz), 144.56, 138.15, 136.20 (d, *J* = 8.1 Hz), 130.66, 129.82, 129.74, 127.82, 124.85 (d, *J* = 3.0 Hz), 116.74 (d, *J* = 20.9 Hz), 115.89 (d, *J* = 23.1 Hz), 80.62, 77.44, 73.45, 69.77, 67.91, 62.05, 21.69, 20.84, 20.71, 20.64, 20.62 ppm. **IR (thin film, cm^{-1}):** 2955, 2923, 2852, 2641, 2322, 2284, 2187, 2164, 2050, 1983, 1751, 1583, 1459, 1376, 1290, 1222, 1147, 1084, 1056, 953, 909, 847, 791, 759, 682, 601, 554, 536, 435. **$[\alpha]_D^{25}$** = -3.6 (c = 0.14, CHCl$_3$). **HRMS** (ESI-TOF): calculated for $C_{29}H_{32}FO_{11}SSe^+$ [M+H$^+$]: 687.0801, found 687.0809.

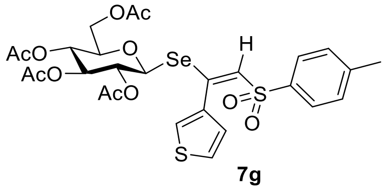

7g

(2*R*,3*R*,4*S*,5*R*,6*S*)-2-(acetoxymethyl)-6-(((*E*)-1-(thiophen-3-yl)-2-tosylvinyl)selanyl)tetrahydro-2*H*-pyran-3,4,5-triyl triacetate

7g was synthesized according to general procedure B and isolated by column chromatography on silica gel using petroleum ether/ethyl acetate (3:1 v/v) as the eluent, giving the title product as a white solid (57.7 mg, 86% yield).

^{1}H NMR (400 MHz, CDCl$_3$) δ 7.44 (d, *J* = 8.3 Hz, 2H), 7.39 (dd, *J* = 3.0, 1.3 Hz, 1H), 7.23 (dd, *J* = 5.0, 3.0 Hz, 1H), 7.17 (d, *J* = 7.8 Hz, 2H), 6.96 – 6.88 (m, 2H), 5.13 – 5.01 (m, 2H), 5.03 – 4.93 (m, 1H), 4.56 – 4.49 (m, 1H), 4.15 – 4.11 (m, 1H), 4.05 – 4.01 (m, 1H), 3.38 – 3.34 (m, 1H), 2.37 (s, 3H), 2.14 (s, 3H), 2.03 (s, 3H), 1.99 – 1.98 (m, 6H) ppm. **^{13}C NMR (101 MHz, CDCl$_3$)** δ 170.79, 170.13, 169.51, 169.36, 145.62, 144.19, 138.23, 134.01, 130.37, 129.57, 128.56, 127.98, 127.64, 125.58, 80.88, 77.38, 73.54, 69.88, 67.98, 62.07, 21.70, 20.88, 20.74, 20.65, 20.63 ppm. **IR (thin film, cm^{-1}):** 2955, 2922, 2852, 2651, 2322, 2286, 2167, 2051, 1981, 1750, 1595, 1459, 1375, 1221, 1148, 958, 909, 853, 802, 760, 702, 659, 600, 556, 456. **$[\alpha]_D^{25}$** = -10.9 (c = 0.11, CHCl$_3$). **HRMS** (ESI-TOF): calculated for $C_{27}H_{31}O_{11}S_2Se^+$ [M+H$^+$]: 675.0468, found 675.0465.

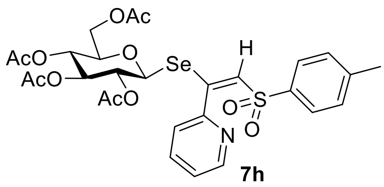

7h

(2*R*,3*R*,4*S*,5*R*,6*S*)-2-(acetoxymethyl)-6-(((*E*)-1-(pyridin-2-yl)-2-tosylvinyl)selanyl)tetrahydro-2*H*-pyran-3,4,5-triyl triacetate

7h was synthesized according to general procedure B and isolated by column chromatography on silica gel using petroleum ether/ethyl acetate (3:1 v/v) as the eluent, giving the title product as a white solid (53.6 mg, 80% yield).

^{1}H NMR (400 MHz, CDCl$_3$) δ 8.60 – 8.55 (m, 1H), 7.76 – 7.72 (m, 1H), 7.61 (d, *J* = 8.3 Hz, 2H), 7.54 (d, *J* = 7.8 Hz, 1H), 7.31 – 7.28 (m, 1H), 7.25 (d, *J* = 8.7 Hz, 2H), 6.83 (s, 1H), 5.10 – 5.08 (m, 2H), 5.00 (t, *J* = 9.5 Hz, 1H), 4.75 (d, *J* = 9.8 Hz, 1H), 4.13 – 4.08 (m, 1H), 3.94 – 3.90 (m, 1H), 3.38 – 3.35 (m, 1H), 2.40 (s, 3H), 2.12 (s, 3H), 1.99 – 1.97 (m, 9H) ppm. **^{13}C NMR (101 MHz, CDCl$_3$)** δ 170.83, 170.14, 169.68, 169.38, 153.33, 149.27, 149.03, 144.60, 138.06, 136.05, 129.91, 129.68, 127.86, 125.29, 124.02, 80.05, 77.37, 73.53, 69.98, 67.99, 61.96, 21.75, 20.85, 20.74, 20.64, 20.64, 20.64 ppm. **IR (thin film, cm^{-1}):** 2954, 2922, 2852, 2644, 2322, 2286, 2164, 2051, 1982, 1750, 1595, 1460, 1430, 1375, 1325, 1290, 1222, 1147, 1084, 1043, 909, 801, 760, 651, 600, 556, 530, 491. **[α]$_D^{25}$** = -41.4 (c = 0.11, CHCl$_3$). **HRMS** (ESI-TOF): calculated for $C_{28}H_{32}NO_{11}SSe^+$ [M+H$^+$]: 670.0856, found 670.0845.

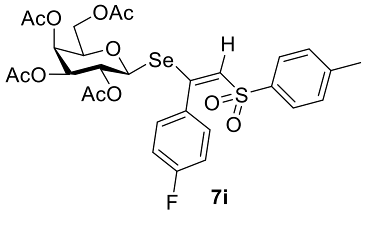

7i

(2*R*,3*S*,4*S*,5*R*,6*S*)-2-(acetoxymethyl)-6-(((*E*)-1-(4-fluorophenyl)-2-tosylvinyl)selanyl)tetrahydro-2*H*-pyran-3,4,5-triyl triacetate

7i was synthesized according to general procedure B and isolated by column chromatography on silica gel using petroleum ether/ethyl acetate (3:1 v/v) as the eluent, giving the title product as a white solid (51.3 mg, 75% yield).

^{1}H NMR (400 MHz, CDCl$_3$) δ 7.42 (d, *J* = 8.2 Hz, 2H), 7.20 – 7.12 (m, 4H), 7.01 – 6.94 (m, 3H), 5.43 – 5.33 (m, 2H), 4.96 – 4.92 (m, 1H), 4.56 (d, *J* = 10.3 Hz, 1H), 4.08 – 4.06 (m, 2H), 3.67 (t, *J* = 6.3 Hz, 1H), 2.39 (s, 3H), 2.17 (s, 3H), 2.11 (s, 3H), 2.04 (s, 3H), 1.98 (s, 3H) ppm. **^{13}C NMR (101 MHz, CDCl$_3$)** δ 170.56, 170.31, 170.00, 169.70, 163.44 (d, *J* = 251.0 Hz), 150.18, 144.30, 138.45, 131.05 (d, *J* = 8.6 Hz), 130.30, 130.27 (d, *J* = 1.0 Hz), 129.62, 127.75, 115.26 (d, *J* = 22.0 Hz), 81.21, 76.25, 71.52, 67.24, 66.97, 61.75, 21.71, 20.84, 20.81, 20.73, 20.65 ppm. **IR (thin film, cm^{-1}):** 2955, 2921, 2852, 2286, 2187,

2051, 1981, 1749, 1600, 1501, 1459, 1372, 1288, 1220, 1138, 1082, 1056, 1018, 949, 900, 841, 810, 759, 720, 645, 585, 555, 531, 492. **[α]D25** = +6.3 (c = 0.12, $CHCl_3$). **HRMS** (ESI-TOF): calculated for $C_{29}H_{32}FO_{11}SSe^+$ [M+H+]: 687.0809, found 687.0804.

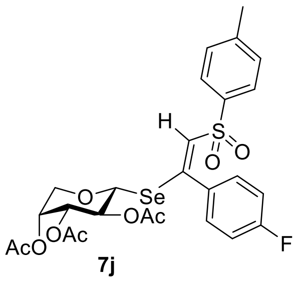

7j

(2*R*,3*S*,4*R*,5*R*)-2-(((*E*)-1-(4-fluorophenyl)-2-tosylvinyl)selanyl)tetrahydro-2*H*-pyran-3,4,5-triyl triacetate

7j was synthesized according to general procedure B and isolated by column chromatography on silica gel using petroleum ether/ethyl acetate (3:1 v/v) as the eluent, giving the title product as a white solid (48.4 mg, 79% yield).

^{1}H NMR (400 MHz, $CDCl_3$) δ 7.42 (d, *J* = 8.3 Hz, 2H), 7.21 – 7.12 (m, 4H), 6.98 (t, *J* = 8.6 Hz, 2H), 6.93 (s, 1H), 5.25 – 5.17 (m, 2H), 5.14 – 5.12 (m, 1H), 5.06 (d, *J* = 4.7 Hz, 1H), 4.05 – 4.00 (m, 1H), 3.61 – 3.57 (m, 1H), 2.38 (s, 3H), 2.09 – 2.07 (m, 9H) ppm. **^{13}C NMR (101 MHz, $CDCl_3$)** δ 169.97, 169.39, 169.32, 163.44 (d, *J* = 250.7 Hz), 151.57, 144.23, 138.43, 131.10 (d, *J* = 8.5 Hz), 130.53 (d, *J* = 3.4 Hz), 129.60, 129.33, 127.74, 115.24 (d, *J* = 21.8 Hz), 81.25, 69.73, 68.07, 65.98, 63.08, 21.69, 20.89, 20.85, 20.81 ppm. **IR (thin film, cm^{-1}):** 2955, 2923, 2853, 2322, 2164, 2051, 1981, 1749, 1600, 1500, 1459, 1373, 1290, 1220, 1140, 1084, 1057, 901, 870, 841, 810, 759, 670, 555, 531. **$[\alpha]_D^{25}$** = +91.0 (c = 0.10, $CHCl_3$). **HRMS** (ESI-TOF): calculated for $C_{26}H_{28}FO_9SSe^+$ [M+H$^+$]: 615.0598, found 615.0593.

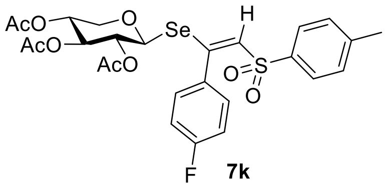

7k

(2*S*,3*R*,4*S*,5*R*)-2-(((*E*)-1-(4-fluorophenyl)-2-tosylvinyl)selanyl)tetrahydro-2*H*-pyran-3,4,5-triyl triacetate

7k was synthesized according to general procedure B and isolated by column chromatography on silica gel using petroleum ether/ethyl acetate (3:1 v/v) as the eluent, giving the title product as a white solid (50.5 mg, 82% yield).

^{1}H NMR (400 MHz, $CDCl_3$) δ 7.45 (d, *J* = 8.3 Hz, 2H), 7.24 – 7.17 (m, 4H), 7.00 (t, *J* = 8.7 Hz, 2H), 6.89 (s, 1H), 5.12 (d, *J* = 4.6 Hz, 1H), 5.00 (t, *J* = 5.3 Hz, 1H), 4.94 (t, *J* = 4.9 Hz, 1H), 4.80 – 4.75 (m,

1H), 4.25 – 4.21 (m, 1H), 3.51 – 3.47 (m, 1H), 2.40 (s, 3H), 2.08 – 2.06 (m, 9H) ppm. **^{13}C NMR (101 MHz, $CDCl_3$)** δ 169.75, 169.40, 168.93, 163.52 (d, *J* = 250.7 Hz), 151.16, 144.35, 138.40, 131.14 (d, *J* = 8.6 Hz), 130.51 (d, *J* = 3.4 Hz), 129.70, 129.38, 127.79, 115.32 (d, *J* = 22.0 Hz), 81.27, 69.23, 67.96, 67.01, 63.64, 21.73, 20.92, 20.86, 20.83 ppm. **IR (thin film, cm^{-1}):** 2955, 2923, 2853, 2645, 2322, 2287, 2084, 2051, 1981, 1750, 1600, 1500, 1460, 1373, 1292, 1220, 1148, 1083, 1063, 937, 901, 873, 840, 810, 760, 671, 646, 601, 555, 530, 432. **$[\alpha]_D^{25}$** = -72.7 (c = 0.15, $CHCl_3$). **HRMS** (ESI-TOF): calculated for $C_{26}H_{28}FO_9SSe^+$ [M+H$^+$]: 615.0598, found 615.0594.

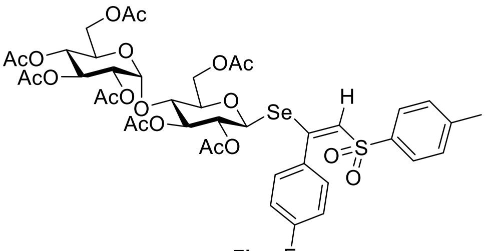

7l

(2*R*,3*R*,4*S*,5*R*,6*R*)-2-(acetoxymethyl)-6-(((2*R*,3*R*,4*S*,5*R*,6*S*)-4,5-diacetoxy-2-(acetoxymethyl)-6-(((*E*)-1-(4-fluorophenyl)-2-tosylvinyl)selanyl)tetrahydro-2*H*-pyran-3-yl)oxy)tetrahydro-2*H*-pyran-3,4,5-triyl triacetate

7l was synthesized according to general procedure B and isolated by column chromatography on silica gel using petroleum ether/ethyl acetate (1:2 v/v) as the eluent, giving the title product as a white solid (88.6 mg, 91% yield).

^{1}H NMR (400 MHz, $CDCl_3$) δ 7.44 (d, *J* = 7.9 Hz, 2H), 7.22 – 7.16 (m, 4H), 7.01 (t, *J* = 8.3 Hz, 2H), 6.92 (s, 1H), 5.35 – 5.24 (m, 2H), 5.11 (t, *J* = 8.8 Hz, 1H), 5.02 (t, *J* = 9.9 Hz, 1H), 4.92 (t, *J* = 9.6 Hz, 1H), 4.86 – 4.78 (m, 1H), 4.54 (d, *J* = 10.2 Hz, 1H), 4.38 – 4.30 (m, 1H), 4.27 – 4.18 (m, 1H), 4.14 – 4.00 (m, 2H), 3.94 – 3.82 (m, 2H), 3.35 – 3.29 (m, 1H), 2.39 (s, 3H), 2.20 (s, 3H), 2.09 (s, 3H), 2.05 – 1.95 (m, 15H). **^{13}C NMR (101 MHz, $CDCl_3$)** δ 170.63, 170.59, 170.08, 170.02, 169.66, 169.54, 163.50 (d, *J* = 251.2 Hz), 149.96, 144.39, 138.31, 131.18 (d, *J* = 8.5 Hz), 130.34, 130.14 (d, *J* = 3.3 Hz), 129.70, 127.75, 115.32 (d, *J* = 21.9 Hz), 95.86, 80.25, 77.43, 75.90, 72.52, 70.59, 70.07, 69.35, 68.81, 68.09, 62.87, 61.63, 21.70, 20.95, 20.93, 20.79, 20.68, 20.65. ppm. **IR (thin film, cm^{-1})**: 3020, 2276, 2207, 2165, 2114, 2043, 2008, 1214, 1014, 747, 568. **$[\alpha]_D^{25}$** = +19.0 (c = 0.10, $CHCl_3$). **HRMS** (ESI-TOF): calculated for $C_{41}H_{47}FO_{19}SSeNa^+$ [M+Na$^+$]: 997.1474, found 997.1455.

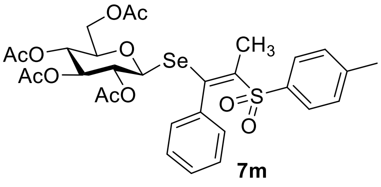

7m

(2*R*,3*R*,4*S*,5*R*,6*S*)-2-(acetoxymethyl)-6-(((*E*)-1-phenyl-2-tosylprop-1-en-1-yl)selanyl)tetrahydro-2*H*-pyran-3,4,5-triyl triacetate

7m was synthesized according to general procedure B and isolated by column chromatography on silica gel using petroleum ether/ethyl acetate (3:1 v/v) as the eluent, giving the title product as a white solid (46.1 mg, 67% yield).

^{1}H NMR (400 MHz, CDCl$_3$) δ 7.39 – 7.33 (m, 4H), 7.26 – 7.22 (m, 1H), 7.19 – 7.14 (m, 2H), 7.11 – 7.06 (m, 2H), 5.05 – 4.98 (m, 1H), 4.93 (t, *J* = 9.7 Hz, 1H), 4.83 (t, *J* = 9.2 Hz, 1H), 4.08 – 4.04 (m, 1H), 3.91 – 3.88 (m, 1H), 3.69 (d, *J* = 10.6 Hz, 1H), 2.72 – 2.68 (m, 1H), 2.38 (s, 3H), 2.22 (s, 3H), 2.10 (s, 3H), 2.01 (s, 3H), 1.94 (s, 6H, *2CH$_3$*) ppm. **^{13}C NMR (101 MHz, CDCl$_3$)** δ 170.63, 170.20, 169.62, 169.32, 147.01, 144.00, 138.08, 135.38, 134.73, 129.58, 129.19 (d, *J* = 37.1 Hz), 128.73, 127.87, 127.65(d, *J* = 97.9 Hz), 80.65, 76.76, 73.63, 69.37, 67.85, 61.87, 21.69, 20.85, 20.80, 20.64, 20.60, 18.63. **IR (thin film, cm^{-1}):** 2955, 2922, 2852, 2285, 2166, 2050, 2013, 1981, 1750, 1593, 1459, 1375, 1316, 1220, 1155, 1081, 1041, 909, 800, 762, 716, 697, 662, 599, 563, 535, 486, 458. **$[\alpha]_D^{25}$** = +76.0 (c = 0.10, CHCl$_3$). **HRMS** (ESI-TOF): calculated for $C_{30}H_{35}O_{11}SSe^+$ [M+H$^+$]: 683.1060, found 683.1052.

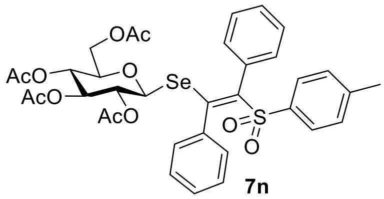

7n

(2*R*,3*R*,4*S*,5*R*,6*S*)-2-(acetoxymethyl)-6-(((*E*)-1,2-diphenyl-2-tosylvinyl)selanyl)tetrahydro-2*H*-pyran-3,4,5-triyl triacetate

7n was synthesized according to general procedure B (with reaction time 10 min) and isolated by column chromatography on silica gel using petroleum ether/ethyl acetate (3:1 v/v) as the eluent, giving the title product as a white solid (49.8 mg, 67% yield).

^{1}H NMR (500 MHz, CDCl$_3$) δ 7.52 – 7.29 (m, 8H), 7.25 – 7.14 (m, 4H), 7.12 – 7.07 (m, 2H), 4.89 – 4.75 (m, 3H), 4.08 – 4.05 (m, 1H), 3.93 – 3.90 (m, 1H), 3.69 (d, *J* = 10.0 Hz, 1H), 2.69 – 2.67 (m, 1H), 2.36 (s, 3H), 2.12 (s, 3H), 1.95 – 1.90 (m, 9H) ppm. **^{13}C NMR (101 MHz, CDCl$_3$)** δ 170.66, 170.23, 169.32, 169.30, 150.57, 144.01, 139.83, 137.64, 134.98, 134.00, 130.87, 129.77, 129.28, 128.97, 128.96 (d, *J* = 47.2 Hz), 128.86, 128.51, 127.84 (d, *J* = 84.8 Hz), 80.74, 76.72, 73.78, 69.24, 67.85, 61.90, 21.71, 20.90, 20.72, 20.63, 20.60 ppm. **IR (thin film, cm^{-1}):** 2954, 2923, 2852, 2646, 2322, 2286, 2165, 2077, 2050, 1981, 1752, 1597, 1459, 1375, 1319, 1222, 1148, 1085, 1053, 962, 909, 790, 757, 699, 675, 634, 588, 566, 460. **$[\alpha]_D^{25}$** = +48.3 (c = 0.09, CHCl$_3$). **HRMS** (ESI-TOF): calculated for $C_{35}H_{37}O_{11}SSe^+$ [M+H$^+$]: 745.1216, found 745.1212.

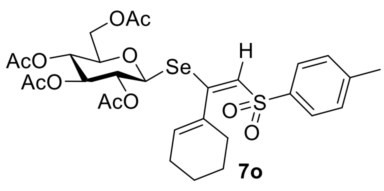

7o

(2*R*,3*R*,4*S*,5*R*,6*S*)-2-(acetoxymethyl)-6-(((*E*)-1-(cyclohex-1-en-1-yl)-2-tosylvinyl)selanyl)tetrahydro-2*H*-pyran-3,4,5-triyl triacetate

7o was synthesized according to general procedure B and isolated by column chromatography on silica gel using petroleum ether/ethyl acetate (3:1 v/v) as the eluent, giving the title product as a white solid (48.8 mg, 73% yield).

^{1}H NMR (400 MHz, CDCl$_3$) δ 7.72 (d, *J* = 8.1 Hz, 2H), 7.31 (d, *J* = 8.0 Hz, 2H), 6.61 (s, 1H), 5.58 (s, 1H), 5.18 (t, *J* = 9.2 Hz, 1H), 5.08 – 5.02 (m, 2H), 4.83 (d, *J* = 10.3 Hz, 1H), 4.18 – 4.13 (m, 1H), 4.08 – 4.05 (m, 1H), 3.62 (m, 1H), 2.43 (s, 3H), 2.13 – 1.96 (m, 16H), 1.62 – 1.55 (m, 4H) ppm. **^{13}C NMR (101 MHz, CDCl$_3$)** δ 170.80, 170.21, 169.53, 169.45, 154.71, 144.29, 139.35, 133.27, 131.23, 129.76, 128.34, 127.90, 81.09, 77.36, 73.73, 69.99, 68.17, 62.29, 28.03, 25.43, 22.22, 21.75, 21.43, 20.80, 20.77, 20.70, 20.68 ppm. **IR (thin film, cm^{-1}):** 2954, 2922, 2852, 2322, 2164, 2050, 1981, 1750, 1568, 1458, 1375, 1316, 1220, 1145, 1084, 1042, 909, 889, 802, 760, 649, 600, 555, 531, 487. **$[\alpha]_D^{25}$** = +16.5 (c = 0.10, CHCl$_3$). **HRMS** (ESI-TOF): calculated for $C_{29}H_{37}O_{11}SSe^+$ [M+H$^+$]: 673.1216, found 673.1208.

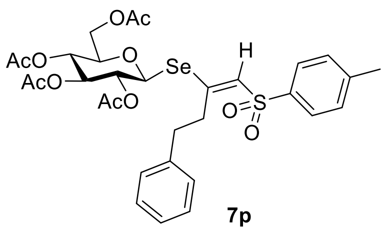

7p

(2*R*,3*R*,4*S*,5*R*,6*S*)-2-(acetoxymethyl)-6-(((*E*)-4-phenyl-1-tosylbut-1-en-2-yl)selanyl)tetrahydro-2*H*-pyran-3,4,5-triyl triacetate

7p was synthesized according to general procedure B (with reaction time 10 min) and isolated by column chromatography on silica gel using petroleum ether/ethyl acetate (3:1 v/v) as the eluent, giving the title product as a white solid (50.1 mg, 72% yield).

^{1}H NMR (400 MHz, CDCl$_3$) δ 7.72 (d, *J* = 8.4, 2H), 7.33 – 7.22 (m, 4H), 7.21 – 7.16 (m, 3H), 6.55 (s, 1H), 5.17 (t, *J* = 9.2 Hz, 1H), 5.07 – 4.89 (m, 3H), 4.14 – 4.08 (m, 1H), 4.04 – 4.00 (m, 1H), 3.69 – 3.65 (m, 1H), 3.22 – 3.12 (m, 1H), 3.06 – 2.97 (m, 1H), 2.89 – 2.75 (m, 2H), 2.41 (s, 3H), 2.05 (s, 3H), 2.01 – 1.95 (m, 9H) ppm. **^{13}C NMR (101 MHz, CDCl$_3$)** δ 170.90, 170.10, 169.48, 169.44, 154.53, 144.61, 140.22, 139.08, 130.21, 128.71, 128.67, 128.15, 127.30, 126.61, 80.65, 77.36, 73.56, 70.13, 67.94, 62.10, 36.62, 35.94, 21.78, 20.79, 20.75, 20.68 ppm. **IR (thin film, cm^{-1}):** 3311, 2955, 2922, 2852, 2322, 2286, 2164, 2110, 2048, 1980, 1751, 1596, 1494, 1457, 1375, 1317, 1221, 1145, 1085, 1035, 909, 801, 701,

644, 599, 552, 528, 486. **[α]$_D^{25}$** = -21.1 (c = 0.09, $CHCl_3$). **HRMS** (ESI-TOF): calculated for $C_{31}H_{37}O_{11}SSe^+$ [M+H$^+$]: 697.1216, found 697.1210.

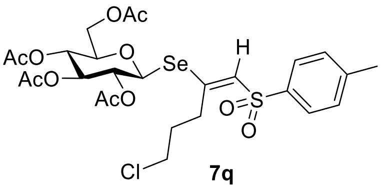

7q

(2*R*,3*R*,4*S*,5*R*,6*S*)-2-(acetoxymethyl)-6-(((*E*)-5-chloro-1-tosylpent-1-en-2-yl)selanyl)tetrahydro-2*H*-pyran-3,4,5-triyl triacetate

7q was synthesized according to general procedure B and isolated by column chromatography on silica gel using petroleum ether/ethyl acetate (3:1 v/v) as the eluent, giving the title product as a white solid (50.1 mg, 75% yield).

^{1}H NMR (400 MHz, $CDCl_3$) δ 7.80 (d, *J* = 8.3 Hz, 2H), 7.37 (d, *J* = 8.2 Hz, 2H), 6.59 (s, 1H), 5.19 (t, *J* = 9.1 Hz, 1H), 5.09 – 4.95 (m, 3H), 4.18 – 4.13 (m, 1H), 4.09 – 4.05 (m, 1H), 3.74 – 3.69 (m, 1H), 3.57 (t, *J* = 6.5 Hz, 2H), 3.07 – 2.88 (m, 2H), 2.45 (s, 3H), 2.11 (s, 3H), 2.03 – 1.98 (m, 11H) ppm. **^{13}C NMR (101 MHz, $CDCl_3$)** δ 170.86, 170.08, 169.48, 169.42, 153.75, 144.80, 138.91, 130.26, 128.59, 127.32, 80.43, 77.36, 73.53, 70.02, 67.89, 62.05, 44.17, 32.51, 31.91, 21.78, 20.82, 20.71, 20.66 ppm. **IR (thin film, cm^{-1}):** 2954, 2922, 2852, 2286, 2166, 2080, 1981, 1751, 1594, 1458, 1375, 1222, 1145, 1085, 1041, 909, 800, 645, 600, 551, 531, 487. **[α]$_D^{25}$** = -12.0 (c = 0.10, $CHCl_3$). **HRMS** (ESI-TOF): calculated for $C_{26}H_{34}ClO_{11}SSe^+$ [M+H$^+$]: 669.0670, found 669.0660.

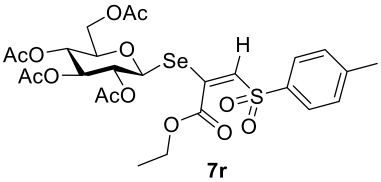

7r

(2*R*,3*R*,4*S*,5*R*,6*S*)-2-(acetoxymethyl)-6-(((*E*)-3-ethoxy-3-oxo-1-tosylprop-1-en-2-yl)selanyl)tetrahydro-2*H*-pyran-3,4,5-triyl triacetate

7r was synthesized according to general procedure B (with reaction time 10 min) and isolated by column chromatography on silica gel using petroleum ether/ethyl acetate (3:1 v/v) as the eluent, giving the title product as a white solid (30.0 mg, 45% yield).

^{1}H NMR (400 MHz, $CDCl_3$) δ 7.83 (d, *J* = 8.3 Hz, 2H), 7.36 (d, *J* = 8.1 Hz, 2H), 6.61 (s, 1H), 5.24 – 5.16 (m, 1H), 5.11 – 5.00 (m, 3H), 4.40 (q, *J* = 7.1 Hz, 2H), 4.20 – 4.16 (m, 1H), 4.06 – 4.02 (m, 1H), 3.69 – 3.65 (m, 1H), 2.45 (s, 3H), 2.10 (s, 3H), 2.03 – 1.98 (m, 9H), 1.42 (t, *J* = 7.1 Hz, 3H) ppm. **^{13}C NMR (101 MHz, $CDCl_3$)** δ 170.81, 170.10, 169.71, 169.45, 164.57, 145.38, 137.21, 136.88, 131.86,

130.23, 128.23, 80.62, 77.48, 73.46, 70.34, 67.96, 63.11, 61.90, 21.85, 20.82, 20.70, 20.67, 14.13 ppm. **IR (thin film, cm^{-1}):** 2954, 2922, 2852, 2322, 2188, 2164, 2050, 1984, 1748, 1595, 1459, 1375, 1328, 1216, 1149, 1085, 1057, 909, 801, 761, 655, 600, 567, 532. **[α]$_D$^{25}** = -4.4 (c = 0.09, $CHCl_3$). **HRMS** (ESI-TOF): calculated for $C_{26}H_{33}O_{13}SSe^+$ [M+H$^+$]: 665.0802, found 665.0795.

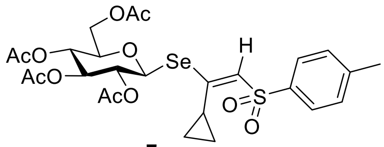

7s

(2*R*,3*R*,4*S*,5*R*,6*S*)-2-(acetoxymethyl)-6-(((*E*)-1-cyclopropyl-2-tosylvinyl)selanyl)tetrahydro-2*H*-pyran-3,4,5-triyl triacetate

7s was synthesized according to general procedure B and isolated by column chromatography on silica gel using petroleum ether/ethyl acetate (3:1 v/v) as the eluent, giving the title product as a white solid (35.1 mg, 56% yield).

^{1}H NMR (400 MHz, CDCl$_3$) δ 7.82 (d, *J* = 8.4 Hz, 2H), 7.36 (d, *J* = 8.0 Hz, 2H), 6.73 (d, *J* = 0.8 Hz, 1H), 5.16 (t, *J* = 9.3 Hz, 1H), 5.04 – 4.94 (m, 2H), 4.79 (d, *J* = 10.3 Hz, 1H), 4.16 – 4.12 (m, 1H), 4.07 – 4.04 (m, 1H), 3.68 – 3.64 (m, 1H), 2.88 – 2.77 (m, 1H), 2.45 (s, 3H), 2.13 (s, 3H), 2.03 – 1.97 (m, 9H), 1.00 – 0.86 (m, 2H), 0.84 – 0.75 (m, 2H) ppm. **^{13}C NMR (101 MHz, CDCl$_3$)** δ 170.95, 170.09, 169.43, 158.32, 144.41, 139.42, 130.08, 129.71, 127.20, 81.64, 77.42, 73.55, 70.22, 67.95, 62.11, 21.77, 20.83, 20.71, 20.66, 14.84, 9.52, 8.85 ppm. **IR (thin film, cm^{-1}):** 2955, 2921, 2852, 2646, 2322, 2050, 2019, 1984, 1751, 1563, 1459, 1375, 1287, 1221, 1147, 1084, 1055, 959, 909, 804, 759, 667, 646, 600, 555, 531. **[α]$_D$^{25}** = -22.2 (c = 0.09, $CHCl_3$). **HRMS** (ESI-TOF): calculated for $C_{26}H_{33}O_{11}SSe^+$ [M+H$^+$]: 633.0903, found 633.0900.

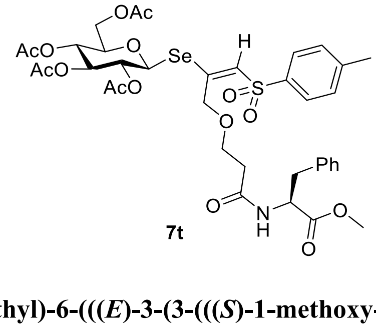

7t

(2*R*,3*R*,4*S*,5*R*,6*S*)-2-(acetoxymethyl)-6-(((*E*)-3-(3-(((*S*)-1-methoxy-1-oxo-3-phenylpropan-2-yl)amino)-3-oxopropoxy)-1-tosylprop-1-en-2-yl)selanyl)tetrahydro-2*H*-pyran-3,4,5-triyl triacetate

A flask was charged with Methyl *L*-phenylalaninate hydrochloride (0.12 mmol, 1.2 equiv.), propargyl-NHS ester (0.10 mmol, 1.0 equiv.), Et_3N (0.15 mmol, 1.5 equiv.) and CH_2Cl_2 (1.0 mL). The reaction mixture was stirred at room temperature for 10 h. Upon completion, the reaction mixture was

concentrated and the resulting residue was subjected directly for the following reaction with a modified procedure B (2.0 equiv. **5a**, solvent: PBS (pH = 9.0) : CH_3CN = 4:1, without addition of Na_2CO_3). **7t** was isolated by column chromatography on silica gel using petroleum ether/ethyl acetate (1:2 v/v) as the eluent, giving the title product as a white solid (1 min: 38.3 mg, 44% yield; 30 min: 61.8 mg, 71%).

^{1}H NMR (400 MHz, $CDCl_3$) δ 7.81 (d, *J* = 7.9 Hz, 2H), 7.38 (d, *J* = 8.0 Hz, 2H), 7.36 – 7.22 (m, 3H), 7.11 (d, *J* = 7.3 Hz, 2H), 6.54 (d, *J* = 1.7 Hz, 1H), 6.30 (d, *J* = 7.8 Hz, 1H), 5.23 (t, *J* = 9.3 Hz, 1H), 5.12 – 4.94 (m, 3H), 4.93 – 4.85 (m, 1H), 4.83 – 4.71 (m, 2H), 4.21 – 4.11 (m, 1H), 4.10 – 4.01 (m, 1H), 3.77 – 3.67 (m, 6H), 3.22 – 3.06 (m, 2H), 2.49 – 2.40 (m, 5H), 2.12 (s, 3H), 2.06 – 1.98 (m, 9H). **^{13}C NMR (101 MHz, $CDCl_3$)** δ 171.98, 170.86, 170.05, 169.46, 169.40, 154.74, 144.85, 138.63, 136.00, 130.28, 129.41, 128.69, 127.24, 127.21, 125.58, 78.95, 77.35, 73.56, 69.89, 69.06, 67.95, 67.51, 62.11, 53.25, 52.41, 37.91, 36.74, 21.77, 20.78, 20.66, 20.64. ppm. **IR (thin film, cm^{-1})**: 3004, 2702, 2599, 2476, 2322, 2019, 1678, 1395, 945, 769, 634. **$[\alpha]_D^{25}$** = +4.4 (c = 0.25, $CHCl_3$). **HRMS** (ESI-TOF): calculated for $C_{37}H_{45}NO_{15}SSeNa^+$ [M+Na$^+$]: 878.1567, found 878.1554.

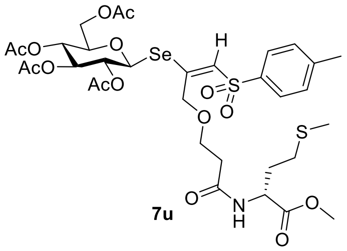

7u

(2*R*,3*R*,4*S*,5*R*,6*S*)-2-(acetoxymethyl)-6-(((*E*)-3-(3-(((*S*)-1-methoxy-3-(methylthio)-1-oxopropan-2-yl)amino)-3-oxopropoxy)-1-tosylprop-1-en-2-yl)selanyl)tetrahydro-2*H*-pyran-3,4,5-triyl triacetate

A flask was charged with methyl *D*-methionine hydrochloride (0.12 mmol, 1.2 equiv.), propargyl-NHS ester (0.10 mmol, 1.0 equiv.), Et_3N (0.15 mmol, 1.5 equiv.) and CH_2Cl_2 (1.0 mL). The reaction mixture was stirred at room temperature for 10 h. Upon completion, the reaction mixture was concentrated and the resulting residue was subjected directly for the following reaction with a modified procedure B (2.0 equiv. **5a**, solvent: PBS (pH = 9.0) : CH_3CN = 4:1, without addition of Na_2CO_3). **7u** was isolated by column chromatography on silica gel using petroleum ether/ethyl acetate (1:2 v/v) as the eluent, giving the title product as a white solid (30 min: 43.6 mg, 52%).

^{1}H NMR (400 MHz, $CDCl_3$) δ 7.78 (d, *J* = 8.0 Hz, 2H), 7.36 (d, *J* = 8.0 Hz, 2H), 6.56 – 6.49 (m, 2H), 5.19 (t, *J* = 9.2 Hz, 1H), 5.06 (t, *J* = 9.7 Hz, 1H), 5.01 – 4.90 (m, 2H), 4.84 – 4.76 (m, 2H), 4.76 – 4.65 (m, 1H), 4.17 – 4.08 (m, 1H), 4.06 – 3.99 (m, 1H), 3.76 – 3.64 (m, 5H), 2.53 – 2.46 (m, 3H), 2.46 – 2.40 (m, 3H), 2.22 – 2.05 (m, 7H), 2.05 – 1.93 (m, 10H), 1.73 – 1.63 (m, 2H) ppm. **^{13}C NMR (101 MHz, $CDCl_3$)** δ 172.45, 170.88, 170.42, 170.05, 169.53, 169.41, 154.40, 144.88, 138.54, 130.30, 127.22, 125.74, 78.94, 77.30, 73.53, 69.85, 69.07, 67.93, 67.51, 62.09, 52.62, 51.67, 36.80, 31.68, 30.07, 21.77,

20.78, 20.71, 20.64, 15.56 ppm. **IR (thin film, cm^{-1})**: 3344, 1742, 1643, 1375, 1222, 1084, 1043. **[α]$_D^{25}$** = -8.1 (c = 0.15, $CHCl_3$). **HRMS** (ESI-TOF): calculated for $C_{33}H_{45}NO_{15}S_2SeNa^+$ [M+Na$^+$]: 862.1288, found 862.1274.

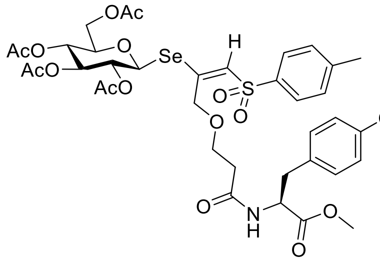

7v

(2*R*,3*R*,4*S*,5*R*,6*S*)-2-(acetoxymethyl)-6-(((*E*)-3-(3-(((S)-3-(4-hydroxyphenyl)-1-methoxy-1-oxopropan-2-yl)amino)-3-oxopropoxy)-1-tosylprop-1-en-2-yl)selanyl)tetrahydro-2*H*-pyran-3,4,5-triyl triacetate

A flask was charged with methyl *L*-tyrosinate hydrochloride (0.12 mmol, 1.2 equiv.), propargyl-NHS ester (0.10 mmol, 1.0 equiv.), Et_3N (0.15 mmol, 1.5 equiv.) and CH_2Cl_2 (1.0 mL). The reaction mixture was stirred at room temperature for 10 h. Upon completion, the reaction mixture was concentrated and the resulting residue was subjected directly for the following reaction with a modified procedure B (2.0 equiv. **5a**, solvent: PBS (pH = 9.0) : CH_3CN = 4:1, without addition of Na_2CO_3). **7v** was isolated by column chromatography on silica gel using petroleum ether/ethyl acetate (1:2 v/v) as the eluent, giving the title product as a white solid (30 min: 40.1 mg, 46%).

^{1}H NMR (400 MHz, $CDCl_3$) δ 7.78 (d, *J* = 8.4 Hz, 2H), 7.35 (d, *J* = 7.9 Hz, 2H), 6.93 – 6.88 (m, 2H), 6.74 – 6.70 (m, 2H), 6.52 – 6.48 (m, 2H), 6.36 (d, *J* = 7.7 Hz, 1H), 5.20 (t, *J* = 9.3 Hz, 1H), 5.11 – 4.97 (m, 2H), 4.92 (d, *J* = 10.2, 1.7 Hz, 1H), 4.87 – 4.79 (m, 1H), 4.78 – 4.65 (m, 2H), 4.22 – 4.03 (m, 3H), 3.78 – 3.68 (m, 3H), 3.68 – 3.56 (m, 2H), 3.08 (dd, *J* = 14.1, 5.4 Hz, 1H), 2.99 (dd, *J* = 14.1, 5.8 Hz, 1H), 2.45 – 2.38 (m, 5H), 2.10 (s, 3H), 2.05 – 1.95 (m, 9H) ppm. **^{13}C NMR (101 MHz, $CDCl_3$)** δ 172.08, 171.13, 170.38, 170.18, 169.80, 169.46, 155.49, 154.84, 145.02, 138.38, 130.52, 130.35, 127.30, 127.22, 125.23, 115.78, 78.93, 77.36, 73.61, 70.00, 69.11, 67.90, 67.69, 62.00, 53.31, 52.48, 36.85, 36.81, 21.79, 20.81, 20.72, 20.67 ppm. **IR (thin film, cm^{-1})**: 2920, 1748, 1653, 1439, 1370, 1222, 1143, 1049. **[α]$_D^{25}$** = -3.2 (c = 0.19, $CHCl_3$). **HRMS** (ESI-TOF): calculated for $C_{37}H_{45}NO_{16}SSeNa^+$ [M+Na$^+$]: 894.1516, found 894.1510.

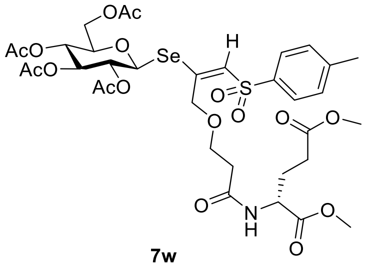

7w

dimethyl (3-(((*E*)-3-tosyl-2-(((2*S*,3*R*,4*S*,5*R*,6*R*)-3,4,5-triacetoxy-6-(acetoxymethyl)tetrahydro-2*H*-

pyran-2-yl)selanyl)allyl)oxy)propanoyl)-*D*-glutamate

A flask was charged with dimethyl *D*- glutamic hydrochloride (0.12 mmol, 1.2 equiv.), propargyl-NHS ester (0.10 mmol, 1.0 equiv.), Et_3N (0.15 mmol, 1.5 equiv.) and CH_2Cl_2 (1.0 mL). The reaction mixture was stirred at room temperature for 10 h. Upon completion, the reaction mixture was concentrated and the resulting residue was subjected directly for the following reaction with a modified procedure B (2.0 equiv. **5a**, solvent: PBS (pH = 9.0) : CH_3CN = 4:1, without addition of Na_2CO_3). **7w** was isolated by column chromatography on silica gel using petroleum ether/ethyl acetate (1:2 v/v) as the eluent, giving the title product as a white solid (30 min: 59.6 mg, 70%).

^{1}H NMR (400 MHz, CDCl$_3$) δ 7.77 (d, *J* = 8.0 Hz, 2H), 7.35 (d, *J* = 8.0 Hz, 2H), 6.55 (d, *J* = 7.8 Hz, 1H), 6.49 (s, 1H), 5.19 (t, *J* = 9.2 Hz, 1H), 5.05 (t, *J* = 9.7 Hz, 1H), 5.01 – 4.90 (m, 2H), 4.83 – 4.72 (m, 2H), 4.64 – 4.54 (m, 1H), 4.16 – 4.05 (m, 1H), 4.05 – 3.98 (m, 1H), 3.76 – 3.61 (m, 9H), 2.50 – 2.33 (m, 7H), 2.08 (s, 3H), 2.03 – 1.94 (m, 11H) ppm. **^{13}C NMR (101 MHz, CDCl$_3$)** δ 173.36, 172.30, 170.84, 170.47, 170.01, 169.51, 169.39, 154.57, 144.83, 138.54, 130.26, 127.18, 125.54, 78.86, 77.24, 73.50, 69.80, 69.03, 67.90, 67.44, 62.07, 52.58, 51.92, 51.72, 36.70, 30.13, 27.32, 21.73, 20.75, 20.65, 20.61, 14.27 ppm. **IR (thin film, cm^{-1})**: 3365, 2976, 2919, 1735, 1644, 1379, 1222, 1083, 1043, 876. **$[\alpha]_D^{25}$** = +8.8 (c = 0.16, $CHCl_3$). **HRMS** (ESI-TOF): calculated for $C_{34}H_{45}NO_{17}SSeNa^+$ [M+Na$^+$]: 874.1466, found 874.1453.

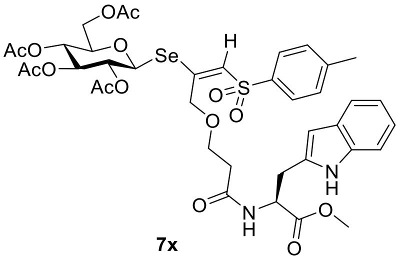

7x

(2*S*,3*R*,4*S*,5*R*,6*R*)-2-(((*E*)-3-(3-(((*S*)-3-(1*H*-indol-2-yl)-1-methoxy-1-oxopropan-2-yl)amino)-3-oxopropoxy)-1-tosylprop-1-en-2-yl)selanyl)-6-(acetoxymethyl)tetrahydro-2*H*-pyran-3,4,5-triyl triacetate

A flask was charged with methyl *L*-tryptophanate hydrochloride (0.12 mmol, 1.2 equiv.), propargyl-NHS ester (0.10 mmol, 1.0 equiv.), Et_3N (0.15 mmol, 1.5 equiv.) and CH_2Cl_2 (1.0 mL). The reaction mixture was stirred at room temperature for 10 h. Upon completion, the reaction mixture was concentrated and the resulting residue was subjected directly for the following reaction with a modified procedure B (2.0 equiv. **5a**, solvent: PBS (pH = 9.0) : CH_3CN = 4:1, without addition of Na_2CO_3). **7x** was isolated by column chromatography on silica gel using petroleum ether/ethyl acetate (1:2 v/v) as the eluent, giving the title product as a white solid (30 min: 55.4 mg, 62%).

^{1}H NMR (400 MHz, CDCl$_3$) δ 8.54 (s, 1H), 7.76 (d, *J* = 8.0 Hz, 2H), 7.52 (d, *J* = 7.9 Hz, 1H), 7.37 – 7.30 (m, 3H), 7.17 (t, *J* = 7.6 Hz, 1H), 7.10 (t, *J* = 7.4 Hz, 1H), 7.01 (d, *J* = 2.4 Hz, 1H), 6.47 (s, 1H), 6.41 (d, *J* = 7.7 Hz, 1H), 5.19 (t, *J* = 9.2 Hz, 1H), 5.08 – 4.95 (m, 2H), 4.95 – 4.82 (m, 2H), 4.73 – 4.60 (m, 2H), 4.24 – 4.09 (m, 1H), 4.08 – 3.99 (m, 1H), 3.69 (s, 3H), 3.67 – 3.57 (m, 3H), 3.32 (d, *J* = 5.4 Hz, 2H), 2.45 – 2.36 (m, 5H), 2.09 (s, 3H), 2.06 – 1.96 (m, 9H) ppm. **^{13}C NMR (101 MHz, CDCl$_3$)** δ 172.39,

170.89, 170.25, 170.09, 169.68, 169.43, 154.59, 144.96, 138.44, 136.24, 130.31, 127.73, 127.18, 125.29, 123.21, 122.24, 119.66, 118.66, 111.51, 109.82, 78.87, 77.35, 73.54, 69.90, 69.01, 67.90, 67.62, 62.02, 60.53, 52.83, 52.50, 36.82, 27.48, 21.76, 20.78, 20.70, 20.66, 14.31 ppm. **IR (thin film, cm^{-1})**: 3356, 2923, 1744, 1645, 1436, 1370, 1222, 1085, 538. **[α]$_D$^{25}** = -9.3 (c = 0.14, $CHCl_3$). **HRMS** (ESI-TOF): calculated for $C_{39}H_{46}N_2O_{15}SSeNa^+$ [M+Na$^+$]: 917.1676, found 917.1671.

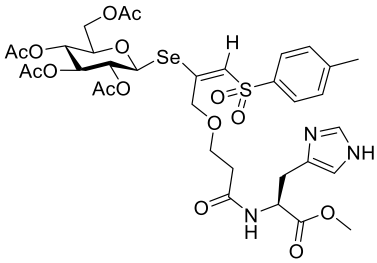

7y

(2*S*,3*R*,4*S*,5*R*,6*R*)-2-(((*E*)-3-(3-(((*S*)-3-(1*H*-imidazol-4-yl)-1-methoxy-1-oxopropan-2-yl)amino)-3-oxopropoxy)-1-tosylprop-1-en-2-yl)selanyl)-6-(acetoxymethyl)tetrahydro-2*H*-pyran-3,4,5-triyl triacetate

A flask was charged with methyl *L*- histidinate dihydrochloride (0.12 mmol, 1.2 equiv.), propargyl-NHS ester (0.10 mmol, 1.0 equiv.), Et_3N (0.15 mmol, 1.5 equiv.) and CH_2Cl_2 (1.0 mL). The reaction mixture was stirred at room temperature for 10 h. Upon completion, the reaction mixture was concentrated and the resulting residue was subjected directly for the following reaction with a modified procedure B (2.0 equiv. **5a**, solvent: PBS (pH = 9.0) : CH_3CN = 4:1, without addition of Na_2CO_3). **7y** was isolated by column chromatography on silica gel using petroleum ether/ethyl acetate (1:2 v/v) as the eluent, giving the title product as a white solid (30 min: 49.0 mg, 58%).

^{1}H NMR (400 MHz, $CDCl_3$) δ 7.77 (d, *J* = 8.0 Hz, 2H), 7.54 (s, 1H), 7.35 (d, *J* = 8.0 Hz, 2H), 7.14 (d, *J* = 7.5 Hz, 1H), 6.79 (s, 1H), 6.49 (s, 1H), 5.19 (t, *J* = 9.2 Hz, 1H), 5.04 (t, *J* = 9.6 Hz, 1H), 5.00 – 4.90 (m, 2H), 4.81 – 4.68 (m, 2H), 4.17 – 3.99 (m, 3H), 3.75 – 3.66 (m, 6H), 3.11 (d, *J* = 5.3 Hz, 2H), 2.50 – 2.41 (m, 5H), 2.08 (s, 3H), 2.04 – 1.95 (m, 10H) ppm. **^{13}C NMR (101 MHz, $CDCl_3$)** δ 171.99, 170.89, 170.45, 170.04, 169.63, 169.39, 154.73, 144.94, 138.44, 135.34, 130.32, 127.18, 125.38, 78.85, 77.30, 73.53, 69.83, 69.10, 67.92, 67.72, 62.04, 60.50, 52.54, 52.50, 36.72, 29.27, 21.75, 20.77, 20.68, 20.62, 14.28 ppm. **IR (thin film, cm^{-1})**: 2920, 2851, 1747, 1656, 1536, 1435, 1371, 1223, 1143, 1084, 1041, 800, 535. **[α]$_D$^{25}** = +4.3 (c = 0.14, $CHCl_3$). **HRMS** (ESI-TOF): calculated for $C_{34}H_{43}N_3O_{15}SSeNa^+$ [M+Na$^+$]: 868.1472, found 868.1445.

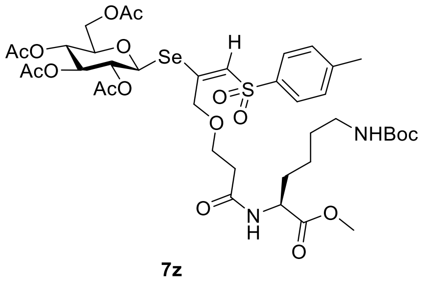

7z

(2*R*,3*R*,4*S*,5*R*,6*S*)-2-(acetoxymethyl)-6-(((*S*,*E*)-10-(methoxycarbonyl)-2,2-dimethyl-4,12-dioxo-18-tosyl-3,15-dioxa-5,11-diazaoctadec-17-en-17-yl)selanyl)tetrahydro-2*H*-pyran-3,4,5-triyl triacetate

A flask was charged with *N*-Boc-*L*-lysine methyl ester hydrochloride (0.12 mmol, 1.2 equiv.), propargyl-NHS ester (0.10 mmol, 1.0 equiv.), Et_3N (0.15 mmol, 1.5 equiv.) and CH_2Cl_2 (1.0 mL). The reaction mixture was stirred at room temperature for 10 h. Upon completion, the reaction mixture was concentrated and the resulting residue was subjected directly for the following reaction with a modified procedure B (2.0 equiv. **5a**, solvent: PBS (pH = 9.0) : CH_3CN = 4:1, without addition of Na_2CO_3). **7z** was isolated by column chromatography on silica gel using petroleum ether/ethyl acetate (1:2 v/v) as the eluent, giving the title product as a white solid (30 min: 63.6 mg, 68%).

^{1}H NMR (400 MHz, $CDCl_3$) δ 7.78 (d, *J* = 8.0 Hz, 2H), 7.37 (d, *J* = 8.0 Hz, 2H), 6.51 (s, 1H), 6.41 (d, *J* = 8.0 Hz, 1H), 5.21 (t, *J* = 9.2 Hz, 1H), 5.09 – 4.93 (m, 3H), 4.78 (s, 2H), 4.70 – 4.53 (m, 2H), 4.19 – 3.99 (m, 2H), 3.82 – 3.65 (m, 6H), 3.07 (d, *J* = 6.8 Hz, 2H), 2.51 – 2.42 (m, 5H), 2.10 (s, 3H), 2.04 – 1.98 (m, 9H), 1.88 – 1.77 (m, 2H), 1.75 – 1.56 (m, 2H), 1.52 – 1.38 (m, 11H) ppm. **^{13}C NMR (101 MHz, $CDCl_3$)** δ 172.92, 170.88, 170.37, 170.07, 169.54, 169.42, 156.20, 154.54, 144.88, 138.58, 130.31, 127.23, 125.62, 78.94, 77.32, 73.56, 69.90, 69.08, 67.96, 67.58, 62.10, 52.49, 52.12, 40.23, 36.79, 32.11, 29.68, 28.55, 22.55, 21.78, 20.80, 20.72, 20.65 ppm. **IR (thin film, cm^{-1})**: 3348, 2975, 2921, 1646, 1381, 1261, 1084, 1043, 877. **$[\alpha]_D^{25}$** = -9.3 (c = 0.26, $CHCl_3$). **HRMS** (ESI-TOF): calculated for $C_{39}H_{56}N_2O_{17}SSeNa^+$ [M+Na$^+$]: 959.2353, found 959.2352.

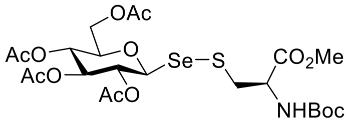

9a

(2*R*,3*R*,4*S*,5*R*,6*S*)-2-(acetoxymethyl)-6-((((*R*)-2-((tert-butoxycarbonyl)amino)-3-methoxy-3-oxopropyl)thio)selanyl)tetrahydro-2*H*-pyran-3,4,5-triyl triacetate

9a was synthesized according to general procedure C and isolated by column chromatography on silica gel using petroleum ether/ethyl acetate (1:1 v/v) as the eluent, giving the title product as a white solid (61.2 mg, 95% yield).

^{1}H NMR (400 MHz, $CDCl_3$) δ 5.33 (d, *J* = 8.5 Hz, 1H), 5.26 – 5.18 (m, 2H), 5.11 (t, *J* = 9.1 Hz, 1H), 4.90 (d, *J* = 8.1 Hz, 1H), 4.61 (d, *J* = 6.6 Hz, 1H), 4.29 – 4.20 (m, 1H), 4.17 – 4.06 (m, 1H), 3.80 – 3.72 (m, 4H), 3.39 – 3.31 (m, 1H), 3.20 – 3.10 (m, 1H), 2.07 – 2.03 (m, 3H), 2.02 – 1.99 (m, 6H), 1.98 (s,

3H), 1.42 (s, 9H). **^{13}C NMR (101 MHz, $CDCl_3$)** δ 171.41, 170.61, 170.20, 169.43, 169.32, 155.10, 83.30, 80.28, 77.20, 73.79, 70.14, 68.06, 62.10, 53.94, 52.61, 41.28, 28.41, 20.73, 20.69, 20.65, 20.62 ppm. **IR (thin film, cm^{-1})**: 2958, 2927, 2829, 2545, 2112, 1986, 1845, 1500, 1210, 980, 785, 664, 585. **$[\alpha]_D^{25}$** = -40.3 (c = 0.37, $CHCl_3$). **HRMS** (ESI-TOF): calculated for $C_{23}H_{35}NO_{13}SSeNa^+$ [M+Na$^+$]: 668.0887, found 668.0870.

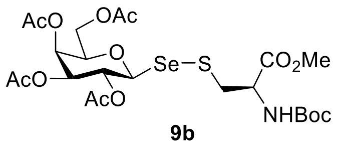

9b

(2*R*,3*S*,4*S*,5*R*,6*S*)-2-(acetoxymethyl)-6-((((*R*)-2-((tert-butoxycarbonyl)amino)-3-methoxy-3-oxopropyl)thio)selanyl)tetrahydro-2*H*-pyran-3,4,5-triyl triacetate

9b was synthesized according to general procedure C and isolated by column chromatography on silica gel using petroleum ether/ethyl acetate (1:1 v/v) as the eluent, giving the title product as a white solid (56.7 mg, 88% yield).

^{1}H NMR (400 MHz, $CDCl_3$) δ 5.46 – 5.31 (m, 3H), 5.10 – 5.03 (m, 1H), 4.96 (d, *J* = 10.1 Hz, 1H), 4.64 (s, 1H), 4.13 (d, *J* = 6.5 Hz, 2H), 4.04 (d, *J* = 6.5 Hz, 1H), 3.74 (s, 3H), 3.40 – 3.31 (m, 1H), 3.25 – 3.16 (m, 1H), 2.14 (s, 3H), 2.03 (s, 3H), 2.02 (s, 3H), 1.96 (s, 3H), 1.43 (s, 9H). **^{13}C NMR (101 MHz, $CDCl_3$)** δ 171.46, 170.53, 170.32, 170.16, 169.82, 155.20, 85.84, 80.34, 75.93, 71.59, 67.65, 67.42, 61.63, 54.01, 52.70, 41.31, 28.42, 20.93, 20.78, 20.76, 20.71 ppm. **IR (thin film, cm^{-1})**: 2945, 2874, 1821, 2504, 2112, 1986, 1750, 1592, 1121, 964, 825, 462. **$[\alpha]_D^{25}$** = -11.7 (c = 0.17, $CHCl_3$). **HRMS** (ESI-TOF): calculated for $C_{23}H_{35}NO_{13}SSeNa^+$ [M+Na$^+$]: 668.0887, found 668.0863.

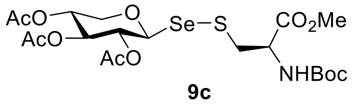

9c

(2*S*,3*R*,4*S*,5*R*)-2-((((*R*)-2-((tert-butoxycarbonyl)amino)-3-methoxy-3-oxopropyl)thio)selanyl)tetrahydro-2*H*-pyran-3,4,5-triyl triacetate

9c was synthesized according to general procedure C and isolated by column chromatography on silica gel using petroleum ether/ethyl acetate (1:1 v/v) as the eluent, giving the title product as a white solid (46.9 mg, 82% yield).

^{1}H NMR (400 MHz, $CDCl_3$) δ 5.46 (d, *J* = 8.5 Hz, 1H), 5.19 – 5.09 (m, 3H), 4.97 – 4.88 (m, 1H), 4.59 (s, 1H), 4.30 – 4.21 (m, 1H), 3.76 (s, 3H), 3.58 – 3.49 (m, 1H), 3.36 – 3.21 (m, 2H), 2.05 (s, 9H, *3OAc*), 1.43 (s, 9H). **^{13}C NMR (101 MHz, $CDCl_3$)** δ 171.32, 169.83, 169.61, 169.47, 155.23, 85.23, 80.30, 70.89, 69.88, 68.10, 65.86, 54.07, 52.64, 41.51, 28.43, 20.81, 20.78 ppm. **IR (thin film, cm^{-1})**: 2954, 2851, 2321, 2257, 2049, 1751, 1460, 1221, 1058, 896, 651, 488. **$[\alpha]_D^{25}$** = -42.7 (c = 0.59, $CHCl_3$). **HRMS** (ESI-

TOF): calculated for $C_{20}H_{31}NO_{11}SSeNa^+$ [M+Na$^+$]: 596.0675, found 596.0657.

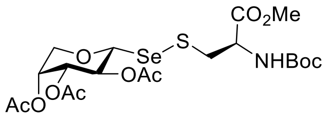

9d

(2*R*,3*S*,4*R*,5*R*)-2-((((*R*)-2-((tert-butoxycarbonyl)amino)-3-methoxy-3-oxopropyl)thio)selanyl)tetrahydro-2*H*-pyran-3,4,5-triyl triacetate

9d was synthesized according to general procedure C with a modification using 1.5 equivalent of **5d** and isolated by column chromatography on silica gel using petroleum ether/ethyl acetate (1:1 v/v) as the eluent, giving the title product as a white solid (56.8 mg, 99% yield).

^{1}H NMR (400 MHz, CDCl$_3$) δ 5.54 – 5.48 (m, 1H), 5.36 – 5.09 (m, 4H), 4.57 (d, *J* = 6.5 Hz, 1H), 4.17 – 4.08 (m, 1H), 3.78 – 3.71 (m, 4H), 3.32 (t, *J* = 5.1 Hz, 2H), 2.11 (s, 3H), 2.08 (s, 3H), 2.07 (s, 3H), 1.43 (s, 9H). **^{13}C NMR (101 MHz, CDCl$_3$)** δ 171.24, 170.14, 169.54, 169.46, 155.24, 85.91, 80.32, 69.38, 69.12, 66.76, 64.53, 54.07, 52.63, 41.27, 28.44, 20.90, 20.80 ppm. **IR (thin film, cm^{-1})**: 2954, 2645, 2285, 1984, 1748, 1594, 1328, 1149, 908, 846, 547, 458. **[α]$_D^{25}$** = +46.4 (c = 0.50, CHCl$_3$). **HRMS** (ESI-TOF): calculated for $C_{20}H_{31}NO_{11}SSeNa^+$ [M+Na$^+$]: 596.0675, found 596.0662.

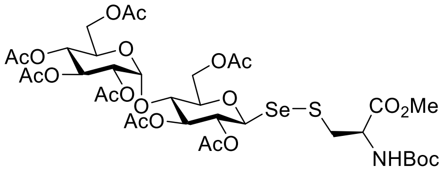

9e

(2*R*,3*R*,4*S*,5*R*,6*R*)-2-(acetoxymethyl)-6-(((2*R*,3*R*,4*S*,5*R*,6*S*)-4,5-diacetoxy-2-(acetoxymethyl)-6-((((*R*)-2-((tert-butoxycarbonyl)amino)-3-methoxy-3-oxopropyl)thio)selanyl)tetrahydro-2*H*-pyran-3-yl)oxy)tetrahydro-2*H*-pyran-3,4,5-triyl triacetate

9e was synthesized according to general procedure C and isolated by column chromatography on silica gel using petroleum ether/ethyl acetate (2:3 v/v) as the eluent, giving the title product as a white solid (78.4 mg, 84% yield).

^{1}H NMR (400 MHz, CDCl$_3$) δ 5.41 – 5.21 (m, 4H), 5.11 – 4.96 (m, 2H), 4.89 (d, *J* = 10.1 Hz, 1H), 4.84 – 4.77 (m, 1H), 4.54 (s, 1H), 4.48 – 4.41 (m, 1H), 4.23 – 4.15 (m, 2H), 4.04 – 3.96 (m, 2H), 3.93 – 3.86 (m, 1H), 3.72 – 3.70 (m, 4H), 3.34 – 3.26 (m, 1H), 3.10 (s, 1H), 2.12 – 1.91 (m, 21H), 1.39 (s, 9H). **^{13}C NMR (101 MHz, CDCl$_3$)** δ 171.23, 170.48, 170.43, 170.34, 170.10, 169.84, 169.46, 169.36, 154.99, 95.69, 82.31, 80.15, 77.28, 76.13, 72.44, 70.66, 69.99, 69.31, 68.53, 67.99, 62.81, 61.41, 53.77, 52.50, 40.93, 28.27, 20.86, 20.65, 20.59, 20.52 ppm. **IR (thin film, cm^{-1})**: 2954, 2852, 2456, 2357, 2050, 1980, 1372, 1120, 802, 697, 477. **[α]$_D^{25}$** = - 54.6(c = 0.24, CHCl$_3$). **HRMS** (ESI-TOF): calculated for

$C_{35}H_{51}NO_{21}SSeNa^+$ [M+Na$^+$]: 956.1732, found 956.1716.

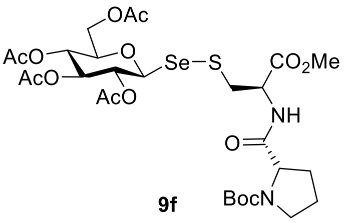

9f

(2*R*,3*R*,4*S*,5*R*,6*S*)-2-(acetoxymethyl)-6-((((*R*)-2-((S)-1-(tert-butoxycarbonyl)pyrrolidine-2-carboxamido)-3-methoxy-3-oxopropyl)thio)selanyl)tetrahydro-2*H*-pyran-3,4,5-triyl triacetate

9f was synthesized according to general procedure C and isolated by column chromatography on silica gel using petroleum ether/ethyl acetate (1:2 v/v) as the eluent, giving the title product as a white solid (69.7 mg, 94% yield).

^{1}H NMR (400 MHz, CDCl$_3$) δ 5.22 (d, *J* = 7.4 Hz, 2H), 5.09 (t, *J* = 9.0 Hz, 1H), 4.89 (d, *J* = 8.1 Hz, 2H), 4.36 – 4.18 (m, 2H), 4.13 (d, *J* = 12.3 Hz, 1H), 3.82 – 3.75 (m, 1H), 3.73 – 3.72 (m, 3H), 3.50 – 3.29 (m, 3H), 3.19 – 3.09 (m, 1H), 2.22 – 2.06 (m, 1H), 2.07 – 1.95 (m, 13H), 1.92 – 1.80 (m, 3H), 1.44 (s, 9H). **^{13}C NMR (101 MHz, CDCl$_3$)** δ 172.23, 170.74, 170.60, 170.17, 169.44, 169.31, 83.36, 80.75, 77.05, 73.73, 70.15, 68.07, 62.07, 52.65, 52.37, 47.15, 41.12, 28.42, 24.48, 22.38, 20.71, 20.64, 20.61 ppm. **IR (thin film, cm^{-1})**: 2956, 2924, 2049, 1752, 1492, 1458, 1376, 1214, 1093, 910, 800, 597, 528. **$[\alpha]_D^{25}$** = -69.1 (c = 0.54, CHCl$_3$). **HRMS** (ESI-TOF): calculated for $C_{28}H_{42}N_2O_{14}SSeNa^+$ [M+Na$^+$]: 765.1414, found 765.1395.

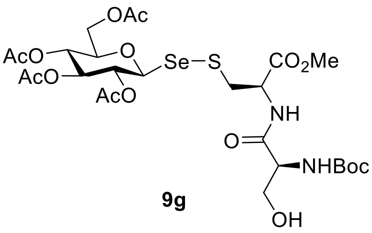

9g

(2*R*,3*R*,4*S*,5*R*,6*S*)-2-(acetoxymethyl)-6-((((*R*)-2-((S)-2-((tert-butoxycarbonyl)amino)-3-hydroxypropanamido)-3-methoxy-3-oxopropyl)thio)selanyl)tetrahydro-2*H*-pyran-3,4,5-triyl triacetate

9g was synthesized according to general procedure C and isolated by column chromatography on silica gel using petroleum ether/ethyl acetate (1:1 v/v) as the eluent, giving the title product as a white solid (51.9 mg, 71% yield).

^{1}H NMR (400 MHz, CDCl$_3$) δ 7.32 (s, 1H), 5.57 (d, *J* = 7.6 Hz, 1H), 5.30 – 5.16 (m, 2H), 5.13 – 5.11 (m, 1H), 4.96 – 4.84 (m, 2H), 4.27 – 4.10 (m, 3H), 4.03 (d, *J* = 11.3 Hz, 1H), 3.83 – 3.72 (m, 4H), 3.69 – 3.60 (m, 1H), 3.43 – 3.33 (m, 1H), 3.27 – 3.10 (m, 2H), 2.08 – 1.96 (m, 12H), 1.43 (s, 9H). **^{13}C NMR**

(101 MHz, CDCl3) δ 171.30, 170.89, 170.74, 170.23, 169.48, 169.41, 156.04, 82.97, 80.64, 77.08, 73.73, 70.08, 68.10, 62.96, 62.13, 55.34, 52.91, 52.88, 40.12, 28.41, 20.74, 20.65, 20.63 ppm. **IR (thin film, cm^{-1})**: 2953, 2923, 2851, 2323, 1652, 1395, 1238, 1041, 901, 767, 731, 643. **[α]$_D$^{25}** = -105.9 (c = 0.29, $CHCl_3$). **HRMS** (ESI-TOF): calculated for $C_{26}H_{40}N_2O_{15}SSeNa^+$ [M+Na$^+$]: 755.1207, found 755.1194.

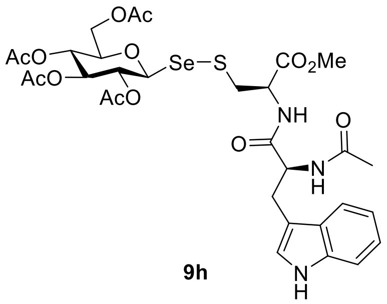

9h

(2*S*,3*R*,4*S*,5*R*,6*R*)-2-((((*R*)-2-((*S*)-2-acetamido-3-(1H-indol-3-yl)propanamido)-3-methoxy-3-oxopropyl)thio)selanyl)-6-(acetoxymethyl)tetrahydro-2*H*-pyran-3,4,5-triyl triacetate

9h was synthesized according to general procedure C and isolated by column chromatography on silica gel using petroleum ether/ethyl acetate (1:2 v/v) as the eluent, giving the title product as a white solid (71.9 mg, 93% yield).

^{1}H NMR (400 MHz, CDCl3) δ 8.47 (s, 1H), 7.74 (d, *J* = 7.8 Hz, 1H), 7.35 (d, *J* = 8.0 Hz, 1H), 7.18 (t, *J* = 7.5 Hz, 1H), 7.15 – 7.08 (m, 2H), 6.62 (d, *J* = 7.9 Hz, 1H), 6.40 (d, *J* = 7.5 Hz, 1H), 5.25 – 5.16 (m, 2H), 5.13 – 5.04 (m, 1H), 4.85 – 4.72 (m, 2H), 4.72 – 4.62 (m, 1H), 4.23 – 4.06 (m, 2H), 3.69 – 3.64 (m, 4H), 3.37 – 3.28 (m, 1H), 3.28 – 3.19 (m, 1H), 3.19 – 3.03 (m, 2H), 2.08 – 1.93 (m, 15H). **^{13}C NMR (101 MHz, CDCl3)** δ 171.28, 170.73, 170.39, 170.30, 169.54, 169.48, 136.46, 127.73, 123.89, 122.33, 119.87, 119.01, 111.47, 110.56, 82.94, 76.94, 73.70, 70.02, 68.12, 62.06, 53.93, 53.09, 52.71, 40.03, 28.50, 23.35, 20.79, 20.72, 20.69, 20.66 ppm. **IR (thin film, cm^{-1})**: 2954, 2853, 2649, 2285, 1746, 1518, 1366, 1213, 908, 711, 468. **[α]$_D$^{25}** = -78.0 (c = 0.61, $CHCl_3$). **HRMS** (ESI-TOF): calculated for $C_{31}H_{39}N_3O_{13}SSeNa^+$ [M+Na$^+$]: 796.1261, found 796.1238.

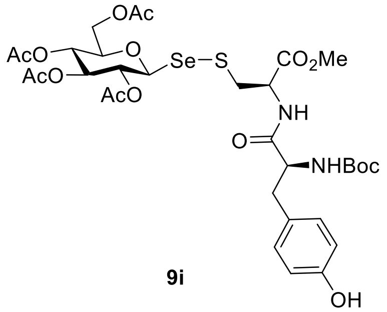

9i

(2*R*,3*R*,4*S*,5*R*,6*S*)-2-(acetoxymethyl)-6-((((*R*)-2-((S)-2-((tert-butoxycarbonyl)amino)-3-(4-hydroxyphenyl)propanamido)-3-methoxy-3-oxopropyl)thio)selanyl)tetrahydro-2*H*-pyran-3,4,5-

triyl triacetate

9i was synthesized according to general procedure C and isolated by column chromatography on silica gel using petroleum ether/ethyl acetate (1:2 v/v) as the eluent, giving the title product as a white solid (66.2 mg, 82% yield).

^{1}H NMR (400 MHz, $CDCl_3$) δ 7.01 (d, J = 8.5 Hz, 2H), 6.92 (s, 1H), 6.79 (d, J = 7.9 Hz, 1H), 6.73 (d, J = 8.5 Hz, 2H), 5.30 – 5.19 (m, 2H), 5.17 – 5.03 (m, 2H), 4.93 – 4.84 (m, 1H), 4.82 (d, J = 6.5 Hz, 1H), 4.31 (s, 1H), 4.27 – 4.20 (m, 1H), 4.15 – 4.09 (m, 1H), 3.81 – 3.74 (m, 1H), 3.71 (s, 3H), 3.33 – 3.29 (m, 1H), 3.12 – 3.08 (m, 1H), 2.97 – 2.96 (m, 2H), 2.04 (s, 3H), 2.01 (s, 6H), 1.99 (s, 3H), 1.40 (s, 9H). **^{13}C NMR (101 MHz, $CDCl_3$)** δ 171.54, 170.92, 170.60, 170.39, 169.67, 169.63, 155.57, 155.44, 130.59, 127.77, 115.78, 82.93, 80.52, 76.91, 73.68, 70.00, 68.04, 62.10, 55.98, 52.80, 52.76, 40.38, 37.47, 28.37, 20.78, 20.76, 20.70, 20.67 ppm. **IR (thin film, cm^{-1})**: 2954, 2852, 2323, 2283, 1697, 1617, 1375, 909, 833, 732, 645, 479. **$[\alpha]_D^{25}$** = -60.4 (c = 0.23, $CHCl_3$). **HRMS** (ESI-TOF): calculated for $C_{32}H_{44}N_2O_{15}SSeNa^+$ [M+Na$^+$]: 831.1520, found 831.1501.

10. X-Ray for compound 5a

To a 20 mL glass bottle, was put a 3 mL tube which contains a solution of **5a** (15 mg of pure **5a** was completely dissolved in 0.5 mL CH_2Cl_2). Then 8 mL of petroleum ether was added into the 20 mL glass bottle. Upon completion, the 20 mL glass bottle was sealed and placed at 5 °C. Two days later, the crystal was collected.

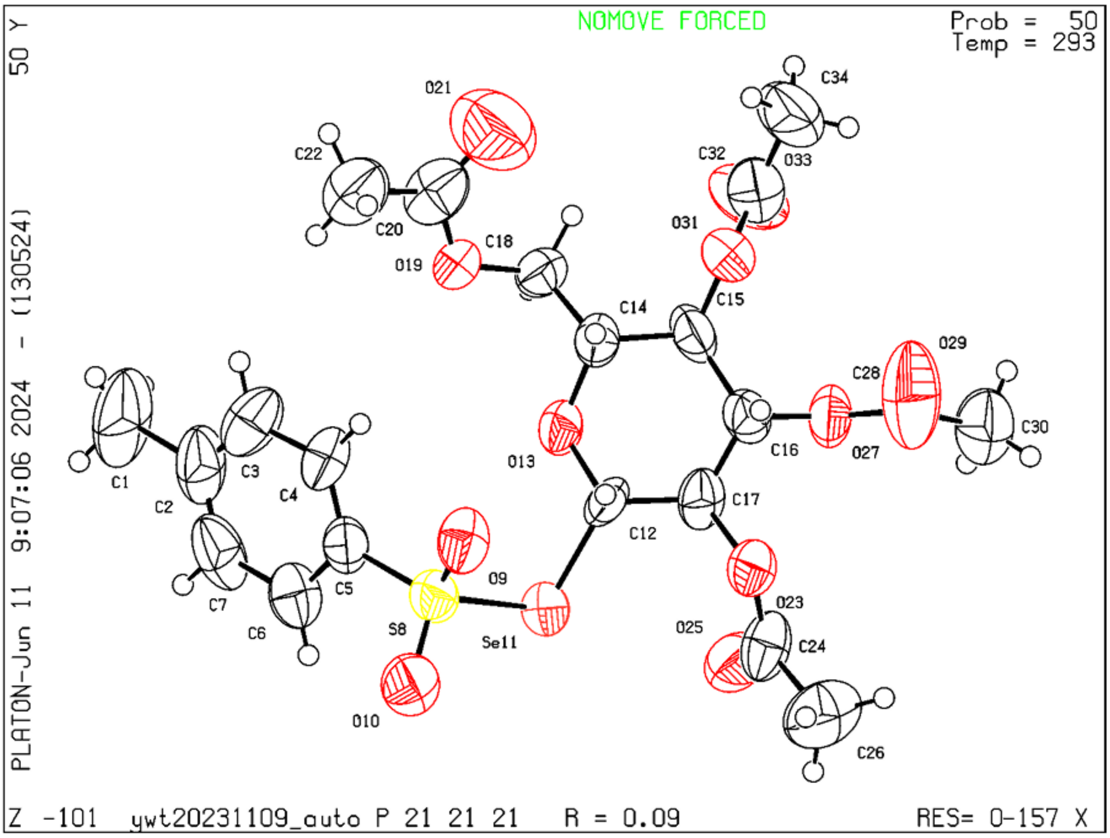

Figure S17. ORTEP diagram of **5a** with 50% probability ellipsoids.

Table S12. Crystal data for **5a**.

Bond precision:	C-C = 0.0312 A		Wavelength=1.54184
Cell:	a=5.7455(4)	b=14.8009(8)	c=31.2759(16)
	alpha=90	beta=90	gamma=90
Temperature:	293 K		

	Calculated	Reported
Volume	2659.7(3)	2659.7(3)
Space group	P 21 21 21	P 21 21 21
Hall group	P 2ac 2ab	P 2ac 2ab
Moiety formula	C21 H26 O11 S Se	C21 H26 O11 S Se
Sum formula	C21 H26 O11 S Se	C21 H26 O11 S Se
Mr	565.44	565.44
Dx,g cm-3	1.412	1.412
Z	4	4
Mu (mm-1)	3.116	3.116
F000	1160.0	1160.0
F000’	1161.73	
h,k,lmax	6,17,36	6,17,36
Nref	4534[2646]	4237
Tmin,Tmax	0.894,0.940	0.026,1.000
Tmin’	0.779	

Correction method= # Reported T Limits: Tmin=0.026 Tmax=1.000
AbsCorr = MULTI-SCAN

Data completeness= 1.60/0.93 Theta(max)= 65.073

R(reflections)= 0.0926(2073) wR2(reflections)= 0.3424(4237)

S = 1.021 Npar= 312

11. References

[1] Kawai, Y.; Ando, H.; Ozeki, H.; Koketsu, M.; Ishihara, H. A Facile Method for β-Selenoglycoside Synthesis Using β-*p*-Methylbenzoyl Selenoglycoside as the Selenating Unit. *Org. Lett.* **2005**, *7*, 4653-4656.

[2] Lin, Y.; Liu, G.; Yi, W. Stereoselective Synthesis of Alkenyl Silanes, Sulfones, Phosphine Oxides, and Nitroolefins by Radical C–S Bond Cleavage of Arylalkenyl Sulfides. *Org. Lett.* **2017**, *19*, 1100–1103.

[3] Tiwari, S.; Kumari, M.; Rawat, D. S.; Air Induced Phosphoryl Radical Mediated Stereoselective Hydrosulfonylation of Alkynes via Halogen Atom Transfer: Ingress of Z-Vinyl Sulfones. *Org. Lett.* **2024**, *26*, 2303–2308

[4] Zhu, M.; Alami, M.; Messaoudi, S. Room-Temperature Pd-Catalyzed Synthesis of 1-(Hetero)aryl Selenoglycosides. *Org. Lett.* **2020**, *22*, 6584-6589.

[5] Ding, Y.; Huang, Y.; Shi, W.; Zheng, N.; Wang, C.; Chen, X.; An, Y.; Zhang, Z.; Liang, Y. Modular Synthesis of Aryl Thio/Selenoglycosides via the Catallani Strategy. *Org. Lett.* **2021**, *23*, 5641-5646

12. NMR Spectra of compounds

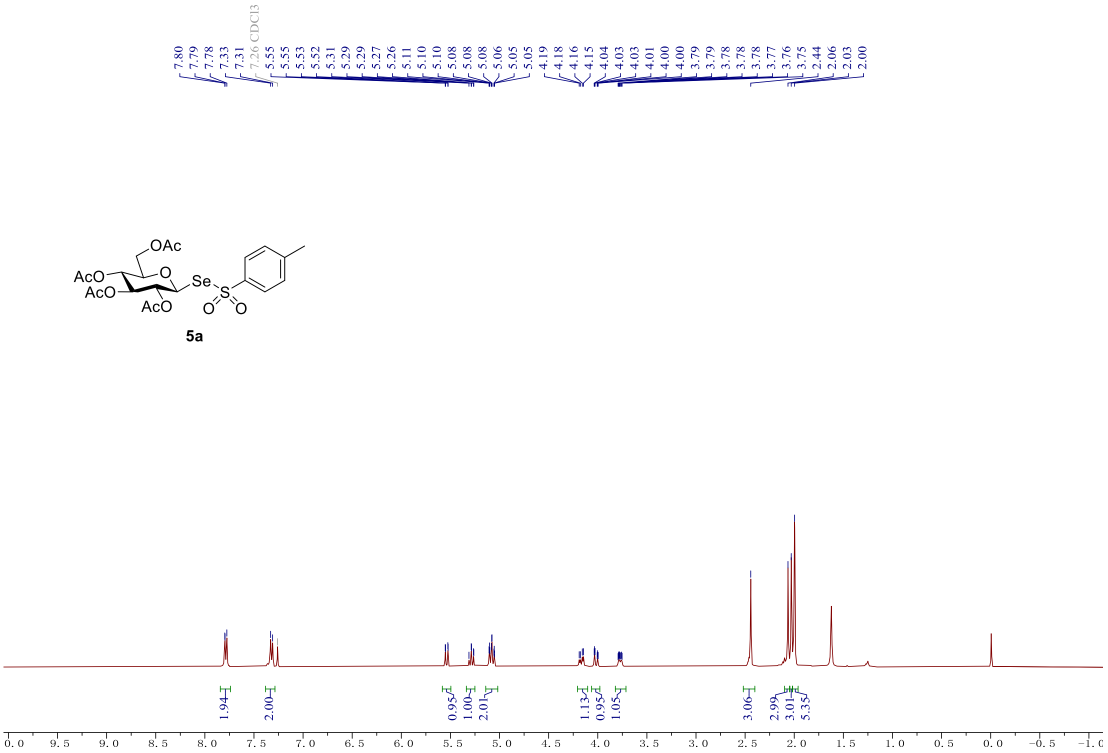

Figure S18. ^{1}H NMR (400 MHz, $CDCl_3$) Spectra for compound **5a**

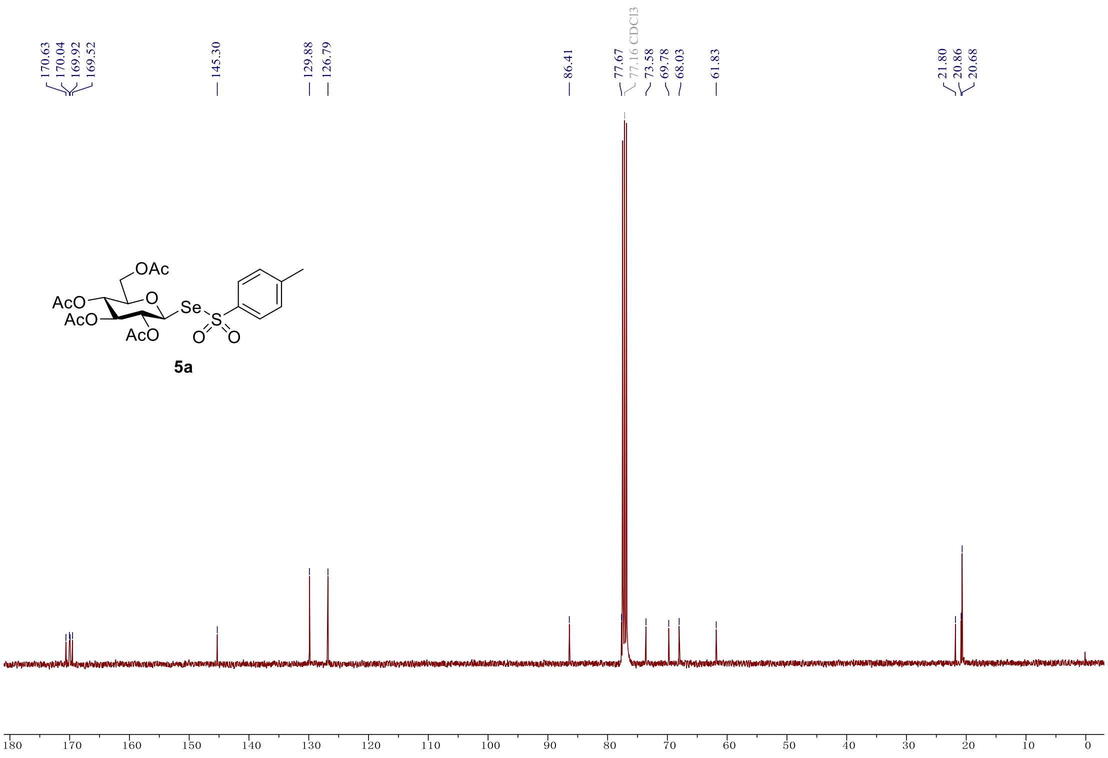

Figure S19. ^{13}C NMR (101 MHz, $CDCl_3$) Spectra for compound **5a**

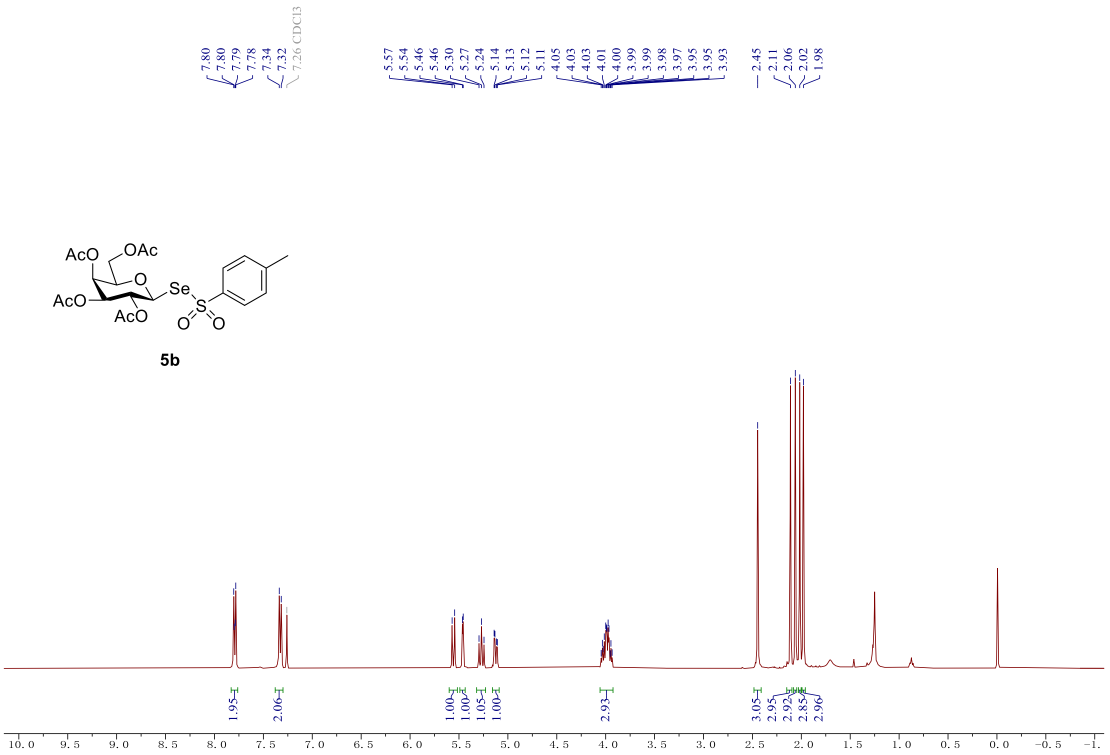

Figure S20. ^{1}H NMR (400 MHz, $CDCl_3$) Spectra for compound **5b**

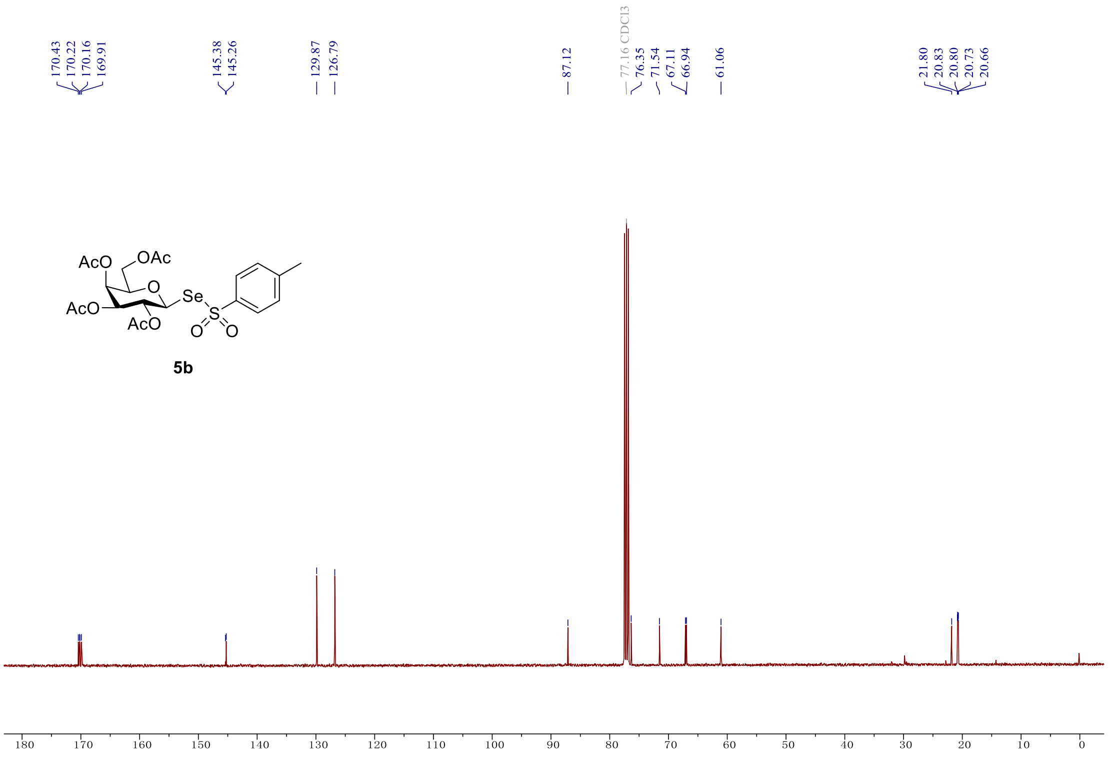

Figure S21. ^{13}C NMR (101 MHz, $CDCl_3$) Spectra for compound **5b**

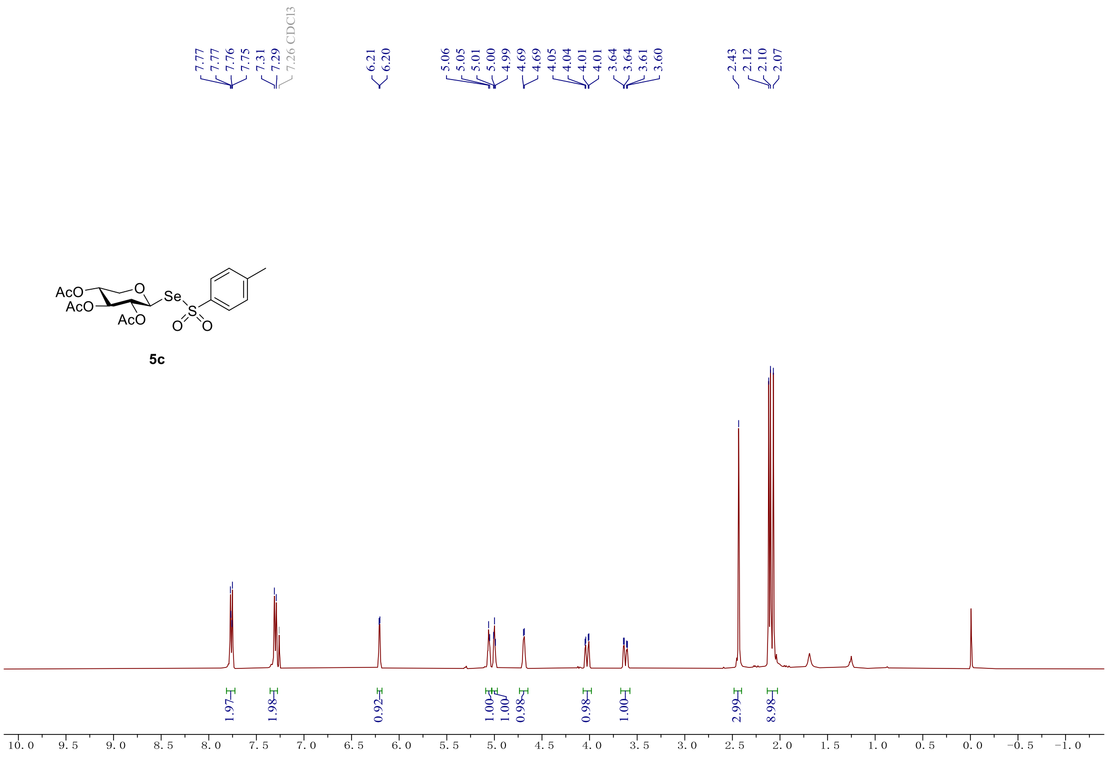

Figure S22. ^{1}H NMR (400 MHz, $CDCl_3$) Spectra for compound **5c**

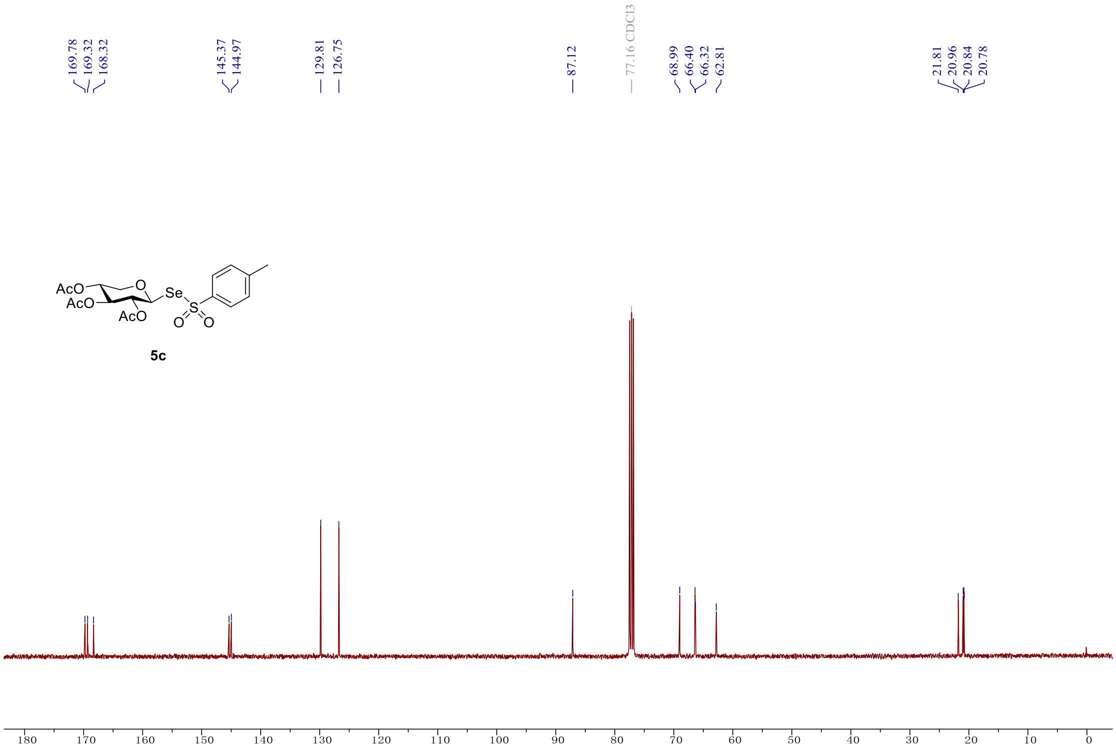

Figure S23. ^{13}C NMR (101 MHz, $CDCl_3$) Spectra for compound **5c**

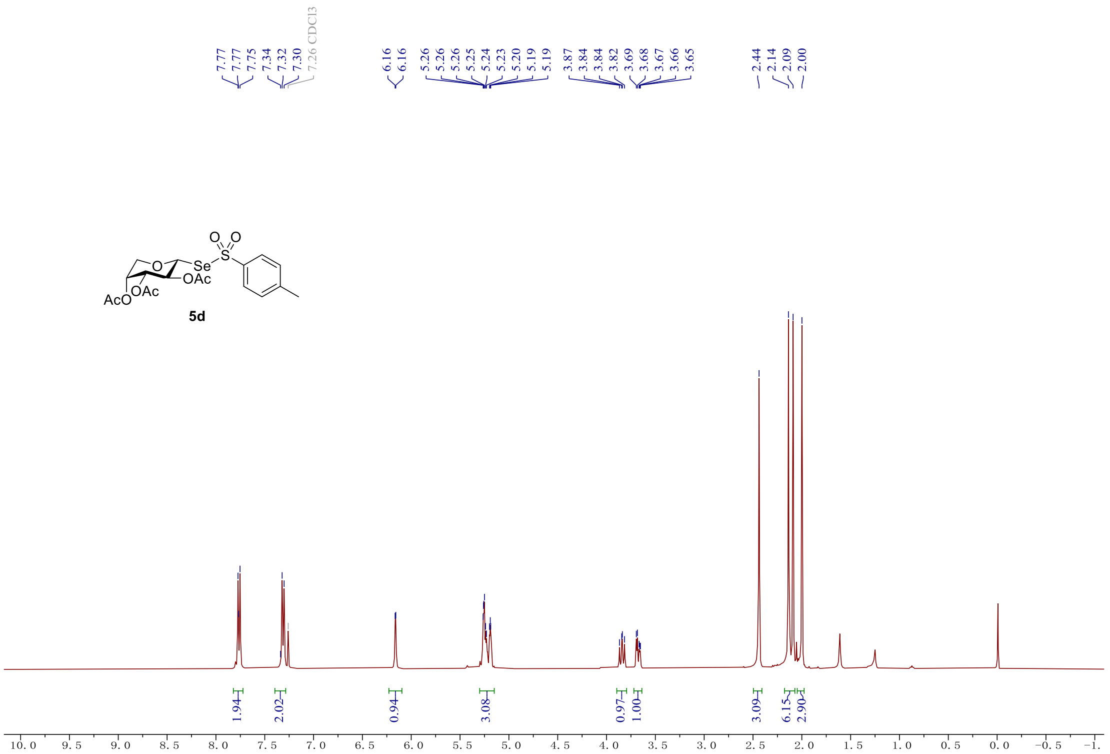

Figure S24. ^{1}H NMR (400 MHz, $CDCl_3$) Spectra for compound **5d**

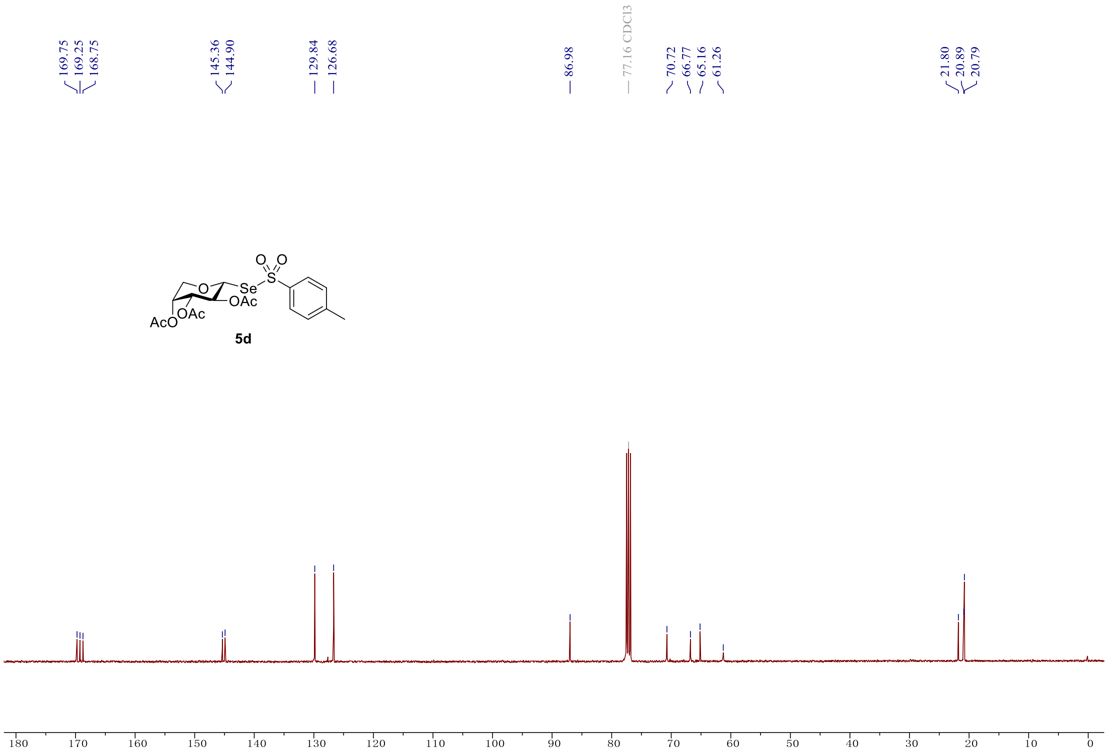

Figure S25. ^{13}C NMR (101 MHz, $CDCl_3$) Spectra for compound **5d**

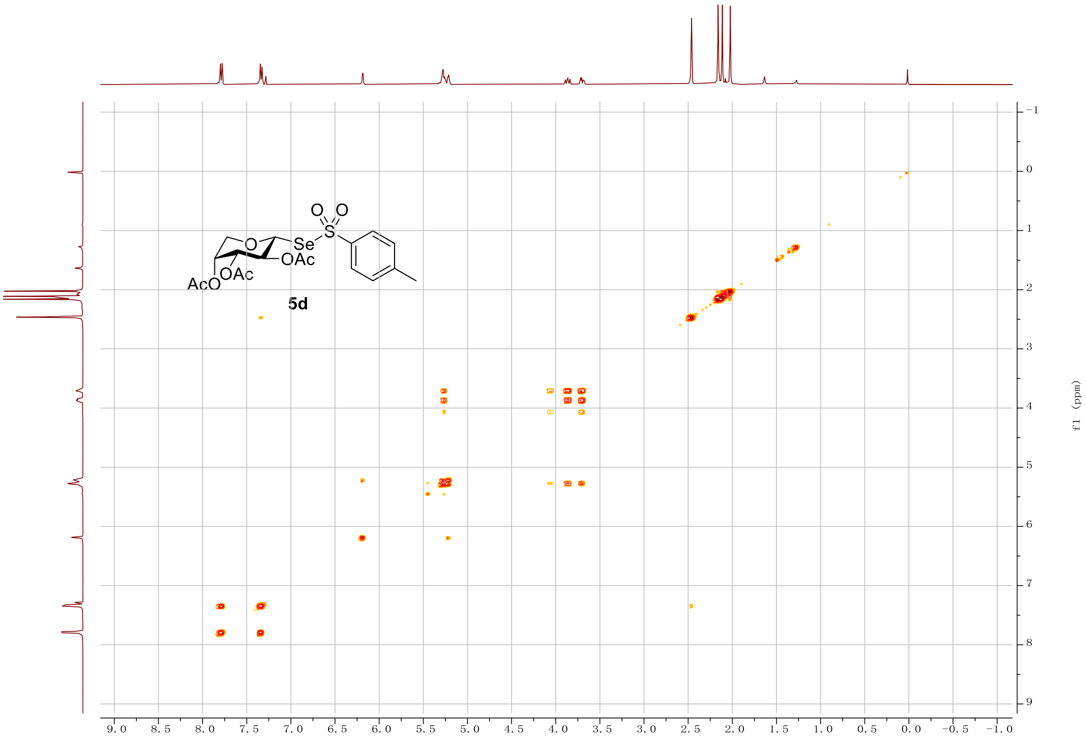

Figure S26. COSY Spectra for compound **5d**

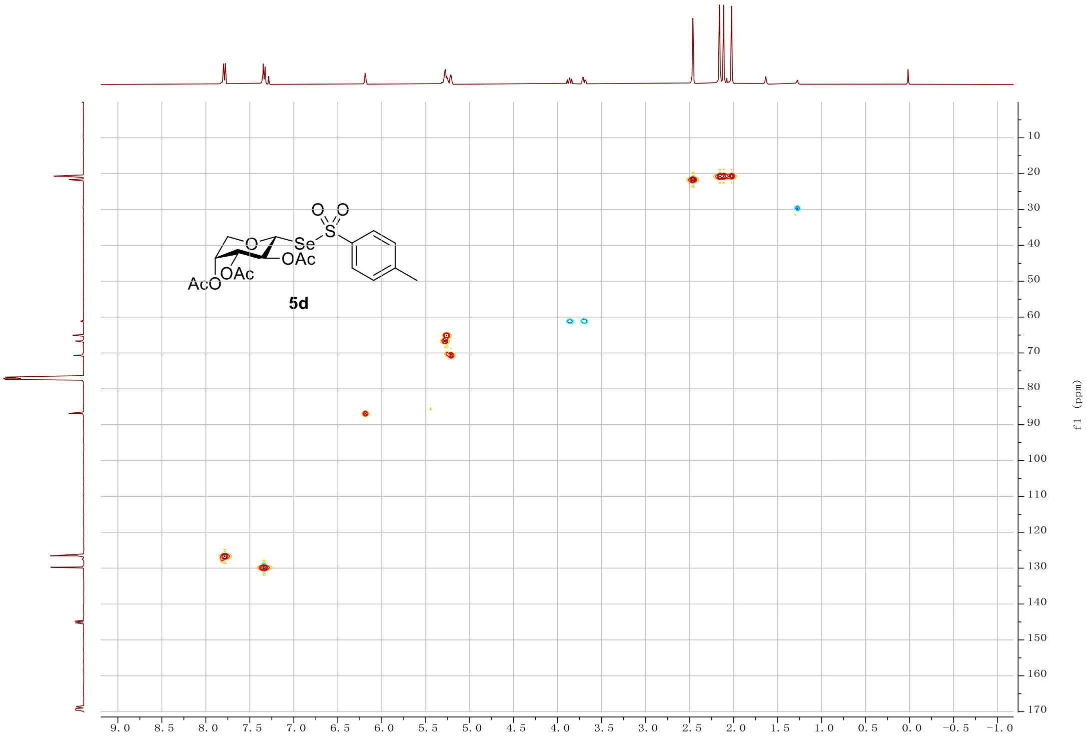

Figure S27. HSQC Spectra for compound **5d**

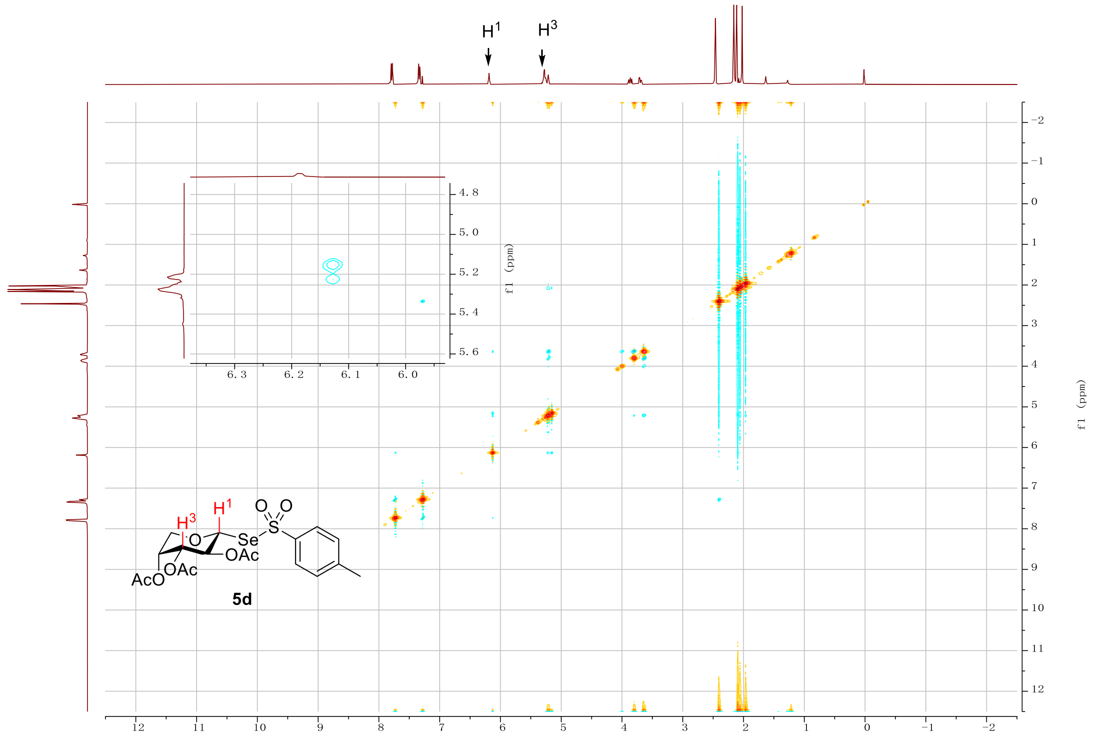

Figure S28. NOESY Spectra for compound **5d**

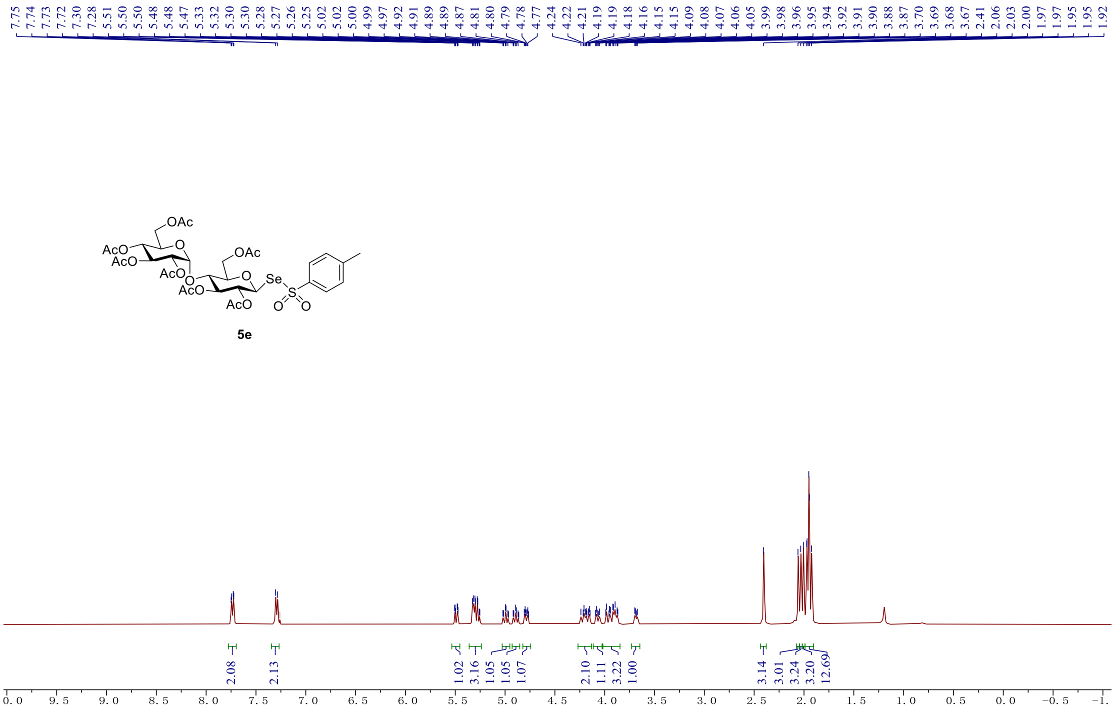

Figure S29. ^{1}H NMR (400 MHz, $CDCl_3$) Spectra for compound **5e**

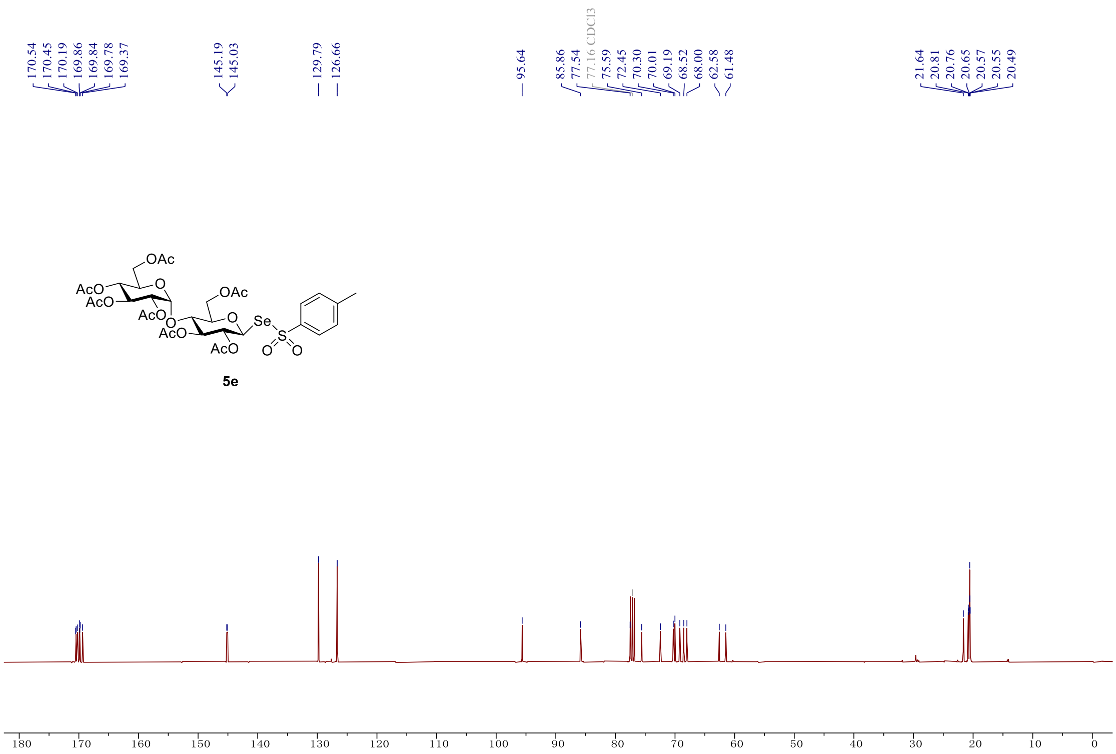

Figure S30. ^{13}C NMR (101 MHz, $CDCl_3$) Spectra for compound **5e**

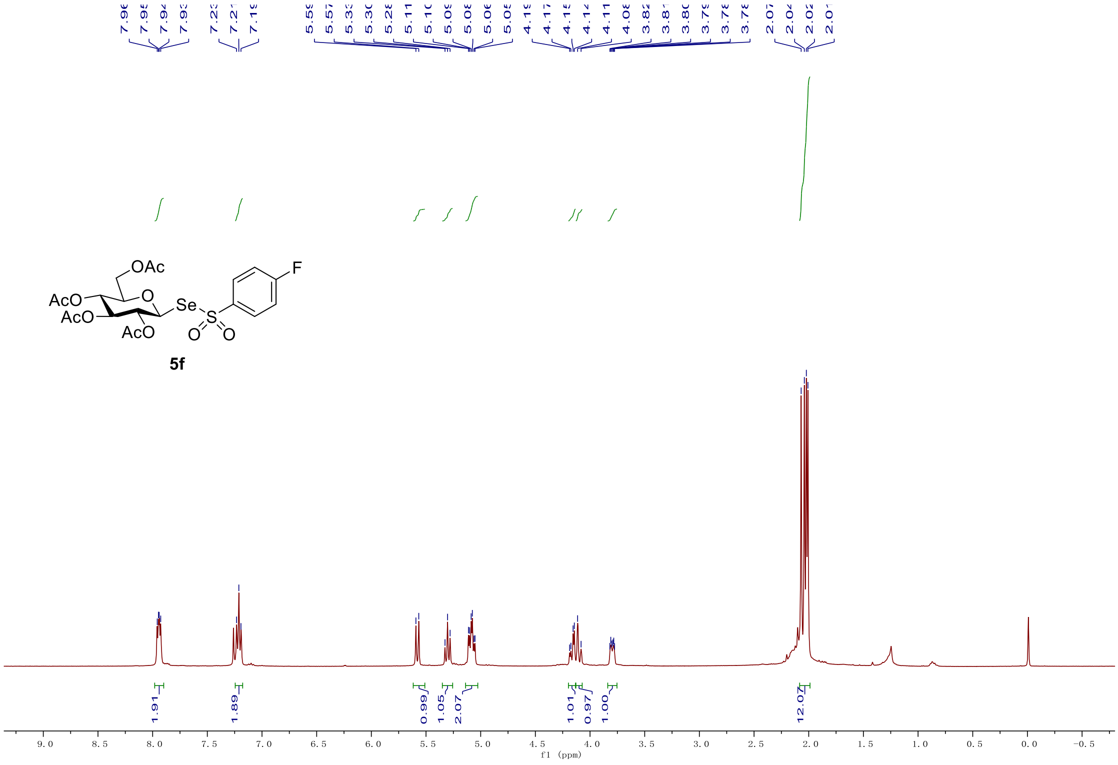

Figure S31. ^{1}H NMR (400 MHz, $CDCl_3$) Spectra for compound **5f**

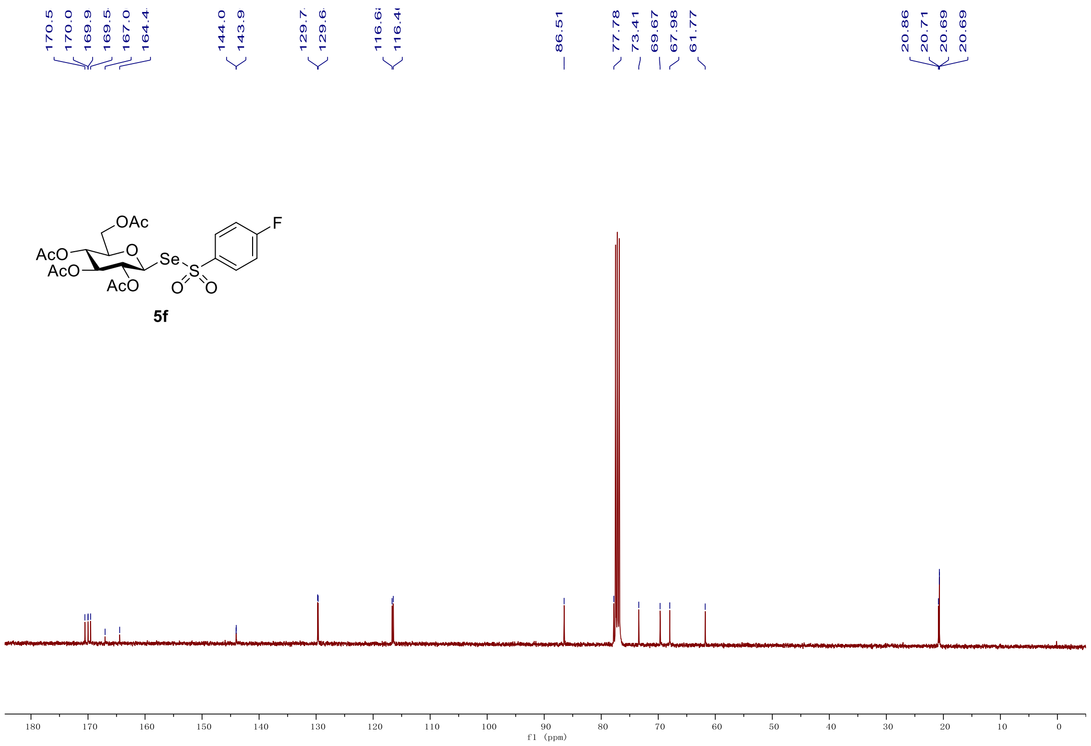

Figure S32. ^{13}C NMR (101 MHz, $CDCl_3$) Spectra for compound **5f**

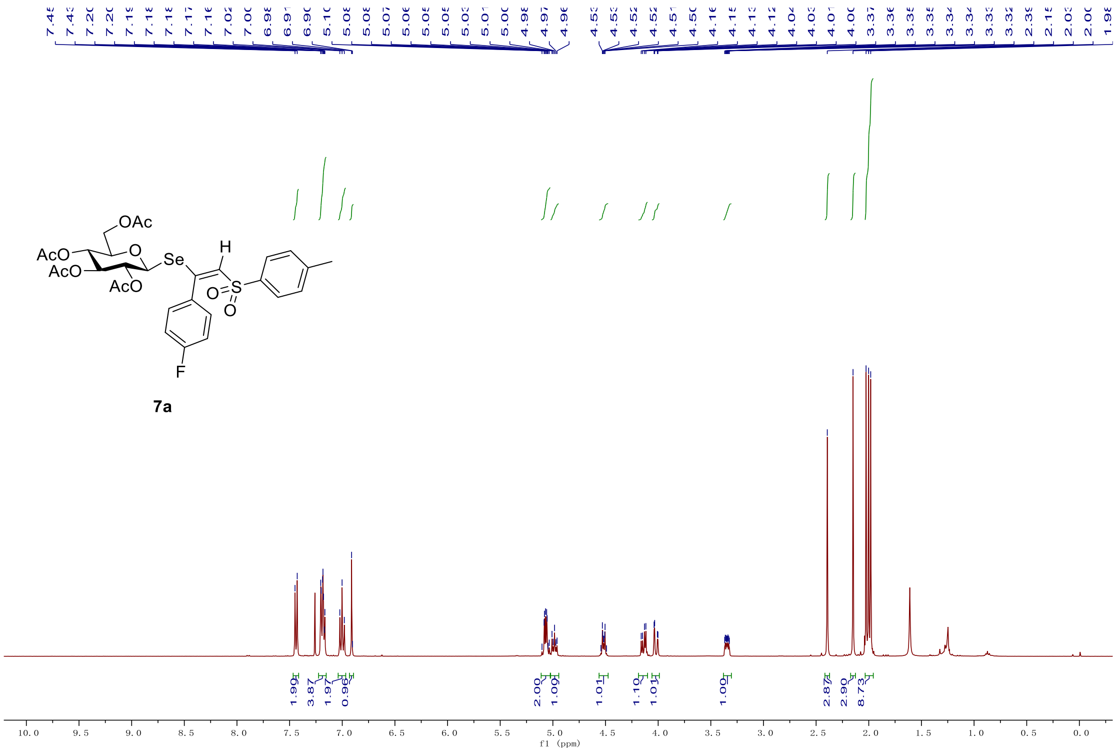

Figure S33. ^{1}H NMR (400 MHz, $CDCl_3$) Spectra for compound **7a**

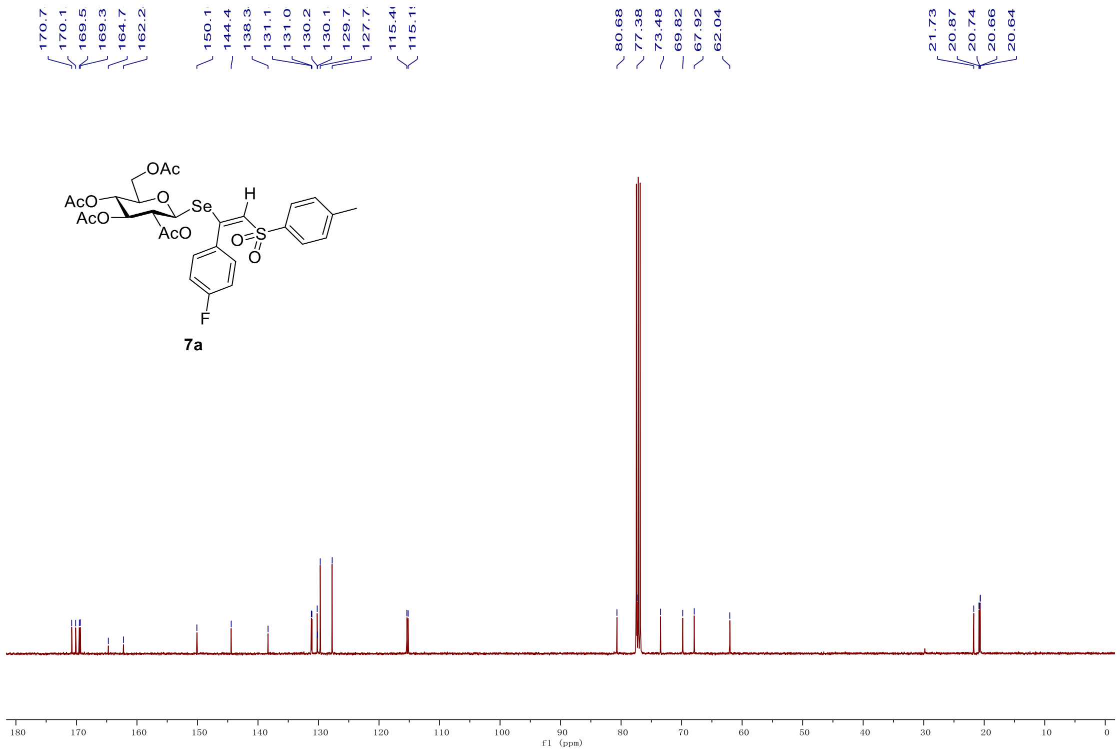

Figure S34. ^{13}C NMR (101 MHz, CDCl$_3$) Spectra for compound **7a**

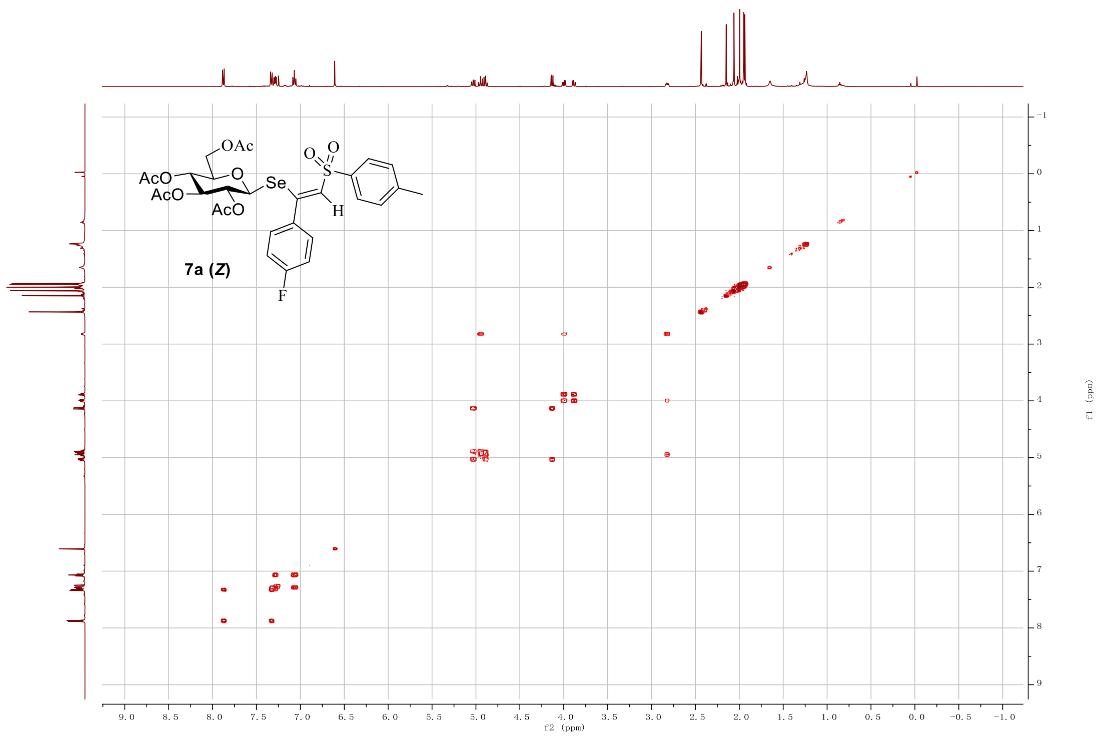

Figure S35. COSY Spectra for compound **7a** (***Z***)

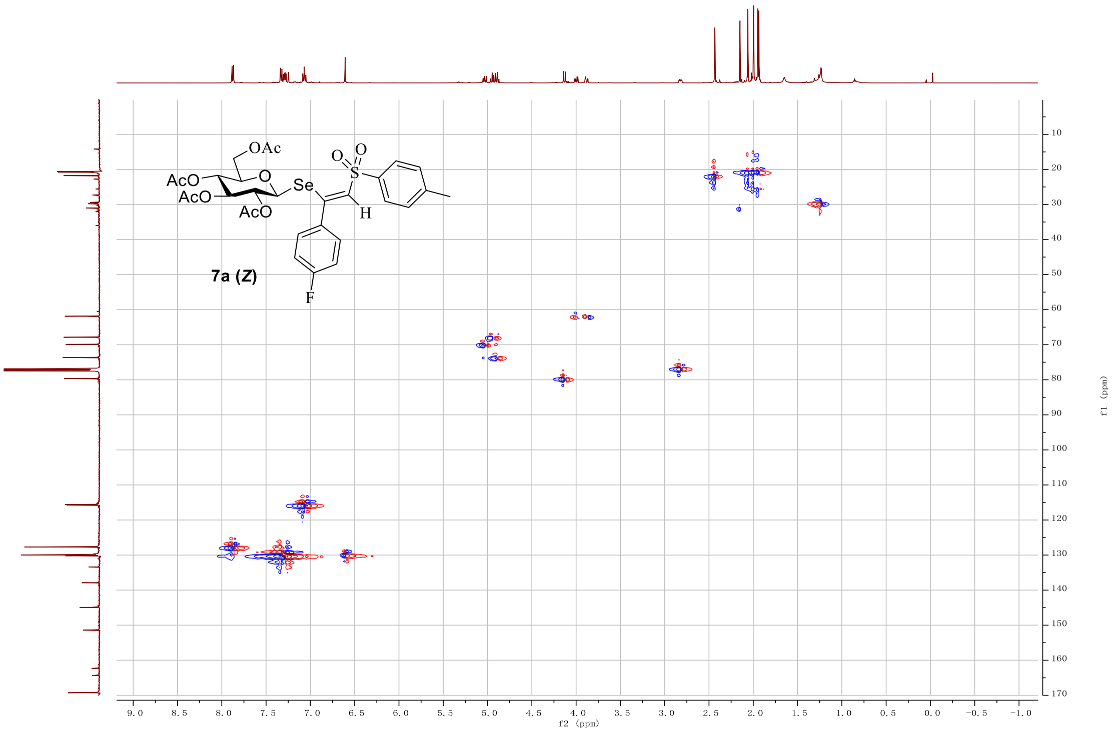

Figure S36. HSQC Spectra for compound **7a** (***Z***)

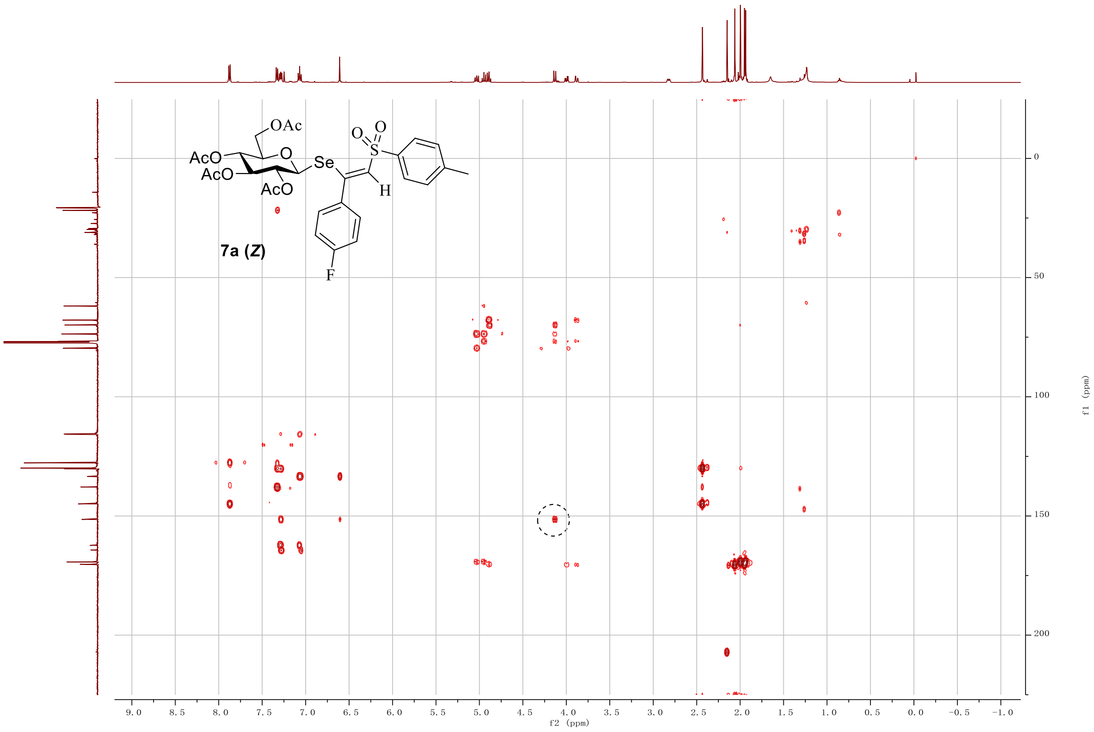

Figure S37. HMBC Spectra for compound **7a** (***Z***)

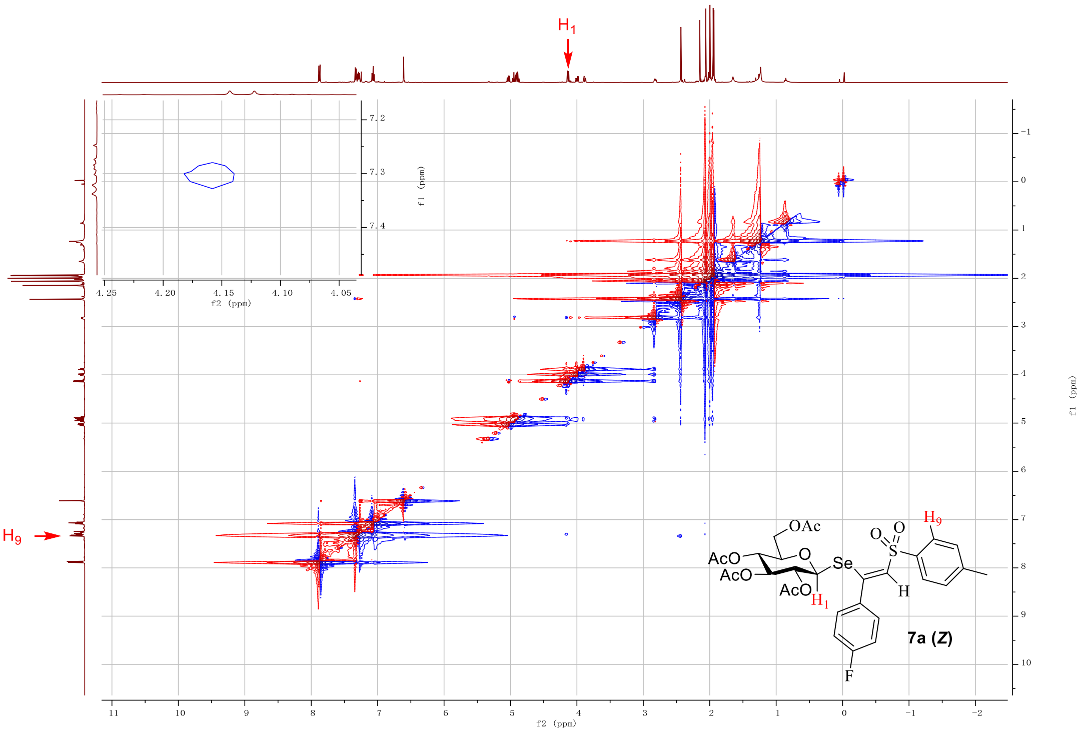

Figure S38. NOESY Spectra for compound **7a (*Z*)**

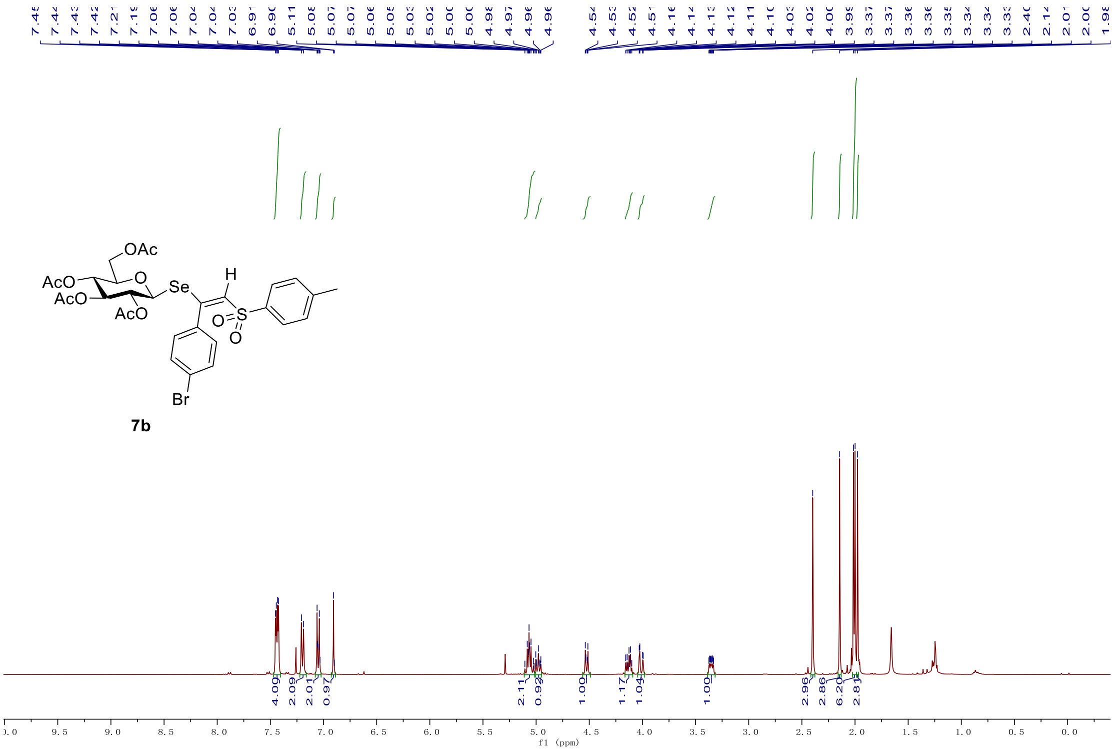

Figure S39. ^{1}H NMR (400 MHz, $CDCl_3$) Spectra for compound **7b**

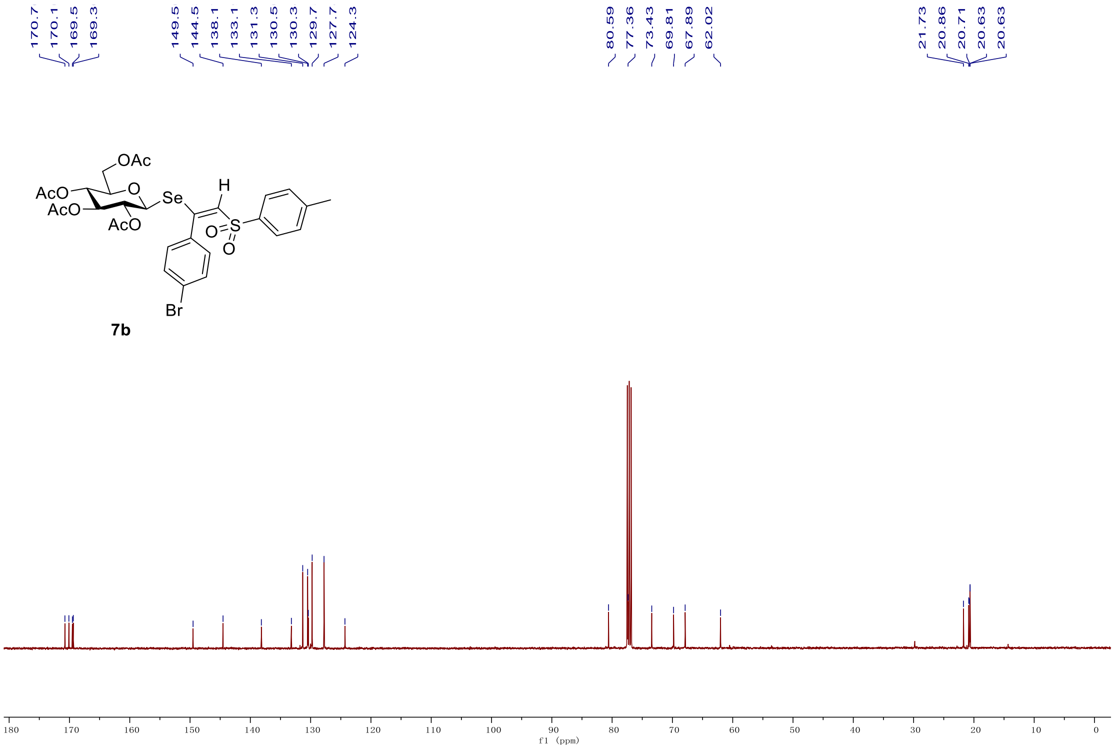

Figure S40. ^{13}C NMR (101 MHz, CDCl$_3$) Spectra for compound **7b**

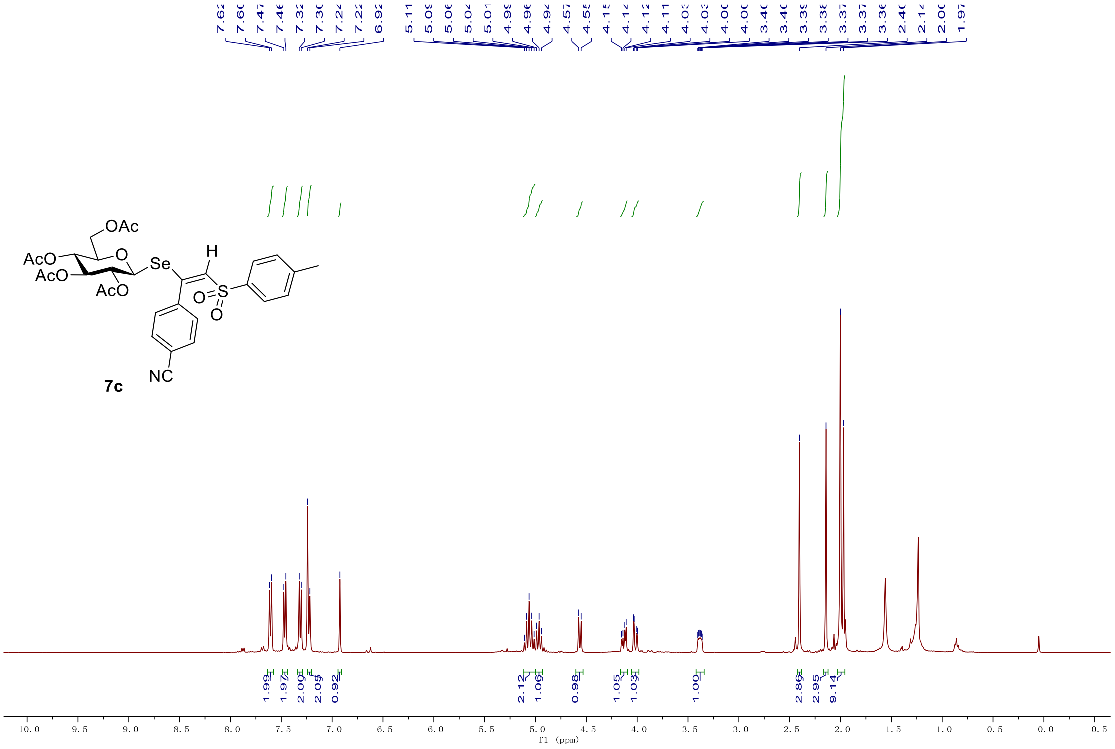

Figure S41. ^{1}H NMR (400 MHz, CDCl$_3$) Spectra for compound **7c**

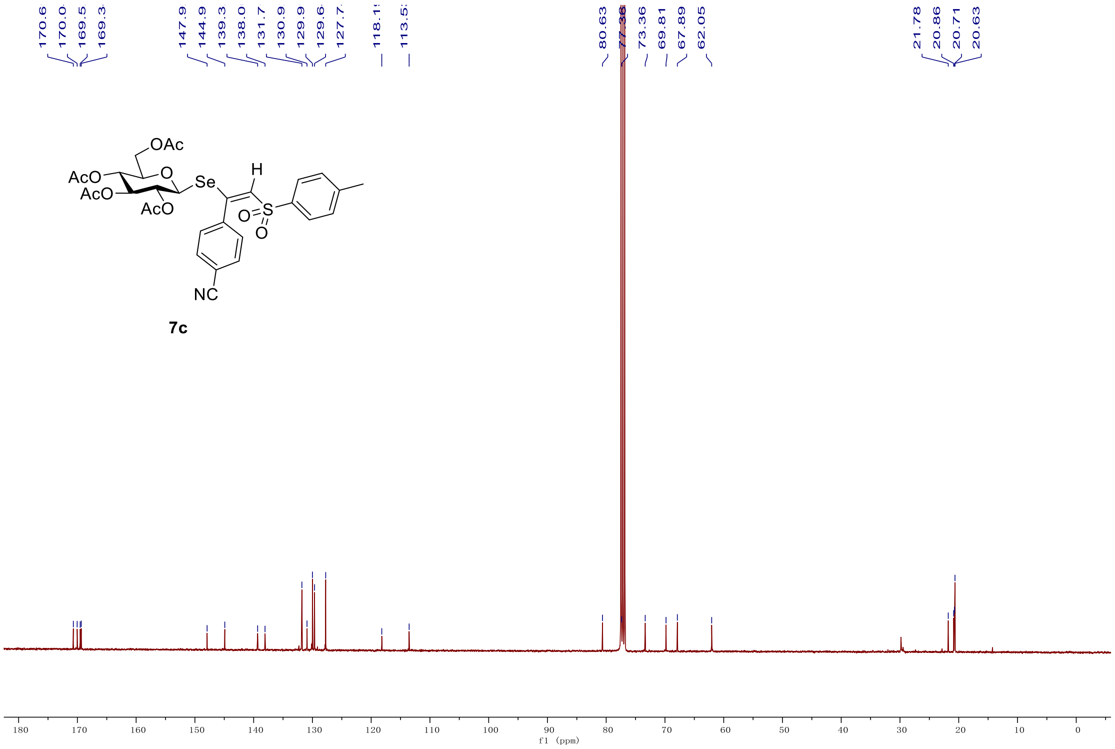

Figure S42. ^{13}C NMR (101 MHz, $CDCl_3$) Spectra for compound **7c**

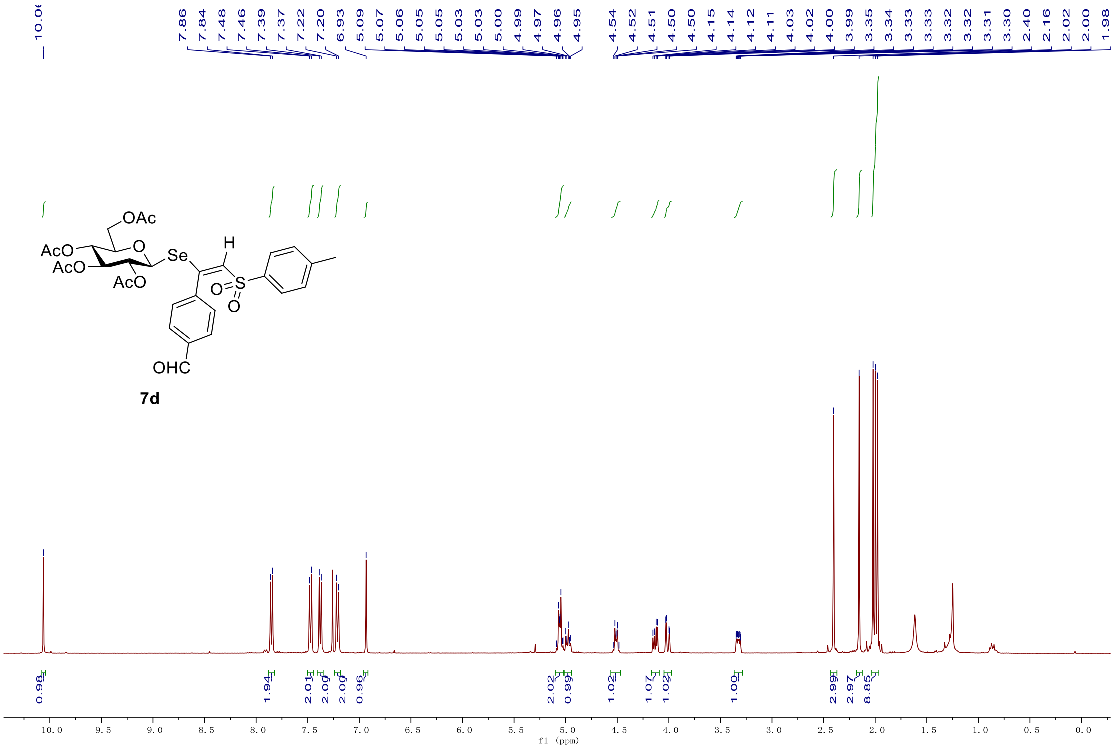

Figure S43. ^{1}H NMR (400 MHz, $CDCl_3$) Spectra for compound **7d**

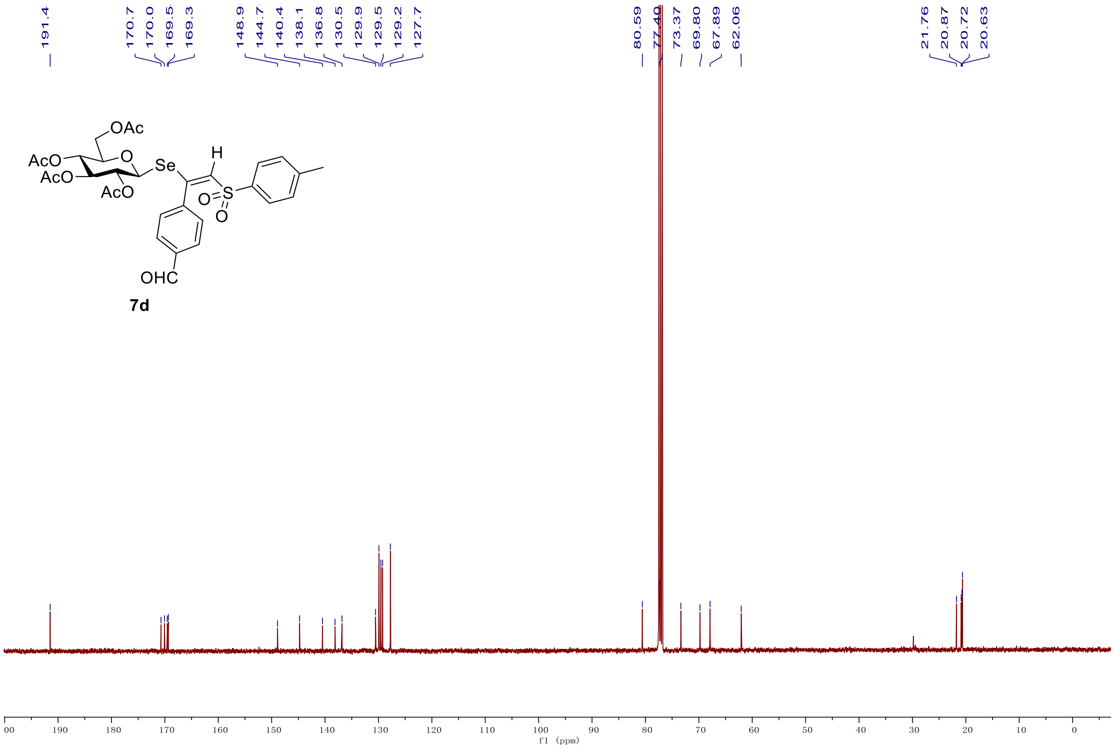

Figure S44. ^{13}C NMR (101 MHz, $CDCl_3$) Spectra for compound **7d**

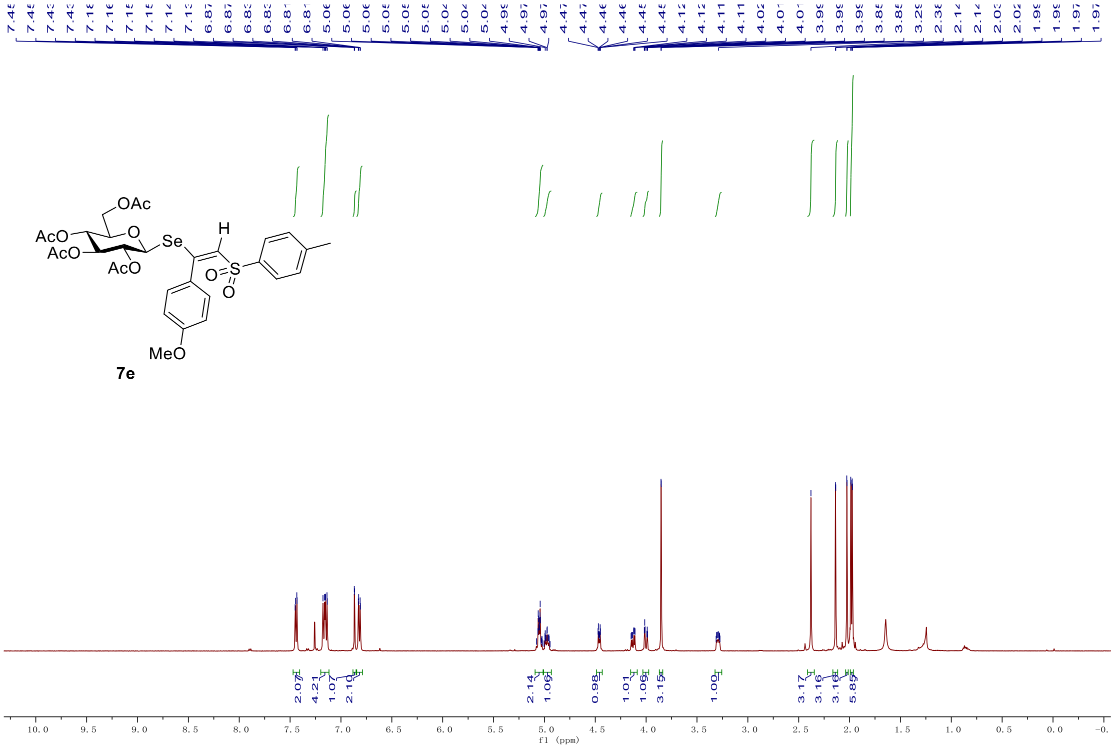

Figure S45. ^{1}H NMR (400 MHz, $CDCl_3$) Spectra for compound **7e**

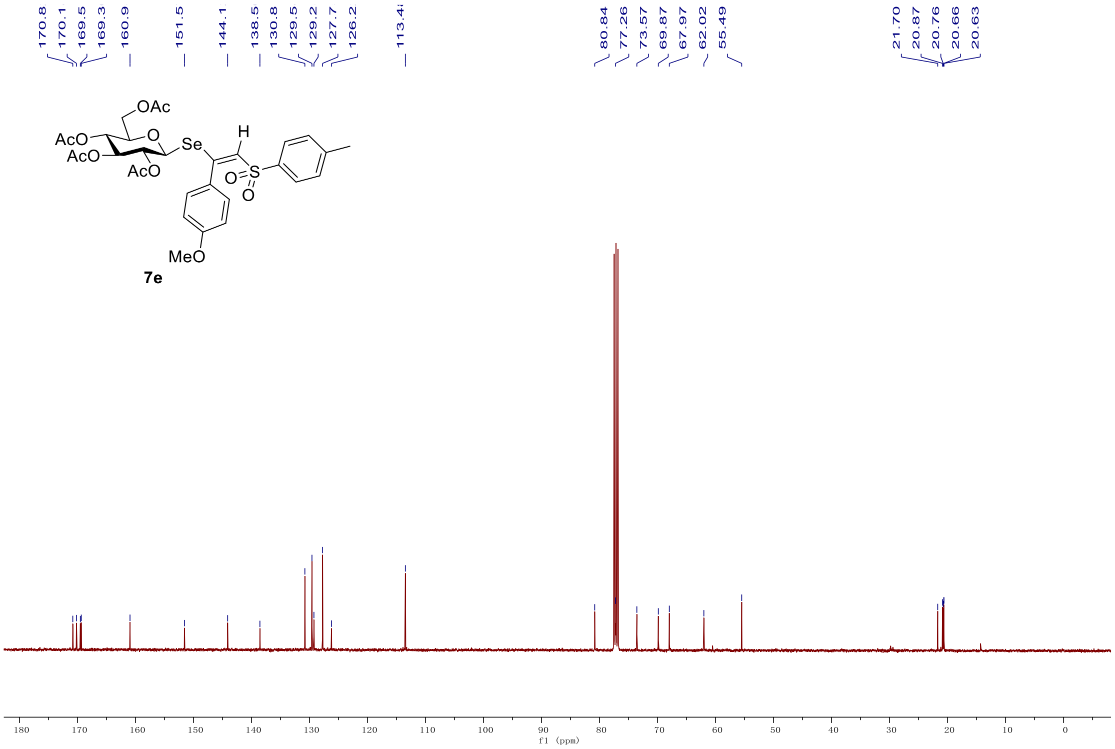

Figure S46. ^{13}C NMR (101 MHz, $CDCl_3$) Spectra for compound **7e**

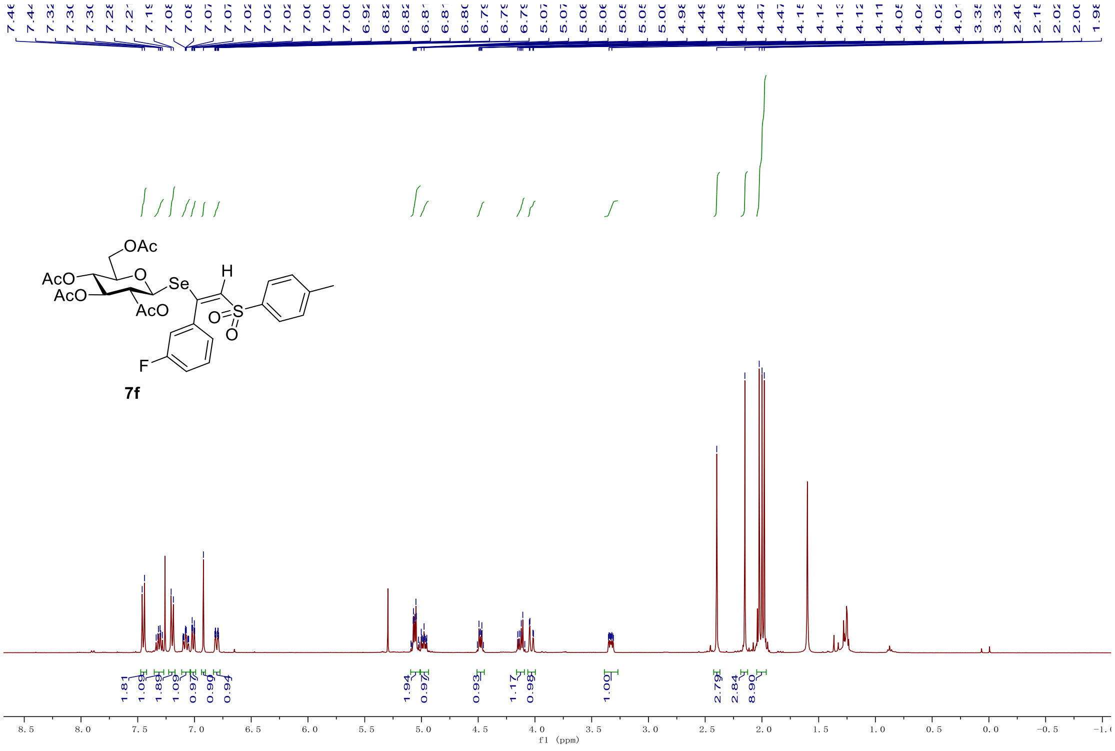

Figure S47. ^{1}H NMR (400 MHz, $CDCl_3$) Spectra for compound **7f**

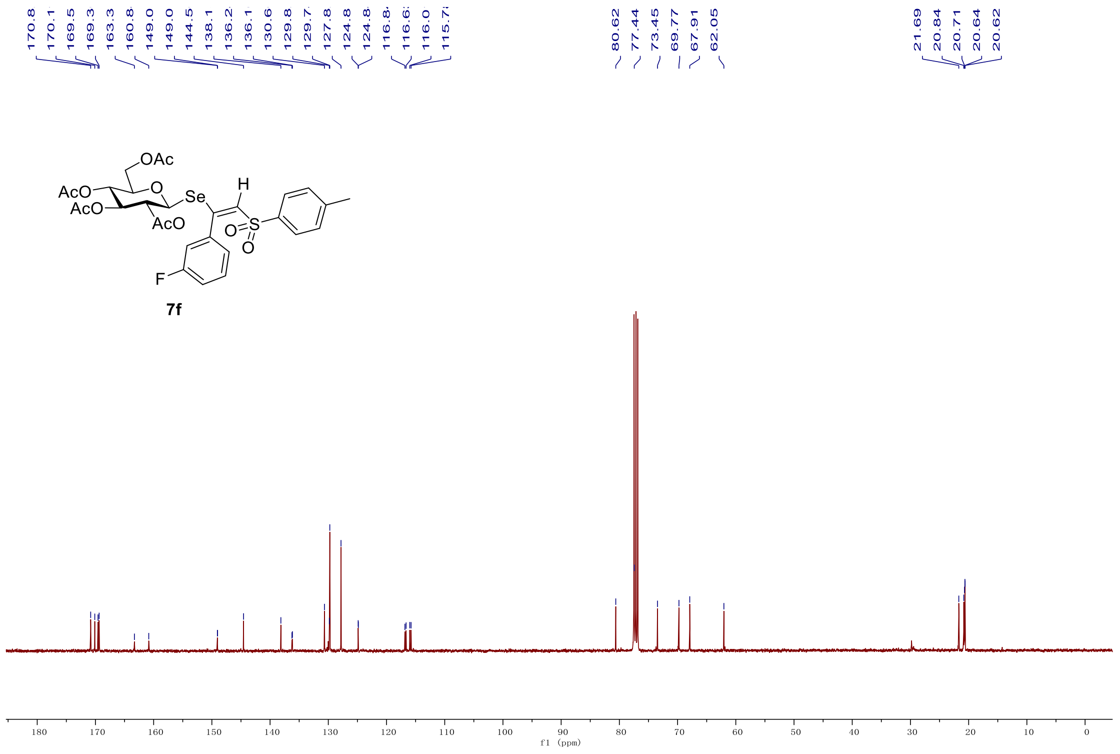

Figure S48. ^{13}C NMR (101 MHz, $CDCl_3$) Spectra for compound **7f**

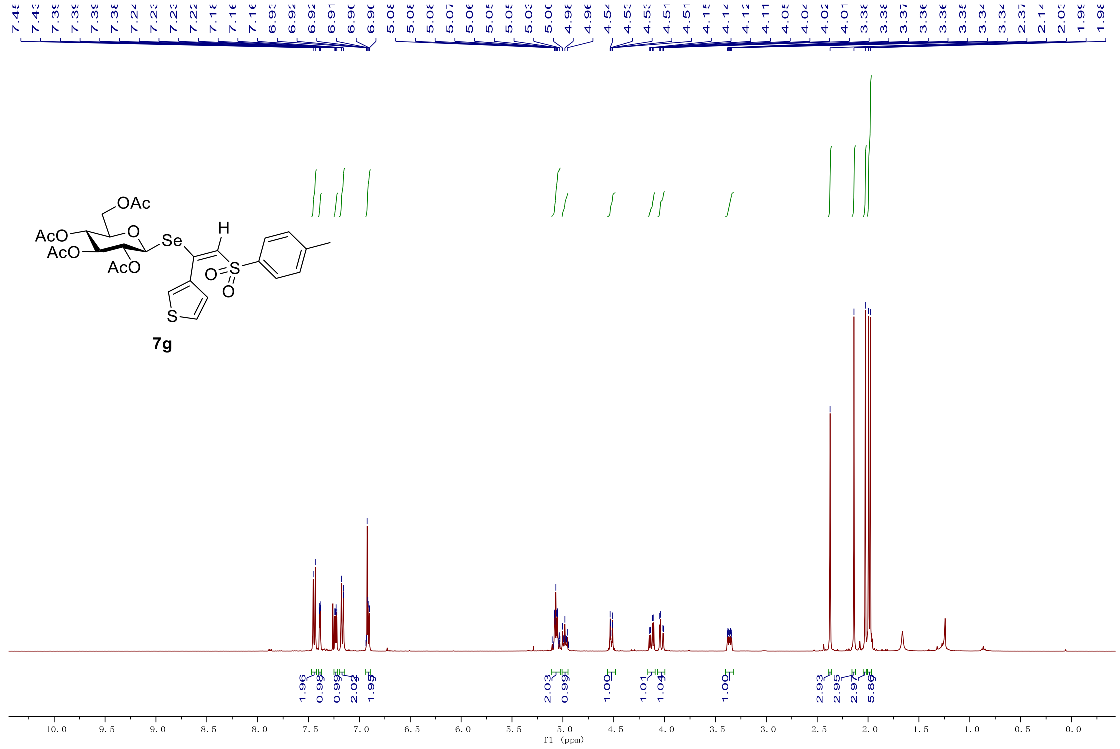

Figure S49. ^{1}H NMR (400 MHz, $CDCl_3$) Spectra for compound **7g**

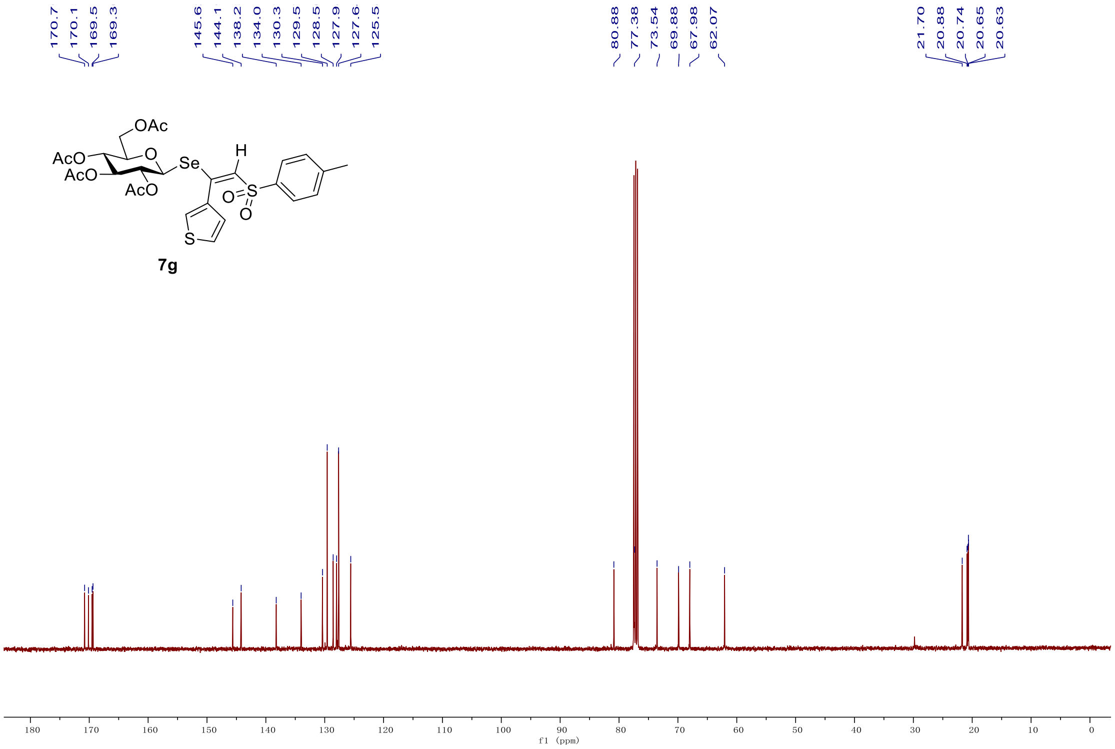

Figure S50. ^{13}C NMR (101 MHz, CDCl$_3$) Spectra for compound **7g**

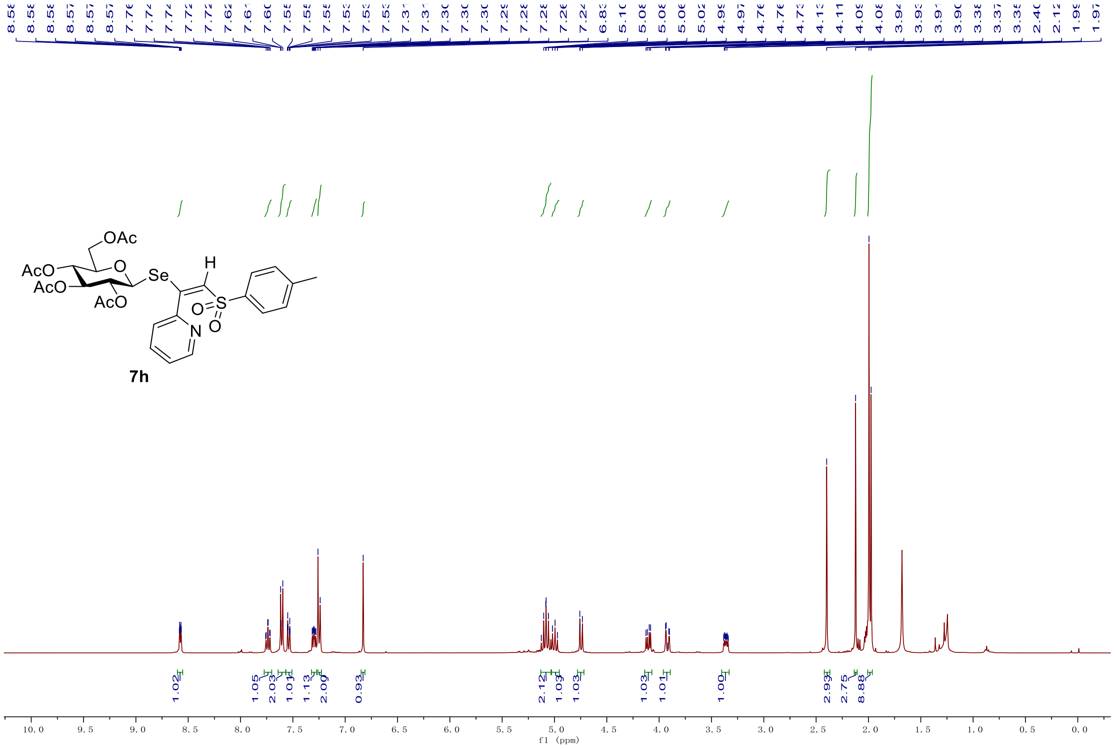

Figure S51. ^{1}H NMR (400 MHz, CDCl$_3$) Spectra for compound **7h**

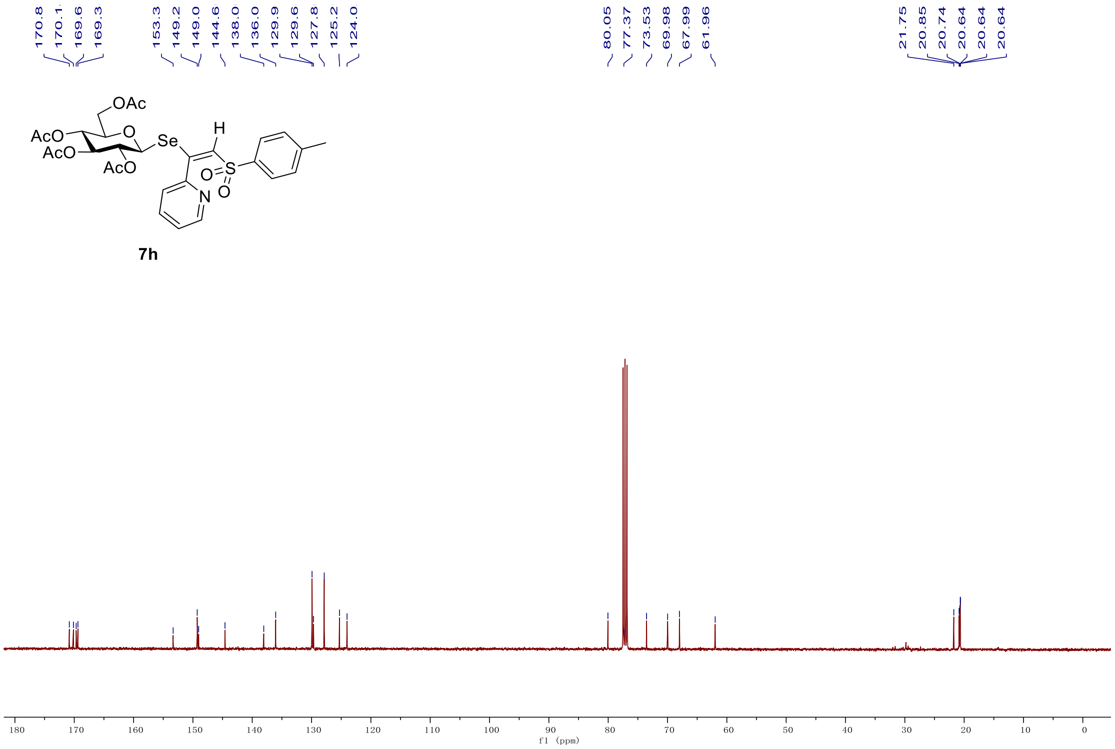

Figure S52. ^{13}C NMR (101 MHz, $CDCl_3$) Spectra for compound **7h**

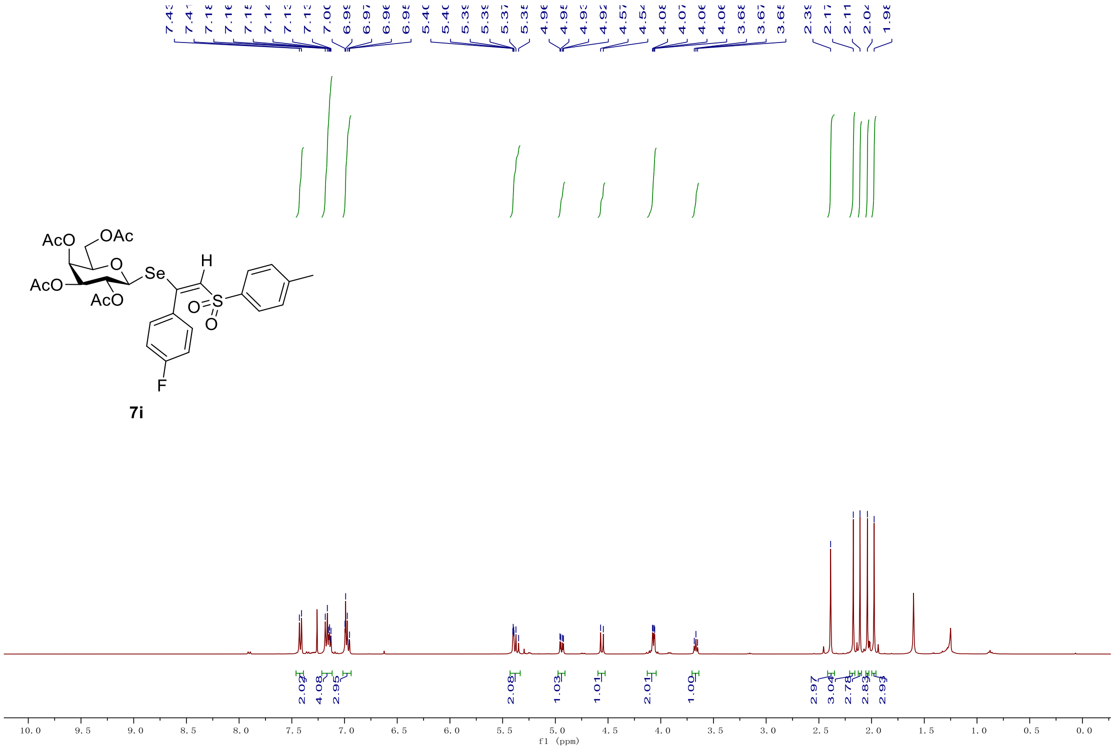

Figure S53. ^{1}H NMR (400 MHz, $CDCl_3$) Spectra for compound **7i**

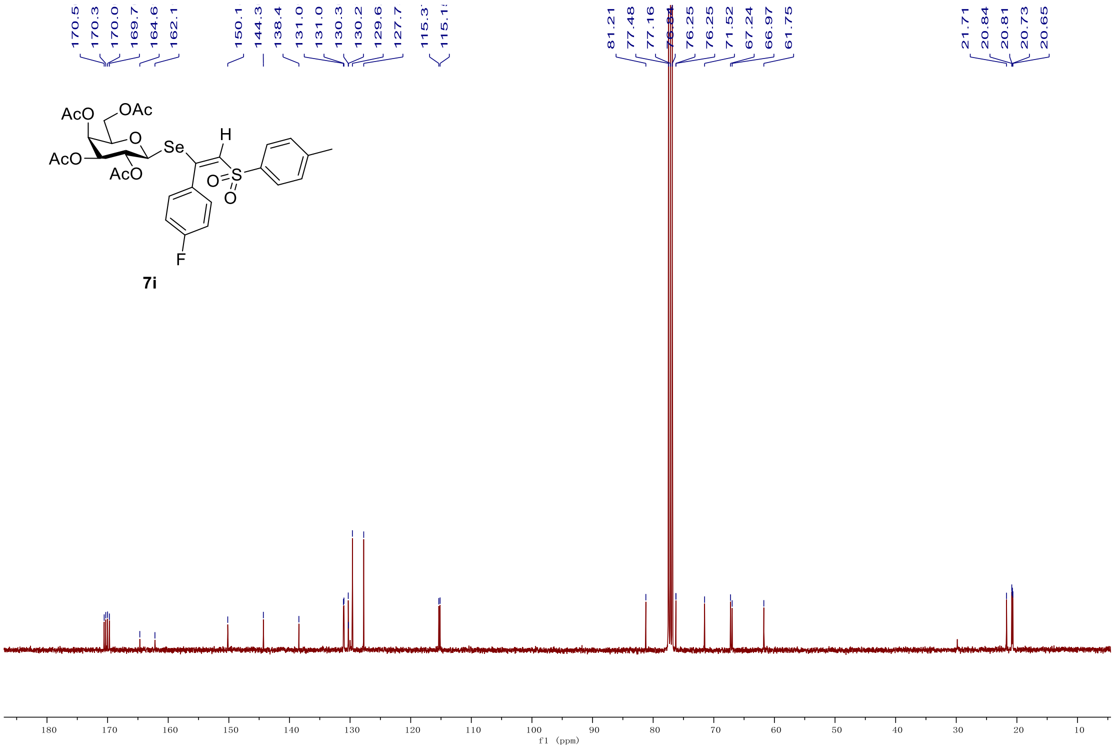

Figure S54. ^{13}C NMR (101 MHz, $CDCl_3$) Spectra for compound **7i**

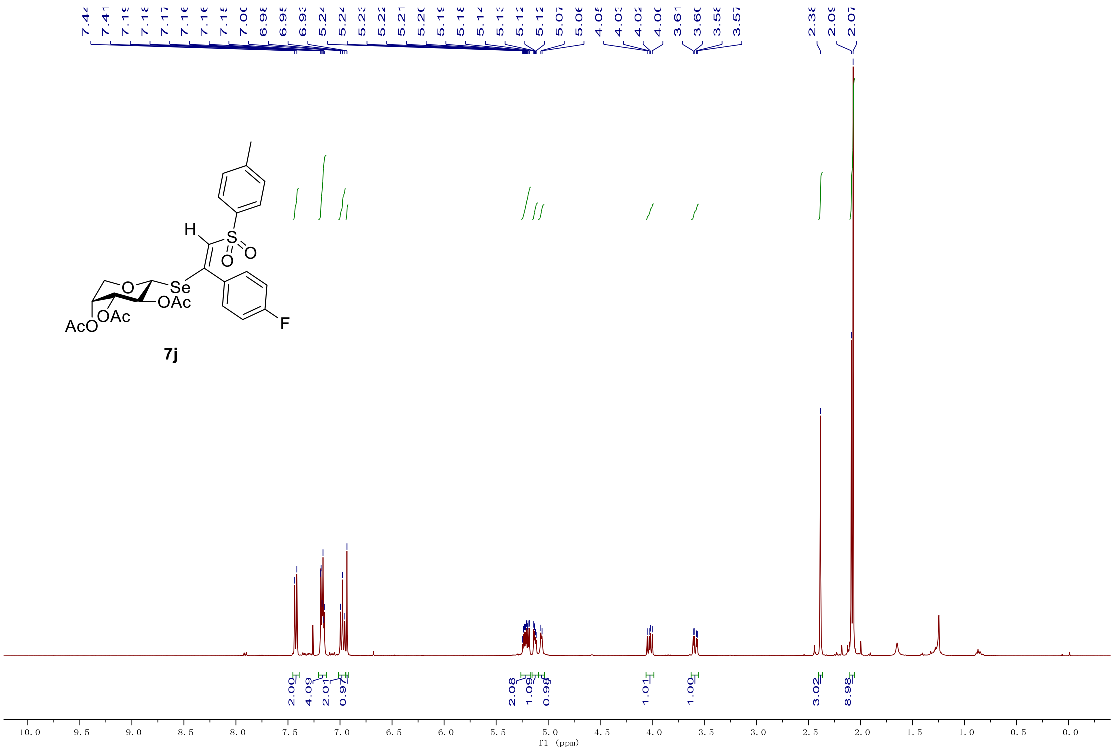

Figure S55. ^{1}H NMR (400 MHz, $CDCl_3$) Spectra for compound **7j**

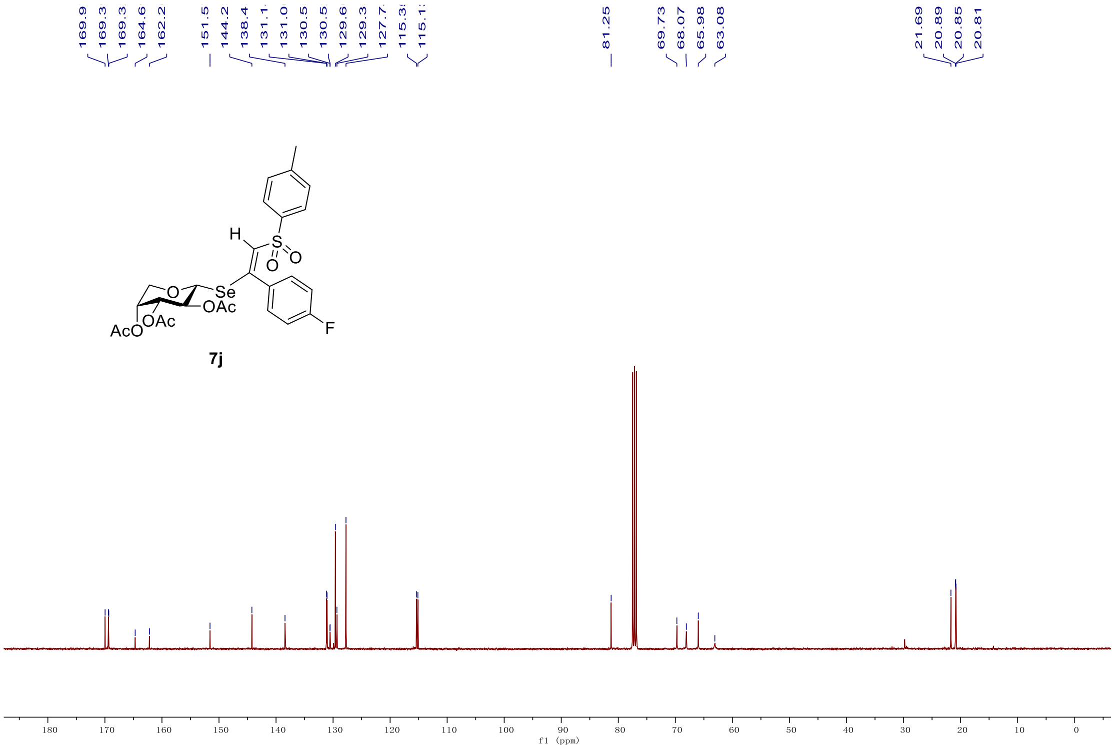

Figure S56. ^{13}C NMR (101 MHz, $CDCl_3$) Spectra for compound **7j**

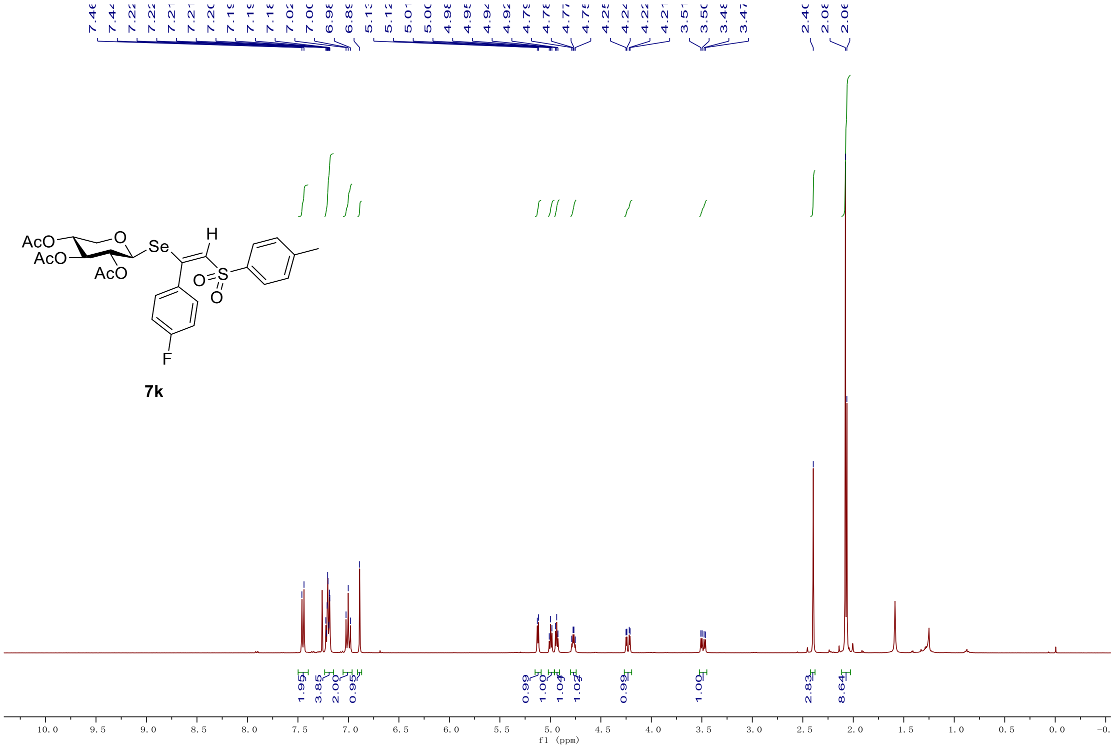

Figure S57. ^{1}H NMR (400 MHz, $CDCl_3$) Spectra for compound **7k**

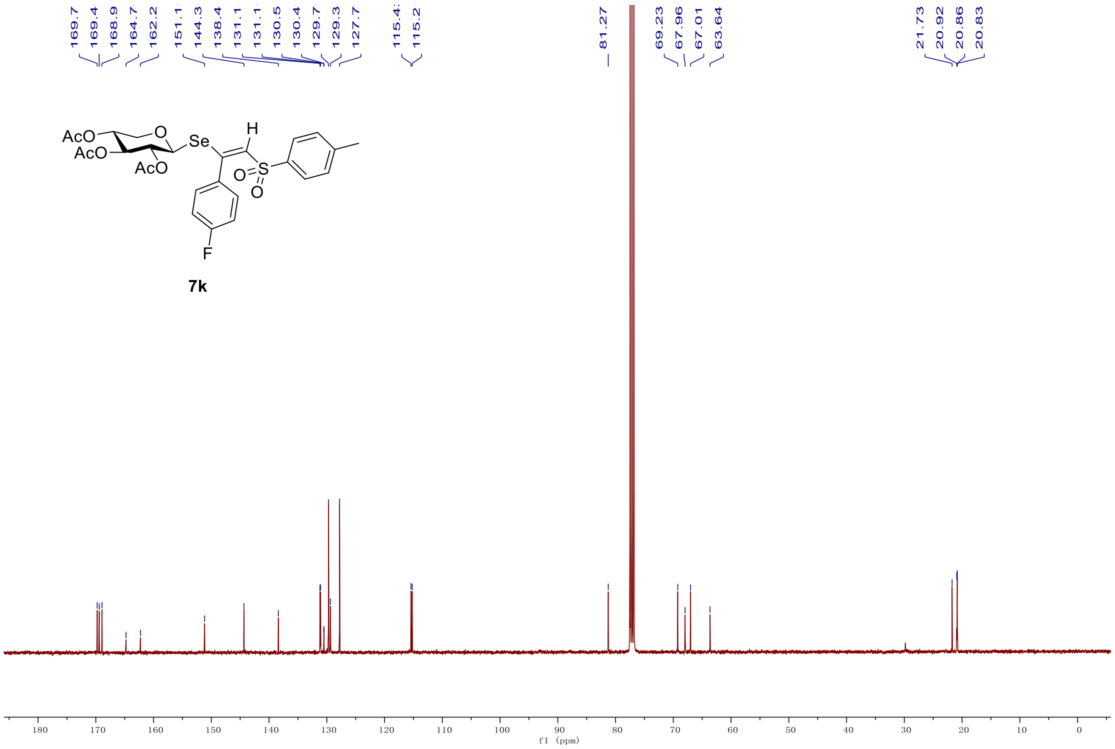

Figure S58. ^{13}C NMR (101 MHz, $CDCl_3$) Spectra for compound **7k**

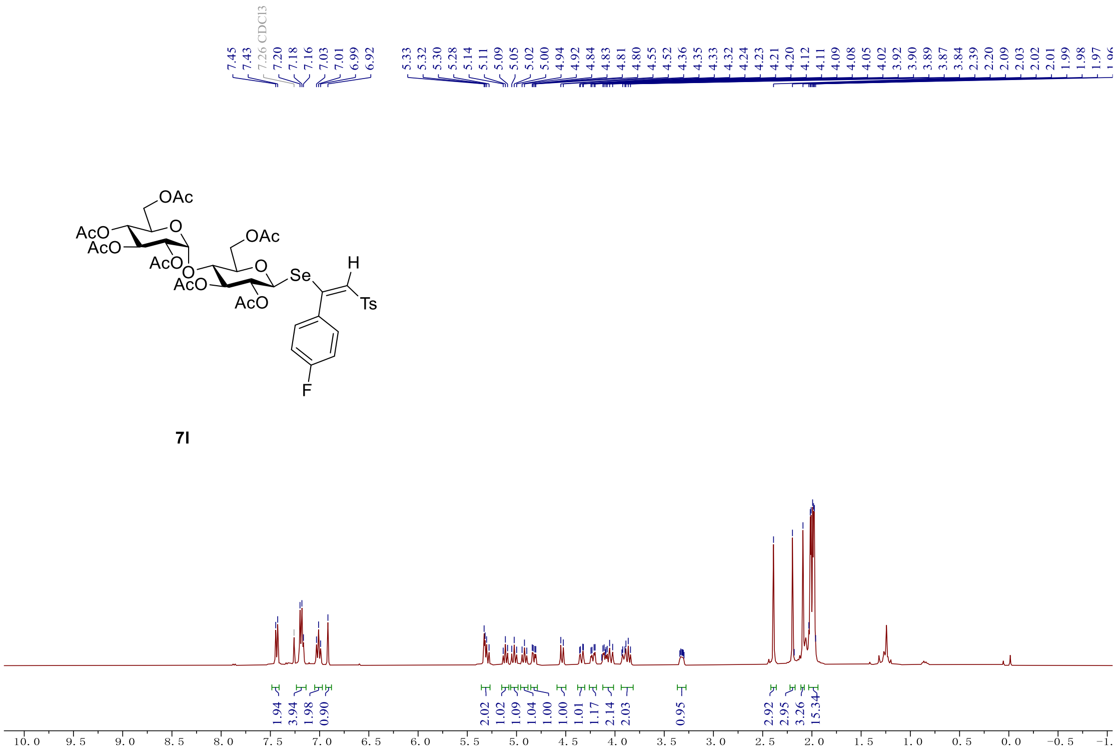

Figure S59. ^{1}H NMR (400 MHz, $CDCl_3$) Spectra for compound **7l**

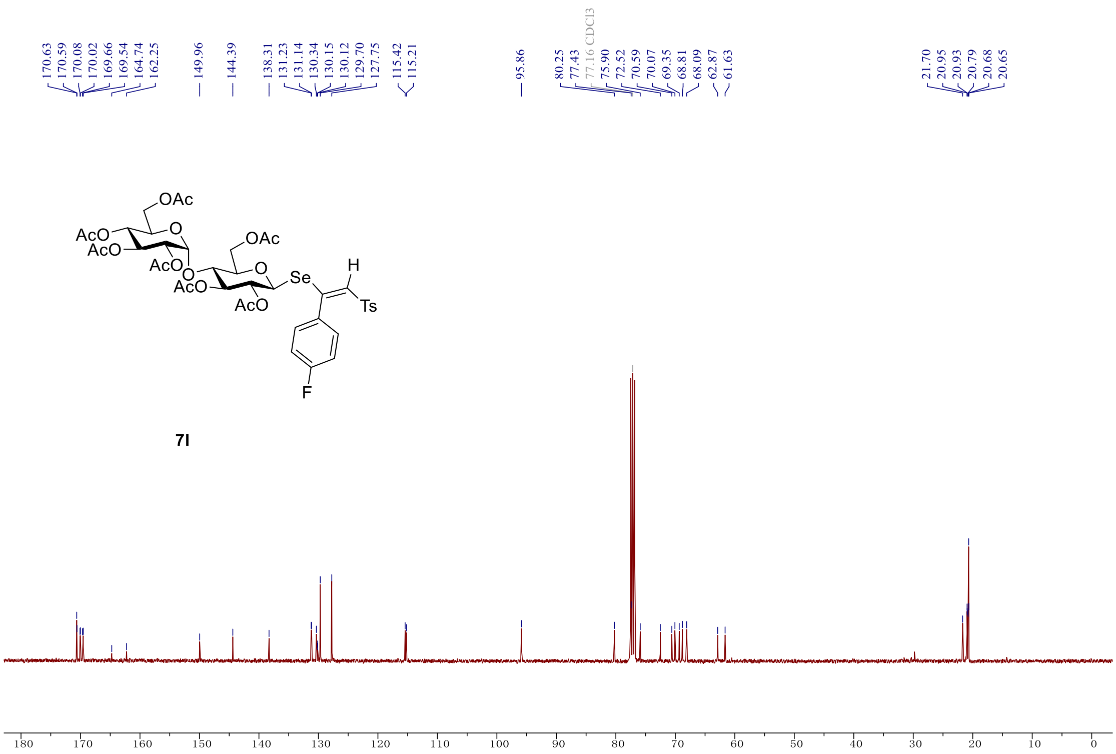

Figure S60. ^{13}C NMR (101 MHz, $CDCl_3$) Spectra for compound **7l**

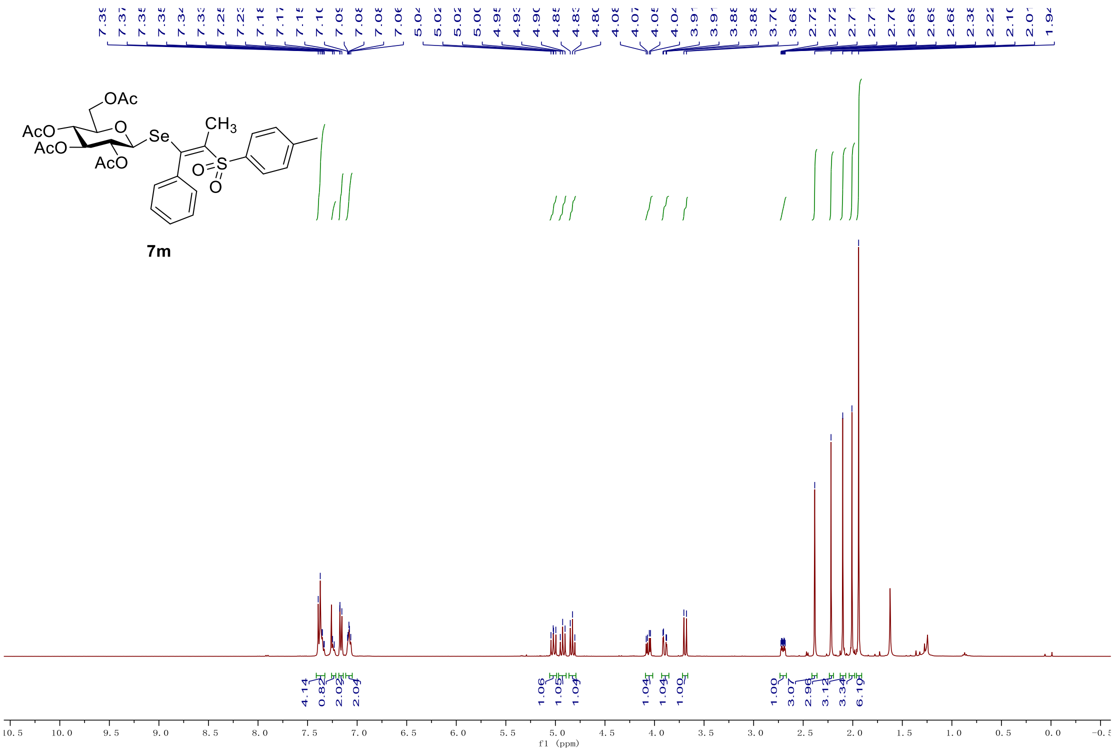

Figure S61. ^{1}H NMR (400 MHz, $CDCl_3$) Spectra for compound **7m**

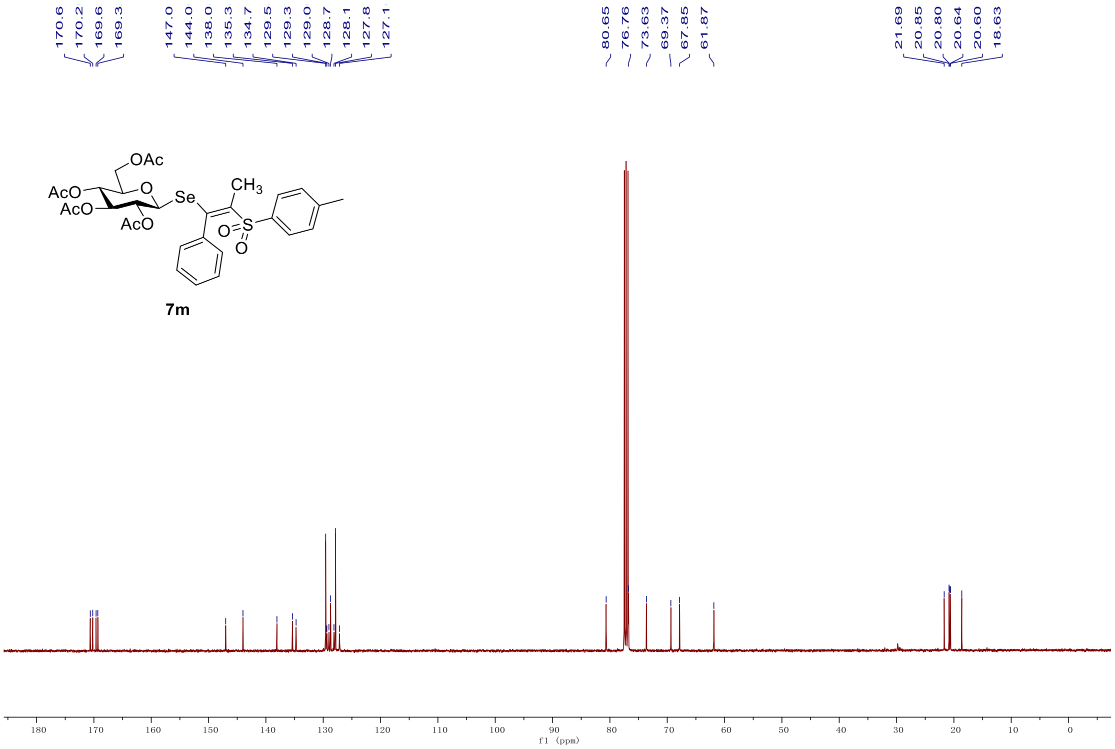

Figure S62. ^{13}C NMR (101 MHz, $CDCl_3$) Spectra for compound **7m**

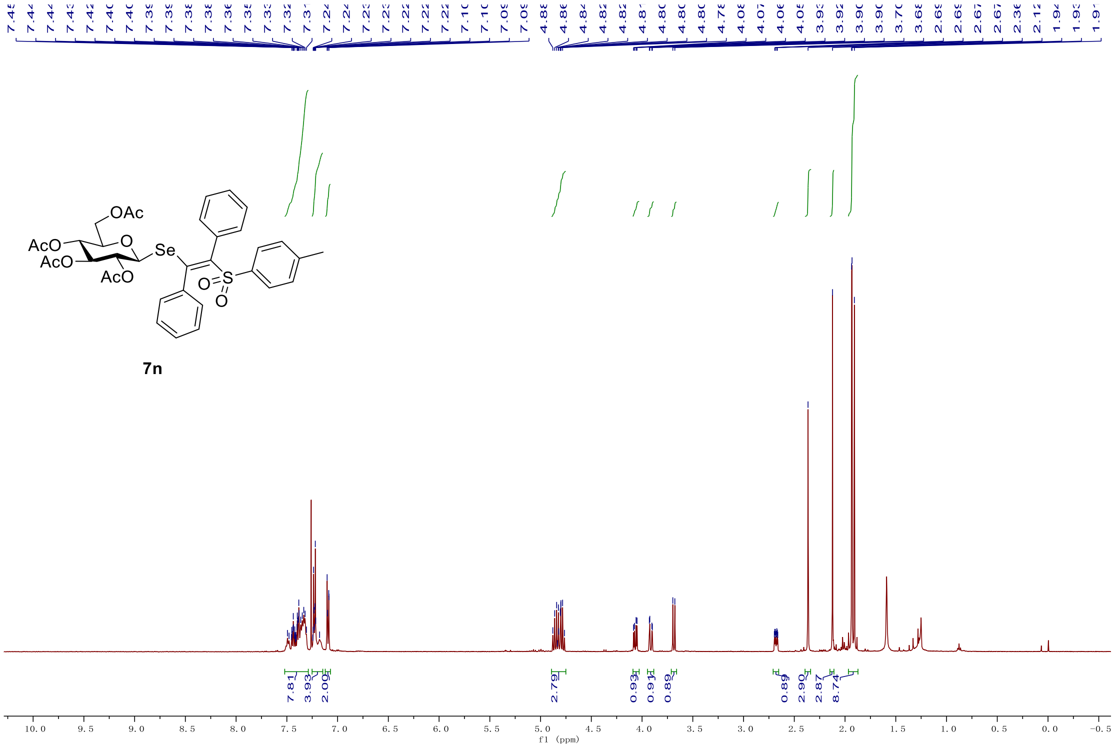

Figure S63. ^{1}H NMR (400 MHz, $CDCl_3$) Spectra for compound **7n**

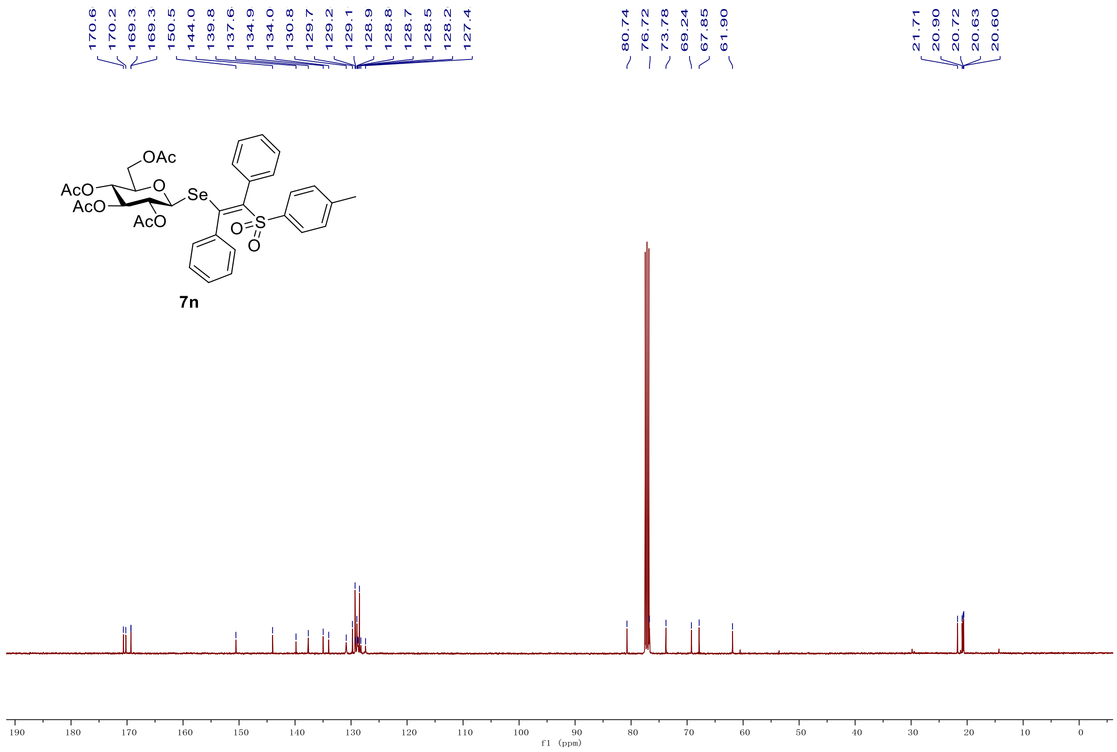

Figure S64. ^{13}C NMR (101 MHz, $CDCl_3$) Spectra for compound **7n**

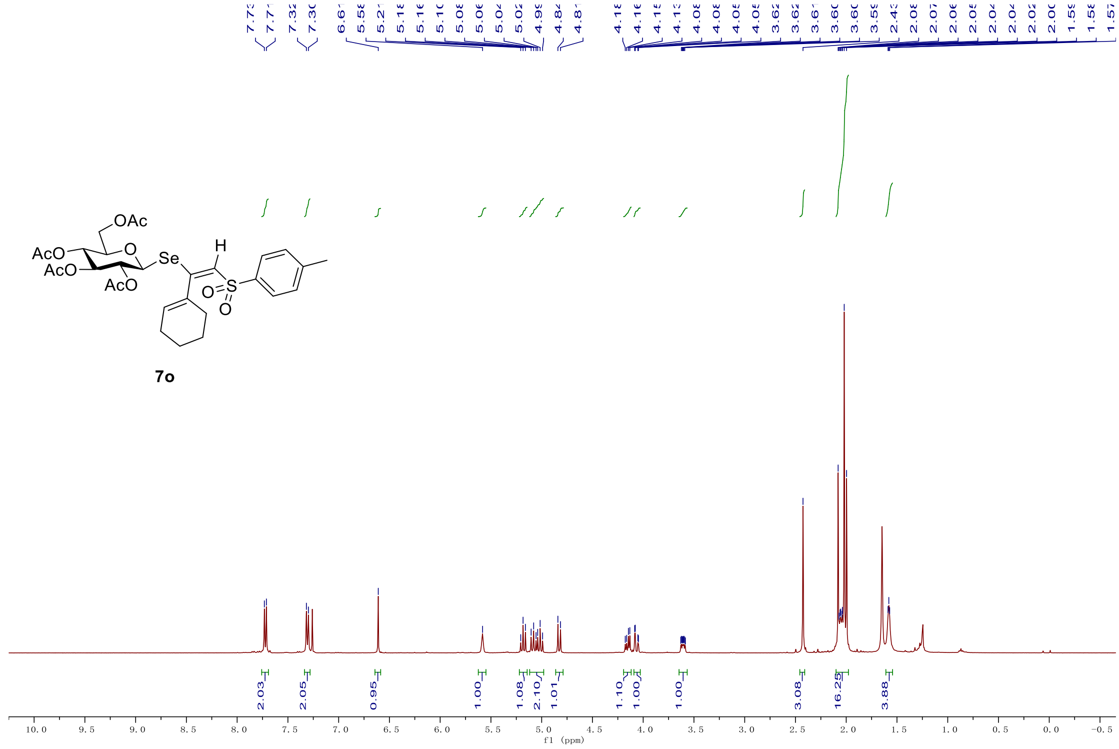

Figure S65. ^{1}H NMR (400 MHz, $CDCl_3$) Spectra for compound **7o**

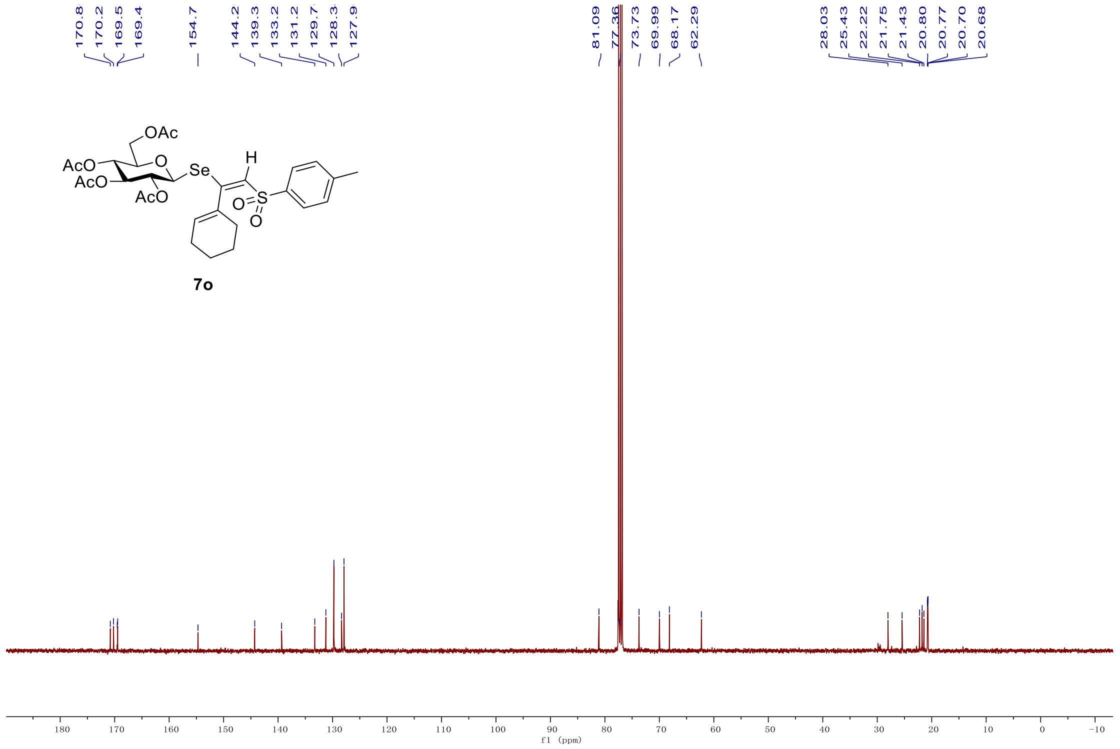

Figure S66. ^{13}C NMR (101 MHz, $CDCl_3$) Spectra for compound **7o**

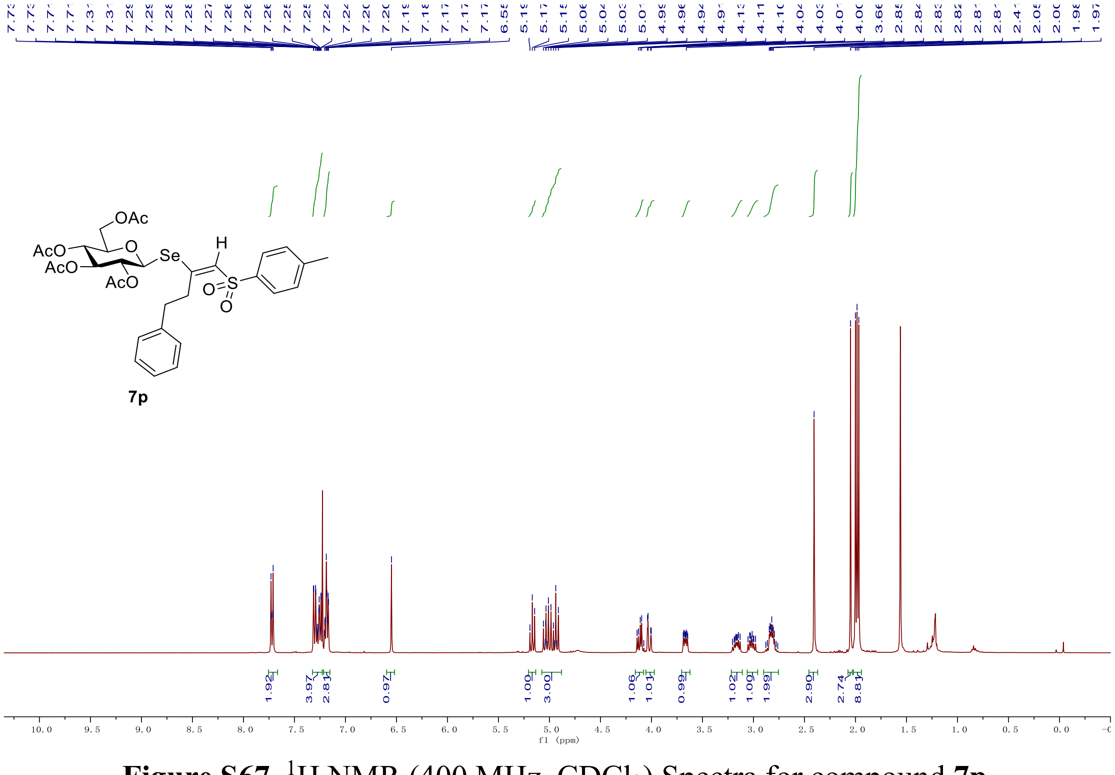

Figure S67. ^{1}H NMR (400 MHz, $CDCl_3$) Spectra for compound **7p**

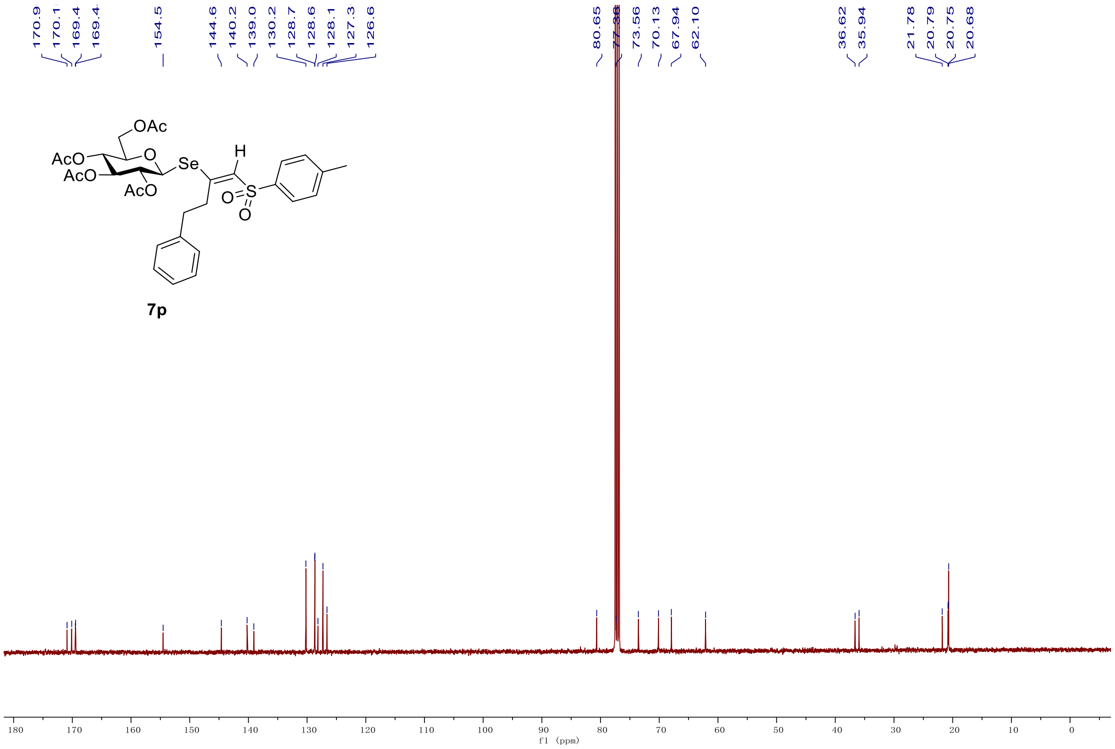

Figure S68. ^{13}C NMR (101 MHz, $CDCl_3$) Spectra for compound **7p**

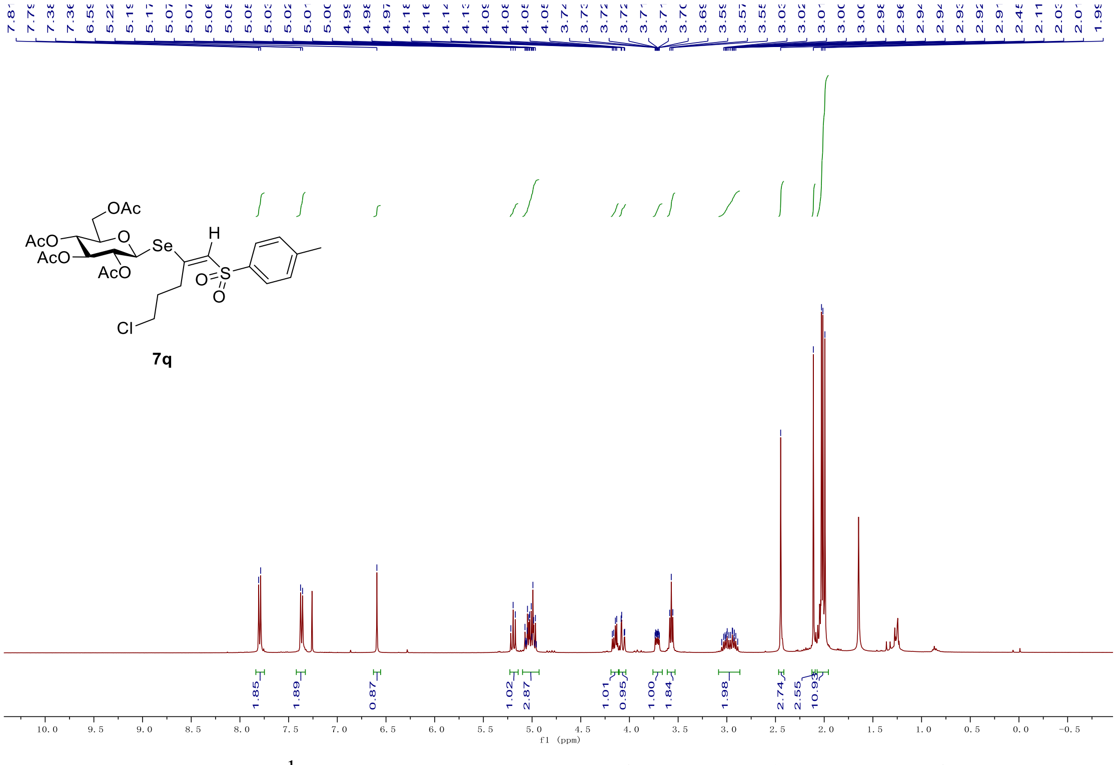

Figure S69. ^{1}H NMR (400 MHz, $CDCl_3$) Spectra for compound **7q**

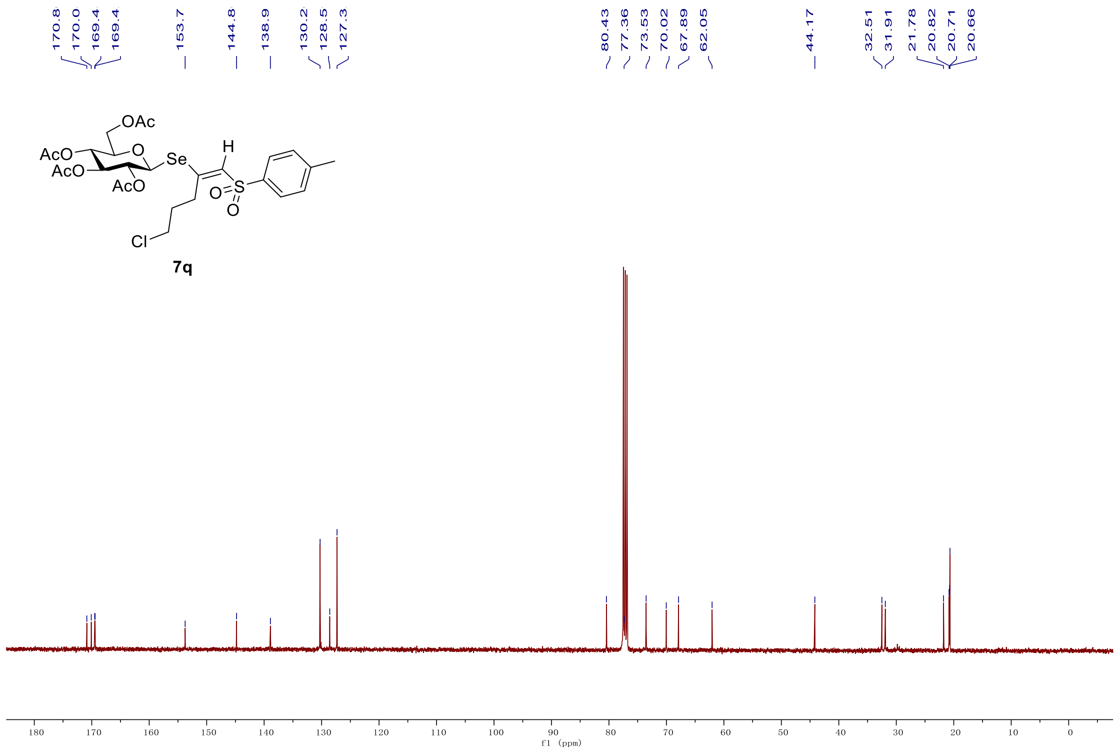

Figure S70. ^{13}C NMR (101 MHz, $CDCl_3$) Spectra for compound **7q**

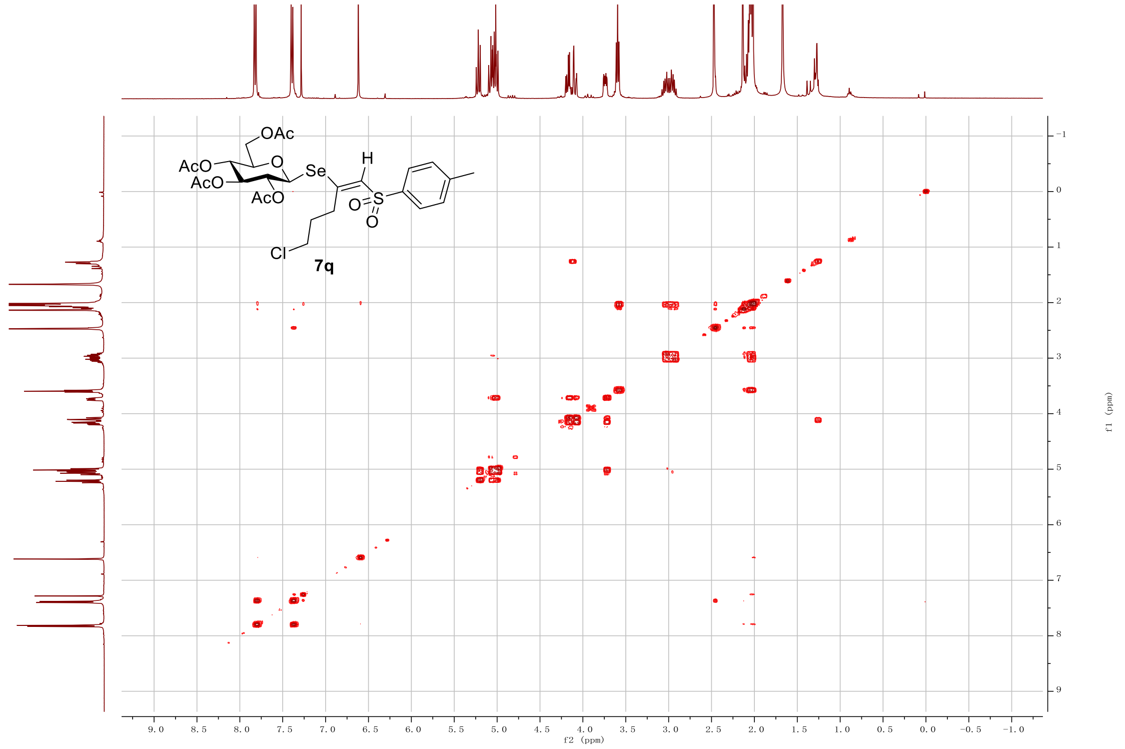

Figure S71. COSY Spectra for compound **7q**

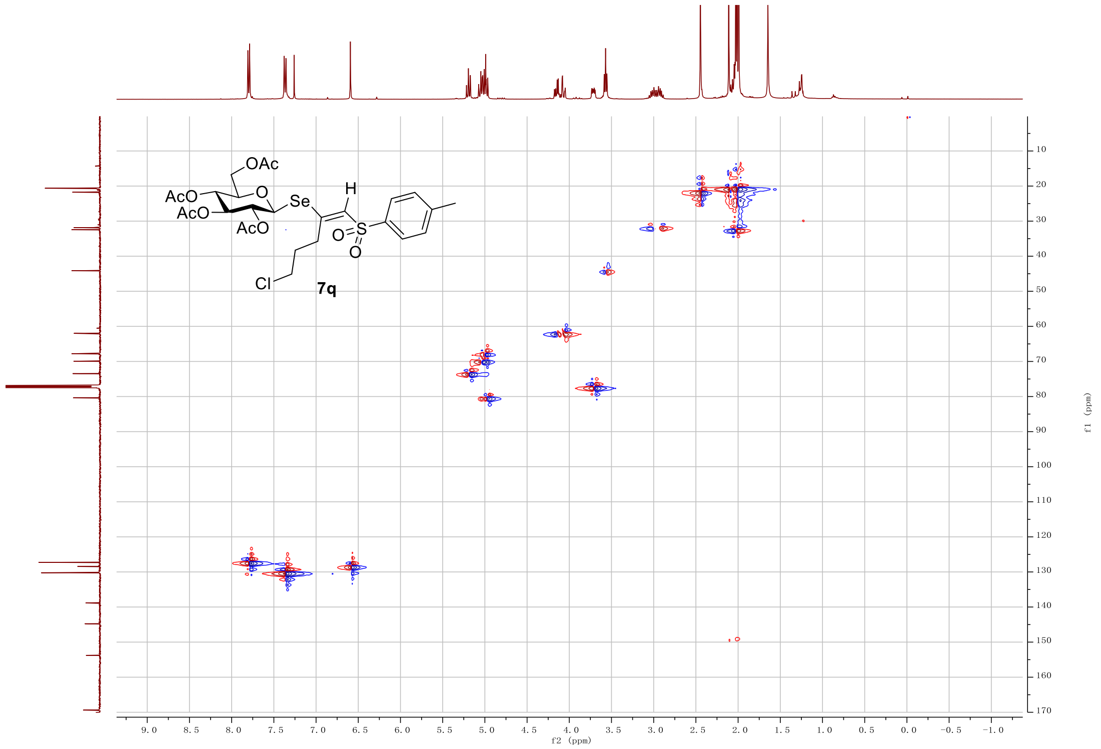

Figure S72. HSQC Spectra for compound **7q**

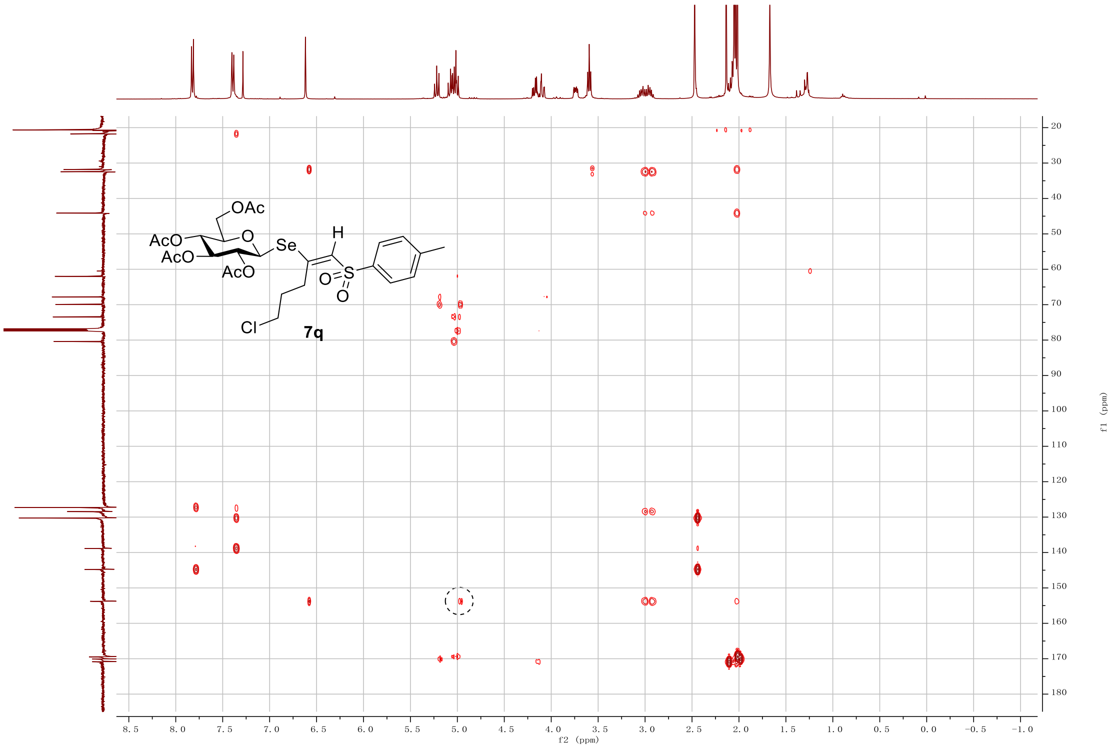

Figure S73. HMBC Spectra for compound **7q**

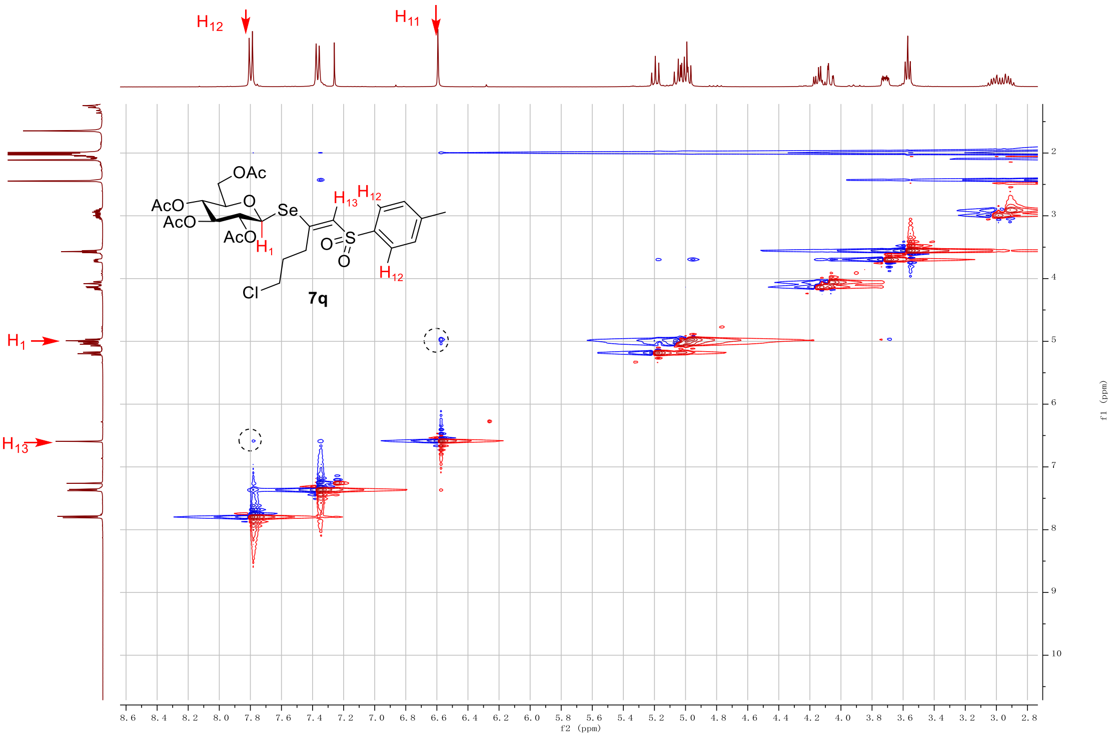

Figure S74. NOESY Spectra for compound **7q**

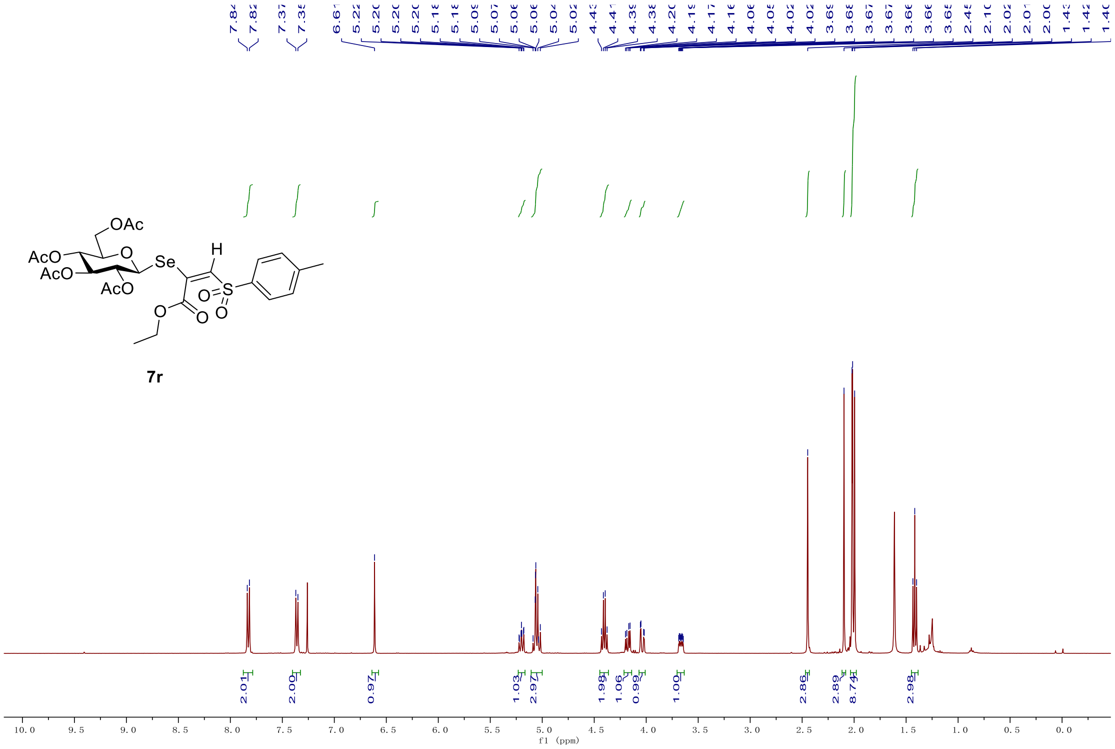

Figure S75. ^{1}H NMR (400 MHz, $CDCl_3$) Spectra for compound **7r**

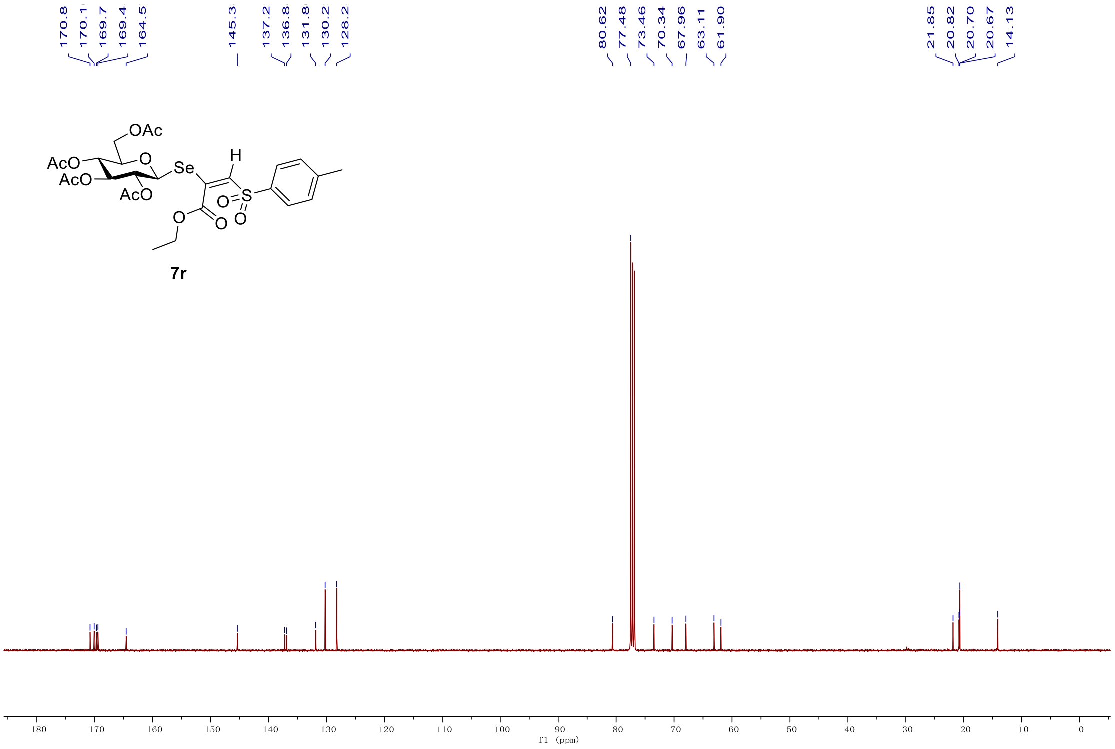

Figure S76. ^{13}C NMR (101 MHz, $CDCl_3$) Spectra for compound **7r**

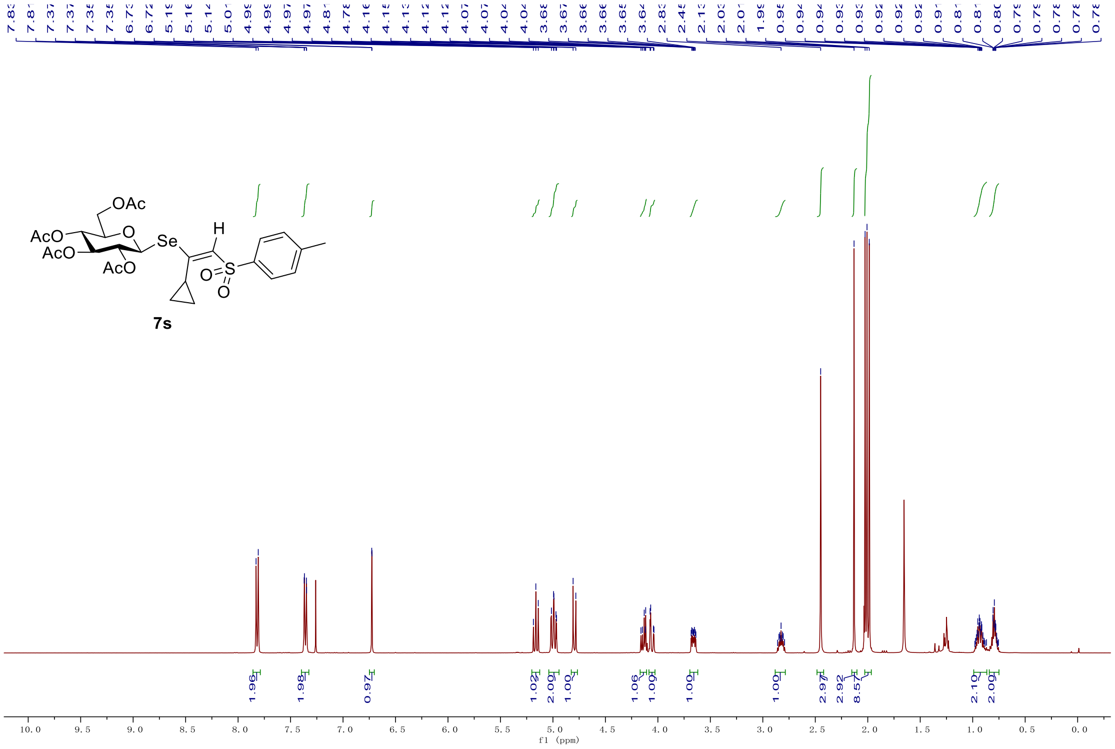

Figure S77. ^{1}H NMR (400 MHz, $CDCl_3$) Spectra for compound **7s**

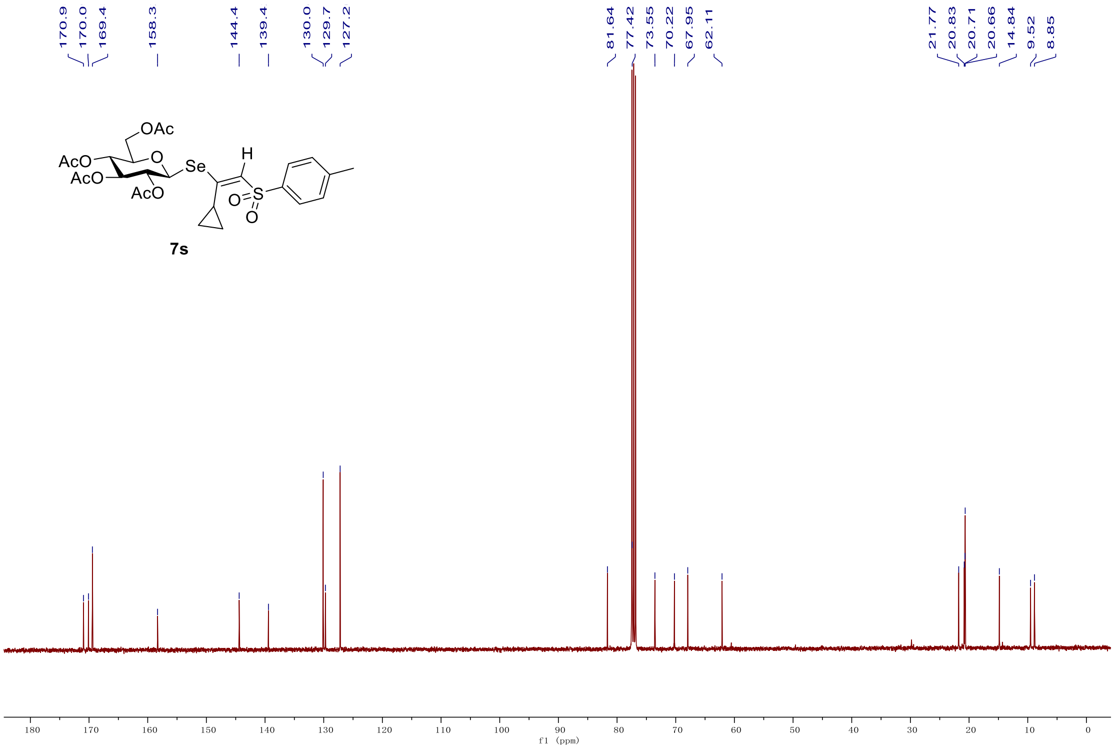

Figure S78. ^{13}C NMR (101 MHz, $CDCl_3$) Spectra for compound **7s**

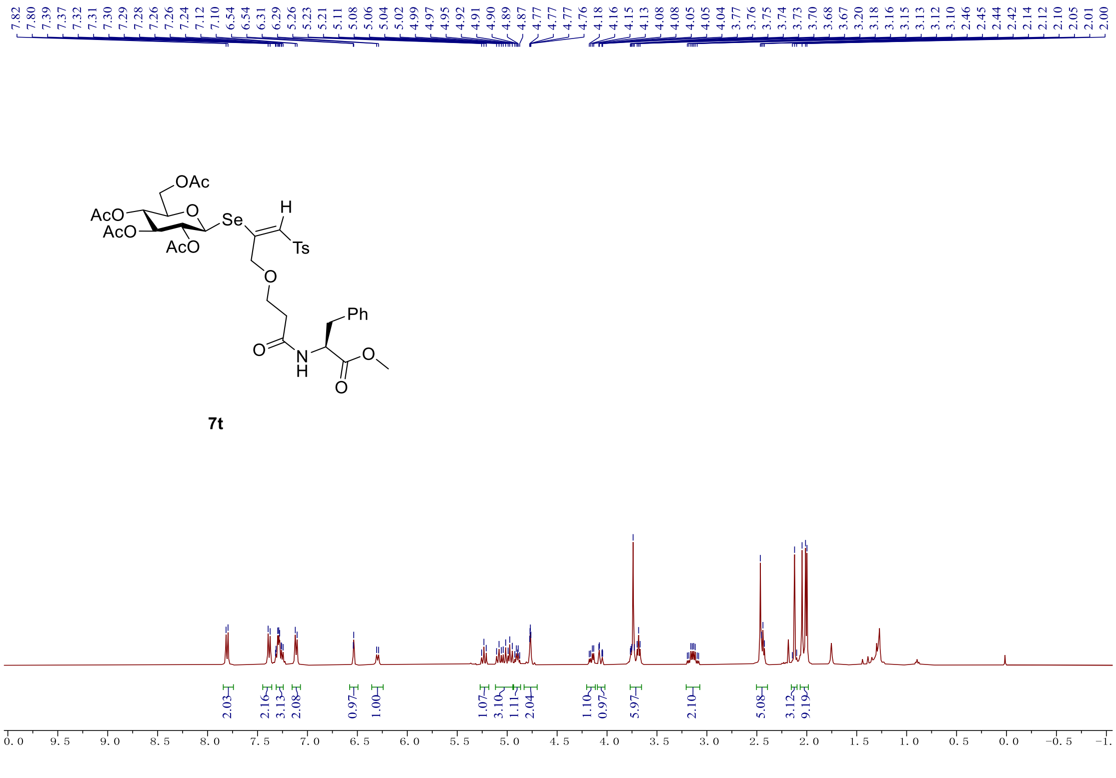

Figure S79. ^{1}H NMR (400 MHz, $CDCl_3$) Spectra for compound **7t**

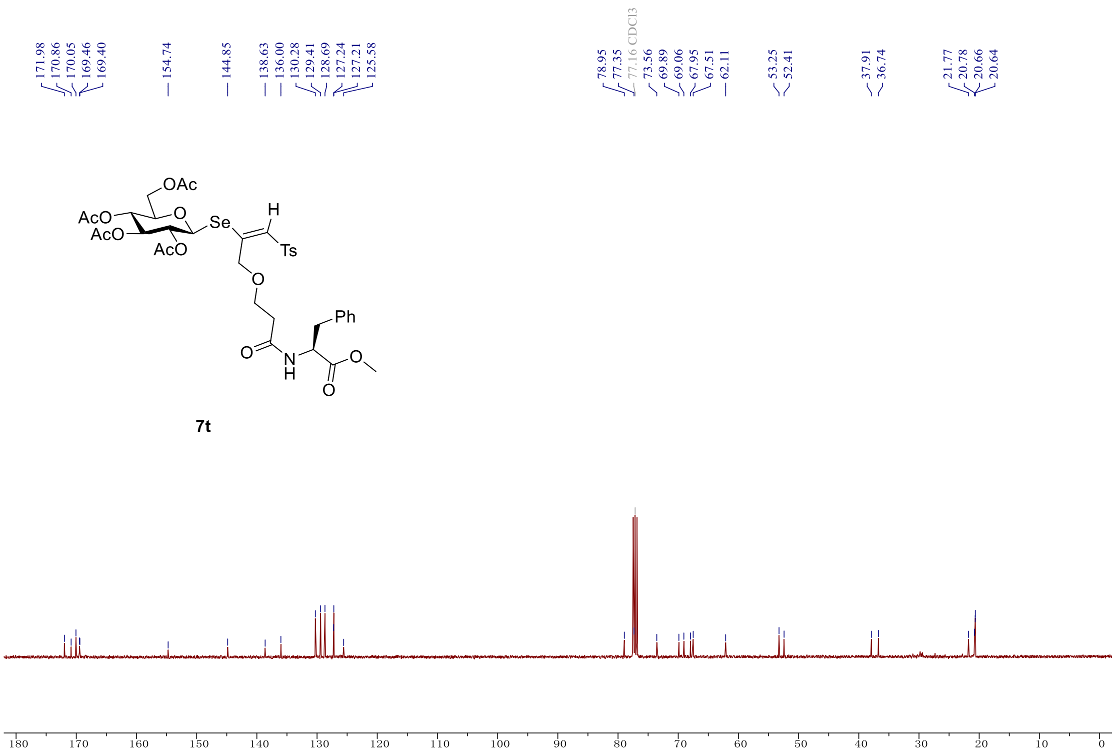

Figure S80. ^{13}C NMR (101 MHz, $CDCl_3$) Spectra for compound **7t**

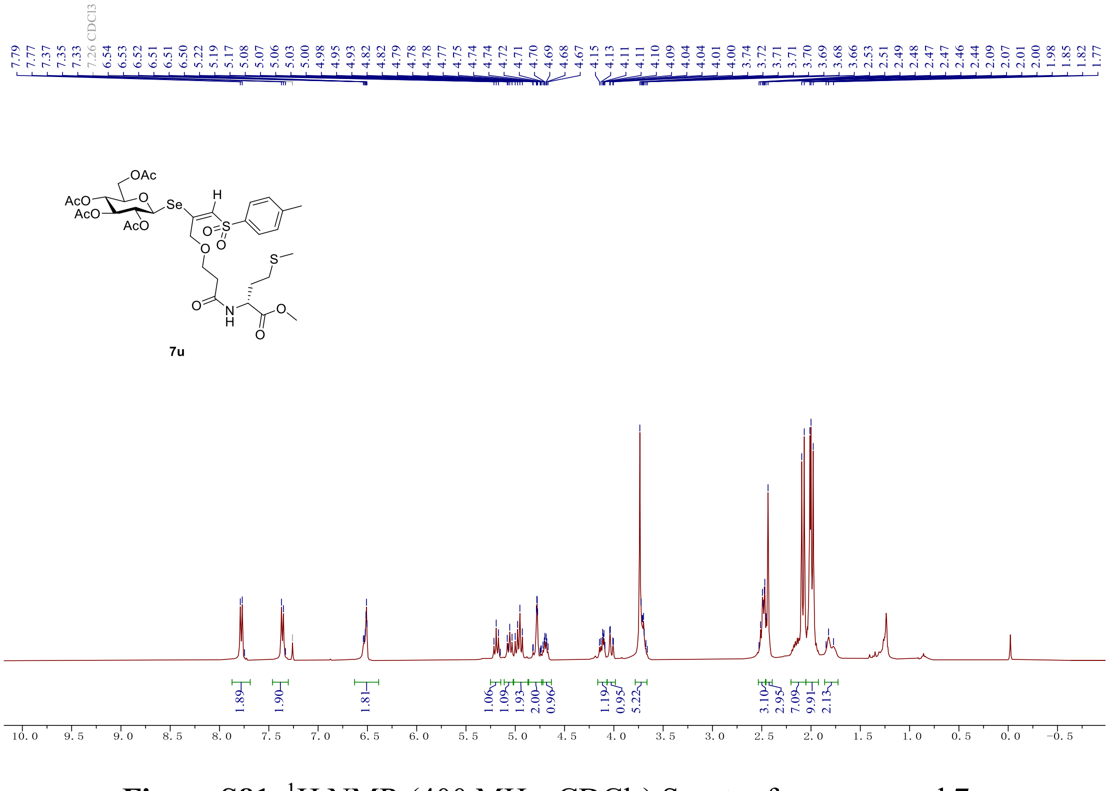

Figure S81. ^{1}H NMR (400 MHz, $CDCl_3$) Spectra for compound **7u**

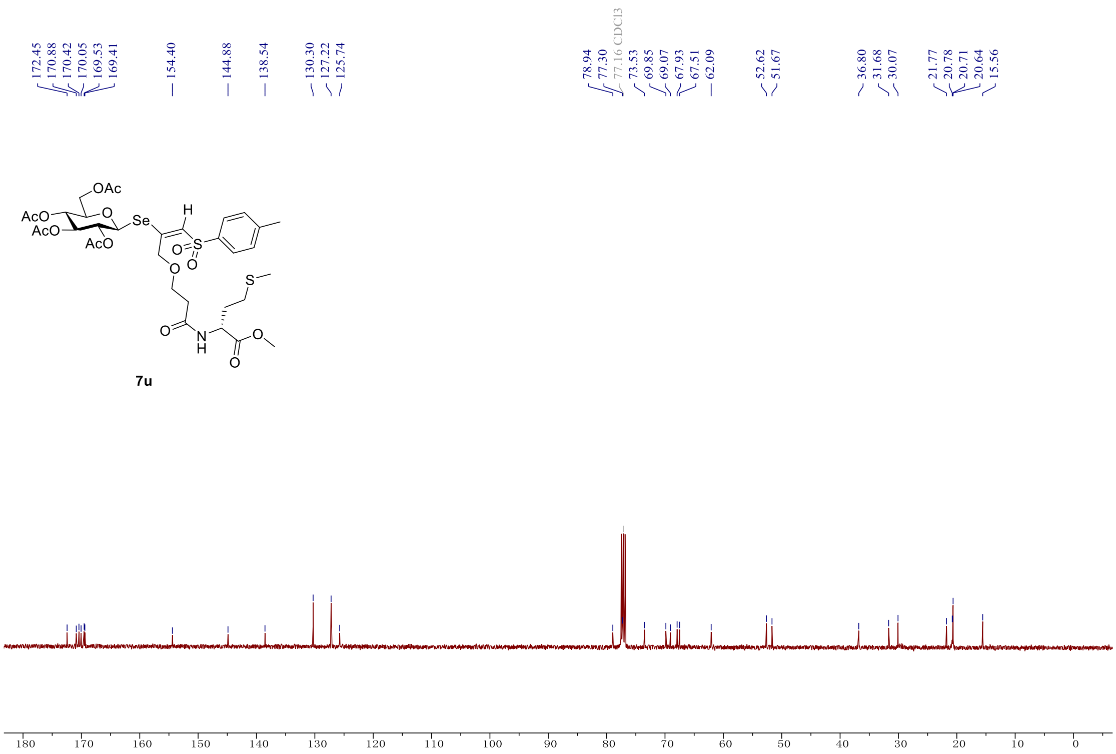

Figure S82. ^{13}C NMR (101 MHz, $CDCl_3$) Spectra for compound **7u**

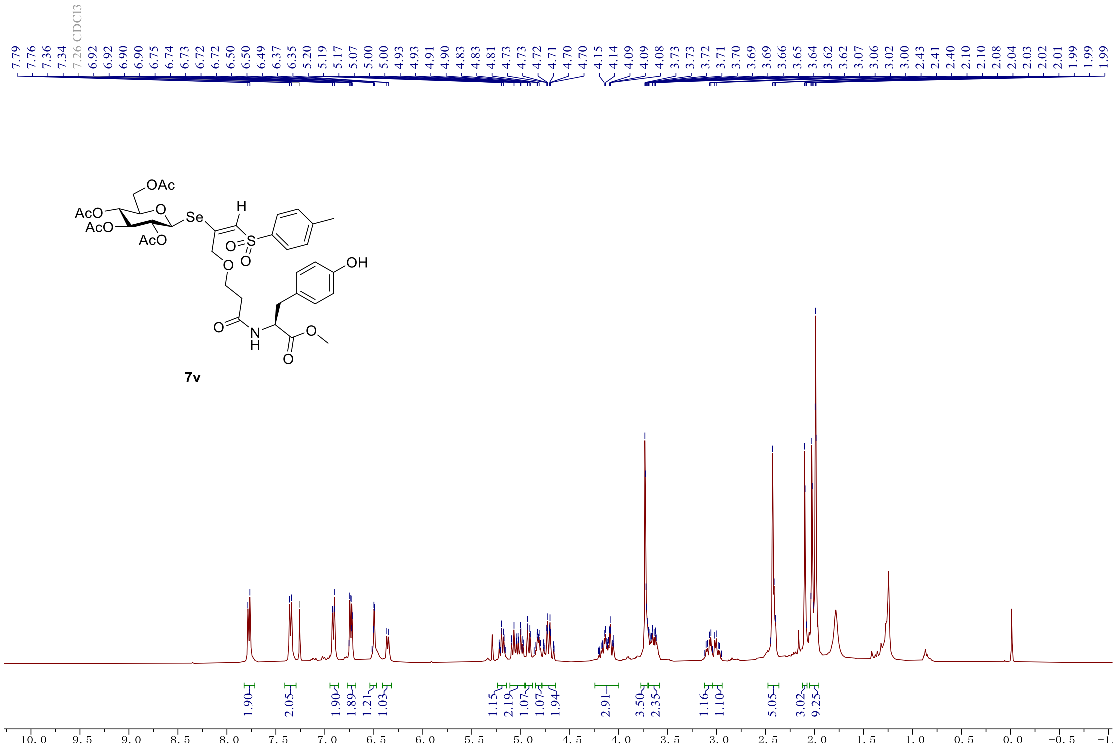

Figure S83. ^{1}H NMR (400 MHz, $CDCl_3$) Spectra for compound **7v**

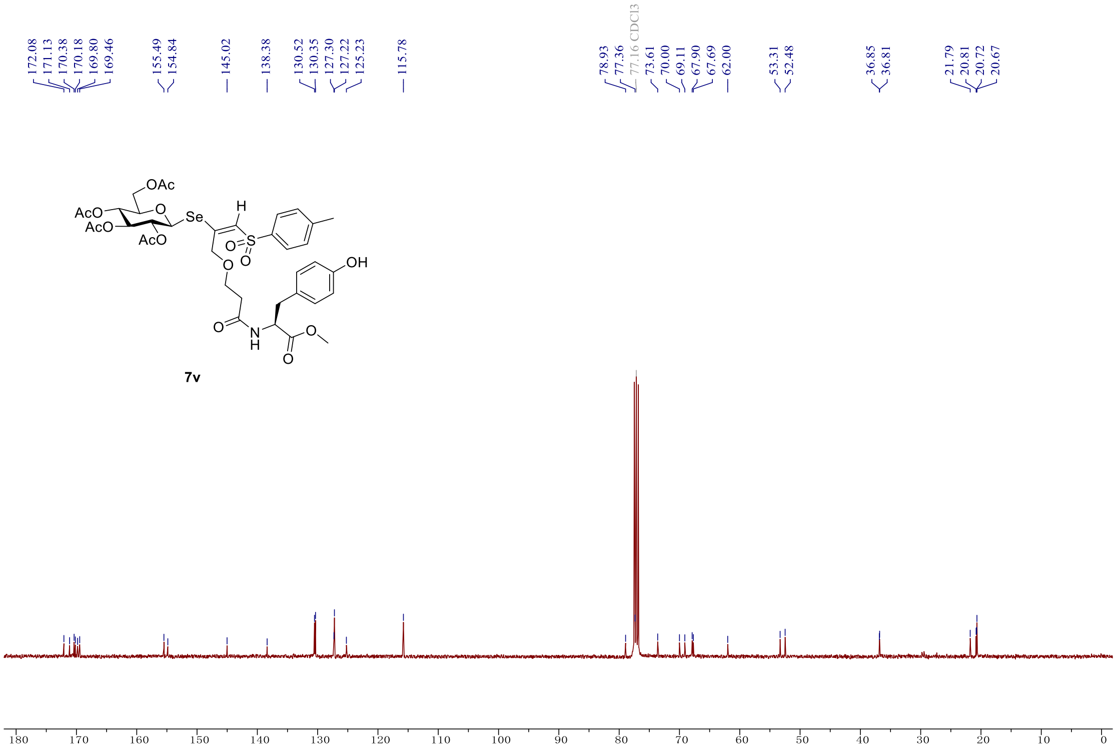

Figure S84. ^{13}C NMR (101 MHz, CDCl$_3$) Spectra for compound **7v**

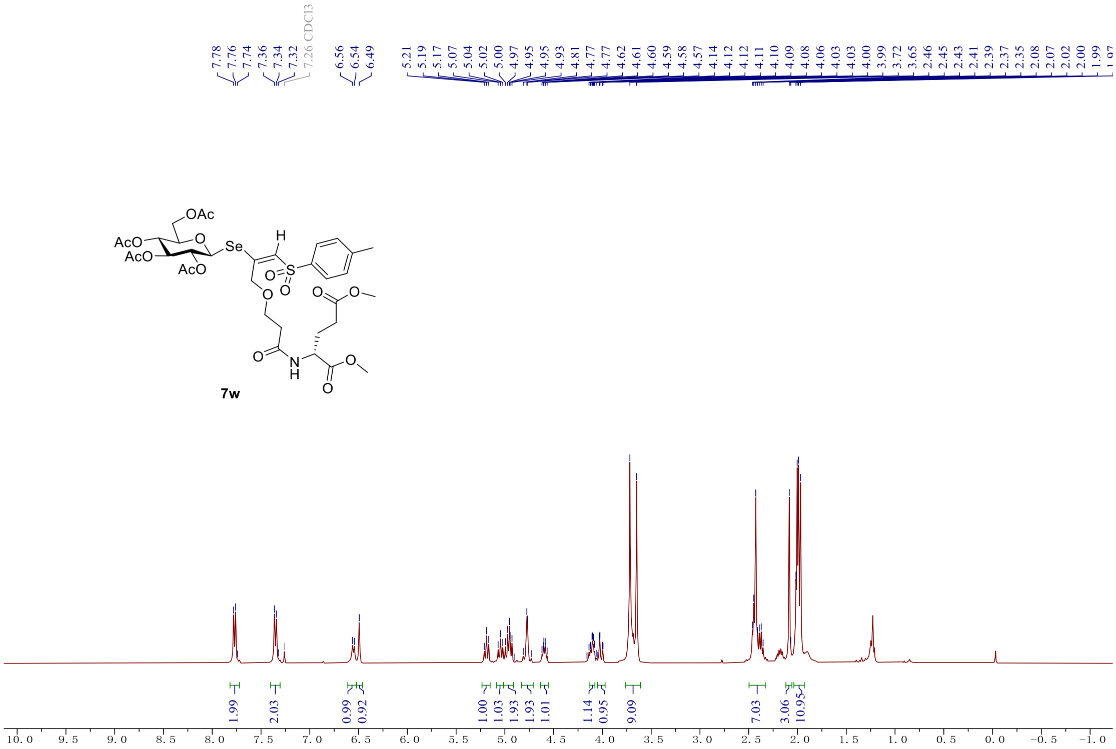

Figure S85. ^{1}H NMR (400 MHz, CDCl$_3$) Spectra for compound **7w**

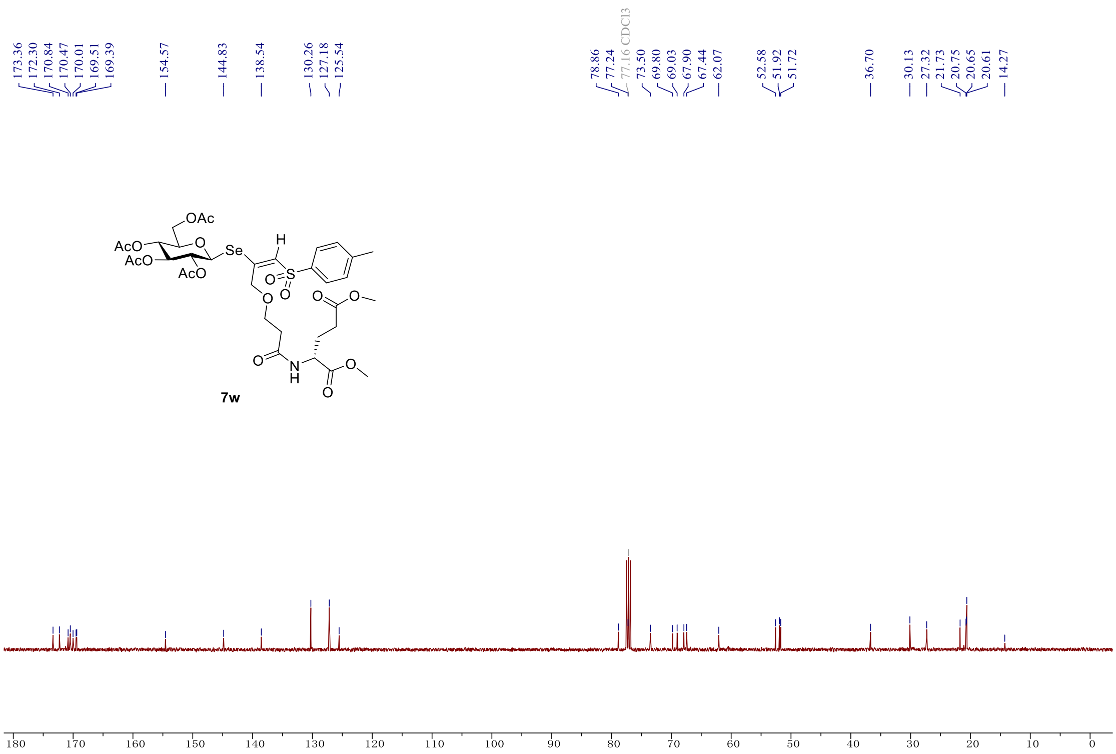

Figure S86. ^{13}C NMR (101 MHz, $CDCl_3$) Spectra for compound **7w**

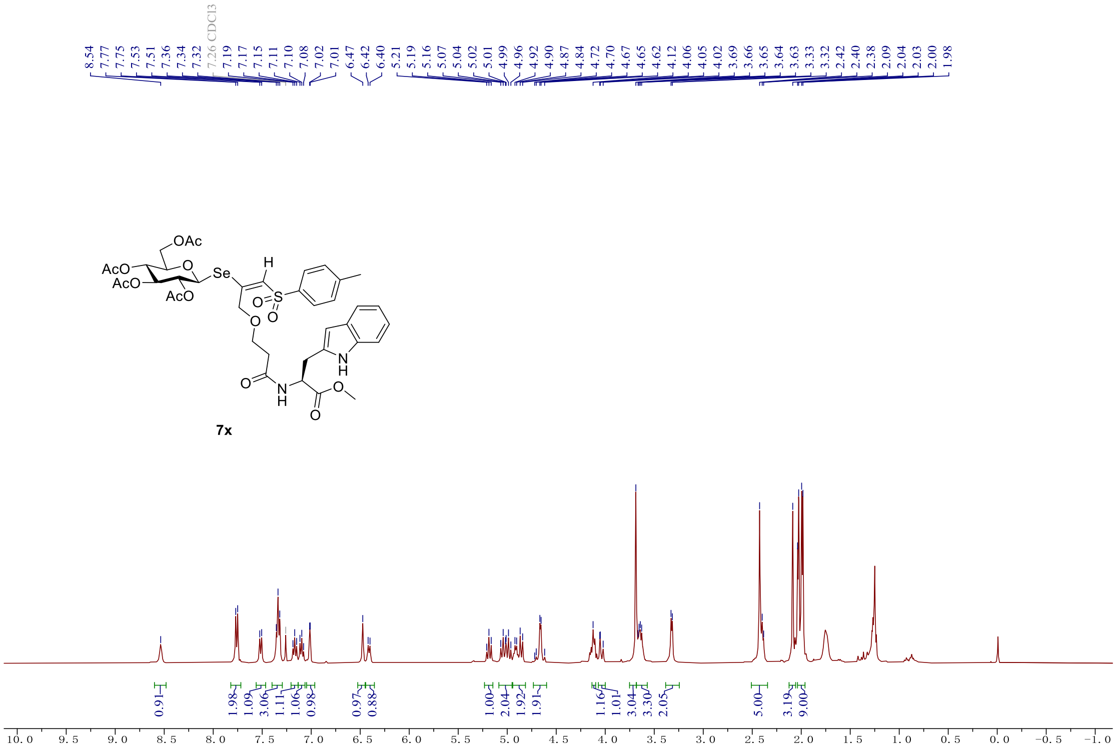

Figure S87. ^{1}H NMR (400 MHz, $CDCl_3$) Spectra for compound **7x**

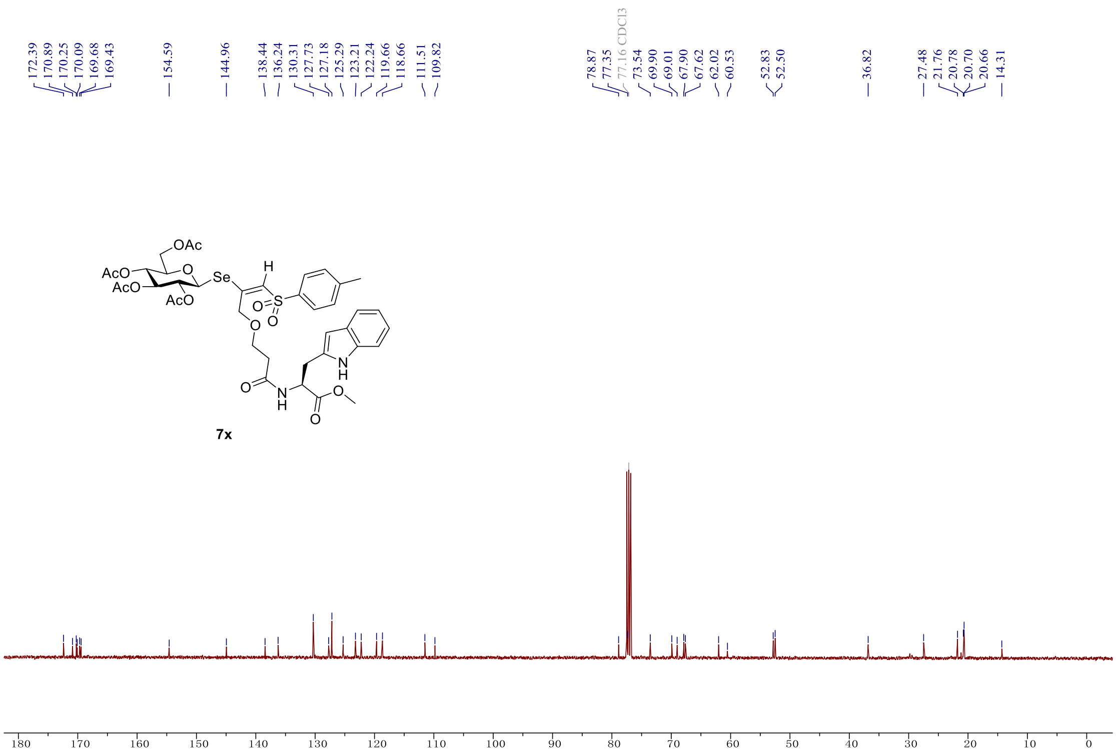

Figure S88. ^{13}C NMR (101 MHz, $CDCl_3$) Spectra for compound **7x**

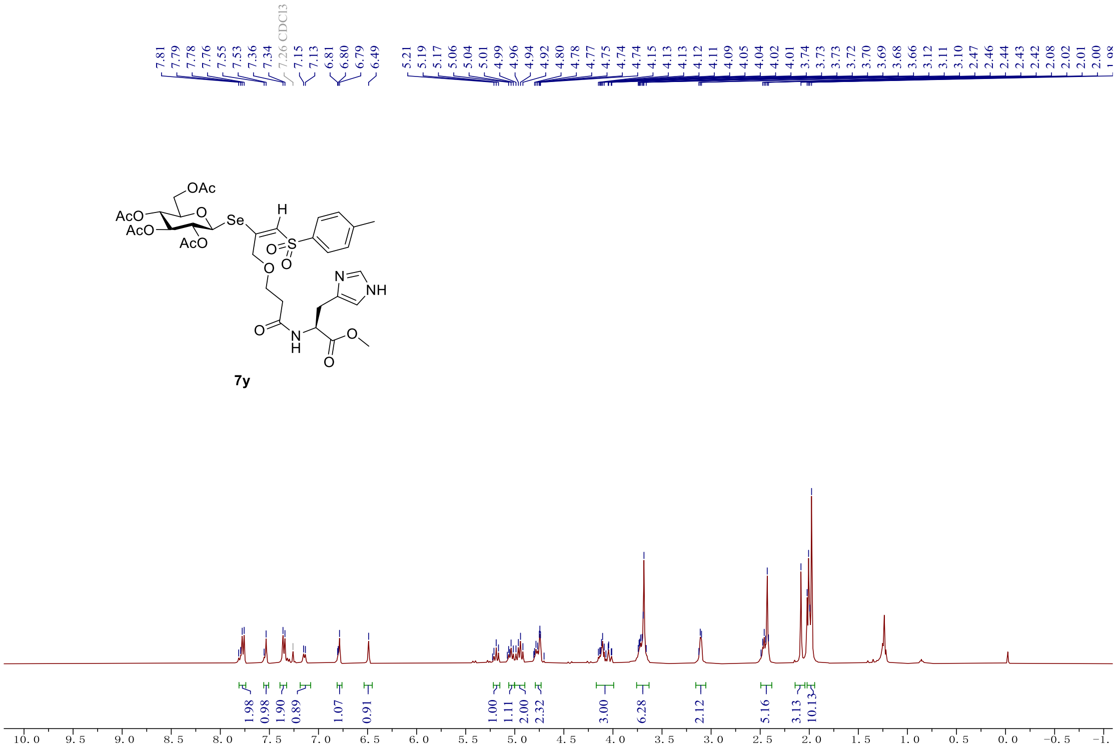

Figure S89. ^{1}H NMR (400 MHz, $CDCl_3$) Spectra for compound **7y**

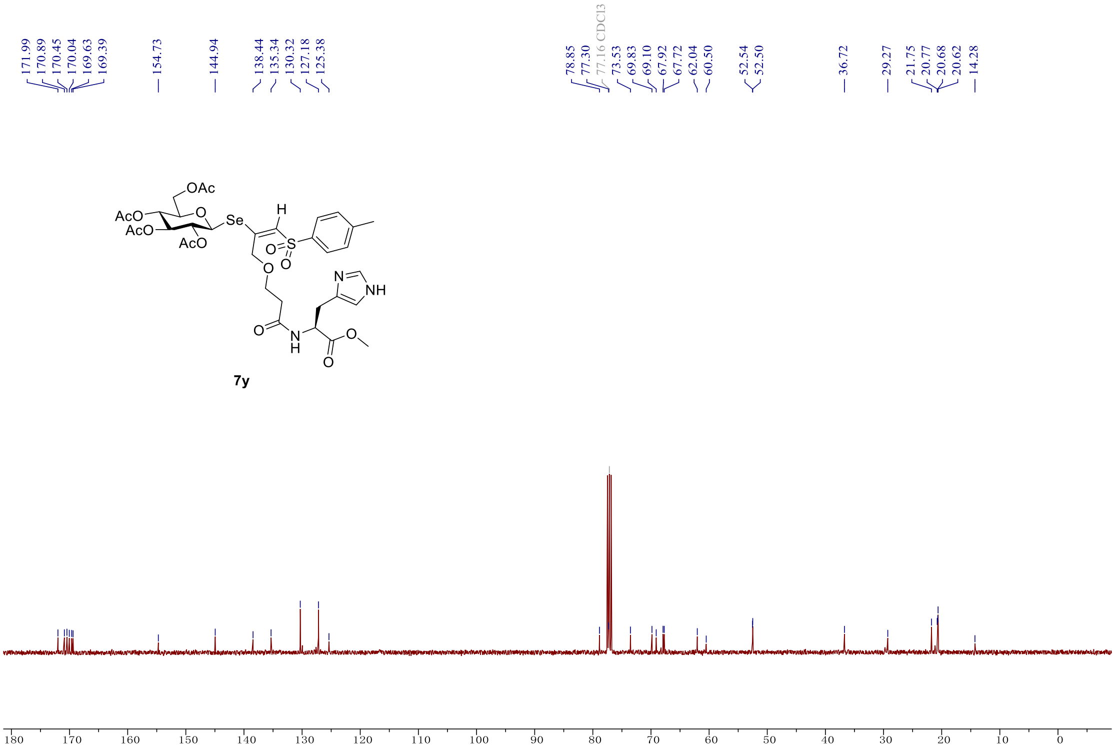

Figure S90. ^{13}C NMR (101 MHz, $CDCl_3$) Spectra for compound **7y**

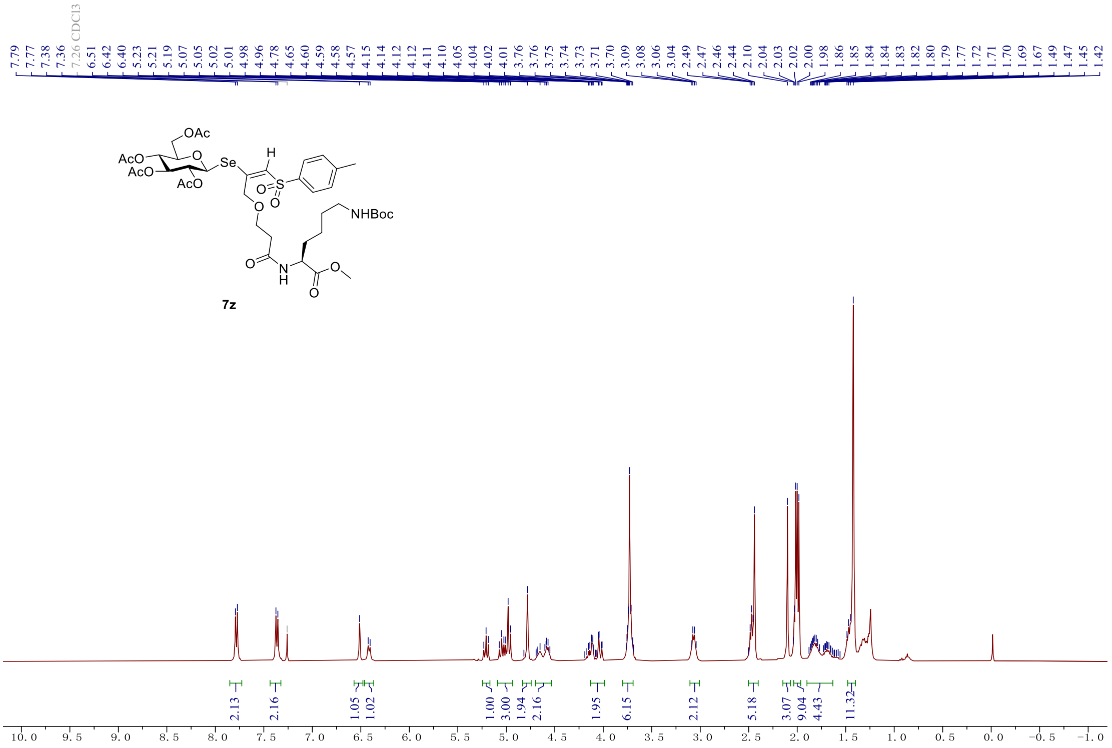

Figure S91. ^{1}H NMR (400 MHz, $CDCl_3$) Spectra for compound **7z**

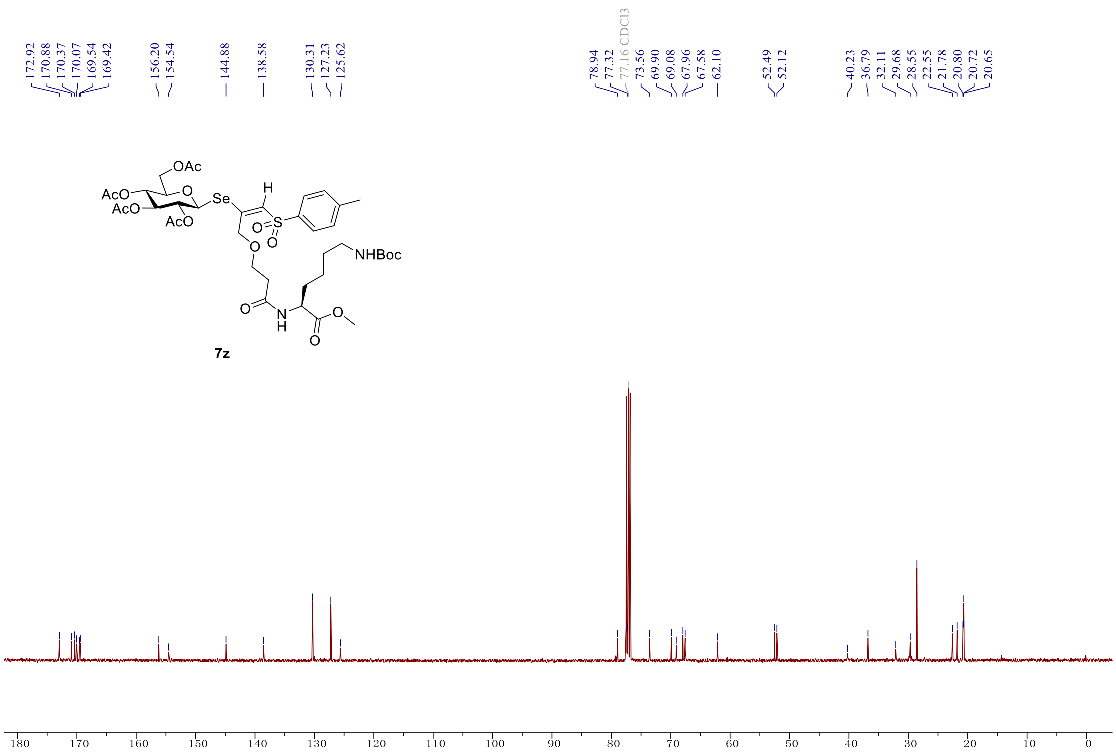

Figure S92. ^{13}C NMR (101 MHz, $CDCl_3$) Spectra for compound **7z**

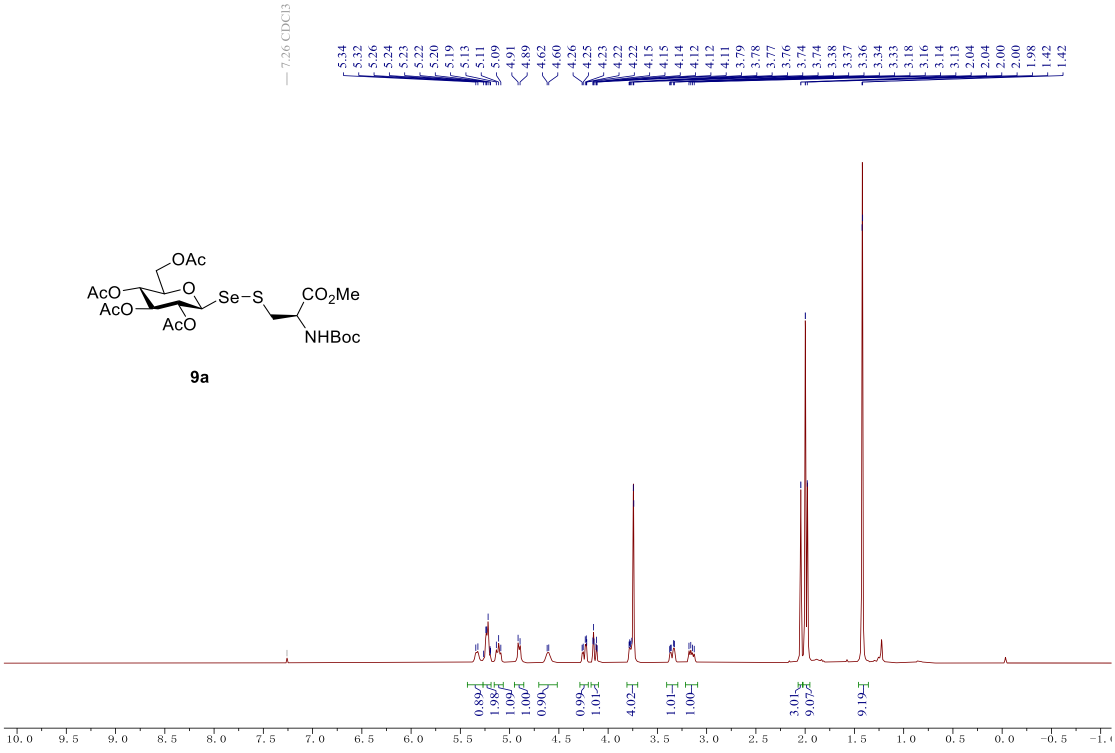

Figure S93. ^{1}H NMR (400 MHz, $CDCl_3$) Spectra for compound **9a**

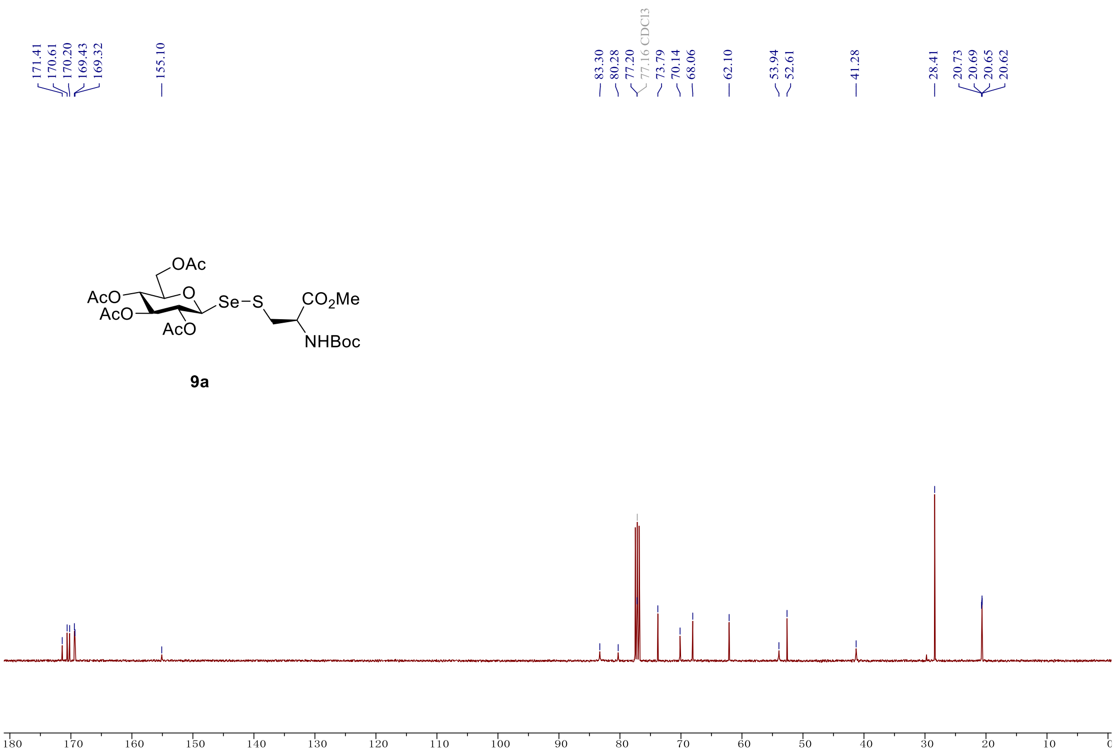

Figure S94. ^{13}C NMR (101 MHz, $CDCl_3$) Spectra for compound **9a**

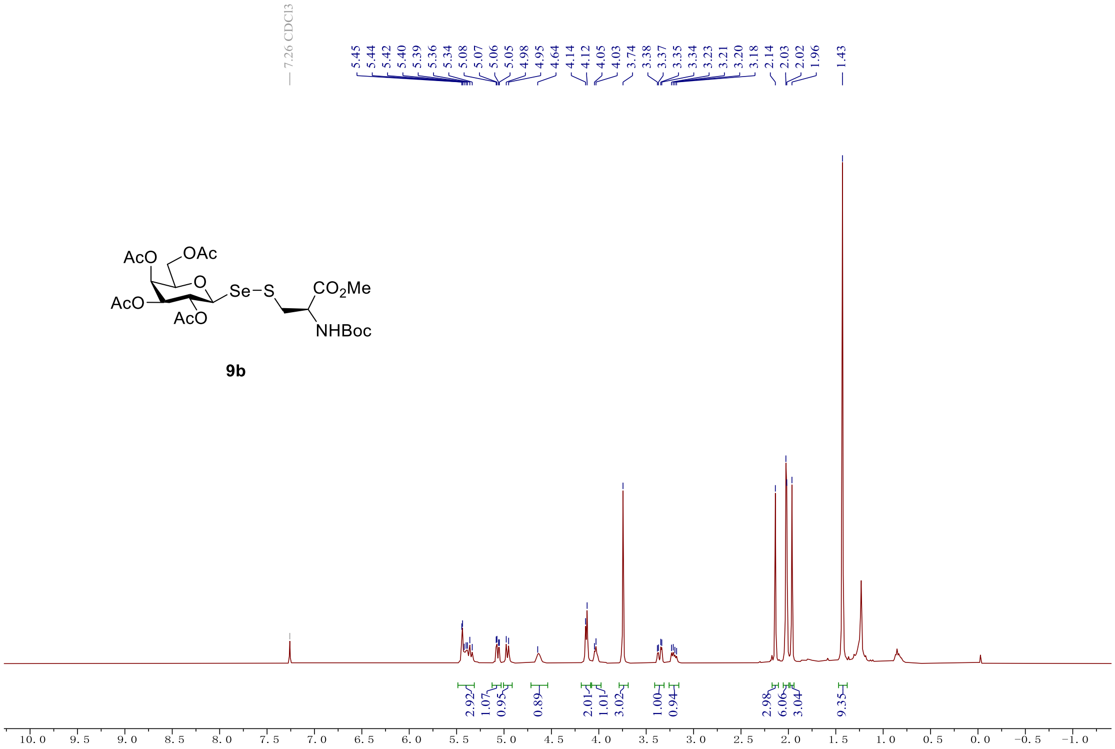

Figure S95. ^{1}H NMR (400 MHz, $CDCl_3$) Spectra for compound **9b**

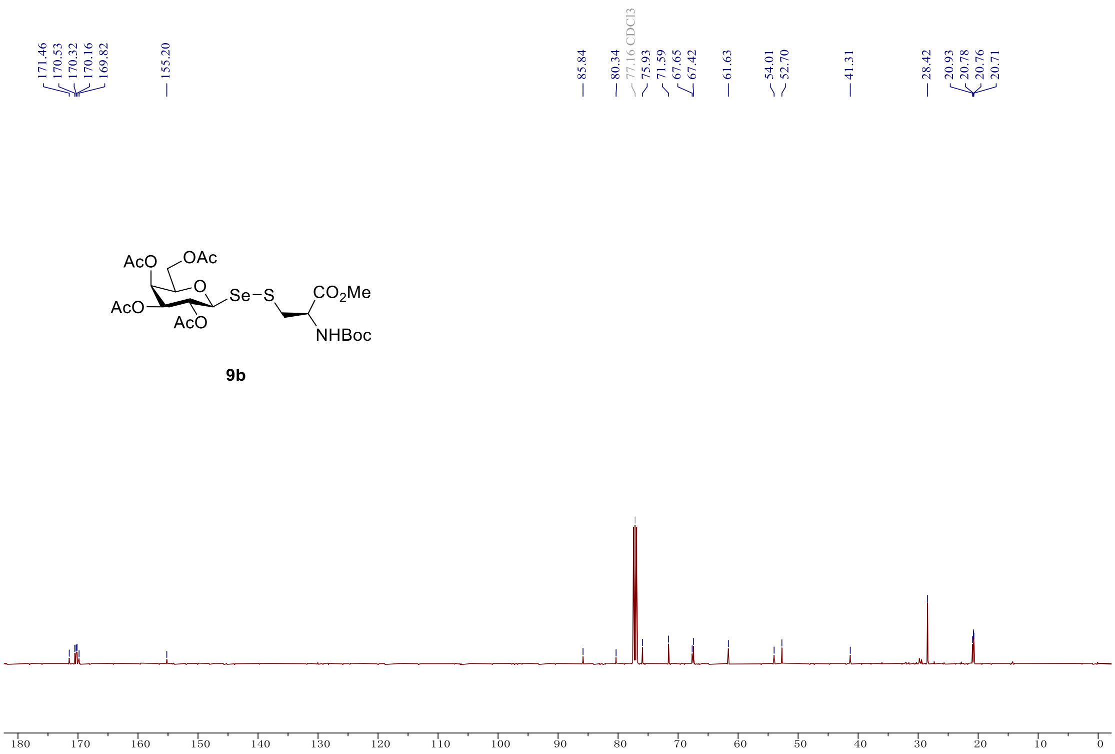

Figure S96. ^{13}C NMR (101 MHz, $CDCl_3$) Spectra for compound **9b**

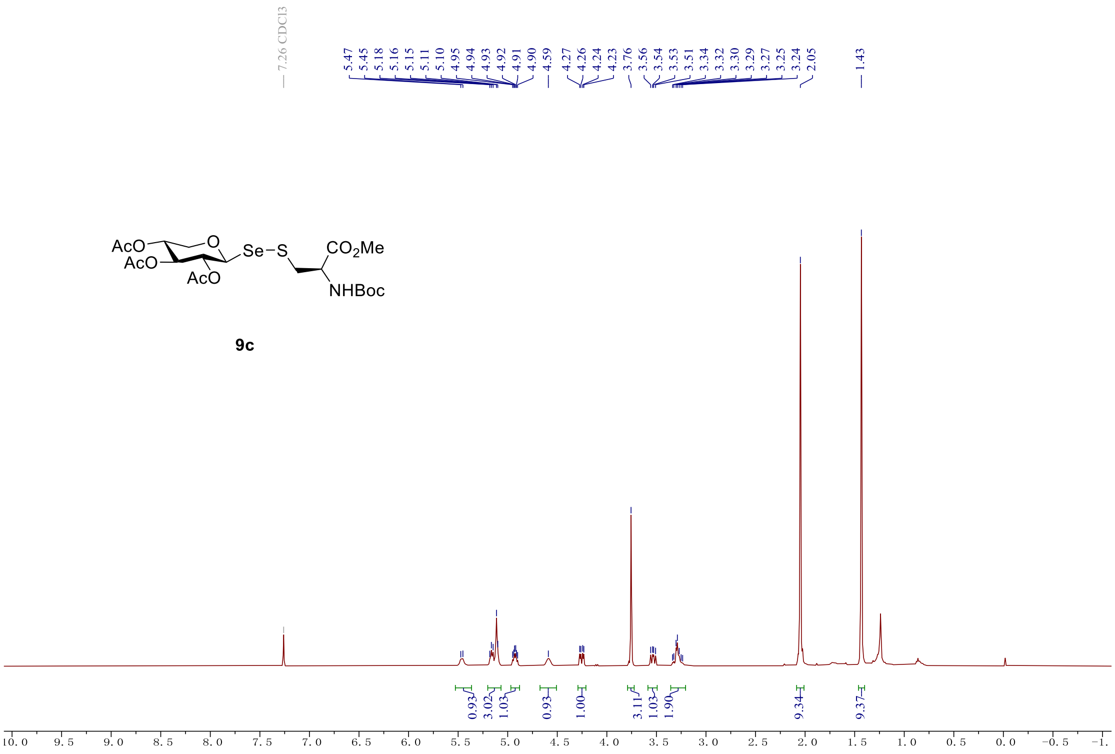

Figure S97. ^{1}H NMR (400 MHz, $CDCl_3$) Spectra for compound **9c**

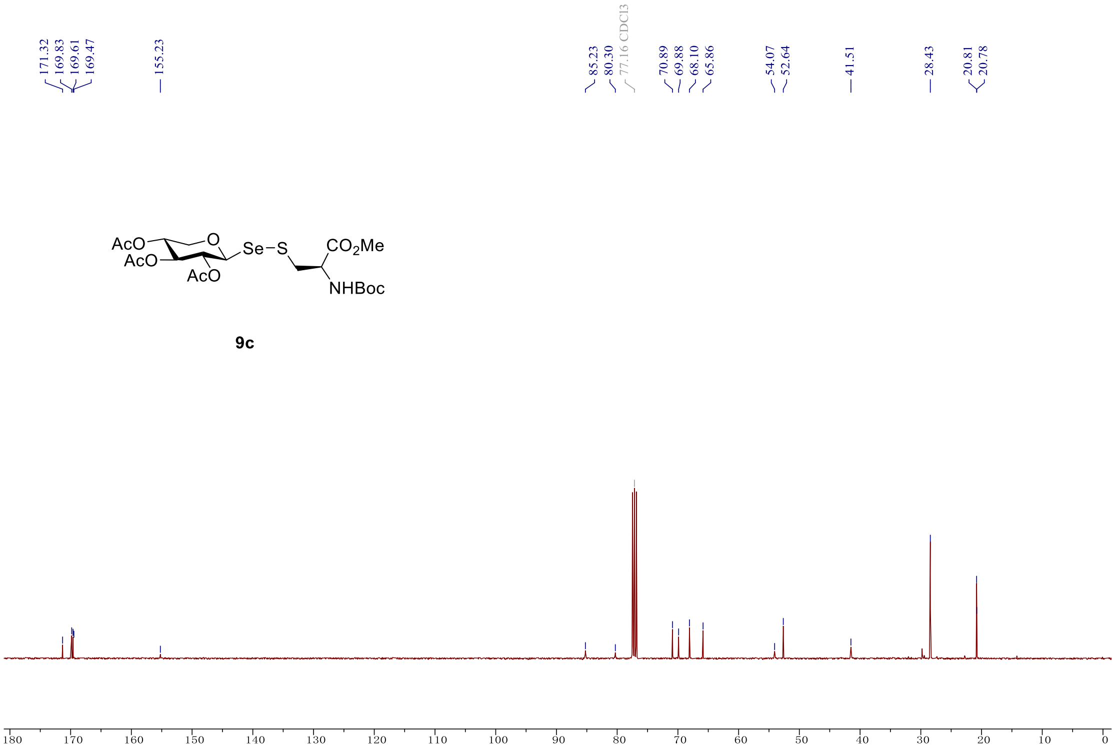

Figure S98. ^{13}C NMR (101 MHz, $CDCl_3$) Spectra for compound **9c**

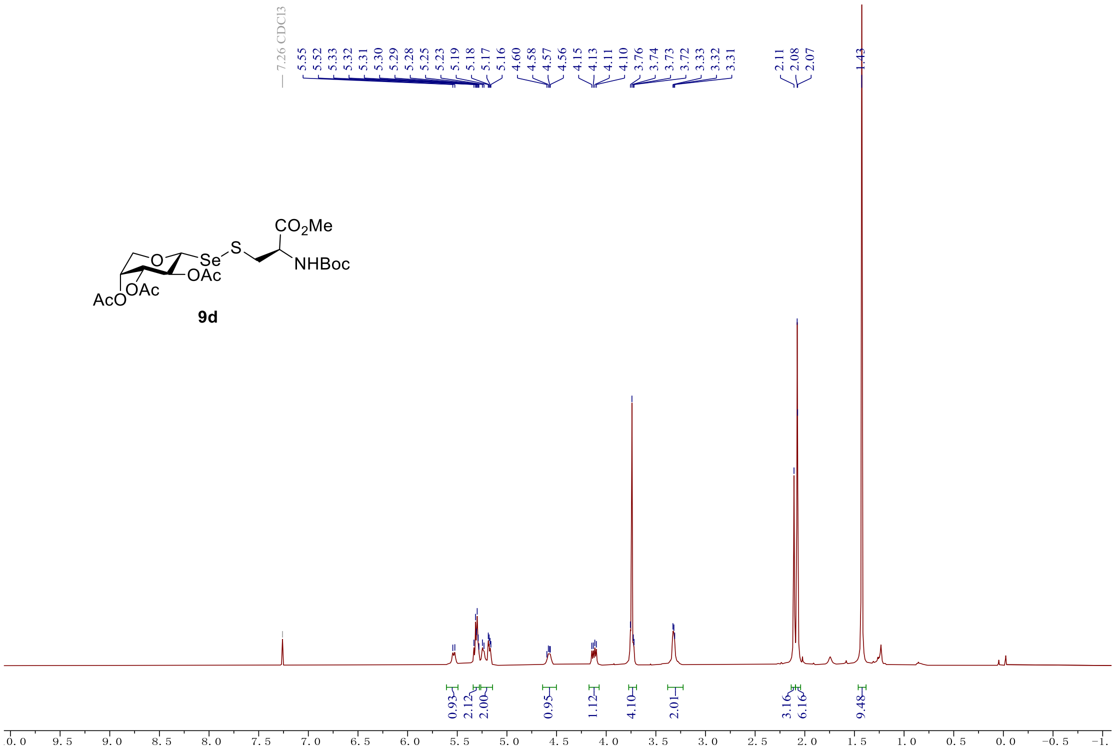

Figure S99. ^{1}H NMR (400 MHz, $CDCl_3$) Spectra for compound **9d**

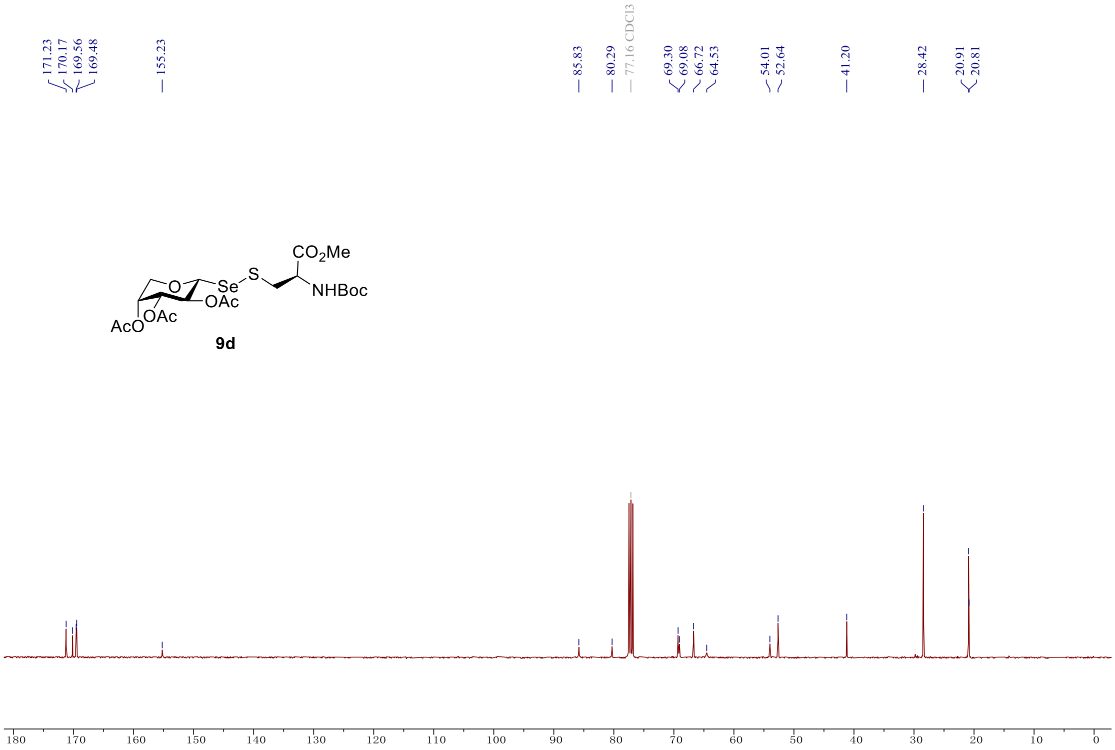

Figure S100. ^{13}C NMR (101 MHz, $CDCl_3$) Spectra for compound **9d**

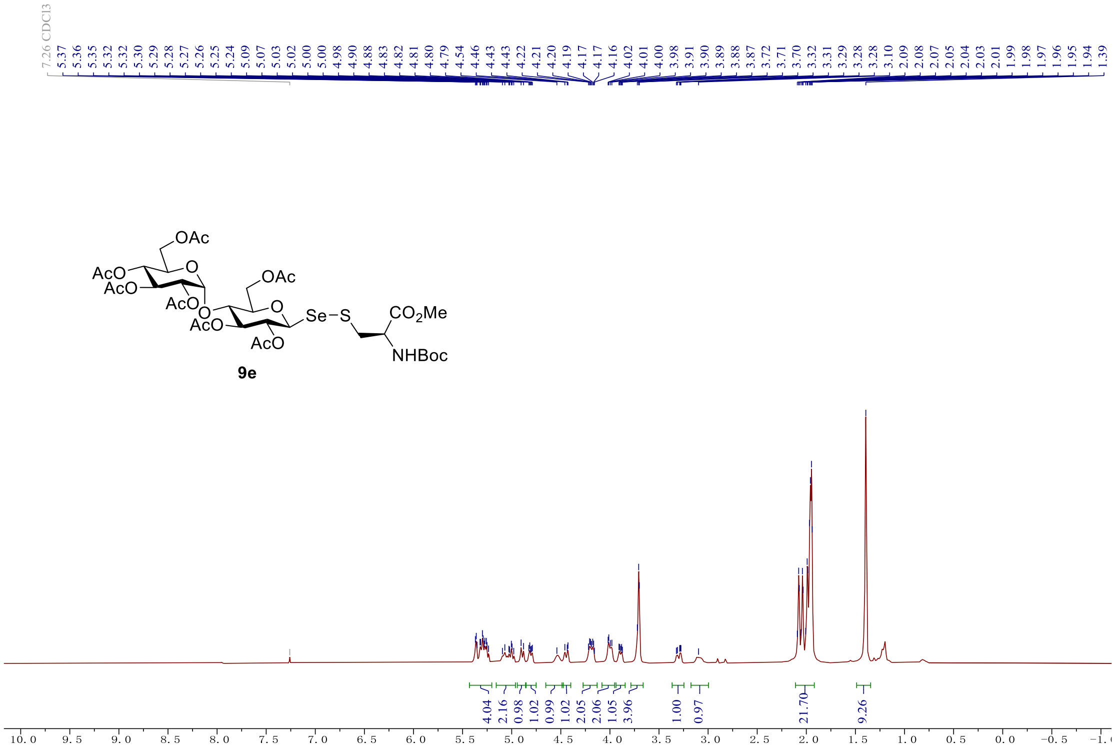

Figure S101. ^{1}H NMR (400 MHz, $CDCl_3$) Spectra for compound **9e**

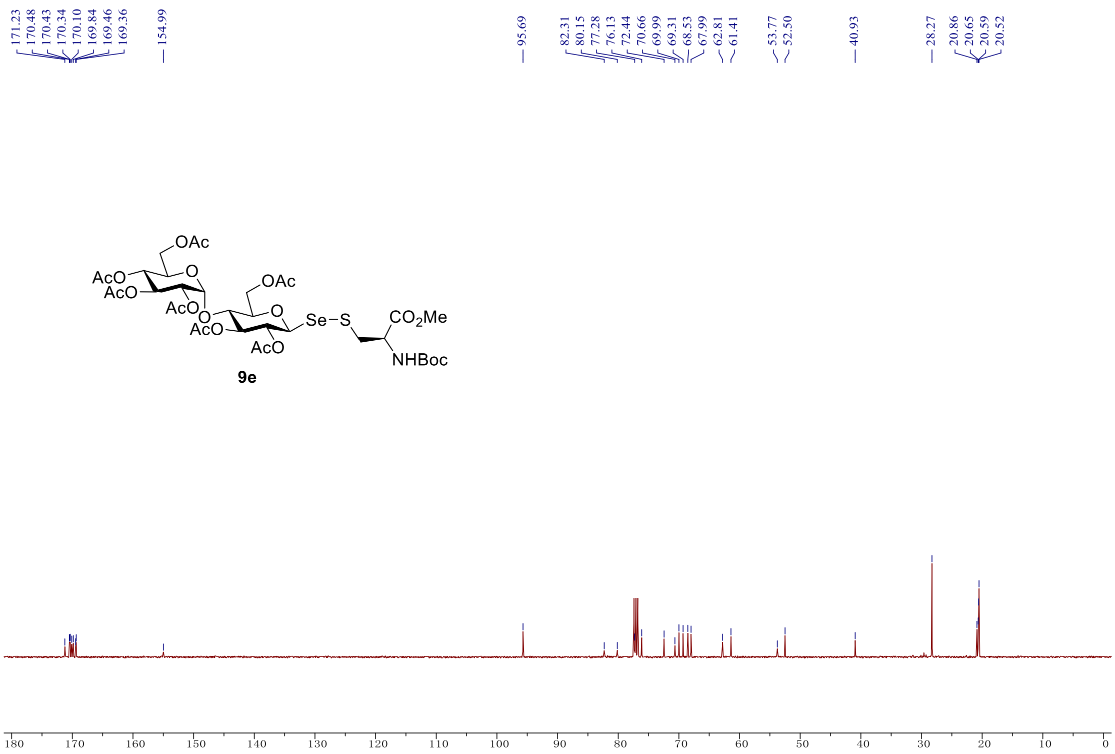

Figure S102. ^{13}C NMR (101 MHz, $CDCl_3$) Spectra for compound **9e**

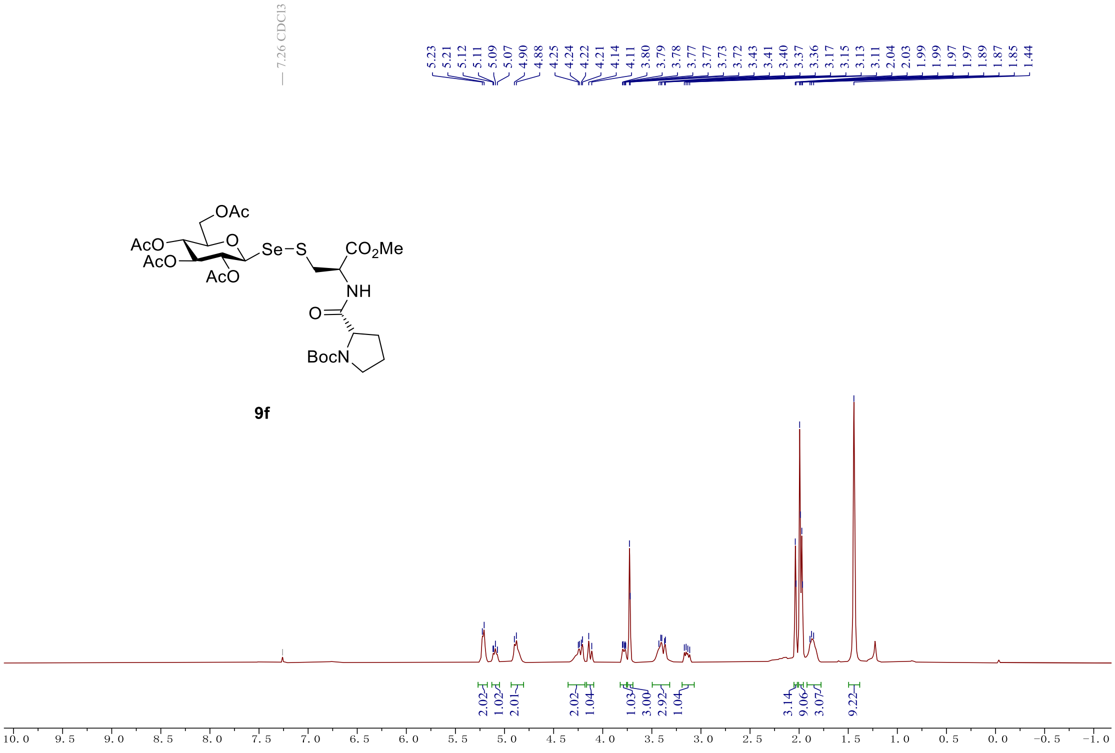

Figure S103. ^{1}H NMR (400 MHz, $CDCl_3$) Spectra for compound **9f**

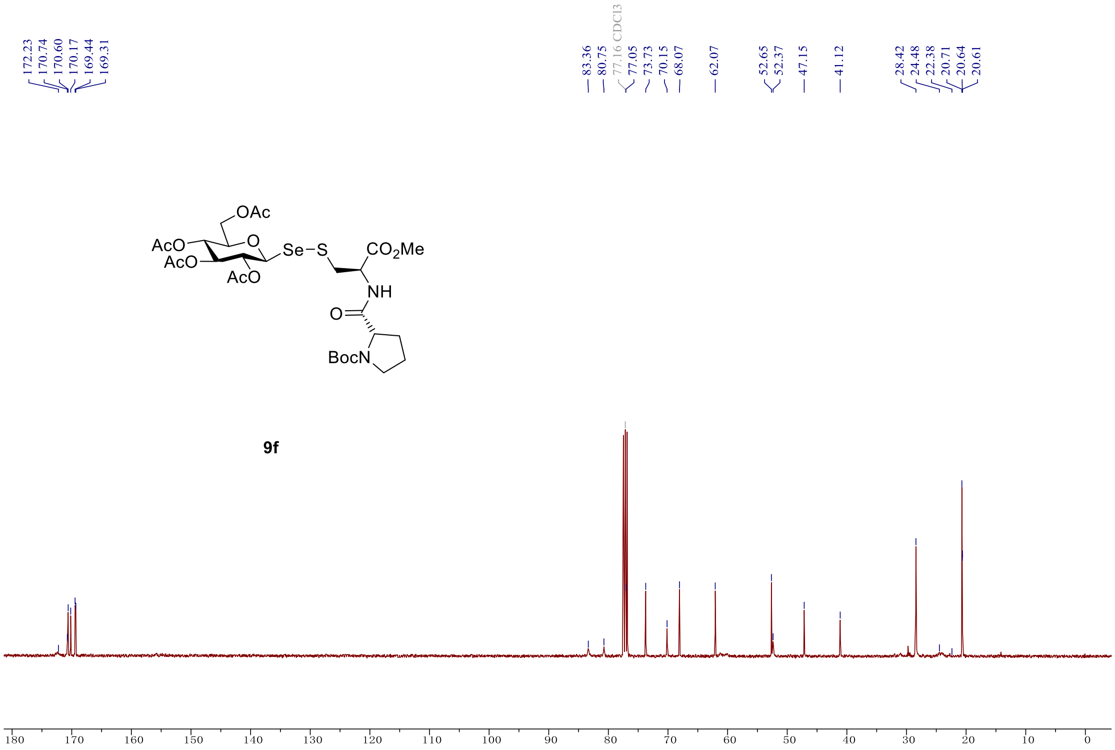

Figure S104. ^{13}C NMR (101 MHz, $CDCl_3$) Spectra for compound **9f**

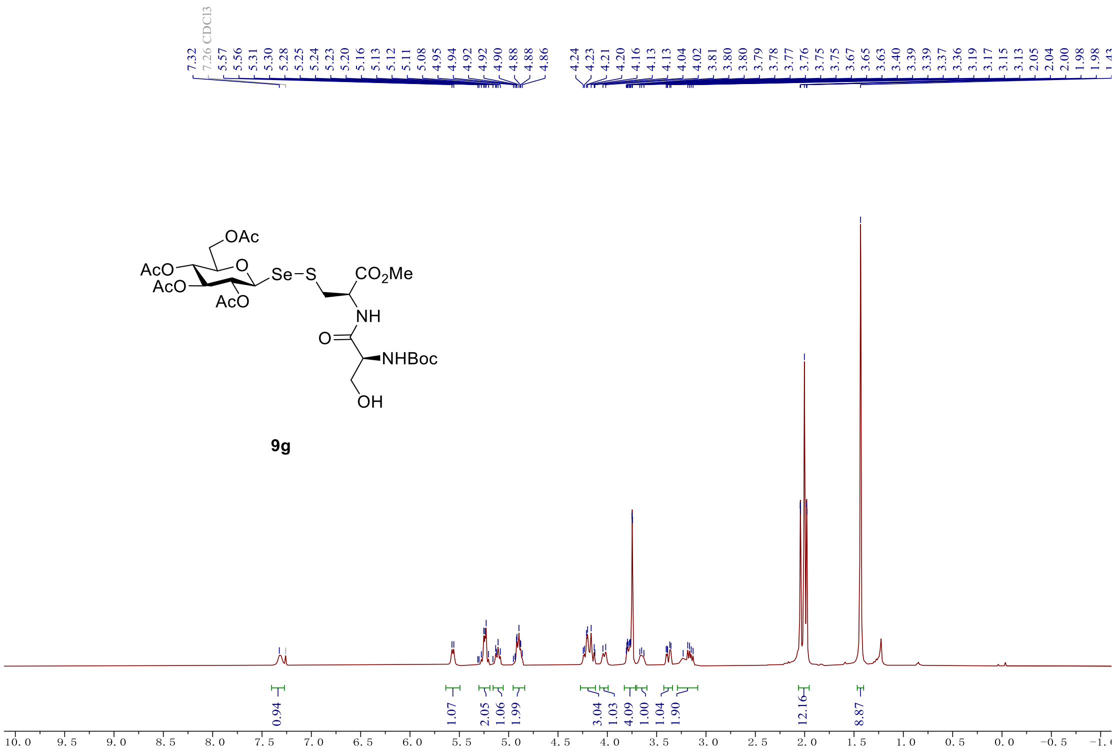

Figure S105. ^{1}H NMR (400 MHz, $CDCl_3$) Spectra for compound **9g**

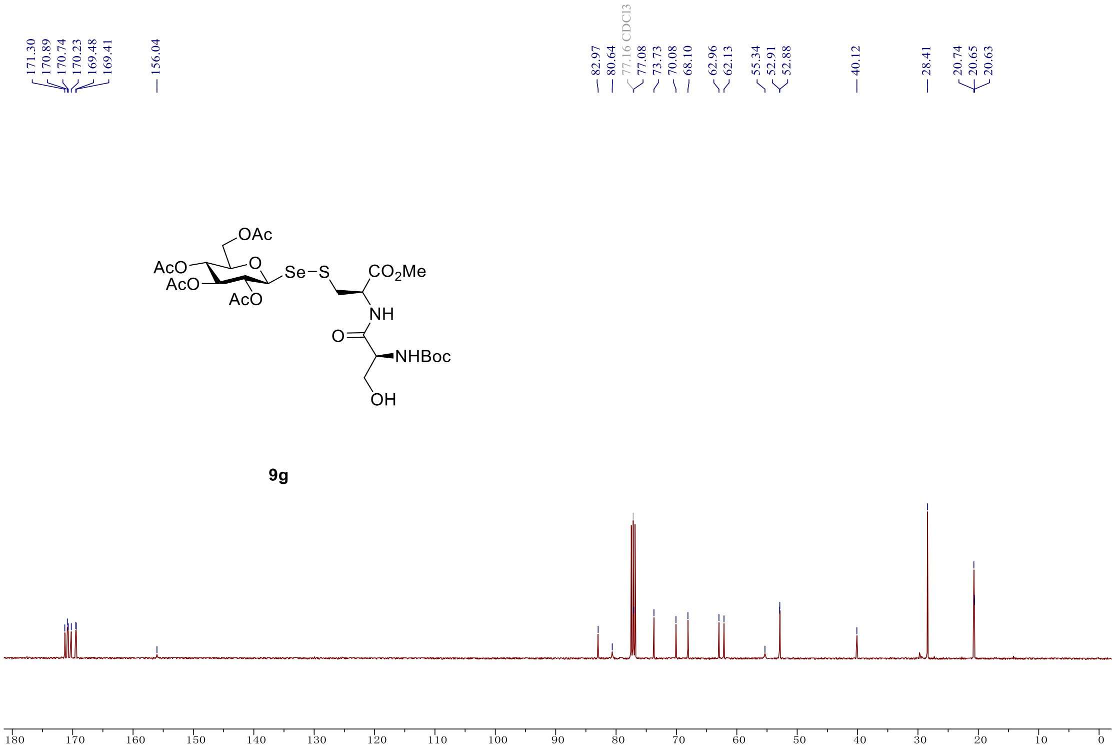

Figure S106. ^{13}C NMR (101 MHz, $CDCl_3$) Spectra for compound **9g**

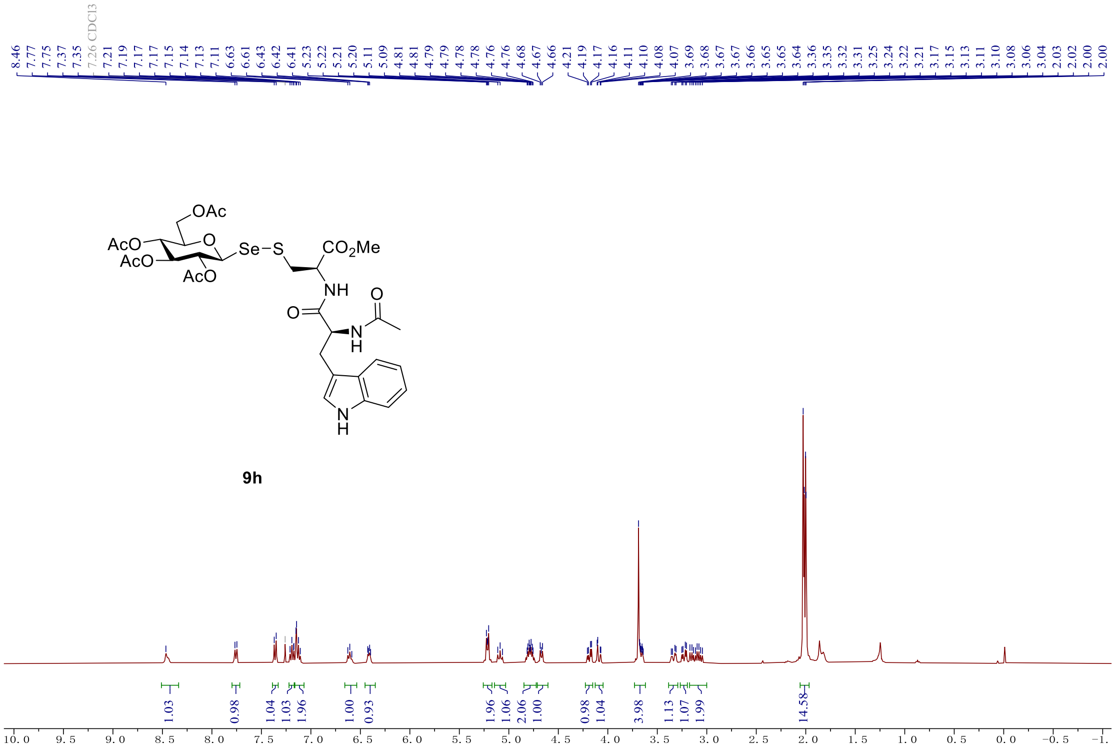

Figure S107. ^{1}H NMR (400 MHz, $CDCl_3$) Spectra for compound **9h**

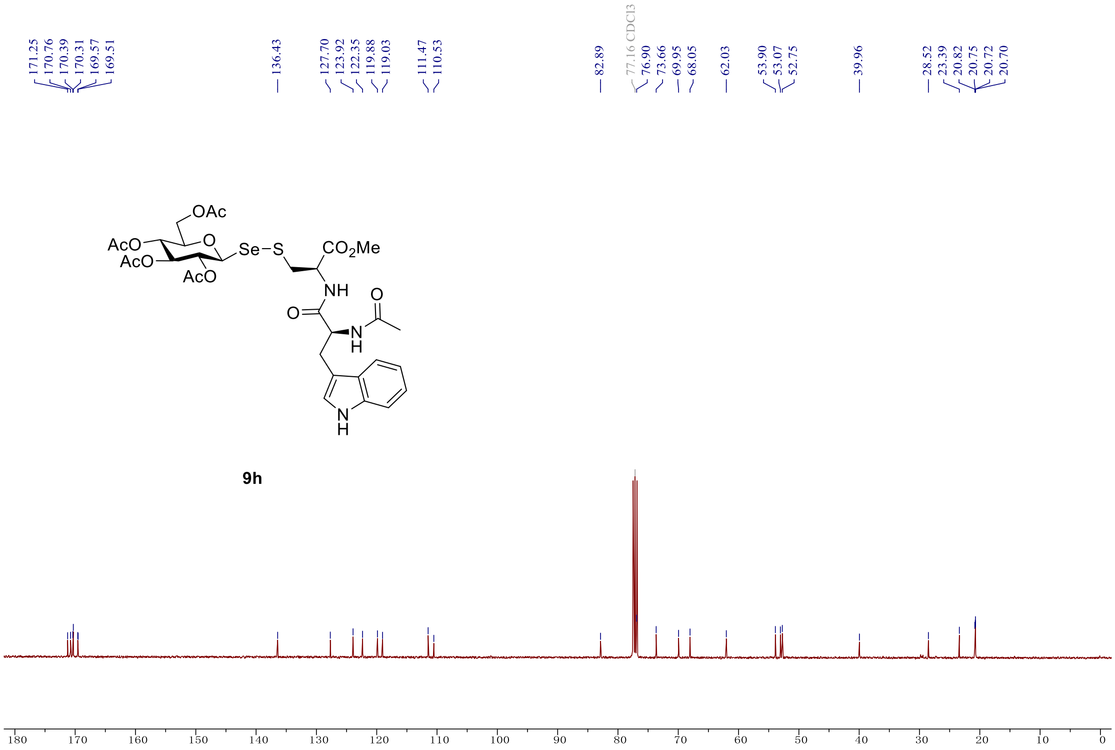

Figure S108. ^{13}C NMR (101 MHz, $CDCl_3$) Spectra for compound **9h**

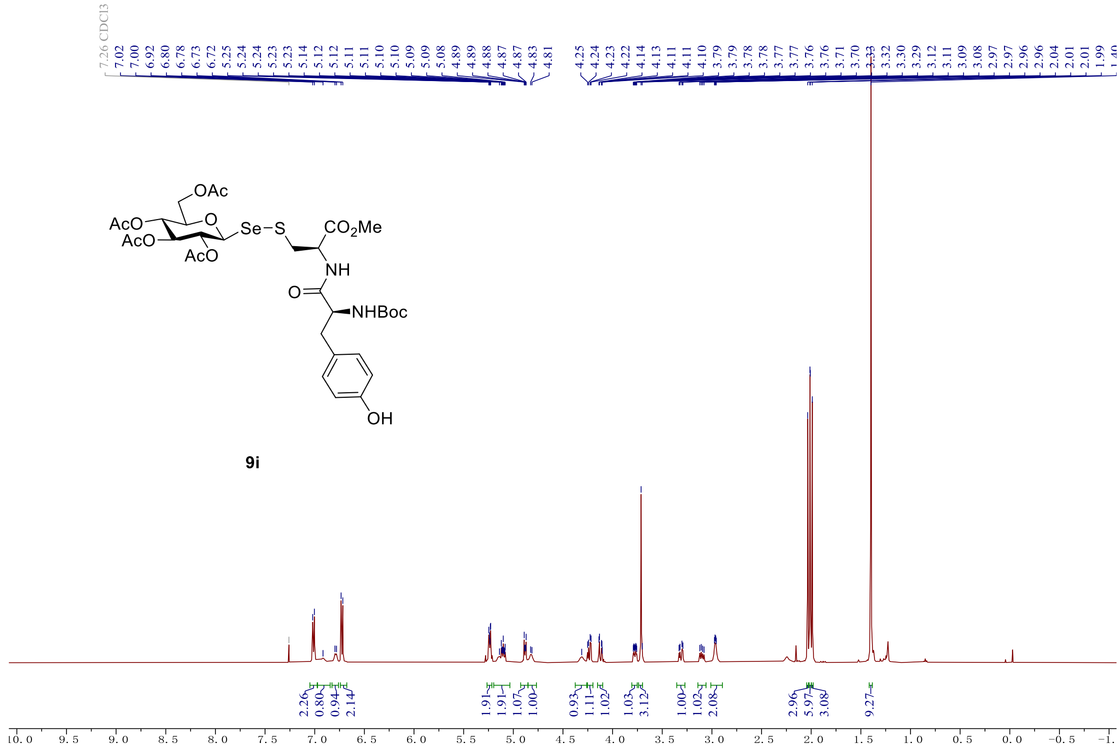

Figure S109. ^{1}H NMR (400 MHz, $CDCl_3$) Spectra for compound **9i**

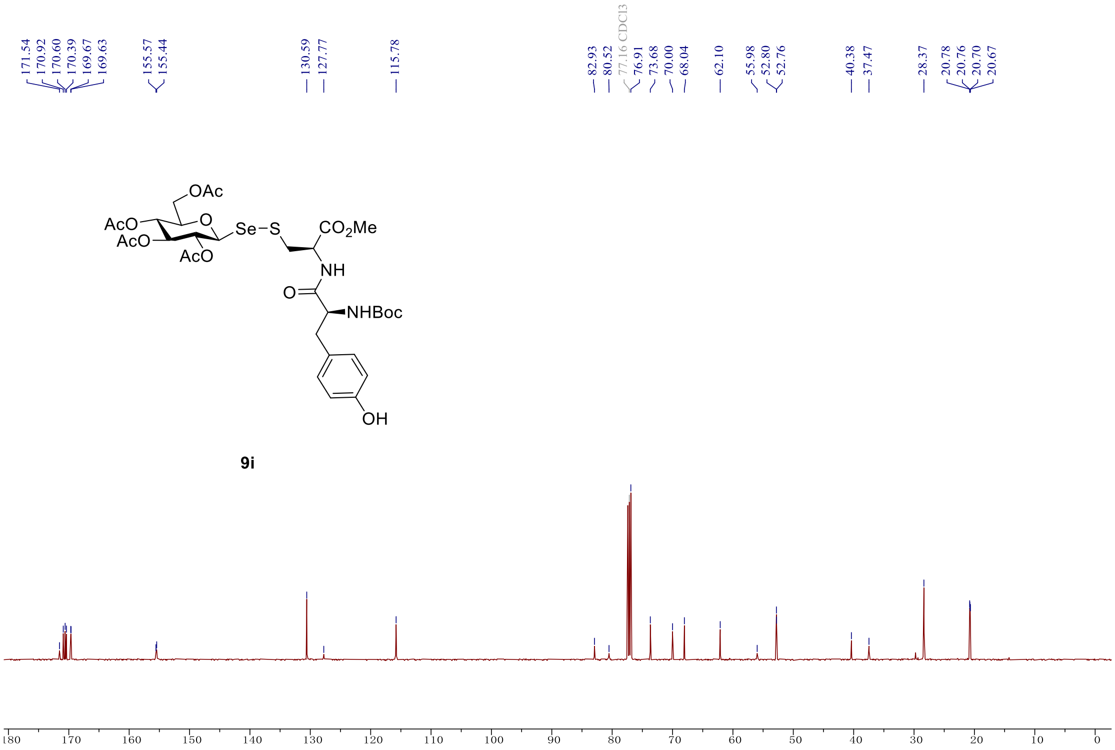

9i

Figure S110. ^{13}C NMR (101 MHz, $CDCl_3$) Spectra for compound **9i**
